# Enlightening the taxonomy darkness of human gut microbiomes with a cultured biobank

**DOI:** 10.1186/s40168-021-01064-3

**Published:** 2021-05-21

**Authors:** Chang Liu, Meng-Xuan Du, Rexiding Abuduaini, Hai-Ying Yu, Dan-Hua Li, Yu-Jing Wang, Nan Zhou, Min-Zhi Jiang, Peng-Xia Niu, Shan-Shan Han, Hong-He Chen, Wen-Yu Shi, Linhuan Wu, Yu-Hua Xin, Juncai Ma, Yuguang Zhou, Cheng-Ying Jiang, Hong-Wei Liu, Shuang-Jiang Liu

**Affiliations:** 1 State Key Laboratory of Microbial Resources, Institute of Microbiology, Chinese Academy of Sciences, No.1 Beichenxi Road, Chaoyang District, Beijing, 100101 PR China; 2 Environmental Microbiology Research Center, Institute of Microbiology, Chinese Academy of Sciences, No.1 Beichenxi Road, Chaoyang District, Beijing, 100101 China; 3 University of Chinese Academy of Sciences, Beijing, 100049 China; 4 Microbial Resources and Big Data Center, Institute of Microbiology, Chinese Academy of Sciences, No.1 Beichenxi Road, Chaoyang District, Beijing, 100101 China; 5 China General Microorganism Culture Collection, Institute of Microbiology, Chinese Academy of Sciences, No.1 Beichenxi Road, Chaoyang District, Beijing, 100101 China; 6 State Key Laboratory of Mycology, Institute of Microbiology, Chinese Academy of Sciences, No. 1 Beichenxi Road, Chaoyang District, Beijing, 100101 China

**Keywords:** Human gut microbiomes, Cultivation, Biobank, Novel taxa, hGMB

## Abstract

**Background:**

In gut microbiome studies, the cultured gut microbial resource plays essential roles, such as helping to unravel gut microbial functions and host-microbe interactions. Although several major studies have been performed to elucidate the cultured human gut microbiota, up to 70% of the Unified Human Gastrointestinal Genome species have not been cultured to date. Large-scale gut microbial isolation and identification as well as availability to the public are imperative for gut microbial studies and further characterizing human gut microbial functions.

**Results:**

In this study, we constructed a human Gut Microbial Biobank (hGMB; homepage: hgmb.nmdc.cn) through the cultivation of 10,558 isolates from 31 sample mixtures of 239 fresh fecal samples from healthy Chinese volunteers, and deposited 1170 strains representing 400 different species in culture collections of the International Depository Authority for long-term preservation and public access worldwide. Following the rules of the International Code of Nomenclature of Prokaryotes, 102 new species were characterized and denominated, while 28 new genera and 3 new families were proposed. hGMB represented over 80% of the common and dominant human gut microbial genera and species characterized from global human gut 16S rRNA gene amplicon data (*n* = 11,647) and cultured 24 “most-wanted” and “medium priority” taxa proposed by the Human Microbiome Project. We in total sequenced 115 genomes representing 102 novel taxa and 13 previously known species. Further in silico analysis revealed that the newly sequenced hGMB genomes represented 22 previously uncultured species in the Unified Human Gastrointestinal Genome (UHGG) and contributed 24 representatives of potentially “dark taxa” that had not been discovered by UHGG. The nonredundant gene catalogs generated from the hGMB genomes covered over 50% of the functionally known genes (KEGG orthologs) in the largest global human gut gene catalogs and approximately 10% of the “most wanted” functionally unknown proteins in the FUnkFams database.

**Conclusions:**

A publicly accessible human Gut Microbial Biobank (hGMB) was established that contained 1170 strains and represents 400 human gut microbial species. hGMB expands the gut microbial resources and genomic repository by adding 102 novel species, 28 new genera, 3 new families, and 115 new genomes of human gut microbes.

**Video abstract**

**Supplementary Information:**

The online version contains supplementary material available at 10.1186/s40168-021-01064-3.

## Introduction

The gut microbiome (GM) is recognized to be crucial to the host’s physical and mental health [1]. When GM dysbiosis occurs, it often induces host immune dysfunction [2], metabolic disorders [3], and impaired cognitive and physiological development [4]. Both culture-dependent and culture-independent studies have obtained unprecedented knowledge of GM diversities and functions [5–7]. Nevertheless, our understanding of human GMs is very limited. According to the most recent work of the Unified Human Gastrointestinal Genome (UHGG) [8], more than 70% of gut microbial species have not been cultured and 40% of the protein-coding sequences have no functional annotations [9, 10]. These unknown microorganisms and their genetic elements are called the “dark matters” of GMs and they hide secrets regarding GM functions and GM-host interactions [9, 11, 12]. To determine the identity and function of these “dark matters,” considerable effort has been made to develop bioinformatic tools and databases [13–16]. However, functional characterization and verification at the biological and molecular levels still rely on culture-based experiments. Cultured microbial resources that harbor unknown genes of interest and/or produce specific metabolites are indispensable. Furthermore, previous research showed that cultured gut microbial resources played fundamental roles not only in culture-dependent causative studies of host-GM interactions [17–19] but also in cultivation-independent omics studies [20–22]. Enlightening the “dark matters” of GMs requires extensive effort on microbial cultivation as well as physiological and genetic characterizations.

Over the past several years, several large-scale cultivation efforts have been made [20, 21, 23–28], and over 1500 microbial species have been cultured from those works. According to our and previous analyses, cultured human gut microbes accounted for 30–50% of the detected human gut microbial species from metagenomic and 16S rRNA gene amplicon datasets [8, 9, 22, 29], with the majority of gut microbes remaining uncultured. The valid taxonomic description and nomination of newly cultured gut microorganisms, on the other hand, lags even further behind [30]. For example, the Culturomics reported 247 novel taxa in 2016 [25], while 117 out of the 247 novel taxa remained unclassified until the time of this writing, as their taxonomic descriptions and nomenclatures did not fulfill the requirements of the International Code of Nomenclature of Prokaryotes (ICNP) [31]. In some other studies [20, 23, 24, 26], taxonomic characterization and nomenclature were absent. For newly isolated taxa without a valid description, their taxonomic names could not be validly approved by the International Committee on Systematics of Prokaryotes (ICSP) even though they were effectively published [30, 31], and their taxonomic information together with the 16S rRNA gene sequences could not be included by authoritative 16S rRNA gene sequence databases widely used for valid taxonomy classification as the EZBioCloud [32], NCBI [33] and SILVA All-species Living Tree Project (LTP) [16]. Consequently, some microbes have been repeatedly claimed to be novel in different cultivation-based studies. One example is that 54 microbial taxa first cultured in 2016 [23] were still considered as novel taxa in the works of 2019 [20, 21] (Table S1). The lack of valid taxonomic description and nomination of the newly cultured taxa complicated the scientific discourse of new microbes among researchers and impeded the accession and exchange of bacterial materials among scientific communities worldwide [34, 35]. The timely characterization and nomenclature of new bacterial isolates are highly important and of strong practical implications.

In this study, we cultured 10,558 bacterial isolates that represented 400 gut microbial species from 239 fecal samples of healthy donors through large-scale cultivation and deposited 1170 representative strains to culture collections of the International Depository Authority (IDA) for global public access. HGMB largely represented the taxonomic composition of the human gut microbial community. We sequenced 115 new bacterial genomes and denominated 102 new bacterial taxa. Data analysis revealed that the newly identified taxa are prevalent in the global human gut microbiome and illuminated a number of “dark taxa.”

## Results

### Construction of hGMB by large-scale bacterial cultivation and characterization

In total, 239 fresh fecal samples obtained from healthy Chinese volunteers were mixed into 32 sample mixtures (see Table S2 for the donor information for each mixture) and used for large-scale gut microbial isolation and cultivation, following a previously established workflow [36] and using 11 pretreatment methods and 67 different culture conditions (Tables S3 and S4). Single colonies on agar plates were collected and sequenced for 16S rRNA genes (> 1.4 kb). We harvested over 18,560 colonies, and 10,558 pure cultures were obtained (culture IDs and full-length 16S rRNA gene sequences are presented in Table S5). The taxonomy of these cultures was determined with BLAST analyses of their 16S rRNA genes against both the EZBioCloud and NCBI 16S ribosomal RNA sequence databases. The 10,558 cultures were phylogenetically grouped into 400 potential taxa at the species level by clustering with a 16S rRNA gene sequence identity threshold of 98.7% [37]. Then, 1170 representative strains of 400 taxa were selected as described in “Methods” for new-taxon characterization and long-term preservation. Out of the 400 taxa, 102 new taxa including 28 novel genera and 3 novel families were characterized and proposed according to the results of (1) phylogenetic analysis, (2) morphology observations, (3) BIOLOG tests, and (4) genomic analysis (see “Methods” for detailed criteria). All the new taxa were denominated following the rules of ICNP, and their protologs are provided in Table 1. More detailed descriptions of new taxa are documented in Supplementary Data 1. In this study, we sequenced 115 genomes, 102 of which represented the newly described taxa (Figure S1, blue color), 6 represented new strains of known species with 16S rRNA gene identities < 98.7% to the corresponding type strains (Figure S1 gray color) and 7 represented new strains of known species (16S rRNA gene identities > 98.7% to the type strains) with no genomes available in the NCBI database. The assembly quality of the 115 genomes (10 complete genomes and 105 draft genomes) was evaluated and is displayed in Table S6. The great majority of the 115 genomes were of good quality as the average completeness of assemblies reached 97.65 ± 5.50% (median value was 99.26%), the average contamination was 0.63 ± 0.96% (median value was 0.19%), and the mean value of the estimated quality score (completeness − 5 × contamination) was 94.49 ± 7.10% (median value was 96.22%). All the genomes are publicly accessible via public databases, such as the NCBI and the China National Microbiology Data Center (NMDC) (see “Availability of data and materials”).

**Table 1:** The protologs of 102 novel taxa in hGMB (rank*: “pebnv” indicated the nomenclatures that were **p**ublished **e**ffectively **b**ut **n**ot **v**alidly, the original publication of the nomenclature was cited after the proposed name; description*: more detailed descriptions are available in Supplementary Data 1)

Taxonomy	Rank*	Etymology	Type designation	Description*	GMCC/KCTC/NBRC accessions
*Yeguiaceae*	fam. nov.	Ye.gui'a.ce'ae. N. L. fem. n. *Yeguia*, type genus of the family. -aceae, ending to denote a family, N.L. fem. pl. n. *Yeguiaceae*, family of the genus *Yeguia*	Type genus: *Yeguia*	The same as for type genus	
*Yeguia*	gen. nov.	Ye.gui'a N.L. fem. n. *Yeguia,* named in honor of the Chinese medical scientist Gui Ye	Type species: *Yeguia hominis*	The same as for type species	
*Yeguia hominis*	sp. nov.	ho'mi.nis. L. gen. masc. n. *hominis*, of a human being, referring to the human gut habitat	NSJ-40^T^ from human feces	Cells are ovoid with peaked ends, non-motile. Growth in modified MGAM medium occurs at 37 °C, pH 7.0–7.5, in 5–10 days. The genomic DNA G+C content of the type strain is 63.58 mol%.	CGMCC 1.32813
*Luoshenia*	gen. nov.	Luo.shen'i.a. N.L. fem. n. *Luoshenia*, named after the Chinese Goddess Luoshen	Type species: *Luoshenia tenuis*	The same as for type species	
*Luoshenia tenuis*	sp. nov.	te'nu.is. L. fem. adj. *tenuis,* thin, slim, referring to the predicted potential function of the strain in weight-loss	NSJ-44^T^ from human feces	Cells are ovoid with spiky ends, non-motile. Growth in modified MGAM medium occurs at 37 °C, pH 7.0–7.5, in 3–10 days. The genomic DNA G+C content of the type strain is 61.02 mol%.	CGMCC 1.32817 /KCTC 25096
*Feifaniaceae*	fam. nov.	Fei.fa.ni.a.ce'ae. N.L. fem. n. *Feifania*, type genus of the family. -aceae, ending to denote a family. N.L. fem. pl. n. *Feifaniaceae*, family of the genus *Feifania*	Type genus: *Feifania*	The same as for type genus	
*Feifania*	gen. nov.	Fei.fa'ni.a. N.L. fem. n. *Feifania*, named after the Chinese microbiologist Feifan Tang	Type species: *Feifania hominis*	The same as for type species	
*Feifania hominis*	sp. nov.	ho'mi.nis. L. gen. masc. n. *hominis*, of a human being, referring to the human gut habitat	BX7^T^ from human feces	Cells are rod-shaped, motile. Growth in modified MGAM medium occurs at 37 °C, pH 7.0–7.5, in 3–12 days. The genomic DNA G+C content of the type strain is 58.80 mol%.	CGMCC 1.32862
*Bianqueaceae*	fam. nov.	Bian.que.a.ce'ae. N.L. fem. n. *Bianquea*, type genus of the family. -aceae, ending to denote a family. N.L. fem. pl. n. *Bianqueaceae*, family of the genus *Bianquea*	Type genus: *Bianquea*	The same as for type genus	
*Bianquea*	gen. nov.	Bian.que'a. N.L. fem. n. *Bianquea*, named after the Chinese medical scientist Bian Que	Type species: *Bianquea renquensis*	The same as for type species	
*Bianquea renqiuensis*	sp. nov.	ren.qu.en'sis. N.L. fem. adj. *renqiuensis*, pertaining Renqiu county of China, the birthplace of Chinese medical scientist QueBian	NSJ-32^T^ from human feces	Cells are rod-shaped, non-motile. Growth in modified MGAM medium occurs at 37 °C, pH 7.0–7.5, in 3–10 days. The genomic DNA G+C content of the type strain is 61.32 mol%.	CGMCC 1.32805
*Gehongia*	gen. nov.	Ge.hong'i.a. N.L. fem. n. *Gehongia*, named after Ge Hong (284-364 AD), a Chinese medical scientist	Type species: *Gehongia tenuis*	The same as for type species	
*Gehongia tenuis*	sp. nov.	te'nu.is. L. fem. adj. *tenuis*, thin, slim, referring to the predicted potential function of the strain in weight-loss	NSJ-53^T^ from human feces	Cells are rod-shaped, non-motile. Growth in modified MGAM medium occurs at 37 °C, pH 7.0–7.5, in 3–10 days. The genomic DNA G+C content of the type strain is 59.00 mol%.	CGMCC 1.32829 /KCTC 25141
*Guopingia*	gen. nov.	Guo.ping'i.a. N.L. fem. n. *Guopingia*, named after the Chinese microbiologist Guoping Zhao	Type species: *Guopingia tenuis*	The same as for type species	
*Guopingia tenuis*	sp. nov.	te'nu.is. L. fem. adj. *tenuis*, thin, slim, referring to the predicted potential function of the strain in weight-loss	NSJ-63^T^ from human feces	Cells are spherical, motile. Growth in modified MGAM medium occurs at 37 °C, pH 7.0–7.5, in 3–10 days. The genomic DNA G+C content of the type strain is53.30 mol%.	CGMCC 1.32839 /KCTC 25142
*Ligaoa*	gen. nov.	Li.gao'a. N.L. fem. n. *Ligaoa*, named in honor of the Chinese medical scientist Li Gao	Type species: *Ligaoa zhengdingensis*	The same as for type species	
*Ligaoa zhengdingensis*	sp. nov.	zheng.ding.en'sis. N.L. fem. adj. *zhengdingensis*, referring to Zhengding county of China, the birthplace of Li Gao	NSJ-31^T^ from human feces	Cells are spherical, non-motile. Growth in modified MGAM medium occurs at 37 °C, pH 7.0–7.5, in 3–10 days. The genomic DNA G+C content of the type strain is 64.87 mol%.	CGMCC 1.32804 /KCTC 25083
*Congzhengia*	gen. nov.	Cong.zheng'i.a. N.L. fem. n. *Congzhengia*, named after the Chinese medical scientist Congzheng Zhang	Type species: *Congzhengia minquanensis*	The same as for type species	
*Congzhengia minquanensis*	sp. nov.	min.quan.en'sis. N.L. fem. adj. *minquanensis*, referring to Minquan county of China, the birthplace of Congzheng Zhang	H8^T^ from human feces	Cells are spherical, non-motile. Growth in modified MGAM medium occurs at 37 °C, pH 7.0–7.5, in 3–10 days. The genomic DNA G+C content of the type strain is 50.19 mol%.	CGMCC 1.32875
*Fumia*	gen. nov.	Fu.mi'a. N.L. fem. n. *Fumia*, named in honor of the Chinese medical scientist Fumi Huang	Type species: *Fumia xinanensis*	The same as for type species	
*Fumia xinanensis*	sp. nov.	xin.an.en'sis. N.L. fem. adj. *xinanensis*, referring to Xin'an county where Fumi Huang was born	NSJ-33^T^ from human feces	Cells are rod-shaped or ovoid, non-motile. Growth in modified MGAM medium occurs at 37 °C, pH 7.0–7.5, in 3–10 days. The genomic DNA G+C content of the type strain is 51.32 mol%.	CGMCC 1.32806 /KCTC 25085
*Wujia*	gen. nov.	Wu.ji'a. N.L. fem. n. *Wujia*, named after the Chinese medical scientist Wuji	Type species: *Wujia chipingensis*	The same as for type species	
*Wujia chipingensis*	sp. nov.	chi.ping'en.sis. N.L. fem. adj. *chipingensis*, referring to Chiping county of China, the birthplace of the Chinese medical scientist Wuji Cheng	NSJ-4^T^ from human feces	Cells are rod-shaped, non-motile. Growth in modified MGAM medium occurs at 37 °C, pH 7.0–7.5, in 3–10 days. The genomic DNA G+C content of the type strain is 43.92 mol%.	CGMCC 1.52560
*Simiaoa*	gen. nov.	Si.miao'a. N.L. fem. n. *Simiaoa,* named after Sun Simiao, a Chinese medical scientist	Type species: *Simiaoa sunii*	The same as for type species	
*Simiaoa sunii*	sp. nov.	sun'i.i. N.L. gen. n. *sunii*, named after the family name of the Chinese medical scientist Simiao Sun	NSJ-8^T^ from human feces	Cells are rod-shaped, non-motile. Growth in modified MGAM medium occurs at 37 °C, pH 7.0–7.5, in 3–10 days. The genomic DNA G+C content of the type strain is 45.91 mol%.	CGMCC 1.52840
*Simiaoa hominis*	sp. nov.	ho'mi.nis. L. gen. masc. n. *hominis*, of a human being, referring to the human gut habitat	H15^T^ from human feces	Cells are rod-shaped, non-motile. Growth in modified MGAM medium occurs at 37 °C, pH 7.0–7.5, in 3–10 days. The genomic DNA G+C content of the type strain is 46.50 mol%.	CGMCC 1.32863
*Jutongia*	gen. nov.	Ju.tong'ia. N.L. fem. n. *Jutongia*, in honor of the Chinese medical scientist Jutong Wu	Type species: *Jutongia huaianensis*	The same as for type species	
*Jutongia hominis*	sp. nov.	ho'mi.nis. L. gen. masc. n. *hominis*, of a human being, referring to the human gut habitat	BX3^T^ from human feces	Cells are rod-shaped with blunt ends, non-motile. Growth in modified MGAM medium occurs at 37 °C, pH 7.0–7.5, in 3–10 days. The genomic DNA G+C content of the type strain is 38.60 mol%.	CGMCC 1.32876
*Jutongia huaianensis*	sp. nov.	huai.an.en'sis. N.L. fem. adj. *huaianensis*, referring to huai'an county of China, the birthplace of the Chinese medical scientist Jutong Wu	NSJ-37^T^ from human feces	Cells are straight rod-shaped, motile. Growth in modified MGAM medium occurs at 37 °C, pH 7.0–7.5, in 3–10 days. The genomic DNA G+C content of the type strain is 51.41 mol%.	CGMCC 1.32810 /KCTC 25089
*Qiania*	gen. nov.	Qian'i.a. N.L. fem. n. *Qiania*, named after the Chinese medical scientist Yi Qian	Type species: *Qiania dongpingensis*	The same as for type species	
*Qiania dongpingensis*	sp. nov.	dong.ping.en'sis. N.L. fem. adj. *dongpingensis*, referring to Dongping county of China, the birthplace of Yi Qian	NSJ-38^T^ from human feces	Cells are ovoid to rod-shaped with tapered ends, non-motile. Growth in modified MGAM medium occurs at 37 °C, pH 7.0–7.5, in 3–10 days. The genomic DNA G+C content of the type strain is 49.22 mol%.	CGMCC 1.32811
*Zhenhengia*	gen. nov.	Zhen.heng'i.a. N.L. fem. n. *Zhenhengia*, named after the Chinese medical scientist Zhenheng Zhu	Type species: *Zhenhengia yiwuensis*	The same as for type species	
*Zhenhengia yiwuensis*	sp. nov.	yi.wu.en'sis. N.L. fem. adj. *yiwuensis*, referring to Yiwu city of China, where Zhenheng Zhu was born	NSJ-12^T^ from human feces	Cells are straight rod-shaped, non-motile. Growth in modified MGAM medium occurs at 37 °C, pH 7.0–7.5, in 3–10 days. The genomic DNA G+C content of the type strain is 53.19 mol%.	CGMCC 1.32465 /KCTC 15954
*Jingyaoa*	gen. nov.	Jing.yao'a. N.L. fem. n. *Jingyaoa*, named after the Chinese medical scientist Jingyao Zhang.	Type species: *Jingyaoa shaoxingensis*	The same as for type species	
*Jingyaoa shaoxingensis*	sp. nov.	shao.xing'en.sis. N.L. fem. adj. *shaoxingensis*, referring to Shaoxing city of China, where Jingyao Zhang was born	NSJ-46^T^ from human feces	Cells are spherical or ovoid or short rod-shaped, non-motile. Growth in modified MGAM medium occurs at 37 °C, pH 7.0–7.5, in 3–10 days. The genomic DNA G+C content of the type strain is 54.52 mol%.	CGMCC 1.32819
*Wansuia*	gen. nov.	Wan.su'i.a. N.L. adj. fem., *Wansuia*, in honor of the Chinese medical scientist Wansu Liu	Type species: *Wansuia hejianensis*	The same as for type species	
*Wansuia hejianensis*	sp. nov.	he.jian.en'sis. N.L. fem. adj. *hejianesis*, referring to Hejian county of China, the birthplace of the Chinese medical scientist Wansu Liu	NSJ-29^T^ from human feces	Cells are ovoid to rod-shaped with spiky ends, non-motile. Growth in modified MGAM medium occurs at 37 °C, pH 7.0–7.5, in 3–10 days. The genomic DNA G+C content of the type strain is 49.35 mol%.	CGMCC 1.32802 /KCTC 25078
*Zhenpiania*	gen. nov.	Zhen.pian'i.a. N.L. fem. n. *Zhenpiania*, named after the Chinese medical scientist Zhenpian Li	Type species: *Zhenpiania hominis*	The same as for type species	
*Zhenpiania hominis*	sp. nov.	ho'mi.nis. L. gen. masc. n. *hominis*, of a human being, referring to the human gut habitat	BX12^T^ from human feces	Cells are rod-shaped, non-motile. Growth in modified MGAM medium occurs at 37 °C, pH 7.0–7.5, in 3–10 days. The genomic DNA G+C content of the type strain is 47.50 mol%.	CGMCC 1.32877
*Lentihominibacter*	gen. nov.	Len.ti.ho.mi.ni.bac'ter. L. masc. n. *lentus*, slow. L. masc. n. *homo*, a man. L. masc. n. *bacter*, a rod. N.L. masc. n. *Lentihominibacter*, slowly growing rod-shaped bacterium from humans	Type species: *Lentihominibacter hominis*	The same as for type species	
*Lentihominibacter faecis*	sp. nov.	fae'cis. L. gen. fem. n. *faecis*, of feces, from which the organism was isolated	BX16^T^ from human feces	Cells are rod-shaped, non-motile. Growth in modified MGAM medium occurs at 37 °C, pH 7.0–7.5, in 3–10 days. The genomic DNA G+C content of the type strain is 47.60 mol%.	CGMCC 1.32878
*Lentihominibacter hominis*	sp. nov.	ho'mi.nis. L. gen. masc. n. *hominis*, of a human being, referring to the human gut habitat	NSJ-24^T^ from human feces	Cells are rod-shaped, non-motile. Growth in modified MGAM medium occurs at 37 °C, pH 7.0–7.5, in 3–10 days. The genomic DNA G+C content of the type strain is 49.12 mol%.	CGMCC 1.32874 /KCTC 25076
*Yanshouia hominis*	sp. nov.	ho'mi.nis. L. gen. masc. n. *hominis*, of a human being, referring to the human gut habitat	BX1^T^ from human feces	Cells are rod-shaped, motile. Growth in modified MGAM medium occurs at 37 °C, pH 7.0–7.5, in 3–10 days. The genomic DNA G+C content of the type strain is 56.30 mol%.	CGMCC 1.32879
*Shuzhengia*	gen. nov.	Shu.zheng'i.a. N.L. fem. n. *Shuzhengia*, named after the Chinese microbiologist Shuzheng Zhang	Type species: *Shuzhengia hominis*	The same as for type species	
*Shuzhengia hominis*	sp. nov.	ho'mi.nis. L. gen. masc. n. *hominis*, of a human being, referring to the human gut habitat	BX18^T^ from human feces	Cells are rod-shaped, non-motile. Growth in modified MGAM medium occurs at 37 °C, pH 7.0–7.5, in 3–10 days. The genomic DNA G+C content of the type strain is 45.40 mol%.	CGMCC 1.32880
*Anaerofilum hominis*	sp. nov.	ho'mi.nis. L. gen. masc. n. *hominis*, of a human being, referring to the human gut habitat	BX8^T^ from human feces	Cells are rod-shaped, motile. Growth in modified MGAM medium occurs at 37 °C, pH 7.0–7.5, in 3–10 days. The genomic DNA G+C content of the type strain is 61.60 mol%.	CGMCC 1.32881 /KCTC 25176
*Zongyangia*	gen. nov.	Zong.yang'i.a. N.L. fem. n. *Zongyangia*, named after the Chinese medical scientist Zongyang Yang	Type species: *Zongyangia hominis*	The same as for type species	
*Zongyangia hominis*	sp. nov.	ho'mi.nis. L. gen. masc. n. *hominis*, of a human being, referring to the human gut habitat	NSJ-54^T^ from human feces	Cells are rod-shaped, non-motile. Growth in modified MGAM medium occurs at 37 °C, pH 7.0–7.5, in 3–10 days. The genomic DNA G+C content of the type strain is 56.40 mol%.	CGMCC 1.32830 /KCTC 25132
*Youxingia*	gen. nov.	You.xing'i.a. N.L. fem. n. *Youxingia*, named after the Chinese medical scientist Youxing Wu	Type species: *Youxingia wuxianesis*	The same as for type species	
*Youxingia wuxianensis*	sp. nov.	wu.xian.en'sis. N.L. fem. adj. *wuxianensis*, referring to the Wuxian county of China, where Youxing Wu was born	NSJ-64^T^ from human feces	Cells are ovoid to rod-shaped, non-motile. Growth in modified MGAM medium occurs at 37 °C, pH 7.0–7.5, in 3–10 days. The genomic DNA G+C content of the type strain is 58.41 mol%.	CGMCC 1.32840 /KCTC 25128
*Qingrenia*	gen. nov.	Qing.re'ni.a. N.L. fem. n. *Qingrenia*, named after the Chinese medical scientist Qingren Wang	Type species: *Qingrenia yutianensis*	The same as for type species	
*Qingrenia yutianensis*	sp. nov.	yu.tian.en'sis. N.L. fem. adj. *yutianensis*, referring to Yutian county of China, where Qingren Wang was born	NSJ-50^T^ from human feces	Cells are ovoid with spiky ends, non-motile. Growth in modified MGAM medium occurs at 37 °C, pH 7.0–7.5, in 3–10 days. The genomic DNA G+C content of the type strain is 55.26 mol%.	CGMCC 1.32823
*Jilunia*	gen. nov.	Ji.lun'i.a. N.L. fem. n. *Jilunia*, named after the Chinese microbiologist Jilun Li	Type species: *Jilunia laotingensis*	The same as for type species	
*Jilunia laotingensis*	sp. nov.	lao.ting.en'sis. N.L. fem. adj. *laotingensis*, referring to the Laoting county where Jilun Li was born	N12^T^ from human feces	Cells are spherical or ovoid or short rod-shaped, non-motile. Growth in modified MGAM medium occurs at 37 °C, pH 7.0–7.5, in 3–10 days. The genomic DNA G+C content of the type strain is 41.64 mol%.	CGMCC 1.32860
*Paratissierella*	gen. nov.	Pa.ra.tis.sier.el'la. Gr. prep. *para*, beside. N.L. fem. dim. n. *Tissierella*, a genus name. N.L. fem. n. *Paratissierella*, resembling the genus *Tissierella*	Type species: *Paratissierella segnis*	The same as for type species	
*Paratissierella segnis*	sp. nov.	seg'nis. L. fem. adj. *segnis*, slow, inactive, lazy, referring to the slow growth of the strain	BX21^T^ from human feces	Cells are rod-shaped, motile. Growth in modified MGAM medium occurs at 37 °C, pH 7.0–7.5, in 3–10 days. The genomic DNA G+C content of the type strain is 33.30 mol%.	CGMCC 1.32882
*Bittarella* (ex Durand et al. 2017)	pebnv	Bit.ta.rel'la. N.L. fem. dim. n. *Bittarella*, in honor of Dr Bittar, a French microbiologist [38]	Type species: *Bittarella massiliensis*	The same as for type species	
*Bittarella massiliensis* (ex Durand et al. 2017)	pebnv	mas.sil.i.en'sis L. fem. adj. *massiliensis*, of Massilia, the Latin name of Marseille where the strain was for the first time isolated [38]	NSJ-19^T^ from human feces	Cells are rod-shaped, non-motile. Growth in modified MGAM medium occurs at 37 °C, pH 7.0–7.5, in 3–10 days. The genomic DNA G+C content of the type strain is 71.15 mol%.	CGMCC 1.32824 /KCTC 25133
*Eggerthella hominis*	sp. nov.	ho'mi.nis. L. gen. masc. n. *hominis*, of a human being, referring to the human gut habitat	NSJ-70^T^ from human feces	Cells are straight rod-shaped, non-motile. Growth in modified MGAM medium occurs at 37 °C, pH 7.0–7.5, in 3–10 days. The genomic DNA G+C content of the type strain is 70.44 mol%.	CGMCC 1.32846 /KCTC 25139
*Gordonibacter massiliensis* (ex Ngom et al. 2020)	pebnv	mas.si.li.en'sis. L. adj. masc. *massiliensis*, of Massilia, Marseille, where the bacterium was for the first time isolated [39]	NSJ-58^T^ from human feces	Cells are rod-shaped, non-motile. Growth in modified MGAM medium occurs at 37 °C, pH 7.0–7.5, in 3–10 days. The genomic DNA G+C content of the type strain is 76.43 mol%.	CGMCC 1.32834 /KCTC 25146
*Bacteroides multiformis*	sp. nov.	mul.ti.for'mis. L. masc. adj. *multiformis*, many-shaped, multiform, referring to the various size and shape of the strain	L5^T^ from human feces	Cells are spherical or ovoid or rod-shaped, non-motile. Growth in modified MGAM medium occurs at 37 °C, pH 7.0–7.5, in 3–10 days. The genomic DNA G+C content of the type strain is 56.50 mol%.	CGMCC 1.32865
*Bacteroides facilis*	sp. nov.	L. masc. adj. *facilis*, easy, referring that the type strain is easily cultured	NSJ-77^T^ from human feces	Cells are rod-shaped in various sizes, non-motile. Growth in modified MGAM medium occurs at 37 °C, pH 7.0–7.5, in 1–3 days. The genomic DNA G+C content of the type strain is 52.58 mol%.	CGMCC 1.32853 /KCTC 25155
*Bacteroides difficilis*	sp. nov.	dif.fi'ci.lis. L. masc. adj. *difficilis*, difficult, referring to the difficulty of culturing the strain	NSJ-74^T^ from human feces	Cells are ovoid or short rod-shaped, non-motile. Growth in modified MGAM medium occurs at 37 °C, pH 7.0–7.5, in 3–10 days. The genomic DNA G+C content of the type strain is 48.67 mol%.	CGMCC 1.32850
*Bacteroides hominis*	sp. nov.	ho'mi.nis. L. gen. masc. n. *hominis*, of a human being, referring to the human gut habitat	NSJ-2^T^ from human feces	Cells are spherical or ovoid or short rod-shaped, non-motile. Growth in modified MGAM medium occurs at 37 °C, pH 7.0–7.5, in 3–10 days. The genomic DNA G+C content of the type strain is 48.67 mol%.	CGMCC 1.31481 /KCTC 15964
*Bacteroides parvus*	sp. nov.	par'vus. L. masc. adj. *parvus*, small, referring that its colonies on MGAM agar media are significantly small.	NSJ-21^T^ from human feces	Cells are ovoid to rod-shaped with round or blunt ends, non-motile. Growth in modified MGAM medium occurs at 37 °C, pH 7.0–7.5, in 3–10 days. The genomic DNA G+C content of the type strain is 46.32 mol%.	CGMCC 1.31612 /KCTC 25073
*Barnesiella faecis*	sp. nov.	fae'cis. L. gen. fem. n. *faecis*, of feces, from which the organism was isolated	BX6^T^ from human feces	Cells are straight or slightly curved rod-shaped, non-motile. Growth in modified MGAM medium occurs at 38 °C, pH 7.0–7.5, in 3–10 days. The genomic DNA G+C content of the type strain is 66.00% mol%.	CGMCC 1.32883
*Butyricimonas hominis*	sp. nov.	ho'mi.nis. L. gen. masc. n. *hominis*, of a human being, referring to the human gut habitat	NSJ-56^T^ from human feces	Cells are ovoid or short rod-shaped, non-motile. Growth in modified MGAM medium occurs at 37 °C, pH 7.0–7.5, in 3–10 days. The genomic DNA G+C content of the type strain is 60.54 mol%.	CGMCC 1.32832
*Parabacteroides acidifaciens*	sp. nov.	a.ci.di.fa'ci.ens. L. neut. n. *acidum*, acid; L. v. *facio*, to produce; N.L. part. adj. *acidifaciens*, acid-producing	426-9^T^ from human feces	Cells are rod-shaped, non-motile. Growth in modified MGAM medium occurs at 38 °C, pH 7.0–7.5, in 1–3 days. The genomic DNA G+C content of the type strain is 45.90 mol%.	CGMCC 1.13558 /NBRC 113433
*Parabacteroides segnis*	sp. nov.	seg'nis. L. masc. adj. *segnis*, slow, inactive, lazy, referring to the slow growth of the strain	BX2^T^ from human feces	Cells are rod-shaped, motile. Growth in modified MGAM medium occurs at 37 °C, pH 7.0–7.5, in 3–10 days. The genomic DNA G+C content of the type strain is 43.00 mol%.	CGMCC 1.32884
*Parabacteroides hominis*	sp. nov.	ho'mi.nis. L. gen. masc. n. *hominis*, of a human being, referring to the human gut habitat	NSJ-79^T^ from human feces	Cells are rod-shaped, motile. Growth in modified MGAM medium occurs at 37 °C, pH 7.0–7.5, in 3–10 days. The genomic DNA G+C content of the type strain is 59.47 mol%.	CGMCC 1.32855 /KCTC 25129
*Alistipes hominis*	sp. nov.	ho'mi.nis. L. gen. masc. n. *hominis*, of a human being, referring to the human gut habitat	New-7^T^ from human feces	Cells are ovoid to short rod-shaped, non-motile. Growth in modified MGAM medium occurs at 37 °C, pH 7.0–7.5, in 3–10 days. The genomic DNA G+C content of the type strain is 58.63 mol%.	CGMCC 1.31637 /KCTC 15866
*Ornithinibacillus hominis*	sp. nov.	ho'mi.nis. L. gen. masc. n. *hominis*, of a human being, referring to the human gut habitat	BX22^T^ from human feces	Cells are rod-shaped, motile. Growth in modified MGAM medium occurs at 37 °C, pH 7.0–7.5, in 3–10 days. The genomic DNA G+C content of the type strain is 37.10 mol%.	CGMCC 1.32885
*Streptococcus hominis*	sp. nov.	ho'mi.nis. L. gen. masc. n. *hominis*, of a human being, referring to the human gut habitat	NSJ-17^T^ from human feces	Cells are ovoid to short rod-shaped, non-motile. Growth in modified MGAM medium occurs at 37 °C, pH 7.0–7.5, in 3–10 days. The genomic DNA G+C content of the type strain is 49.41 mol%.	CGMCC 1.32470 /KCTC 15949
*Christensenella tenuis*	sp. nov.	te'nu.is. L. fem. adj. *tenuis*, thin, slim, referring to the predicted potential function of the strain in weight-loss	NSJ-35^T^ from human feces	Cells are rod-shaped with spiky ends, non-motile. Growth in modified MGAM medium occurs at 37 °C, pH 7.0–7.5, in 3–10 days. The genomic DNA G+C content of the type strain is 55.54 mol%.	CGMCC 1.32808 /KCTC 25087
*Clostridium hominis*	sp. nov.	ho'mi.nis. L. gen. masc. n. *hominis*, of a human being, referring to the human gut habitat	NSJ-6^T^ from human feces	Cells are ovoid to rod-shaped, non-motile. Growth in modified MGAM medium occurs at 37 °C, pH 7.0–7.5, in 3–10 days. The genomic DNA G+C content of the type strain is 53.42 mol%.	CGMCC 1.32461 /KCTC 15960
*Clostridium lentum*	sp. nov.	len'tum. L. neut. adj. *lentum*, slow, referring to the slow growth of the type strain	NSJ-42^T^ from human feces	Cells are rod-shaped, non-motile. Growth in modified MGAM medium occurs at 37 °C, pH 7.0–7.5, in 3–10 days. The genomic DNA G+C content of the type strain is 52.68 mol%.	CGMCC 1.32815 /KCTC 25094
*Clostridium facile*	sp. nov.	fa'ci.le. L. neut. adj. *facile*, easy, without difficulty, referring that the type strain is easily cultured	NSJ-27^T^ from human feces	Cells are rod-shaped, non-motile. Growth in modified MGAM medium occurs at 37 °C, pH 7.0–7.5, in 1–3 days. The genomic DNA G+C content of the type strain is 48.02 mol%.	CGMCC 1.32800 /KCTC 25079
*Anaerosacchariphilus hominis*	sp. nov.	ho'mi.nis. L. gen. masc. n. *hominis*, of a human being, referring to the human gut habitat	NSJ-68^T^ from human feces	Cells are rod-shaped, non-motile. Growth in modified MGAM medium occurs at 37 °C, pH 7.0–7.5, in 3–10 days. The genomic DNA G+C content of the type strain is 54.45 mol%.	CGMCC 1.32844 /KCTC 25150
*Anaerostipes hominis*	sp. nov.	ho'mi.nis. L. gen. masc. n. *hominis*, of a human being, referring to the human gut habitat	NSJ-7^T^ from human feces	Cells are rod-shaped, non-motile. Growth in modified MGAM medium occurs at 37 °C, pH 7.0–7.5, in 3–10 days. The genomic DNA G+C content of the type strain is 49.83 mol%.	CGMCC 1.32462 /KCTC 15959
*Blautia massiliensis* (ex Durand et al. 2017)	.pebnv	mas.si.li.en'sis. L. fem. adj. *massiliensis*, of Massilia, the Latin name of Marseille, where the bacterium was for the first time isolated [40]	4-46^T^ from human feces	Cells are rod-shaped, non-motile. Growth in modified MGAM medium occurs at 37 °C, pH 7.0–7.5, in 3–10 days. The genomic DNA G+C content of the type strain is 54.53 mol%.	CGMCC 1.52830 /NBRC 113773
*Blautia intestinalis*	sp. nov.	in.tes.ti.na'lis. N.L. fem. adj. *intestinalis*, pertaining to the intestines where the type strain inhabits	27-44^T^ from human feces	Cells are rod-shaped, non-motile. Growth in modified MGAM medium occurs at 37 °C, pH 7.0–7.5, in 3–10 days. The genomic DNA G+C content of the type strain is 54.62 mol%.	CGMCC 1.52850 /NBRC 113774
*Blautia segnis*	sp. nov.	seg'nis. L. fem. adj. *segnis*, slow, inactive, lazy, referring to the slow growth of the strain	BX17^T^ from human feces	Cells are rod-shaped, non-motile. Growth in modified MGAM medium occurs at 37 °C, pH 7.0–7.5, in 3–10 days. The genomic DNA G+C content of the type strain is 44.70 mol%.	CGMCC 1.32886
*Blautia tarda*	sp. nov.	tar'da. L. fem. adj. *tarda*, slow, inactive, lazy, referring to the slow growth of the strain	BX19^T^ from human feces	Cells are rod-shaped with tapered ends, non-motile. Growth in modified MGAM medium occurs at 37 °C, pH 7.0–7.5, in 3–10 days. The genomic DNA G+C content of the type strain is 44.20 mol%.	CGMCC 1.32887
*Blautia celeris*	sp. nov.	ce'le.ris. L. fem. adj. *celeris*, rapid, pertaining to fast growth of the strain	NSJ-34^T^ from human feces	Cells are rod-shaped with tapered ends, non-motile. Growth in modified MGAM medium occurs at 37 °C, pH 7.0–7.5, in 1–3 days. The genomic DNA G+C content of the type strain is 54.21 mol%.	CGMCC 1.32807 /KCTC 25086
*Blautia lenta*	sp. nov.	len'ta. L. fem. adj. *lenta*, slow, referring to the slow growth of the type strain	M16^T^ from human feces	Cells are curved or straight rod-shaped, non-motile. Growth in modified MGAM medium occurs at 37 °C, pH 7.0–7.5, in 3–10 days. The genomic DNA G+C content of the type strain is 29.50 mol%.	CGMCC 1.32888
*Blautia difficilis*	sp. nov.	dif.fi'ci.lis. L. fem. adj. *difficilis*, difficult, referring to the difficulty of culturing the strain	M29^T^ from human feces	Cells are ovoid to short rod-shaped, non-motile. Growth in modified MGAM medium occurs at 37 °C, pH 7.0–7.5, in 3–10 days. The genomic DNA G+C content of the type strain is 54.51 mol%.	CGMCC 1.32889
*Clostridium segne*	sp. nov.	seg'ne. L. neut. adj. *segne*, slow, inactive, lazy, referring to the slow growth of the strain	BX14^T^ from human feces	Cells are rod-shaped, non-motile. Growth in modified MGAM medium occurs at 37 °C, pH 7.0–7.5, in 3–10 days. The genomic DNA G+C content of the type strain is 48.50 mol%.	CGMCC 1.32890
*Coprococcus hominis*	sp. nov.	ho'mi.nis. L. gen. masc. n. *hominis*, of a human being, referring to the human gut habitat	NSJ-10^T^ from human feces	Cells are ovoid, non-motile. Growth in modified MGAM medium occurs at 37 °C, pH 7.0–7.5, in 3–10 days. The genomic DNA G+C content of the type strain is 51.02 mol%.	CGMCC 1.32463 /KCTC 15956
*Dorea hominis*	sp. nov.	ho'mi.nis. L. gen. masc. n. *hominis*, of a human being, referring to the human gut habitat	NSJ-36^T^ from human feces	Cells are straight rod-shaped, non-motile. Growth in modified MGAM medium occurs at 37 °C, pH 7.0–7.5, in 3–10 days. The genomic DNA G+C content of the type strain is 51.44 mol%.	CGMCC 1.32809 /KCTC 25088
*Enterocloster hominis*	sp. nov.	ho'mi.nis. L. gen. masc. n. *hominis*, of a human being, referring to the human gut habitat	BX10^T^ from human feces	Cells are straight rod-shaped with peaked ends, non-motile. Growth in modified MGAM medium occurs at 37 °C, pH 7.0–7.5, in 3–10 days. The genomic DNA G+C content of the type strain is 52.60 mol%.	CGMCC 1.32891
*Eubacterium segne*	sp. nov.	seg'ne. L. neut. adj. *segne*, slow, inactive, lazy, referring to the slow growth of the strain	BX4^T^ from human feces	Cells are rod-shaped, non-motile. Growth in modified MGAM medium occurs at 37 °C, pH 7.0–7.5, in 3–10 days. The genomic DNA G+C content of the type strain is 35.10 mol%.	CGMCC 1.32892
*Eubacterium difficile*	sp. nov.	dif.fi'ci.le. L. neut. adj. *difficile*, difficult, referring to the difficulty of culturing the strain	M5^T^ from human feces	Cells are curved or straight rod-shaped, non-motile. Growth in modified MGAM medium occurs at 37 °C, pH 7.0–7.5, in 3–10 days. The genomic DNA G+C content of the type strain is 51.20 mol%.	CGMCC 1.32893
*Hungatella hominis*	sp. nov.	ho'mi.nis. L. gen. masc. n. *hominis*, of a human being, referring to the human gut habitat	NSJ-66^T^ from human feces	Cells are fusiform rod-shaped, non-motile. Growth in modified MGAM medium occurs at 37 °C, pH 7.0–7.5, in 3–10 days. The genomic DNA G+C content of the type strain is 52.65 mol%.	CGMCC 1.32842 /KCTC 25127
*Lachnospira hominis*	sp. nov.	ho'mi.nis. L. gen. masc. n. *hominis*, of a human being, referring to the human gut habitat	NSJ-43^T^ from human feces	Cells are rod-shaped, non-motile. Growth in modified MGAM medium occurs at 37 °C, pH 7.0–7.5, in 3–10 days. The genomic DNA G+C content of the type strain is 52.93 mol%.	CGMCC 1.32816
*Ruminococcus hominis*	sp. nov.	ho'mi.nis. L. gen. masc. n. *hominis*, of a human being, referring to the human gut habitat	NSJ-13^T^ from human feces	Cells are spiral or vibrio or rod-shaped, non-motile. Growth in modified MGAM medium occurs at 37 °C, pH 7.0–7.5, in 3–10 days. The genomic DNA G+C content of the type strain is 59.53 mol%.	CGMCC 1.52490
*Mediterraneibacter hominis*	sp. nov.	ho'mi.nis. L. gen. masc. n. *hominis*, of a human being, referring to the human gut habitat	NSJ-55^T^ from human feces	Cells are rod-shaped, non-motile. Growth in modified MGAM medium occurs at 37 °C, pH 7.0–7.5, in 3–10 days. The genomic DNA G+C content of the type strain is 41.50 mol%.	CGMCC 1.32831 /KCTC 25143
*Ruminococcus difficilis*	sp. nov.	dif.fi'ci.lis. L. masc. adj. *difficilis*, difficult, referring to the difficulty of culturing the strain	M6^T^ from human feces	Cells are rod-shaped, non-motile. Growth in modified MGAM medium occurs at 37 °C, pH 7.0–7.5, in 3–10 days. The genomic DNA G+C content of the type strain is 53.90 mol%.	CGMCC 1.32867
*Roseburia lenta*	sp. nov.	len'ta. L. fem. adj. *lenta*, slow, referring to the slow growth of the type strain	NSJ-9^T^ from human feces	Cells are short comma-shaped or long, thin rod-shaped, non-motile. Growth in modified MGAM medium occurs at 37 °C, pH 7.0–7.5, in 3–10 days. The genomic DNA G+C content of the type strain is 44.90 mol%.	CGMCC 1.32469 /KCTC 15957
*Roseburia yibonii*	sp. nov.	yi.bo'ni.i N.L. gen. masc. n. *yibonii*, referring to Chinese actor Yibo Wang, whose series inspired the researcher during the bacterial identification	BX0805^T^ from human feces	Cells are comma-shaped with spiky ends or clavate ends, non-motile. Growth in modified MGAM medium occurs at 37 °C, pH 7.0–7.5, in 3–10 days. The genomic DNA G+C content of the type strain is 46.40 mol%.	CGMCC 1.32827
*Roseburia zhanii*	sp. nov.	zha’ni.i N.L. gen. masc. n. *zhanii*, of Zhan, referring to Zhan Xiao, a Chinese actor whose series inspired the researcher during the bacterial identification	BX1005^T^ from human feces	Cells are rod-shaped or comma-shaped with spiky ends, non-motile. Growth in modified MGAM medium occurs at 37 °C, pH 7.0–7.5, in 3–10 days. The genomic DNA G+C content of the type strain is 40.30 mol%.	CGMCC 1.32828 /KCTC 25140
*Roseburia rectibacter*	sp. nov.	rec.ti.bac'ter. L. masc. adj. *rectus*, straight; N.L. masc. n. *bacter*, rod; N.L. masc. n. *rectibacter*, straight rod-shaped, referring to the cell shape of the strain	NSJ-69^T^ from human feces	Cells are rod-shaped, non-motile. Growth in modified MGAM medium occurs at 37 °C, pH 7.0–7.5, in 3–10 days. The genomic DNA G+C content of the type strain is 41.00 mol%.	CGMCC 1.32845
*Roseburia difficilis*	sp. nov.	dif.fi'ci.lis. L. fem. adj. *difficilis*, difficult, referring to the difficulty of culturing the strain	NSJ-67^T^ from human feces	Cells are spherical, non-motile. Growth in modified MGAM medium occurs at 37 °C, pH 7.0–7.5, in 3–10 days. The genomic DNA G+C content of the type strain is 50.40 mol%.	CGMCC 1.32843 /KCTC 25138
*Agathobaculum hominis*	sp. nov.	ho'mi.nis. L. gen. masc. n. *hominis*, of a human being, referring to the human gut habitat	M2^T^ from human feces	Cells are ovoid to rod-shaped spiky ends, non-motile. Growth in modified MGAM medium occurs at 37 °C, pH 7.0–7.5, in 3–10 days. The genomic DNA G+C content of the type strain is 59.81 mol%.	CGMCC 1.32866
*Agathobaculum faecis*	sp. nov.	fae'cis. L. gen. fem. n. *faecis*, of feces, from which the organism was isolated	NSJ-28^T^ from human feces	Cells are rod-shaped, non-motile. Growth in modified MGAM medium occurs at 37 °C, pH 7.0–7.5, in 3–10 days. The genomic DNA G+C content of the type strain is 65.58 mol%.	CGMCC 1.32801 /KCTC 25080
*Anaerotruncus massiliensis* (ex Togo et al. 2016)	pebnv	mas.si.li.en'sis. L. masc. adj. *massiliensis*, pertaining to Marseille, France, where the organism was for the first time isolated [41]	22A2-44^T^ from human feces	Cells are rod-shaped, non-motile. Growth in modified MGAM medium occurs at 37 °C, pH 7.0–7.5, in 3–10 days. The genomic DNA G+C content of the type strain is 63.36 mol%.	CGMCC 1.52380 /NBRC 113434
*Dysosmobacter segnis*	sp. nov.	seg'nis. L. masc. adj. *segnis*, slow, inactive, lazy, referring to the slow growth of the strain	BX15^T^ from human feces	Cells are rod-shaped, non-motile. Growth in modified MGAM medium occurs at 37 °C, pH 7.0–7.5, in 3–10 days. The genomic DNA G+C content of the type strain is 55.50 mol%.	CGMCC 1.32894
*Dysosmobacter hominis*	sp. nov.	ho'mi.nis. L. gen. masc. n. *hominis*, of a human being, referring to the human gut habitat	NSJ-60^T^ from human feces	Cells are rod-shaped, non-motile. Growth in modified MGAM medium occurs at 37 °C, pH 7.0–7.5, in 3–10 days. The genomic DNA G+C content of the type strain is 61.80 mol%.	CGMCC 1.32836 /KCTC 25148
*Faecalibacterium hominis*	sp. nov.	ho'mi.nis. L. gen. masc. n. *hominis*, of a human being, referring to the human gut habitat	4P-15^T^ from human feces	Cells are rod-shaped, non-motile. Growth in modified MGAM medium occurs at 37 °C, pH 7.0–7.5, in 3–10 days. The genomic DNA G+C content of the type strain is 59.53 mol%.	CGMCC 1.52500 /NBRC 113913
*Flintibacter faecis*	sp. nov.	fae'cis. L. gen. fem. n. *faecis*, of feces, from which the organism was isolated	BX5^T^ from human feces	Cells are rod-shaped, non-motile. Growth in modified MGAM medium occurs at 37 °C, pH 7.0–7.5, in 3–10 days. The genomic DNA G+C content of the type strain is 58.60 mol%.	CGMCC 1.32861
*Flintibacter hominis*	sp. nov.	ho'mi.nis. L. gen. masc. n. *hominis*, of a human being, referring to the human gut habitat	New-19^T^ from human feces	Cells are rod-shaped with spiky ends, non-motile. Growth in modified MGAM medium occurs at 37 °C, pH 7.0–7.5, in 3–10 days. The genomic DNA G+C content of the type strain is 60.49 mol%.	CGMCC 1.31644 /KCTC 15861
*Lawsonibacter hominis*	sp. nov.	ho'mi.nis. L. gen. masc. n. *hominis*, of a human being, referring to the human gut habitat	NSJ-51^T^ from human feces	Cells are rod-shaped, non-motile. Growth in modified MGAM medium occurs at 37 °C, pH 7.0–7.5, in 3–10 days. The genomic DNA G+C content of the type strain is 66.51 mol%.	CGMCC 1.32825 /KCTC 25134
*Lawsonibacter faecis*	sp. nov.	fae'cis. L. gen. fem. n. *faecis*, of feces, from which the organism was isolated	NSJ-52^T^ from human feces	Cells are club-shaped rod-shaped, non-motile. Growth in modified MGAM medium occurs at 37 °C, pH 7.0–7.5, in 3–10 days. The genomic DNA G+C content of the type strain is 67.26 mol%.	CGMCC 1.32826 /KCTC 25135
*Lawsonibacter celer*	sp. nov.	ce'ler. L. masc. adj. *celer*, rapid, pertaining to fast growth of the strain	NSJ-47^T^ from human feces	Cells are straight rod-shaped, non-motile. Growth in modified MGAM medium occurs at 37 °C, pH 7.0–7.5, in 1–3 days. The genomic DNA G+C content of the type strain is 64.83 mol%.	CGMCC 1.32820 /KCTC 25098
*Neobittarella* (ex Bilen et al. 2018)	pebnv	Neo.bit.ta.rel'la Gr. masc. adj.* neos* new; N.L. fem. n. *Bittarella* a bacterial genus name; N.L. fem. n. *Neobittarella* a new *Bittarella* [42]	Type species: *Neobittarella massiliensis*	The same as for type species	
*Neobittarella massiliensis* (ex Bilen et al. 2018)	pebnv	mas.si.li.en'sis L. fem. adj. *massiliensis*, referring to Marseille, where the organism was isolated [42]	NSJ-65^T^ from human feces	Cells are rod-shaped, non-motile. Growth in modified MGAM medium occurs at 37 °C, pH 7.0–7.5, in 3–10 days. The genomic DNA G+C content of the type strain is 63.13 mol%.	CGMCC 1.32841 /KCTC 25131
*Oscillibacter hominis*	sp. nov.	ho'mi.nis. L. gen. masc. n. *hominis*, of a human being, referring to the human gut habitat	NSJ-62^T^ from human feces	Cells are rod-shaped with straight spiky ends, motile. Growth in modified MGAM medium occurs at 37 °C, pH 7.0–7.5, in 3–10 days. The genomic DNA G+C content of the type strain is 58.60 mol%.	CGMCC 1.32838 /KCTC 25149
*Pseudoflavonifractor hominis*	sp. nov.	ho'mi.nis. L. gen. masc. n. *hominis*, of a human being, referring to the human gut habitat	New-38^T^ from human feces	Cells are ovoid or rod-shaped, non-motile. Growth in modified MGAM medium occurs at 37 °C, pH 7.0–7.5, in 3–10 days. The genomic DNA G+C content of the type strain is 49.22 mol%.	CGMCC 1.31611 /KCTC 15862
*Ruminococcus bicirculans*	pebnv	bi.cir.cu’lans L. masc. adj. *bicirculans*, have two circles, referring the cell shapes of the type strain [43]	NSJ-14^T^ from human feces	Cells are spherical, non-motile. Growth in modified MGAM medium occurs at 37 °C, pH 7.0–7.5, in 3–10 days. The genomic DNA G+C content of the type strain is 50.61 mol%.	CGMCC 1.52640 /KCTC 15952
*Ruminococcus intestinalis*	sp. nov.	in.tes.ti.na'lis. N.L. masc. adj. *intestinalis*, pertaining to the intestine habitat	NSJ-71^T^ from human feces	Cells are spherical or ovoid, non-motile. Growth in modified MGAM medium occurs at 37 °C, pH 7.0–7.5, in 3–10 days. The genomic DNA G+C content of the type strain is 39.10 mol%.	CGMCC 1.32847
*Paeniclostridium hominis*	sp. nov.	ho'mi.nis. L. gen. masc. n. *hominis*, of a human being, referring to the human gut habitat	NSJ-45^T^ from human feces	Cells are straight rod-shaped, non-motile. Growth in modified MGAM medium occurs at 37 °C, pH 7.0–7.5, in 3–10 days. The genomic DNA G+C content of the type strain is 55.45 mol%.	CGMCC 1.32818
*Romboutsia faecis*	*sp. nov.*	*fae’cis* L. gen. fem. n. *faecis*, referring to fecal origin	NSJ-18^T^ from human feces	Cells are curved or straight rod-shaped, non-motile. Growth in modified MGAM medium occurs at 37 °C, pH 7.0–7.5, in 3–10 days. The genomic DNA G+C content of the type strain is 51.91 mol%.	CGMCC 1.31399 /KCTC15948
*Intestinimonas massiliensis* (ex Durand et al. 2017)	pebnv	mas.si.li.en'sis. L. fem. adj. *massiliensis*, of Massilia, the Latin name of Marseille, where the bacteria was for the first time isolated [44]	NSJ-30^T^ from human feces	Cells are rod-shaped, non-motile. Growth in modified MGAM medium occurs at 37 °C, pH 7.0–7.5, in 3–10 days. The genomic DNA G+C content of the type strain is 60.58 mol%.	CGMCC 1.32803 /KCTC 25082
*Hydrogeniiclostridium hominis*	sp. nov.	ho'mi.nis. L. gen. masc. n. *hominis*, of a human being, referring to the human gut habitat	NSJ-41^T^ from human feces	Cells are curved or straight rod-shaped, non-motile. Growth in modified MGAM medium occurs at 37 °C, pH 7.0–7.5, in 3–10 days. The genomic DNA G+C content of the type strain is 53.47 mol%.	CGMCC 1.32814 /KCTC 25093
*Catenibacterium faecis*	sp. nov.	fae'cis. L. gen. fem. n. *faecis*, of feces, from which the organism was isolated	NSJ-22^T^ from human feces	Cells are rod-shaped, non-motile. Growth in modified MGAM medium occurs at 37 °C, pH 7.0–7.5, in 3–10 days. The genomic DNA G+C content of the type strain is 55.29 mol%.	CGMCC 1.31663
*Eubacterium hominis*	sp. nov.	ho'mi.nis. L. gen. masc. n. *hominis*, of a human being, referring to the human gut habitat	New-5^T^ from human feces	Cells are rod-shaped, motile. Growth in modified MGAM medium occurs at 37 °C, pH 7.0–7.5, in 3–10 days. The genomic DNA G+C content of the type strain is 35.21 mol%.	CGMCC 1.32837 /KCTC 15860
*Holdemanella hominis*	sp. nov.	ho'mi.nis. L. gen. masc. n. *hominis*, of a human being, referring to the human gut habitat	L34^T^ from human feces	Cells are ovoid or rod-shaped with spiky ends, non-motile. Growth in modified MGAM medium occurs at 37 °C, pH 7.0–7.5, in 3–10 days. The genomic DNA G+C content of the type strain is 33.78 mol%.	CGMCC 1.32895 /KCTC 25157
*Megasphaera hominis*	sp. nov.	ho'mi.nis. L. gen. masc. n. *hominis*, of a human being, referring to the human gut habitat	NSJ-59^T^ from human feces	Cells are ovoid, motile. Growth in modified MGAM medium occurs at 37 °C, pH 7.0–7.5, in 3–10 days. The genomic DNA G+C content of the type strain is 56.12 mol%.	CGMCC 1.32835 /KCTC 25147
*Veillonella hominis*	sp. nov.	ho'mi.nis. L. gen. masc. n. *hominis*, of a human being, referring to the human gut habitat	NSJ-78^T^ from human feces	Cells are spherical (tetracocci), non-motile. Growth in modified MGAM medium occurs at 37 °C, pH 7.0–7.5, in 3–10 days. The genomic DNA G+C content of the type strain is 53.79 mol%.	CGMCC 1.32854 /KCTC 25159
*Tissierella hominis*	sp. nov.	ho'mi.nis. L. gen. masc. n. *hominis*, of a human being, referring to the human gut habitat	NSJ-26^T^ from human feces	Cells are rod-shaped, non-motile. Growth in modified MGAM medium occurs at 37 °C, pH 7.0–7.5, in 3–10 days. The genomic DNA G+C content of the type strain is 51.85 mol%.	CGMCC 1.31394 /KCTC 25080
*Fusobacterium hominis*	sp. nov.	ho'mi.nis. L. gen. masc. n. *hominis*, of a human being, referring to the human gut habitat	NSJ-57^T^ from human feces	Cells are spherical, non-motile. Growth in modified MGAM medium occurs at 37 °C, pH 7.0–7.5, in 2–3 days. The genomic DNA G+C content of the type strain is 29.05 mol%.	CGMCC 1.32833 /KCTC 25145
*Escherichia hominis*	sp. nov.	ho'mi.nis. L. gen. masc. n. *hominis*, of a human being, referring to the human gut habitat	NSJ-73^T^ from human feces	Cells are straight rod-shaped, non-motile. Growth in modified MGAM medium occurs at 37 °C, pH 7.0–7.5, in 1–3 days. The genomic DNA G+C content of the type strain is 62.38 mol%.	CGMCC 1.32849

**Fig. 1:**
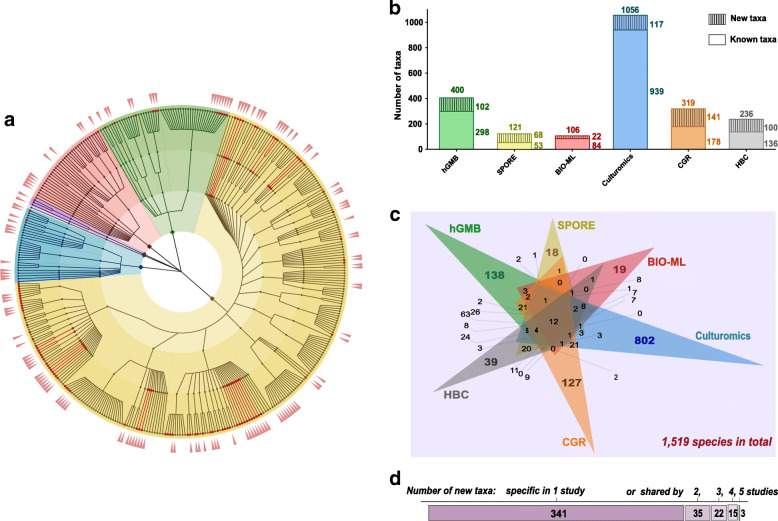
The taxonomic diversity and specificity of hGMB. **a** The taxonomic cladogram displaying the taxonomic diversity of hGMB. The nodes of 102 newly characterized species, 28 novel genus and 3 novel family are indicated in red. The background is color-coded according to 6 phyla, yellow: Firmicutes, green: Bacteroides, red: Proteobacteria, blue: Actinobacteria, gray: Fusobacteria, purple: Verrucomicrobia. The outer ring (the coral red pointers) shows the unique 138 species that are solely covered by hGMB. **b** The taxonomic diversity of gut microbes from different gut microbial collections. hGMB (this study): a human gut microbial culture collection constructed in this study contains 400 species with 102 novel taxa; SPORE [23]: a human gut microbial culture collection constructed in 2016 comprises 121 species including 68 novel-taxon candidates; BIO-ML [21]: a human gut microbial culture collection constructed in 2019 comprises 106 species with 20 novel-taxon candidates; Culturomics [25]: the culturomics study of human gut microbes in 2016 reveal the discovery of 1056 species including 247 novel taxa, of which 117 were still novel-taxon candidates by the time of manuscript preparation; CGR [20]: a human gut microbial culture collection constructed in 2019 comprises 319 species determined based on the 16S rRNA gene sequence clustering at identity of 98.7%, of which 141 taxa are novel-taxon candidates; HBC [24]: a human gut microbial culture collection constructed in 2019 contains 236 species with 100 novel-taxon candidates. **c** The Venn diagram displaying the unique and shared taxa in each study. The numbers of taxa uniquely in one collection or shared by different studies are labeled in the panel. **d** Summary of novel taxa claimed by 1 or more than 1 study. Numbers in the bar represent the number of novel taxa. Note: The taxonomic diversity of each previously published study compared in panel b and c was re-mined and summarized as described in “Methods,” and by the time of this writing, all the mentioned “novel-taxon candidates” are never described

With the above efforts, we constructed the human Gut Microbial Biobank (hGMB), which comprises 1170 strains (Table S7) that represent 400 bacterial species from 159 genera, 53 families, and 6 phyla (Fig. 1a). All 1170 strains in hGMB have been deposited in China General Microbiological Culture Collection Center (CGMCC) for public access (hGMB homepage at the CGMCC website: http://www.cgmcc.net/english/hgmb), and the type strains of novel taxa were also deposited in Korean Collection for Type Cultures (KCTC) or NITE Biological Resource Center (NBRC) (Table 1). The strain accessions, phenotypical features, and genomic data of hGMB members are also available at the hGMB homepage (hgmb.nmdc.cn) [45] and eLMSG [46].

#### hGMB expands the existing human gut microbial collections and provides the “most wanted” gut microbes

To better understand the cultured bacterial diversity of the human gut microbiota and to demonstrate the expansion of the existing publicly available human gut bacterial repository by hGMB, we compared hGMB with recent major works on large-scale collections of human gut microbes, as of SPORE [23], CGR [20], BIO-ML [21], Culturomics [25], and HBC [24]. By revisiting the data and extracting the taxonomic information from the mentioned studies as described in “Methods,” we individually profiled the taxonomic diversity of each study (Fig. 1b). Notably, except for hGMB, all the new taxon candidates from the other 5 studies (SPORE [23], CGR [20], BIO-ML [21], Culturomics [25], and HBC [24]) had never been described. Further analysis revealed that the taxon pools of different collections overlapped with one another, and the distribution of shared and unique taxa among 6 studies is shown in Fig. 1c and d. The 6 studies collected in total 1519 nonredundant cultured bacterial species from the human gut. hGMB provides 138 unique gut microbial species to the large-scale-cultivation-based gut microbial repository (Fig. 1c). Notably, 76 of the 138 unique hGMB species were novel taxa. As shown in Fig. 1d, the 6 collections contributed 416 nonredundant novel taxon candidates, 102 of which were well described and denominated in this study under the rules of ICNP by hGMB, accounting for 24.5% of the total novel taxa.

By BLAST analysis, we further identified that 24 hGMB species were on the list of “most-wanted” or “medium priority” taxa proposed by the Human Microbiome Project [47] (Table S7). One “most-wanted” taxon-the *Eubacterium difficile* sp. nov. (Taxon_69) and 9 “medium priority” taxa including three novel genera (*Simiaoa* gen. nov.*, Jutongia* gen. nov. and *Wansuia* gen. nov.) were novel taxa first described in this work (Table 1).

### hGMB largely represents the taxonomic diversity of the human gut microbiota

**Fig. 2:**
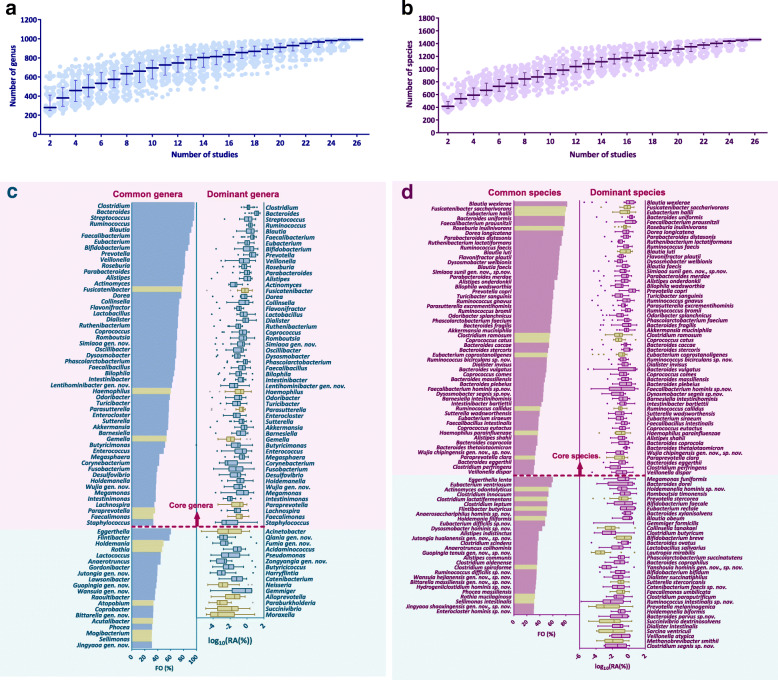
The cultured recovery of major composition of human gut microbiota by hGMB at genus and species levels. **a** and **b** The rarefaction curves displaying the increase trend of the numbers of assigned genera (**a**) and species (**b**) as 1 to 26 16S rRNA gene amplicon datasets (Table S8) were sampled for combined analysis. **c** The coverage of human gut common (bar chart) and dominant (box-and-whiskers plot) genera by hGMB. All the genera covered by hGMB were colored in blue while the genera absent in hGMB were color in olive brown. **d** The coverage of human gut common (bar chart), dominant (box and whiskers plot) species by hGMB. All the genera covered by hGMB were colored in purple while the genera absent in hGMB were color in olive brown. Common genera/species: genera/species with equally weighted average frequency of occurrence (FO) > 30% (definition: FO = 100% is defined when a taxon presents in all samples, while FO = 0 is defined when a taxon presents in none of the samples; The equally weighted average FO is the mean value of the average FOs of the 26 analyzed studies); Dominant genera/species: genera/species with equally weighted average relative abundance (RA) > 0.1% (log 10 (RA(%)) > − 1) (definition: The equally weighted average RA is the mean value of the average RAs of the 26 analyzed studies). The light-pink background in panel c and d highlighted the core genera/species shared by both dominant and common genera/species, while the light-blue background marked out the taxa presenting uniquely in either dominant or common genera/species. The bar chart in panel c and d shows the mean values of the 26 FO averages (%), while the box-and-whiskers plot shows the Log 10 of average Ras (%) of each taxon in each study, center line: median, bounds of box: quartile, whiskers: Tukey extreme

To further evaluate the taxonomic representativeness of hGMB to the main taxonomic composition of human gut microbiota, we collected publicly available 16S rRNA gene amplicon datasets of 26 studies (*N* = 26) from the NCBI SRA database (date: 2020-02-22). These 26 datasets had specimen numbers ranging from 102 to 3538, representing human gut microbiota from donors of diverse genetic and environmental backgrounds (see Table S8 for accessions of the studies). The 26 datasets were separately processed, quality-controlled, and weighted by a standard USEARCH-based analysis pipeline as described in the “Methods” section. Results showed that the 26 datasets contained a total of 11, 647 quality-controlled samples (*n* = 11,647) and each had 228 ± 85 OTUs. The taxonomy status of each OTU was annotated using LTP_vhGMB customized by supplementation of LTP database v132 with the 102 novel taxa. The equally weighted average relative abundance (RA) and frequency of occurrence (FO) for each annotated species or genus were calculated as described in “Methods.” The results showed that 76.3 ± 8.0% and 53.7 ± 11.8% of the total reads were assigned to 990 genera and 1461 species, respectively. As shown in Fig. 2a and b, the accumulative curves were almost saturated after sampling 24 datasets from the 26 studies, at either the genus or species level. The taxonomic composition of the 26 studies could largely represent the taxonomically defined human gut microbiota composition at the genus and species levels. We identified 386 genera that appeared in over 1% (equally weighted average FO > 1%) of the 26 study samples (*n* = 11,647), and hGMB covered 129 genera. If we defined the genera with equally weighted average RAs > 0.1% as “dominant genera,” and those genera with equally weighted average FOs > 30% as “common genera,” 69 and 74 genera were recognized as dominant and common genera, respectively (Fig. 2c). The 69 dominant genera represented 94.7 ± 4.7%, while the 74 common genera represented 91.3 ± 11.3%, of the total annotated 16S amplicon reads. hGMB covered 85.1% and 84.1% of the common and dominant genera, respectively. If the same criteria were used to define “dominant species” (equally weighted average RAs > 0.1%) and “common species” (equally weighted average FOs > 30%), 91 dominant and 84 common species were recognized from the 26 studies (Fig. 2d). hGMB covered 79.1% of the dominant species and 80.9% of the common species. There were 12 and 16 newly described species of hGMB belonging to the dominant and common species, respectively.

### Novel taxa are prevalent in the global human gut microbiome and illuminate “dark taxa”

**Fig. 3:**
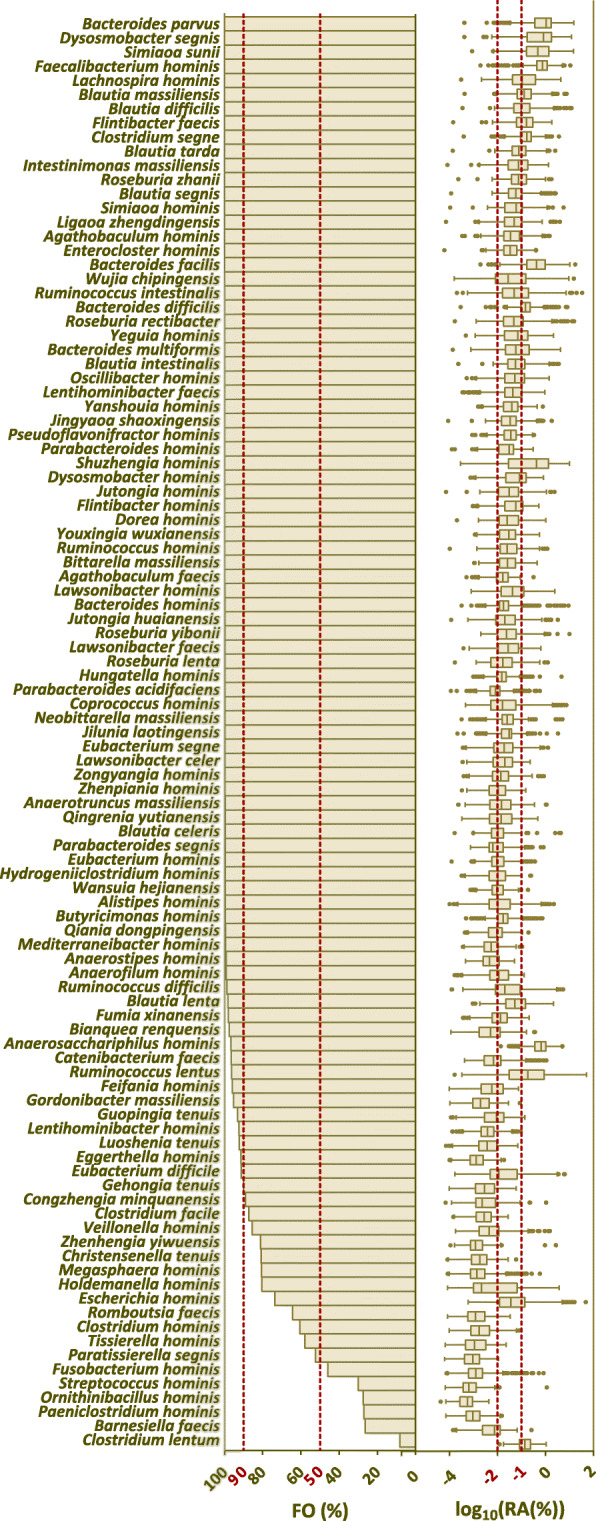
The prevalence of novel taxa in hGMB among global health human gut metagenomes (*n* = 1129). The bar charts demonstrated the frequency of occurrence (FO) of each novel taxa among 1129 analyzed health human gut metagenomes (Table S9) (definition: FO = 100% is defined when a taxon presents in all samples, while FO = 0 is defined when a taxon presents in none of the samples); The box-and-whiskers plot displayed the relative abundance (RA) of each novel taxa among all samples in Log 10 format. center line: median, bounds of box: quartile, whiskers: Tukey extreme

A total of 102 of the 400 hGMB species were reported for the first time, and they represent new taxa. To display the distribution and abundance of the new taxa in human gut microbiomes, we retrieved open-access metagenomes (*n* = 1129) representing healthy human GMs globally for combined analysis. The metagenomic datasets (Table S9) were selected by searching in GMrepo [48] with defined filter conditions as described in “Methods.” The distribution of 102 new taxa among the 1129 metagenomes was investigated by kraken2-based annotation of each sample with customized taxonomically defined GTDB database supplemented with 102 hGMB new species genomes, and the relative abundance of novel taxa in each sample was estimated by Bracken (see “Methods” for details). On average 72.4 ± 14.3% of the total reads of the 1129 metagenomes were taxonomically classified, and the novel hGMB taxa covered 15.4 ± 7.4% of the classified reads. The results shown in Table S10 and Fig. 3 revealed that 101 out of the 102 novel taxa were annotated in at least one metagenome, and 31 of the 101 novel taxa had average RAs>0.1% (box-and-whisker plot in Fig. 3). Notably, the new hGMB taxa were widely distributed among global human gut metagenomes, as 95, 82, and 17 of the novel taxa were found in > 50%, > 90%, and 100% of the investigated metagenomic samples (*n* = 1129), respectively, accounting for 93.1%, 80.4%, and 16.7% of all the novel taxa described in this study, respectively (bar chart in Fig. 3).

Most recently, researchers identified 4644 inferred prokaryotic species by the construction of the largest-to-date Unified Human Gastrointestinal Genome (UHGG) database, and 70% of the UHGG species were assigned based on metagenome-assembled genomes (MAGs) but lacked cultured representatives [8]. To assess the possible contribution of new genomes in hGMB to the improvement of cultured representatives of UHGG species as well as to the illumination of potential “dark taxa” that had not been identified by culture-independent metagenomic studies, the Mash distance between the 102 novel taxon genomes and 4644 UHGG representatives were calculated, and the genome pairs maintaining a distance < 0.05 were identified from the same species. As shown in Table S10, 78 out of the 102 new genomes matched the UHGG species, and 22 of them were uncultured species having only MAG representatives in the UHGG. Thus, the hGMB species made the 22 UHGG genomes cultured. Additionally, 6 UHGG species matched by hGMB genomes had only cultured genomes from unknown environments, demonstrating that their representatives occurred in the human gut. Notably, 24 new hGMB genomes did not match any UHGG species-level genomes, indicating that they were “dark species” in human GMs that had not been identified by previous cultivation-based or metagenomic studies.

### New hGMB genomes enrich global human gut gene catalogs and recover cultured “dark” gene repositories

**Fig. 4:**
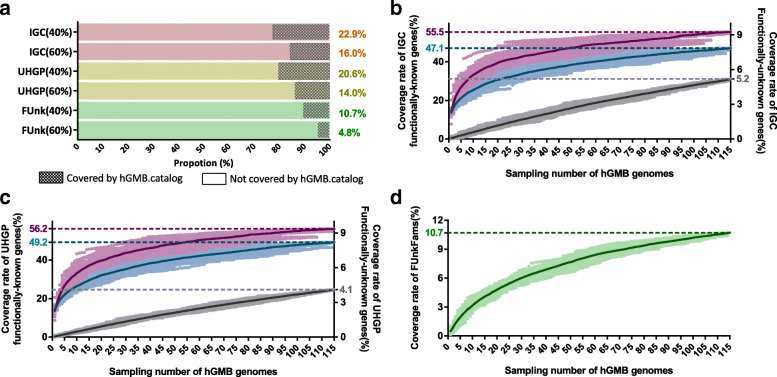
The functional coverage of global human gut gene catalogs by new hGMB genomes. **a** The coverage of IGC [49], UHGP [8] and FUnkFams [50] by hGMB.catalog. The hGMB.catalog was constructed by extraction of 341,876 nonredundant genes from 115 newly sequenced genomes in hGMB and was BLAST analyzed against subject gene catalog IGC (pink bars), UHGP (yellow bars) and FUnkFams (green bars) with cutoff sequence identities of 40% and 60%, respectively. The *y*-axis names indicated the names of subject gene catalog and the sequence identities used for BLAST (in bracket). The coverage rates were listed in panel on the right side of each bar. **b** and **c** The rarefaction curves displaying the accumulatively increased coverage of the KOs (purple), GOs (blue) and unannotated genes (gray) in IGC (**b**) and UHGP (**c**) catalogs. The sampling was repeated for 50 times at each *x*-axis point; Light purple dot: the coverage rates of KO functions of IGC or UHGP gene catalogs when specified numbers of genomes were randomly sampled from 115 hGMB genomes; dark purple line: the mean coverage rate of KO functions; light blue dot: the coverage rates of GO functions of IGC or UHGP gene catalogs; dark blue line: the mean coverage rate of GO functions; gray dot: the coverage rates of unannotated genes of IGC or UHGP; black line: the mean coverage rate of unannotated genes of IGC or UHGP. **d** The rarefaction curves displaying the accumulatively increased coverage of conserved functionally unknown proteins in FUnkFams. The sampling was repeated for 50 times at each *x*-axis point; light green dot: the coverage rates of FUnkFams proteins when sampled randomly; dark green line: the mean value of the coverage rates

Gene cataloging outlines human GM functionality potentials, and several gene catalogs have been established [8, 49]. We created nonredundant gene catalogs containing 341,876 nonredundant genes with 115 newly sequenced hGMB genomes (named hGMB.catalog) and compared them with the largest-to-date human GM catalogs, the Unified Human Gastrointestinal Protein (UHGP) catalog and the Integrated Gene Catalog (IGC) by BLAST analysis. Although the majority (79–90%) of the nonredundant genes in hGMB catalogs were represented by IGC and UHGP (Table S11), hGMB further enriched human GM gene catalogs. With a threshold value of 60% amino acid sequence identity (for functional conservation), hGMB contributed 45,388 and 79,982 new nonredundant sequences to the UHGP and IGC, respectively. When the identity value was decreased to 40% (for structural conservation), the numbers of new genes added to the UHGP and IGC were 32,669 and 44,924, respectively. As shown in Fig. 4a, the hGMB.catalog covered 14.9% and 21.5% of IGC genes under threshold identities of 60% and 40%, respectively. For UHGP, the coverages by hGMB.catalog were 13.7% and 20.3% at the functional and structural levels, respectively.

We then investigated the representativeness of hGMB genomes to the characterized functions of human GMs. For this purpose, the UHGP, IGC, and all 115 hGMB genomes were annotated with eggNOG [15]. A cumulative analysis of the KO and GO profiles was conducted to determine the coverages of IGC and UHGP by random incremental selection of the hGMB genomes, and the results are shown in rarefaction curves (Fig. 4b and c). The hGMB genomes covered 55.5% and 56.2% of the KO genes from the IGC and UHGP catalogs, respectively (purple lines in Fig. 4b and c). Similarly, hGMB genomes represented 47.1% and 49.2% of the known GO functions of IGC and UHGP catalogs, respectively (blue lines in Fig. 4b and c).

In addition to the representativeness of functionally known genes of human GMs, hGMB also provided a cultured repository of functionally unknown genes within global gene catalogs, and the recovery of these “dark genes” by cultured hGMB members would facilitate the culture-based experimental studies to bring more “dark functions” in the human gut to light. The eggNOG annotation results of IGC and UHGP catalogs revealed that 30.9% and 30.6% of genes/proteins were functionally unknown. BLAST analysis (amino acid sequence identity > 40% and query coverage > 70%) revealed that the hGMB genomes covered 4.0% (gray line in Fig. 4b) and 3.5% (gray line in Fig. 4c) of the unannotated genes in IGC and UHGP, respectively. The functionally unknown genes matched for IGC and UHGP are listed in Tables S12 and S13, respectively. We also plotted the coverage of the Function Unknown Families of homologous proteins (FUnkFams), a “most wanted” list of conserved microbial protein families with no known domains and prioritized for functional characterization [50] by hGMB.catalog and hGMB genomes. The results revealed that, with a threshold value of 40% sequence identity and 70% query coverage, hGMB covered 5987 out of 61,970 (9.7%) of the functionally unknown proteins in FUnkFams (Fig. 4a and d). The profiles of the FUnkFams sequences matched to the hGMB genomes are summarized in Table S14, thereby facilitating further culture-based study of these functionally unknown genes.

## Discussion

By implicating previous experiences in cultivation and understanding of gut microbial physiology and ecology [23, 25, 26, 36], in this study, we adopted 11 pretreatments and 67 culture conditions (including different media) and obtained 10,558 pure bacterial isolates. Intensive efforts were made to modify culture media, particularly in diversifying the ingredients in the media (Table S4). For example, based on our previous study [36], we found that mouse gut microbes preferred 8 carbon sources (d-mannose, d-fructose, fructo-oligosaccharide, d-galactose, palatinose, l-rhamnose, d-(+)-cellobiose, and d-trehalose) for growth. In this study, the 8-carbohydrate mixture was supplemented with media to improve human gut microbial cultivability (Table S4). The results indicated that this mixture improved the growth of a notable number of gut bacterial isolates, especially members of *Clostridiales* and *Erysipelotrichales*. According to our statistics, *Eubacterium hominis* sp. nov., *Eubacterium segne* sp. nov., *Agathobaculum hominis* sp. nov., *Fusobacterium hominis* sp. nov., *Wujia chipingensis* gen. nov. sp. nov. and *Luoshenia tenuis* gen. nov. sp. nov. were all exclusively isolated from agar plates of modified mGAM supplemented with an 8-carbohydrate mixture. To increase the diversity of cultured taxa and to reduce workload, we pooled fecal samples collected at the same time and geography and used them for microbial isolation (please refer to supplemental Table S2 for more details). As a result, each strain in hGMB can only be traced back to its donor’s geography, rather than exact personal information, rendering hGMB a less appropriate repository for future studies relying strictly on a one-to-one link between isolates and donors. As shown in Table S7, the 102 new species identified in hGMB belonged to 24 different families (including 3 novel families), and *Lachnospiraceae* was the most abundant family including 29 new species and 7 new genera (*Wujia* gen. nov., *Simiaoa* gen. nov., *Jutongia* gen. nov., *Qiania* gen. nov., *Zhenhengia* gen. nov., *Jingyaoa* gen. nov., and *Wansuia* gen. nov.). Similarly, *Lachnospiraceae* is one of the most dominant families in the GM of healthy adults, accounting for 10–45% of the total bacteria in feces [51], and is considered to play diverse but controversial roles in the maintenance of host gut homeostasis [27, 52]. On the one hand, *Lachnospiraceae* members, such as the *Roseburia* species, were beneficial to hosts via the production of short-chain fatty acids (SCFAs) and secondary bile acids [53–55], protection of hosts from pathogen infections [54, 56, 57], and stress-induced visceral hypersensitivity [53]. On the other hand, studies have demonstrated positive correlations between *Lachnospiraceae* and diseases such as nonalcoholic fatty liver disease (NAFLD) [58] and chronic kidney disease (CKD) [59]. Animal experiments demonstrated that gavage with *Lachnospiraceae* accelerated the development of diabetes in obese mice [60] and aggravated the inflammation of intestinal epithelial cells in TLR5^−/−^ mice [61]. The contradictory conclusions signified that the function(s) of *Lachnospiraceae*, a predominant gut microbial family in humans, are complicated. Accordingly, the culture-based study of *Lachnospiraceae*-host interactions would enable a better understanding of their complex roles in health and disease, on the condition that diverse cultured *Lachnospiraceae* members are available. hGMB contains 93 strains from 49 different *Lachnospiraceae* species and provides an accessible *Lachnospiraceae* repository for future study.

hGMB also provides members of *Christensenellaceae*, including *Christensenella minuta*, *Christensenella tenuis*, and 3 new genera (*Guopingia* gen. nov., *Luoshenia* gen. nov. and *Gehongia* gen. nov.). *Christensenellaceae* is a recently identified gut commensal bacterial family containing limited cultured representatives [62] and has been considered a promising probiotic candidate for the intervention of obesity and other metabolic syndromes [63, 64]. In particular, *Christensenella minuta* was experimentally verified to reduce weight gain in recipient mice [65]. To explore and evaluate *Christensenellaceae*’s therapeutic potential, more studies are necessary. hGMB provides resources serving further studies. Notably, *Guopingia* and its type species *Guopingia tenuis* widely occurred in global human GMs as they were found in all investigated datasets, making it an interesting candidate for study. In addition to the contribution of previously uncultured gut microbes to the public (Table S7, Figs. 2 and 3), hGMB also includes considerable numbers of strains representing known species that were research hotspots in human GM studies. Some of these “star species” are commonly recognized to have probiotic potential, such as *Akkermansia muciniphila* [66], *Faecalibacterium prausnitzii* [67], *Roseburia intestinalis* [68], and *Lactobacillus* and *Bifidobacterium* members [69, 70], while others, such as *Enterococcus faecium* [71], *Ruminococcus gnavus* [72], *Clostridioides difficile* [73], and *Klebsiella* species [74], have been revealed to play pathogenic roles in hosts. There is a large group of gut microbial species that were reported to have strain-specific effects on hosts [75, 76]. One example is *Bacteroides fragilis*, as both pathogenic and probiotic strains were identified from this species [76, 77]. Most recently, the *Bacteroides xylanisolvens* strain from hGMB has been demonstrated to function as a probiotic in the alleviation of nonalcoholic hepatic steatosis via the Bacteroides-Folate-Liver Axis [78]. In summary, hGMB contribute to cultured GM diversity and thus would facilitate in-depth and extensive studies of the functional features of these microbes.

## Conclusion

In this study, 10,558 bacterial isolates from 239 fecal samples of healthy Chinese volunteers were obtained. These bacterial isolates represent 400 species of 159 genera, belonging to 53 families and 6 phyla. A publicly accessible human Gut Microbial Biobank (hGMB) that contains 1170 representative bacterial strains of 400 human gut microbial species was established. hGMB expands gut microbial resources and genomic repositories by adding 102 new species and 115 new genomes of human gut microbes. Based on the newly discovered species in this study, 28 new genera and 3 new families of human gut microbes were identified and proposed. All novel taxa were described and denominated following the rules of ICNP for later valid approval of nomenclatures. Further analysis revealed that hGMB represented over 80% of the prevalent microbial genera and species in the human gut, and covered 50% of KEGG Orthology functions and 10% of the functionally unknown genes in FUnkFams. By integrative analysis of hGMB genomes with the UHGG database and 1129 global health human gut metagenomes, we profiled the taxonomic prevalence, distribution, and genetic features of the 102 new hGMB species among human GMs, demonstrating that hGMB has great potential in bringing more human gut microbial “dark matters” to light.

## Methods

### Sample collection and treatment

The whole project was approved by the Research Ethics Committee of the Institute of Microbiology, Chinese Academy of Science, and the assigned number authority of the ethical approval is APIMCAS2017049. We inquired each donor candidate about the health conditions, history of clinical visits for the last half-year, and history of antibiotic treatments for the last two months in person before a consent form was signed for the donation of feces, and the ones without any clearly diagnosed chronic and malignant disease were considered as healthy donors. The feces samples (*n* = 239) were collected from healthy volunteers who did not receive any medical treatment for the last 2 months before sampling. The sample donors were mainly from six different areas of China (Beijing, Henan, Heibei, Xinjiang, Guangdong, Inner Mongolia). The samples collected in Beijing were kept fresh and transferred into an anaerobic workstation (AW500, Electrotek, UK) for sample pretreatment within 2 h, while the feces from the other areas were frozen on dry ice immediately after sampling and delivered to the Lab for pretreatment. To enable a better recovery of diversity, as listed in Table S2, about 10 or less samples collected at the same time and geographic location were pooled together for pretreatment and subsequent isolation steps. The 11 pretreatment conditions are given in Table S3 and the alcohol pretreatment strategies were derived from Browne et al. [23]. The gas flow composition in the anaerobic workstation was 85% N_2_, 5% CO_2_, and 10% H_2_.

### Bacterial isolation and cultivation

The pretreated samples were filtered using a cell strainer (BD Falcon, USA) to remove the large insoluble particles in suspension and serially diluted into 10^−1^ to 10^−8^-folds. Then, 100 μl of each dilution was spread onto different agar plates for either aerobic or anaerobic incubations at 37 °C. We applied 67 different culture conditions for bacterial cultivation and isolation as shown in Table S5. The detailed recipes of 21 base media and supplements used in this study are provided in Supplementary Methods. The supplementation of clarified rumen fluid and sheep blood in culture media was conducted by following Lagier et al. [25]. The colony isolation and identification were performed as described in our previous study [36]: All the single colonies appearing on the agar plates after incubation for 2 to 60 days were picked. The picked colonies were then inoculated into 48-well plates containing 700 μl of broth media in each well. The 96-well plates containing isolates were incubated at 37 °C for 2–30 days depending on the growth rate of isolates. Then, 50 μl of the media in each well were collected and centrifuged at 13,000 rpm for 1 min. The bacterial pellet was lysed with 2 μl of NaOH/SDS lysis buffer (Amresco, USA) and diluted with 100 μl deionized water. Two microliters of dilution were used as a template for PCR-based amplification of 16S rRNA gene sequences with DreamTaq Green PCR Master Mix (Thermo Fisher Scientific, USA) (primers: 27 F: 5′-AGAGTTT GATCCTGGCTCAG-3′; 1492 R: 5′-GGTTACCTTGTTACGACTT-3′). The PCR products were sequenced using Sanger sequencing (TIANYI HUIYUAN Ltd., China). The wells containing a single 16S rRNA gene were further enlarged and cultured by inoculation in tubes containing 5 ml of liquid medium and streaking on agar plates for further purification, preservation, and characterization either anaerobically or aerobically. During the taxonomic characterization and preparation of strain transferred to IDAs, strains were serially inoculated into new media and cultured and transferred for several generations. In each inoculation and cultivation step, the 16S rRNA gene sequences of the new culture were sequenced and checked. The taxonomy of all the cultured isolates was recognized by BLAST analysis of the 16S rRNA gene sequences against both the EZBioCloud and the NCBI 16S ribosomal RNA sequence database (Update date: 2020/08/08, number of sequences: 21,632). The isolates with 16S rRNA gene sequence identities > 98.7% to any species (valid names only) in EZBioCloud were considered as known species [37]. The isolates with 16S rRNA gene sequence identities ≤ 98.7% to any known species in both databases were considered as candidates of novel taxa [37]. All the isolates potentially representing novel taxa were further grouped into different species-level clusters based on the 16S rRNA gene sequence identity (cutoff value 98.7% for different species) and for each species-level novel taxon, 1 strain was designed as type strain for later genomic sequencing and polyphasic characterization.

### The preservation strategy of bacterial strains

We performed the isolation using mixed fecal samples for 16 batches. For each batch, we deposit at least 1 representative strain of every identified species for long-term cryopreservation in CGMCC for public use, no matter whether strains of these species had ever been preserved or not in previous batch of work. We use such a redundant-preservation strategy to (1) ensure that at least 1 strain for each species could be properly recovered after long-term storage and (2) enable a better strain-level diversity in hGMB considering that different strains of the same species from different donors might differ in genomic or physiological features. The cryopreservation of selected strains was performed as described in previous work [36]: Pure cultures were inoculated onto agar plates and incubated until enough single colonies appearing on the plates. All the colonies on agar plates were collected using a cell scraper, suspended in a protective solution (15% glycerol and 85% bovine serum solution), and stored at − 80 °C or in liquid nitrogen. The CGMCC accessions of 1170 preserved strains were available in Table S6 and hGMB special page on CGMCC (http://www.cgmcc.net/english/hgmb). To meet the rules of the International Code of Nomenclature of Prokaryotes (ICNP), the 102 type strains of new species in hGMB were also preserved in a second IDA as KCTC or NBRC, and the majority of accessions could be found in Table 1 and hGMB homepage.

### Polyphasic characterization and nomenclature of novel taxa

The delineations of novel taxa were based on the analysis of each type of strain in terms of phylogenetic, genomic, physiological, and morphological characteristics as described in previous work [36, 79] and documented in Supplementary Data 1. For each new species, the phylogenetic tree was constructed with the 16S rRNA gene sequences of the type strains from the phylogenetically close neighboring genus and species using MEGA7 [80] under the neighbor-joining method to depict the phylogenetic distribution and taxonomic relation of each novel taxa and its closely related taxa (Figure SD-1a to Figure SD-108a in Supplementary Data 1). Additionally, the genome-based phylogenomic tree for each new species was also constructed using gtdb-tk with classify_wf command under default parameters [81] (Figure SD-1c to Figure SD-108c in Supplementary Data 1). The closely related taxa on phylogenetic and phylogenomic trees were used for further genome-based analysis. The genome-based analysis of novel taxa included the calculation of the average nucleotide identity (ANI), digital DNA:DNA hybridization (dDDH) and the percentage of conserved proteins (POCP). The ANI values and the heatmaps (Figure SD-1d to Figure SD-108d in Supplementary Data 1) were generated using OrthoANI OTA software [82]. The dDDH value between draft genome of new species and its phylogenetically and phylogenomically closest genomes were calculated using the Genome-to-Genome Distance Calculator 2.1 (GGDC) [83]. The POCP between each genome and its phylogenetically closest genome was calculated using BLASTp v2.9.0+ and was used for taxonomy delineation at the genus level [79, 84]. The physiological and biochemical features of type strains of novel taxa were profiled using ANI MicroPlates (BIOLOG, the USA) following the manufacturer’s instruction. The bacterial cell morphology was observed using a transmission electron microscope (TEM) JEM-1400 (JOEL, Japan) (Figure SD-1b to Figure SD-108b in Supplementary Data 1). The motility of bacteria was examined with the light microscopy Axiostar plus 156 (ZEISS, Germany). The nomenclature of each characterized novel taxa was proposed according to the rules of ICNP. After comprehensive consideration of several main works [36, 37, 79, 84–86], the following criteria were used for proposing novel taxa: 1, Taxon meeting the following three criteria simultaneously was defined as new species: (1) the 16S rRNA sequence identity < 98.7%, (2) dDDH value < 70%, (3) ANI < 95%, or ANI between 95~96% but the morphology and physiology feature of the novel taxon was distinct from that of its closely related species. 2, If the new species simultaneously had (1) a 16S rRNA gene sequence identity < 95% to any known species, (2) a POCP value < 50% to its closely related taxon, (3) any significant difference in morphology and physiology with neighbor genera, and (4) location at an independent clade on the phylogenetic tree, it would be further defined as new genus. 3, If the type species in the new genus (1) had a 16S rRNA gene sequence identity < 90% to any known species, (2) was clustered on a separate clade distant from any known genera on the phylogenetic tree and its closest neighbor genera were from at least two different families, and (3) maintained significant difference in morphology and physiology to the neighbor families, the taxon would be further defined as new family.

### Genome sequencing and analysis

The genomes of all 102 novel taxa, 6 new strains of known species with 16S rRAN gene identity < 98.7% to the corresponding type strains and 7 new strains with 16S rRAN gene identity > 98.7% to the type strains of known species but with no genome available in NCBI were sequenced. The genomic DNA was extracted using either the DNeasy Blood & Tissue Kit (Qiagen, Germany) or the Wizard Genomic DNA Purification kit (Promega, USA). The DNA concentrations were measured using Qubit 4.0 (Thermo Fisher Scientific, USA). The degradation of purified DNA was checked by electrophoresis, and the DNA was considered as undegraded if no apparent smear was observed on the agarose gel. The bacterial species having more than 5 mg undegraded DNA were sequenced using the PacBio SMRT technique for the achievement of complete genomes. The qualified genomic DNA was fragmented with G-tubes and end-repaired to prepare SMRTbell DNA template libraries (with fragment size of > 10 Kb selected by the bluepippin system) according to the manufacturer’s specification (PacBio, USA). Library quality was detected by a Qubit 3.0 Fluorometer (Life Technologies, USA) and average fragment size was estimated on an Agilent 4200 (Agilent, CA). SMRT sequencing was performed on the Pacific Biosciences RSII sequencer (PacBio, USA), according to standard protocols. The raw reads were filtered by the SMRT 2.3.0 to discard low-quality reads and the filtered reads were assembled to generate one contig without gaps. The hierarchical genome-assembly process (HGAP) pipeline was used to correct for random errors in the long seed reads (seed length threshold 6 Kb) by aligning shorter reads from the same library against them. The corrected, preassembled reads were used for de novo assembly. For the genomic DNAs not qualified for SMRT sequencing were sequenced using Hiseq X-ten platform (Illumina, USA) to generate draft genomes. The sequencing libraries were generated with NEB Next® Ultra™ DNA Library Prep Kit for Illumina® (New England Biolabs, USA) following the manufacturer’s recommended procedures and the index codes were added. The library quality was evaluated by the Qubit 3.0 Fluorometer (Life Technologies, USA) and the average fragment size was estimated using Agilent 4200 (Agilent, CA). The DNA library was sequenced on an Illumina Novaseq platform and 1-2 GB 150 bp paired-end reads were generated. The raw data were quality controlled using company’s own compiling pipeline. The filtered paired reads were assembled using the SPAdes software v3.9.0 [87] into a number of contigs (k-mer sizes of 59, 79, 99, and 119), and the contigs longer than 500 nt were retained as final splicing. The assembled contigs were then BLASTed against NCBI nt database using blastn with e-value of 1e-5 to remove potential contamination contigs not hitting to the target taxonomic classification. Above library preparation, sequencing and assembly steps were performed by a commercial company (Guangdong Magigene Biotechnology Co.,Ltd., China). The quality and assembly information of the genomes from the commercial company were further assessed in the lab. The numbers and N50 of contigs in each genome, the contamination, and the completeness were estimated using CheckM v1.0.12 (lineage_wf function) [88] and are listed in Table S6. The Estimated quality score of each assembly was calculated by “completeness − 5 × contamination” [8]. Genomes with contamination > 5% were further decontaminated using MAGpurify v2.1.2 [29]. If any quality-controlled genome had <50% completeness, or >5% contamination, or an estimated quality score < 50 would be re-sequenced. For the estimation of average coverage depth of assembly to the sequencing short reads, bwa v0.7.17 (mem function) [89]were used for reads mapping, and samtools v 1.9 (view -F 4 -bS and depth commands) [90] were used for depth estimation. A one-line script (less samtools.depth.output.file|awk '{sum+=$3; sumsq+=$3*$3} END { print "Average = ",sum/NR}’) were used for extraction of average depth from the output of samtools and the results.

For the genome component prediction, the coding genes were predicted with glimmer3 [91] and Prodigal v2.6.3 [92], and the rRNA genes were retrieved by RNAmmer v1.2 [93]. The function annotations of all genomes were performed with eggNOG database v4.5 by local emapper v1.0.3 (-m diamond) [15]. The comparison of novel-taxon genomes with 4644 species-level genomes in UHGG were performed using Mash v2.2.2 (dist function), and the genome pairs maintaining a mash distance < 0.05 (corresponding in most cases to ANI > 95%) were identified to represent the same species [94]. The MAGnify accession and culturing status of UHGG genomes hit by hGMB new genomes were extracted from the Table S3 of the UHGG publication [8]. Default parameters were used for each software unless otherwise specified.

### Human gut metagenome collection and analysis

The publically available metagenomic data representing the global health human GMs were selected by search with defined filter conditions (experiment_type = 'Metagenomics' AND QCStatus = ‘Good runs’ AND host age > 5 AND country=is not null AND Recent Antibiotics Use = 'No' AND Phenotype = ‘Health’) in GMrepo [48]. In total 1168 entries obtained from the above query from 6 different studies including male and female donors from 5 countries worldwide (Canada, United Republic of Tanzania, Italy, China, and the USA), and 1129 packages of the qualified raw data were successfully downloaded from NCBI using sra toolkit v2.10.8 [95] and used for further analysis. The accession information of the 1129 samples is listed in Table S9. The distribution of novel taxa among metagenomes was estimated by Kraken 2 v2.0.9-beta [96]. A customized Kraken 2 database was constructed for taxonomic annotation by collection of all the representative genomes (*n* = 8377) with defined species designation from GTDB release 95 [97] and combination of them with 102 novel taxon genomes from hGMB to generate the GTDB-species_vhGMB database. Then, the 1129 metagenomes were taxonomically annotated. The abundance of assigned species in each metagenomic sample was estimated using Bracken [98]. Default parameters were used for each software unless otherwise specified.

### Bacterial diversities of different culture collections

We collected the taxonomic information of cultures from five representative large-scale cultivation-based studies (CGR [20], BIO-ML [21], SPORE [23], HBC [24], and Culturomics [25]) of human GM for diversity comparison and determination of the resource overlaps. The taxonomic information of all known species was directly mined from corresponding publications, and the taxonomic names of them were used for further comparison. For those unclassified new isolates without validly published names, their corresponding 16S rRNA gene sequences were used for bacterial diversity comparison. The 16S rRNA gene sequences were either retrieved from publication (Culturomics [25]) or extracted from genome data using RNAmmer v1.2 [93] (for CGR [20], BIO-ML [21], SPORE [23], and HBC [24]). The genome-derived 16S rRNA gene sequences > 1 kb were retained for further analysis. The 16S rRNA gene sequences of novel taxa isolates/genomes from one study were clustered using Usearch11 (command: -cluster_fast query.fasta -id 0.987 -centroids clustered.16S.fasta -uc clusters.uc) to reveal the nonredundant 16S rRNA gene sequences of species-level novel taxa in each study. There were 68, 100, 141, and 22 novel taxa recovered from genomes for study SPORE [23], CGR [20], HBC [24], and BIO-ML [21], respectively, based on a 16S rRNA gene identity < 98.7% to any known species in the EZBioCloud and the NCBI 16S ribosomal RNA sequence database (Update date: 2020/08/08, number of sequences: 21,632). With this method, we totally recovered 1056 species for Culturomics, 106 for BIO-ML [21], 121 for SPORE [23], 236 for CGR [20], and 319 for HBC [24]. For SPORE [23], CGR [20], and HBC [24], the number of recovered species was a bit less than that was reported in original papers, which was due to the use of different criteria (genome-based ANI or 16S rRNA gene sequence identity) in species identification depending on each work. We then analyzed the overlaps of potentially novel taxa among studies. The 16S rRNA gene sequences representing novel taxa in each study were combined together, and the Kimura 2-parameter model–based evolution distance between 16S rRNA gene sequences was calculated using MEGA7 [80]. If the new isolates from different studies had 16S rRNA gene sequence distance < 0.013 to each other, they were regarded as the “shared” species by those studies; otherwise, the isolates were defined as study-unique novel taxa. To display hGMB coverage of Human Microbiome Project’s Most Wanted taxa [47], the OTU sequences of the “Most Wanted” taxa analysis were collected and used for BLAST analysis against the 16S rRNA gene sequences of hGMB members with Blastn v2.9.0+ [99]. If the 16S rRNA gene sequences of hGMB members had sequence identities > 98.7% to the OTUs representing taxa of high and middle priority defined in previous work, then the corresponding hGMB members were considered as cultured “most wanted” taxa and indicated in Table S7 (Column named as “Most wanted” taxa). All the taxa included by hGMB were exhibited as taxonomic cladogram using GraPhlAn v1.1.3 [100], and the species presenting exclusively in hGMB were displayed as the outer ring of the cladogram. The unique and shared bacteria within hGMB and five investigated collections were displayed using Venn and bar charts generated by Jvenn [101]. Default parameters were used for each software unless otherwise specified.

### The 16S rRNA gene amplicon data collection and analysis

We collected 26 publicly available 16S rRNA gene amplicon datasets from NCBI SRA database. The accessions, sample size, location, host phenotype, and other basic information of the 26 NCBI Bioprojects are given in Table S8. To enable an equally weighted representation of human GMs, the 26 studies were separately processed and quality-controlled by 64-bit Usearch v11 [102] following the recommended uparse-based pipeline (https://drive5.com/usearch/manual/uparse_pipeline.html). The only modification of the procedure was that an additional chimera removal step was introduced after OTU sequences were generated with the command “-uchime2_ref” against SILVA v132 database. After the generation of an OTU table for each study, the samples maintaining < 10,000 reads were removed. As a result, 11,647 out of the 13,055 samples from 26 studies were retained for further analysis, and each sample contained 228 ± 85 OTUs. The OTU sequences of each study were then annotated using a customized database LTP_vhGMB developed by the update of the LTP database v132 [16] with the taxonomic information of 102 novel taxa in hGMB. The RA and FO of annotated species, genera, and families for each separate study and for all the 26 studies together were calculated as described in our previous publication [36]. The equally weighted average values (RA and FO) were further calculated by averaging the mean values of each study. All the mean values of RAs and FOs relating to the 26 studies were presented as the equally weighted average values ± standard deviation (SD) unless otherwise specified. The equally weighted average RA > 0.1% was the criterion to define dominant species/genera, while the equally weighted average FOs > 30% was the criteria for definition of common species/genera in global human GMs. The saturability of sampled studies were calculated using the specaccum function in the vegan R package [103] and displayed as accumulating curves. The distribution of dominant taxa in global human GMs was displayed as box-and-whiskers plots while the common taxa were displayed as bar charts.

### Gene catalog construction and analysis

The representative metagenome-based human gut Integrated Gene Catalog (IGC) [49] containing over 9.3 million nonredundant genes, the largest-to-date genome-based Unified Human Gastrointestinal Protein (UHGP) catalog [8, 10] comprising 13 million nonredundant protein sequences and the Function Unknown Families of homologous proteins (FUnkFams) catalogs [50] comprising 61,970 amino acid sequences from 6668 conserved protein families were downloaded and reannotated with eggNOG database v4.5 by emapper v1.0.3 (-m diamond) [15] and generated indexed databases for each gene catalogs with DIAMOND v0.9.24 (makedb command) [104]. The nonredundant gene catalog hGMB.catalog was constructed using 115 genomes sequenced in this study by CD-HIT software v4.5.8 [105] (-o out.file -c 0.95 -aS 0.9 -n 5 -M 64000 -T 48). The hGMB.catalog containing 341,876 nonredundant genes were then annotated with eggNOG database v4.5 by emapper v1.0.3 [15]. The eggNOG orthologs, COG categories, KOs, GOs and functionally unknown genes were summarized from the eggNOG annotation results. It revealed that 69.0% of genes in the IGC catalog were annotated into seed eggNOG orthologs, 59.7% into COGs, 38.4% into KOs, and 19.3% into GOs (Gene Orthologs). For all proteins of the UHGP catalog, 69.4%, 60.2%, 39.5%, and 20.9% of the UHGP-90 sequences (sequences clustered at 90% identity) were annotated into seed eggNOG orthologs, COGs, KOs, and GOs, respectively. The identities of KOs and GOs in ICG and UHGP catalogs and in hGMB genomes were extracted. For the calculation of gene coverage (%), the profiles of annotated genes in different gene catalogs and single genomes were tabularized in the form of presence/absence binary code (0/1), which were further calculated using the specaccum function in the vegan R package [103] to generate data used for the construction of cumulative curves. The BLAST analysis of single genomes in hGMB and hGMB gene catalogs against the IGC, UHGP, and FUnkFams catalogs were performed using DIAMOND blastp (--query-cover 70 -id 40 --more-sensitive -f 6 qseqid sseqid pident length qlen slen qcovhsp evalue qseq full_sseq mismatch gapopen qstart qend sstart send). The coverage rates of hGMB.catalog to the global gene catalogs were calculated with two different cutoff values of the amino acid sequence identity 60% and 40%, respectively. The 40% was the threshold identity value of Structural Classification of Proteins (SCOP), while 60% was the minimum amino acid sequence identity for function conservation [106–108].

To profile the coverage of functionally unknown genes of IGC, UHGP, and FUnkFams by hGMB genomes, DIAMOND-based BLAST analysis [104] of single genomes in hGMB against three gene catalogs were performed as described in the last paragraph with a sequence identity cutoff value of 40% and query coverage cutoff value of 70%. The presence of each covered unannotated gene in 115 hGMB genomes was profiled as Tables S12, S13, S14. The ratios of unannotated genes of genomes in hGMB were calculated based on the eggNOG annotations of single genomes. The unannotated rates between two groups were displayed as box and whiskers plots.

### Statistical analysis

All statistical analyses were performed with IBM SPSS Statistics 20. All the box and whiskers plots, bar charts, and accumulative curves were generated using Graphpad Prism v6 [109] unless indicated otherwise. Comparison of two groups of data was statistically assessed with the Mann–Whitney *U* test, while a comparison of multi groups (>2) of data was evaluated by the Kruskal–Willis test. *p* < 0.05 was considered being statistically significant (*p* < 0.05: *, *p* < 0.01: **, *p* < 0.001: ***). The RA, FO, and coverage values relating to 26 amplicon studies were exhibited in the forms of equally weighted average values ± SD. All the other calculations were expressed in the form of mean ± SD unless indicated otherwise. The boxplots showed the median values and whiskers extending to include all the valid data denoted by the Tukey test.

## Supplementary Information

**Additional file 1: Figure S1.** The phylogenetic tree of 108 novel taxon candidates. The phylogenetic tree was constructed with the 16S rRNA gene sequences of each strain using MEGA7 [80] under the neighbor-joining method. The bootstrap value is 1000. The ANIs between adjacent taxa on the tree was calculated using OrthoANI OTA solfware [80] and list in the panel (red color). The names of 102 novel-taxon candidates that were later identified to represent novel taxa were colored in blue, while the 6 candidates that were later determined to be new strains of known species were colored in grey and the 16S rRNA gene identity to the known species were listed in the brackets.

**Additional file 2: Tables S1.** The overlapping new taxa within three cultivation-based studies.

**Additional file 3: Table S2.** The basic information of sample mixtures and the donors.

**Additional file 4: Table S3.** The pretreatments used for large-scale human gut microbe isolation.

**Additional file 5: Table S4.** The media and culture conditions used for large-scale human gut microbe isolation.

**Additional file 6: Table S5.** The taxonomic information of all 10,558 isolates. The 16S rRNA gene identities were derived by BLAST against the NCBI 16S rRNA gene sequence database.

**Additional file 7: Table S6.** The quality and assembly information of hGMB genomes.

**Additional file 8: Table S7.** The taxonomic and 16S rRNA gene information of hGMB.

**Additional file 9: Table S8.** Gerneral information of 26 16S rRNA gene amplicon studies as specified in the table.

**Additional file 10: Table S9.** The accessions and information of 1129 globle human gut metagenomes.

**Additional file 11: Table S10.** The distribution of new hGMB taxa in global human gut metagenoms and UHGG.

**Additional file 12: Table S11.** The number of genes in hGMB.catalog matched to UHGP, IGC.

**Additional file 13: Table S12.** The profile of unannotated genes in IGC recovered by hGMB genomes.

**Additional file 14: Table S13.** The profile of UHGP unannotated genes recovered by hGMB genomes.

**Additional file 15: Table S14.** The profile of FUnkFam genes recovered by hGMB genomes.

**Additional file 16: Supplementary Methods.** The documentation of detailed recipes and references of basic media used in this study.

**Additional file 17: Supplementary Data 1.** The detailed taxonomic descriptions of novel taxa in hGMB.

## Data Availability

The datasets generated and analyzed in this study are available as the following: Basically, all the descriptive information and data related to 400 hGMB species is available at the hGMB homepage (http://hgmb.nmdc.cn) [45]. The 1170 strains and their 16S rRNA gene sequences were accessible via hGMB special page on CGMCC official website (http://www.cgmcc.net/english/hgmb). The taxonomic descriptions of all novel taxa are also accessible at eLMSG under accessions from MSG071057 to MSG071268, MSG071857 and MSG071858 (link type: https://www.biosino.org/elmsg/record/MSG071057) [46]. All assembled genomes and the raw data obtained in this study are available at NCBI under Bioproject PRJNA656402 (https://www.ncbi.nlm.nih.gov/bioproject/PRJNA656402) [110], NODE with the project accession OEP001106 (https://www.biosino.org/node/project/detail/OEP001106) [111], and NMDC under Project NMDC10014003 (http://hgmb.nmdc.cn/subject/hgmb/download). The sequences of 16S rRNA genes of all taxa in hGMB are deposited in Genbank under Bioproject PRJNA656402 (https://www.ncbi.nlm.nih.gov/bioproject/PRJNA656402) [110], and in NMDC under accessions NMDC10014003 (http://hgmb.nmdc.cn/subject/hgmb) [112]. The gene catalog hGMB.catalog is deposited at the hGMB homepage [45].
