# Correction: Enlightening the taxonomy darkness of human gut microbiomes with a cultured biobank

**DOI:** 10.1186/s40168-022-01370-4

**Published:** 2022-10-03

**Authors:** Chang Liu, Meng-Xuan Du, Rexiding Abuduaini, Hai-Ying Yu, Dan-Hua Li, Yu-Jing Wang, Nan Zhou, Min-Zhi Jiang, Peng-Xia Niu, Shan-Shan Han, Hong-He Chen, Wen-Yu Shi, Linhuan Wu, Yu-Hua Xin, Juncai Ma, Yuguang Zhou, Cheng-Ying Jiang, Hong-Wei Liu, Shuang-Jiang Liu

**Affiliations:** 1 State Key Laboratory of Microbial Resources, Institute of Microbiology, Chinese Academy of Sciences, No.1 Beichenxi Road, Chaoyang District, Beijing, 100101 PR China; 2 Environmental Microbiology Research Center, Institute of Microbiology, Chinese Academy of Sciences, No.1 Beichenxi Road, Chaoyang District, Beijing, 100101 China; 3 University of Chinese Academy of Sciences, Beijing, 100049 China; 4 Microbial Resources and Big Data Center, Institute of Microbiology, Chinese Academy of Sciences, No.1 Beichenxi Road, Chaoyang District, Beijing, 100101 China; 5 China General Microorganism Culture Collection, Institute of Microbiology, Chinese Academy of Sciences, No.1 Beichenxi Road, Chaoyang District, Beijing, 100101 China; 6 State Key Laboratory of Mycology, Institute of Microbiology, Chinese Academy of Sciences, No. 1 Beichenxi Road, Chaoyang District, Beijing, 100101 China

**Correction: Microbiome 9, 119 (2021)**

**https://doi.org/10.1186/s40168-021-01064-3**

Following the publication of the original article [1], the author reported that there is a typo error in Table 1, page 13. The correct GMCC/KCTC/NBRC accessions for “Faecalibacterium hominis” taxonomy shoud be “CGMCC 1.5250” and not “CGMCC 1.52500”
