# Supplementary figures and images for: Enlightening the taxonomy darkness of human gut microbiomes with a cultured biobank
Source: Microbiome. 2021 May 21;9:119. doi: 10.1186/s40168-021-01064-3 (PMC8140505; doi:10.1186/s40168-021-01064-3)

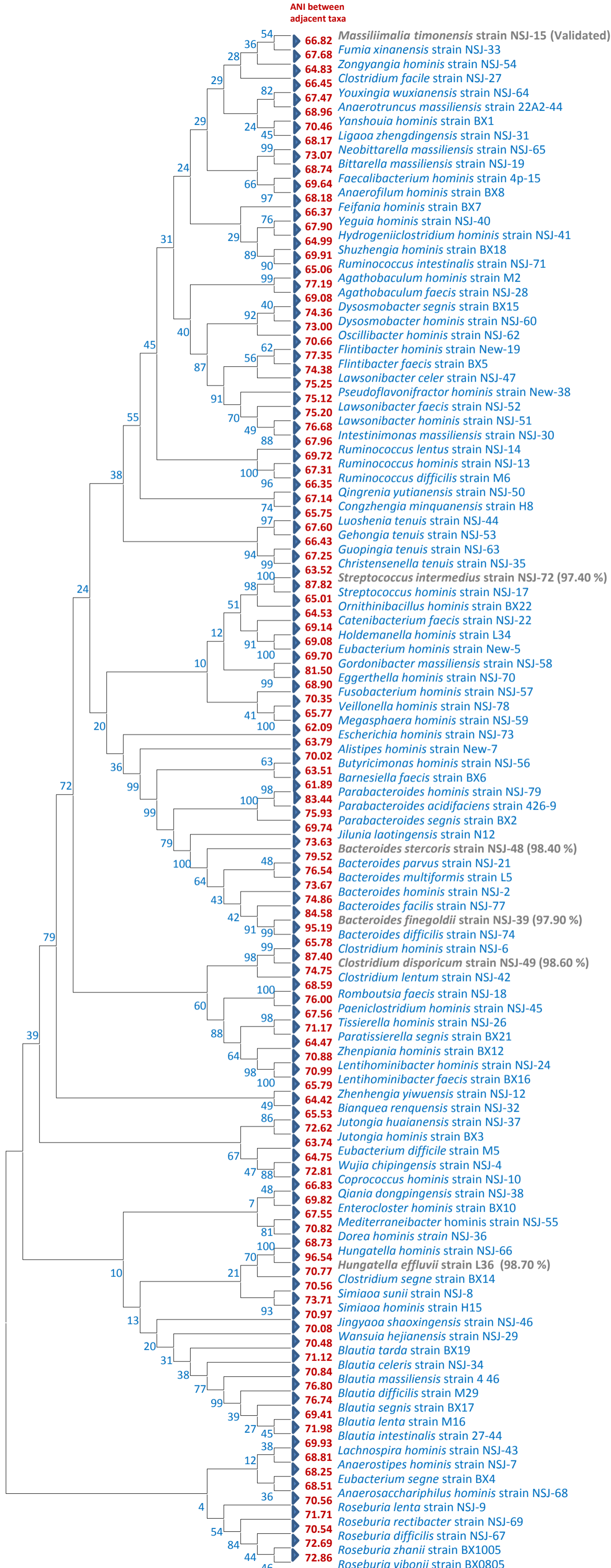

0.20

Supplement: Supplementary file 2 — Additional file 1: Figure S1. The phylogenetic tree of 108 novel taxon candidates. The phylogenetic tree was constructed with the 16S rRNA gene sequences of each strain using MEGA7 [80] under the neighbor-joining method. The bootstrap value is 1000. The ANIs between adjacent taxa on the tree was calculated using OrthoANI OTA solfware [80] and list in the panel (red color). The names of 102 novel-taxon candidates that were later identified to represent novel taxa were colored in blue, while the 6 candidates that were later determined to be new strains of known species were colored in grey and the 16S rRNA gene identity to the known species were listed in the brackets. [file 40168_2021_1064_MOESM2_ESM.pdf]
