# Supplementary material for: Enlightening the taxonomy darkness of human gut microbiomes with a cultured biobank
Source: Microbiome. 2021 May 21;9:119. doi: 10.1186/s40168-021-01064-3 (PMC8140505; doi:10.1186/s40168-021-01064-3)
Supplement: Supplementary file 17 — Additional file 16: Supplementary Methods. The documentation of detailed recipes and references of basic media used in this study. [file 40168_2021_1064_MOESM17_ESM.docx]

**Supplementary Methods**

**Recipes of culture media and supplements:**

**Modified R medium (Derived from R medium ^1^):**

Solution A (900mL):

Casein hydrolysate 5.67 g, Peptone 5 g, Yeast Extract 5 g, Glucose 1 g, Inulin 1 g, D-fructose 1 g, D-(+)-Cellobiose 1 g, NaCl 1.5 g, Hamin(0.1%) 5 mL, Resazurin(0.1%) 1 mL, Rumen fluid 10%, pH 7.2, autoclave at 12 1℃ for 15 min.

Solution B (100mL):

L-cysteine 0.4 g, Ascorbic Acid 1 g, Glutathione 0.1 g, α-Ketoglutarate 2 g, K_2_HPO_4_ 0.45 g, KH_2_PO_4_ 0.9 g, pH 7.2. Filtrate to sterile.

**YCFA medium ^2^:**

Casitone 10 g, Yeast Extract 2.5 g, Glucose 2 g, Sodium Bicarbonate 4 g, Dipotassium Phosphate 0.45g, Monopotassium Phosphate 0.45 g, Sodium Chloride 0.9 g, Magnesium Sulfate Heptahydrate 0.01 g, Calcium Chloride 0.09 g, and Haemin 0.01 g, L-cystein 1 g, 1 mL of Resazurin (0.1 %), [Distilled Water](https://en.wikipedia.org/wiki/Distilled_water) 1 L, pH 7, autoclave at 115 ℃ for 25 min.

**Nutrient agar ^3^：**

[Peptone](https://en.wikipedia.org/wiki/Peptone) 0.05 g, [Beef Extract](https://en.wikipedia.org/wiki/Beef_extract)/[Yeast Extract](https://en.wikipedia.org/wiki/Yeast_extract) 0.03 g, [Agar](https://en.wikipedia.org/wiki/Agar) 0.015 g, [Sodium Chloride](https://en.wikipedia.org/wiki/Sodium_Chloride) 0.05 g, [Distilled Water](https://en.wikipedia.org/wiki/Distilled_water) 1 L, pH 6.8, 121 ℃ for 15 min.

**Schaedler Broth ^4^:**

Casein Peptone 5.66 g, Soy Peptone 1 g, Yeast Extract 5 g, Peptone Mixture 5 g, Glucose 5.83 g, K_2_HPO_4_ 0.83 g, NaCl 1.66 g, Tris (hydroxymethyl aminomethane) 3 g, Resazurin 0.001 g, Hemin 0.01 g, Cysteine-HCl·H_2_O 0.4 g, Distilled Water 1 L.

**MRS medium ^5^:**

Casein Peptone 10 g, Meat Extract 10 g, Yeast Extract 5 g, Glucose 20 g, Tween 80 1 g, K_2_HPO_4_ 2 g, Sodium Acetate 5 g, Triammonium Citrate 2 g, MgSO_4_·7 H_2_O 0.2 g, MnSO_4_·H_2_O 0.05 g, Distilled Water 1 L, pH 6.4±0.2, autoclave at 115 ℃ for 25 min.

**PYG medium ^6^:**

Trypticase Peptone 5 g, Peptone 5 g, Yeast extract 10 g, Beef extract 5 g, Glucose 5 g, K_2_HPO_4_ 2 g, Tween 80 1 mL, Cysteine-HCl·H_2_O 0.5 g, Resazurin 0.001 g, Salt solution (see below) 40 mL, Distilled water 950 mL, Haemin solution (see below) 10 ml. pH 7.2, autoclave at 115 ℃ for 25 min.

Salt solution:

CaCl_2_·2H_2_O 0.25 g, MgSO_4_·7 H_2_O 0.50 g, K_2_HPO_4_ 1 g, KH_2_PO_4_ 1 g, NaHCO_3_ 10 g, NaCl 2 g, Distilled Water 1 L.

Haemin solution:

Dissolve 0.05 g Haemin in 1 mL 1 N NaOH, make up to 100 mL with Distilled Water. Store refrigerated.

Dissolve 0.1 mL of vitamin K1 in 20 mL 95% ethanol and filter sterilize. Store refrigerated.

**MGAM Medium ^7^:**

Casitone 10 g, Soya Petone 3 g, Proteose Peptone 15 g, Digested Serum 13.5 g, Yeast Extract 5 g, Meta Extract 2 g, Liver Extract 1.2 g, Dextrose 3 g, Soluble starch 0.3 g, L-Cysteine Hydrochloride 0.5 g, L-Arginine 0.5 g, L-Tryptophan 0.3 g, NaHCO_3_ 2 g, KH_2_PO_4_ 2.5 g, NaCl 3 g, CH_2_(SH)COONa 0.15 g, CH_3_COONa 2.46 g, Hemin 0.01 g, Resazurin 0.001 g, Distilled Water 1 L, pH 7.2 ±0.1, autoclave at 115 ℃ for 25 min.

**Macconkey Medium ^8^:**

Peptone 20 g, Lactose 10 g, Bile Salt 5 g, NaCl 5 g, Neutral Red 0.075 g, Distilled Water 1 L, pH 7.2 ±0.1, autoclave at 115 ℃ for 25 min.

**Luria Bertani Medium ^9^:**

NaCl 10 g, Peptone 10 g, Yeast Extract 5 g, Distilled Water 1 L, pH 7.2 ±0.1, 121 ℃ for 20 min.

**Fastidious Anaerobe Broth ^10^:**

Mix peptone 15 g, Yeast Extract 10 g, NaCl 2.5 g, Hemin 05 g, Sodium Thioglycolate 0.5 g, V_k_ 0.005 g, Resazurin 0.1 g, NaHCO_3_ 0.4 g, Distilled Water 1 L,pH 7.2±0.2, autoclave at 115 ℃ for 25 min.

**Columbia Blood Medium ^11^:**

Special Peptone 23 g, Starch 1 g, Sodium Chloride 5 g, Sheep Blood 5%, Distilled Water 1 L, pH 7.3±0.2, autoclave at 115 ℃ for 25 min.

**Brucella Broth ^12^:**

Pancreatic Digest of Casein 10 g, Enzymatic Digest of Soybean Meal 10 g, Dextrose 1 g, Yeast Extract 2 g, NaCl 5 g, Sodium Hydrogen Sulfite 0.1 g, Distilled Water 1 L, pH 7.3±0.2, 115 ℃ for 25 min.

**Clostridium Enrichment Medium ^13^ :**

Peptone 10 g,Glucose 5 g, NaCl 5 g, Sodium Acetate 3 g, Yeast Extract 3 g, Beef Leaching Powder 10g, Soluble Starch 1 g, L-Cysteine monohydrochloride 0.5 g, Distilled Water 1 L, pH 6.8±0.2, autoclave at 115 ℃ for 25 min.

**Enterobacteria Enrichment Broth ^14^:**

Gelatin Trypsin Hydrolysate 10 g, Na_2_HPO_4_·2H_2_O 8 g, Bile Salt 20 g, Brilliant Green 0.015 g,Glucose 5 g, KH_2_PO_4_ 2 g, Distilled Water 1 L, pH 7.2±0.2, autoclave at 115 ℃ for 25 min.

**2216E Medium ^15^:**

Peptone 5 g, Yeast Extract 1 g, Ferric Phosphate 0.01 g, AGAR 15 g, Boiling Sodium Hydroxide (5%) to adjust the, Distilled Water 1 L, pH 7.6-7.8, autoclave at 121 ℃ for 20 min.

**Wilkins Chalgren Anaerobe Broth ^16^:**

Casein Hydrolysate 10 g, Pepticdigest of Animal Tissue 10 g, Yeast Extract 5 g, Glucose 1 g,

Distilled Water 1 L, pH 7.2±0.2, autoclave at 115 ℃ for 25 min.

**Deoxycholalate Agar (Hopebio HB0112, China):**

Lactose Peptone 10 g, Sodium Chloride 5 g, Sodium Citrate 1 g, Citric Acid Iron 1 g, Deoxidation Cholic Acid Sodium 1 g, Neutral Red 0.03 g, Potassium Hydrogen Phosphate 2 g, AGAR 13 g, pH 7.3±0.1.

Usage take this article 43 grams, add 1 L distilled water, heat to dissolve doesn't stop stirring, boil for 1 minute, cooled to 45 to 50℃, pour into the aseptic agar, without pressure.

**DZP Mixpeptone Medium:**

Casein Hydrolysate 2 g, Peptone 2 g, Casein Peptone 2 g, Soy Peptone 2 g, Trypticase Peptone 2 g, Peptone Mixture 2 g, Meat Peptone 2 g, Proteose Peptone 2 g, Fish Peptone 2 g, Hipolypepton 2 g, Pig Intestines Extract, Hemin 0.01 g, Resazurin 0.001 g, Clarified Rumen Fluid 10%, pH 7.2 ±0.1, Distilled Water 1 L, 115 ℃ for 25 min, cooled to 45 to 50℃,add Vitamin Solution, Mineral Solution, Small Molecular Acids Mixture.

**Carbohydrate mixture:**

D-mannose 0.5 g, D-fructose 0.5 g, Fructo-oligosaccharide 0.5 g, Synanthrin/Inulin probiotics 0.5 g, D-galactose 0.5 g, Palatinose 0.5 g, L-Rhamnose 0.5 g, D-(+)-Cellobiose 0.5 g, D-Trehalose 0.5 g, Distilled Water 1 L. Filtrate and eliminate bacteria. Store refrigerated.

**Small molecular acids mixture:**

Valeric acid (1mM), isovaleric acid (1mM), propionic acid (9mM), isobutyric acid (1mM), pH 7.0. Filtrate and eliminate bacteria. Store refrigerated.

**Vitamin K1 solution (Hopebio HB8462-a, China):**

Dissolve 0.1 g of Vitamin K1 in 20 mL 95% ethanol and filter sterilize. Store refrigerated.

**Wolfe's Vitamin solution (Coolaber MKC101-50ml, China):**

Biotin 0.002 g, Folic Acid 0.002 g, Pyridoxine Hydrochloride 0.01 g, Thiamine-HCl x 2H_2_O 0.005 g, Riboflavin 0.005 g, Nicotinic Acid 0.005 g, D-Ca-pantothenate 0.005 g, Vitamin B_12_ 0.0001 g, p-Aminobenzoic Acid 0.005 g, Thioctic Acid 0.005 g, Distilled Water 1 L.

**Wolfe's Mineral solution (Coolaber MKC102-50ml, China):**

Nitrilotriacetic Acid 1.5 g, MgSO_4_·7H_2_O 3 g, MnSO_4_·H_2_O 0.5 g, NaCl 1 g, FeSO_4_·7H_2_O 0.1 g, CoCl_2_·6H_2_O 0.1 g, CaCl_2_ 0.1 g, ZnSO_4_·7H_2_O 0.1 g, CuSO_4_·5H_2_O 0.01 g, ALK(SO_4_)_2_·12H_2_O 0.01 g, H_3_BO_3_ 0.01 g, Na_2_MoO_4_·2H_2_O 0.01 g, Distilled Water 1 L.

**Reference**

1 Dione, N., Khelaifia, S., La Scola, B., Lagier, J. & Raoult, D. A quasi-universal medium to break the aerobic/anaerobic bacterial culture dichotomy in clinical microbiology. *Clin Microbiol Infec* **22**, 53-58 (2016).

2 Browne, H. P. et al. Culturing of ‘unculturable’ human microbiota reveals novel taxa and extensive sporulation. Nature 533, 543 (2016).

3 Awaad, A. S., AL-Mudhayyif, H. A., Al-Othman, M. R., Zain, M. E. & El-Meligy, R. M. Amhezole, A Novel Fungal Secondary Metabolite from Aspergillus terreus for Treatment of Microbial Mouth Infection. *Phytotherapy Research* **31**, 395-402, doi:10.1002/ptr.5760 (2017).

4 Stalons, D. R., Thornsberry, C. & Jr, D. V. Effect of Culture Medium and Carbon Dioxide Concentration on Growth of Anaerobic Bacteria Commonly Encountered in Clinical Specimens. *Appl Microbiol* **27**, 1098-1104 (1974).

5 De Man, J., Rogosa, d. & Sharpe, M. E. A medium for the cultivation of lactobacilli. *Journal of applied Bacteriology* **23**, 130-135 (1960).

6 Bergmanson, J. P., Wang, E., Gire, A. I. & Osato, M. S. In vitro effects of medium tonicity, nutrient concentration, and free chlorine content on Acanthamoeba. *Contact Lens and Anterior Eye* **34**, 164-168 (2011).

7 Rettedal, E. A., Gumpert, H. & Sommer, M. O. A. Cultivation-based multiplex phenotyping of human gut microbiota allows targeted recovery of previously uncultured bacteria. Nat. Commun. 5, 4714 (2014)

8 MacConkey, A. Lactose-fermenting bacteria in faeces. *Epidemiology & Infection* **5**, 333-379 (1905).

9 Bertani, G. Studies on lysogenesis I.: the mode of phage liberation by lysogenic Escherichia coli1. *Journal of bacteriology* **62**, 293 (1951).

10 Ganguli, L. A., Turton, L. J. & Tillotson, G. S. Evaluation of Fastidious Anaerobe Broth as a blood culture medium. *J Clin Pathol* **35**, 458-461 (1982).

11 Dusch, H., Zbinden, R. & Von Graevenitz, A. Growth differences of Capnocytophaga canimorsus strains and some other fastidious organisms on various Columbia-based blood agar media. *Zentralblatt für Bakteriologie* **282**, 362-366 (1995).

12 NIROOMAND, F. & FUNG, D. Y. EFFECT OF OXYGEN REDUCING MEMBRANE FRAGMENTS ON GROWTH OF CAMPYLOBACTER SPP. 1. *Journal of Rapid Methods & Automation in Microbiology* **2**, 247-277 (1993).

13 PEARLJEET, K. A. *Effect of Substrate Concentration in Producing Higher Butanol Compared to Ethanol by Using Clostridium Acetobutylicum*, University Malaysia Pahang, (2010).

14 Choi, D. *et al.* Improvement of Enterobacteriaceae enrichment broth by supplementation with sodium citrate for detection of Cronobacter sakazakii using real-time PCR. *Food science and biotechnology* **25**, 1205-1209 (2016).

15 Zhang, Z. *et al.* Bioactive bafilomycins and a new N-Arylpyrazinone derivative from marine-derived Streptomyces sp. HZP-2216E. *Planta medica* **83**, 1405-1411 (2017).

16 Buck, G. E. & Kelly, M. T. Susceptibility testing of Campylobacter fetus subsp. jejuni, using broth microdilution panels. *Antimicrob Agents Ch* **21**, 274-277 (1982).
