# Supplementary material for: Enlightening the taxonomy darkness of human gut microbiomes with a cultured biobank
Source: Microbiome. 2021 May 21;9:119. doi: 10.1186/s40168-021-01064-3 (PMC8140505; doi:10.1186/s40168-021-01064-3)
Supplement: Supplementary file 18 — Additional file 17: Supplementary Data 1. The detailed taxonomic descriptions of novel taxa in hGMB. [file 40168_2021_1064_MOESM18_ESM.pdf]

Supplementary Data 1

Taxon 1: *Yeguia hominis* gen.nov. sp. nov.

***Description of Yeguiaceae* fam.nov.**

Yeguiaceae (Ye.gui'a'ce.ae. N. L. neut. n. *Yeguia*, type genus of the family. -aceae, ending to denote a family,N. L. fem. pl. n. *Yeguiaceae*, family of the genus *Yeguia*)
This family is affiliated with the order *Clostridiales* in the class *Clostridia*. This family encompasses bacteria that divide by binary fission. Cells are oval shaped with peaked or spiky ends, and appear singly or in dividing pairs. Affiliation of new species to this family should depend on their phylogenetic position based on the 16S rRNA gene and genomic DNA idendity. The detailed description is as the same as that for type genus *Yeguia*.

***Description of Yeguia* gen.nov.**

Yeguia (Ye.gui'a N.L. fem. n. *Yeguia*, named in honour of the Chinese medical scientist Gui Ye)
The closest phylogenetic neighbor of Taxon 1 as represented by strain NSJ-40^T, is *Clostridium jeddahense* (Figure SD-1.1a). *C. jeddahense* and strain NSJ-40^T (16S rRNA sequence accession number in NMDC (National Microbiology Data Center) is NMDCN0000101) has 16S rRNA gene identity of 91.97 %, and their DNA G+C content is different by 11.68 %. The genome of strain NSJ-40^T was sequenced, and the NMDC accession number is NMDC60014001. Genome-based analysis on *C. jeddahense* strain and type strain NSJ-40^T reveals that the dDDH and ANI values between *C. jeddahense* JCD^T (NZ ABGD00000000) and strain NSJ-40^T are 12.70 % and 72.19 %, respectively, and the POCP value is 31.62 %, suggesting *C. jeddahense* and strain NSJ-40^T are from different genera. According to the phylogenominc tree (Figure SD-1c), the ANIs and dDDH values of genomes between strain NSJ-40^T (NMDC accession number is NMDCN0000101) and phylogenomically neighbored genomes were calculated, and the highest ANI and dDDH values are 69.8 and 34.8 %, respectively, between the query genome of *Caproiciproducens galactitolivorans* (NZ_SRMQ00000000.1); and difference in % G+C between genomes of strain NSJ-40^T and *Caproiciproducens galactitolivorans* (NZ_SRMQ00000000.1) is 3.02; OrthoANI heatmap (Figure SD-1d) shows the phylogenomic status of the corresponding neighbor species based on OrthoANI values. Cells of strain NSJ-40^T are oval to short rod shaped. The G+C content is around 63.58 mol %. The type species is *Yeguia hominis.*

Description of *Yeguia hominis* sp. nov.

Yeguia hominis sp. nov. (ho'mi.nis L. gen. masc. n. *hominis*, of a human being, referring to the human gut habitat)
Cells are strictly anaerobic,oval shaped with peaked ends (1.3-1.8 µm long by 0.7-0.9

µm wide, Figure SD-1.1b); cells appear singly or in dividing pairs. Pinpoint, irregular, cream-colored, moist, colonies appear on modified mGAM agar plate after 3 days of incubation. Growth occurs 37 °C and at pH 7.0-7.5. The organism uses succinic acid mono-methyl ester, L-phenylalanine and L-serine as the carbon source for growth, and weakly metabolizes N-acetyl-D glucosamine, N-acetyl-β-D mannosamine, glyoxylic acid, thymidine-5'-monophosphate, succinamic acid, L-valine plus L-methionine and L-aspartic acid. The G+C content of the type strain NSJ-40^T is around 63.58 mol %. The type strain NSJ-40^T (CGMCC= 1.32813^T) was isolated from the faeces of a healthy adult.

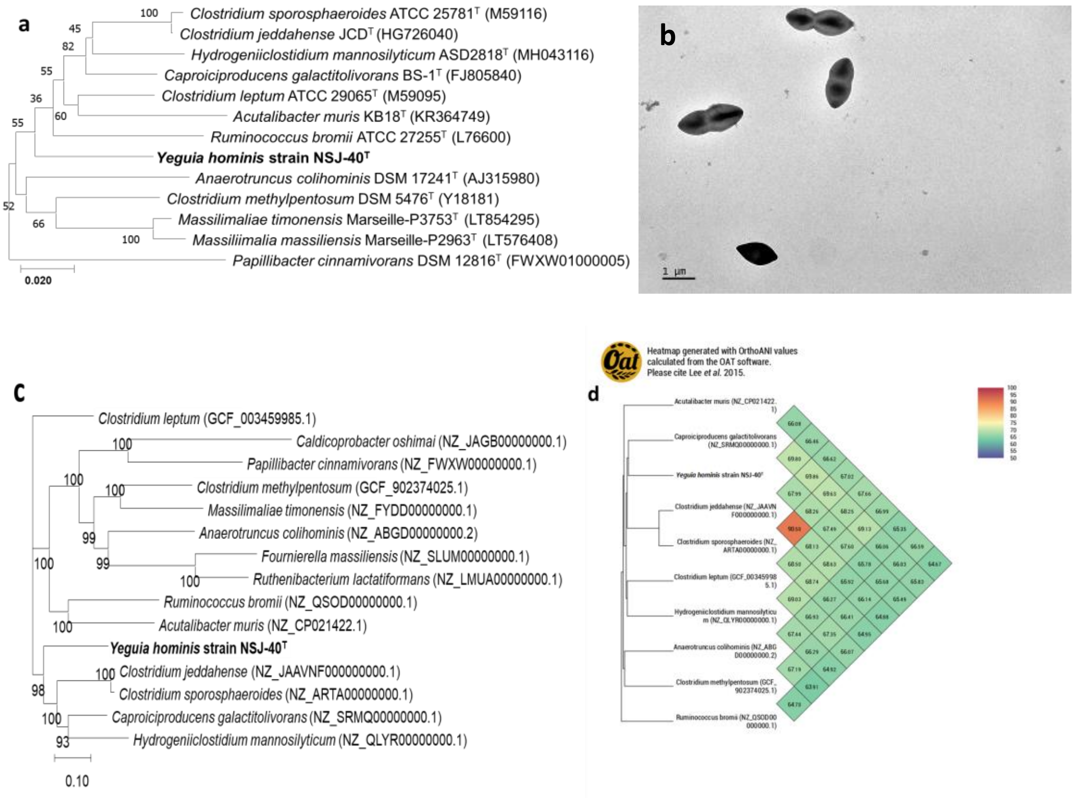

Figure SD-1. The Neighbour-joining phylogenetic tree based on 16S rRNA gene sequences (a) and the cell morphology (b) of strain NSJ-40^T. GenBank accession numbers are given in parentheses. Percentages of bootstrap support are shown at branch nodes. Bootstrap value was 1000. Bar: 0.02 substitutions per nucleotide position. The phylogenominc tree based on gemome-sequence-alignment (c) and OrthoANI heatmap (d) of strain NSJ-40^T and its neighbor species. Refseq ID of representative genomes are given in parentheses. Percentages of bootstrap support are shown at branch nodes. Bar: 0.1 substitutions per nucleotide position.

Taxon 2: *Luoshenia tenuis* gen. nov. sp. nov.

Description of *Luoshenia* gen. nov. (Luo.shen'ia N.L. fem. n. *Luoshenia*, named after the Chinese Goddess Luoshen)

The closest phylogenetic neighbor of Taxon 2 as represented by strain NSJ-44^T is *Christensenella minuta* (Figure SD-2.1a). *C. minuta* and strain NSJ-44^T (16S rRNA sequence accession number in NMDC is NMDCN0000102) has 16S rRNA gene identity of 87.07 %, and their DNA G+C content is different by 9.52 %. The strain NSJ-44^T can not cluster with the *Christensenella* members or any type species in *Christensenellaceae* family. The genome of strain NSJ-44^T was sequenced and the NMDC accession number is NMDC60014002. Genome-based analysis on *C. minuta* DSM 22607^T (NZ CP029256) and type strain NSJ-44^T reveals that the dDDH and ANI values between *C. minuta* DSM 22607^T and strain NSJ-44^T are 24.90 % and 63.17 %, respectively, and the POCP value is 33.28 %, suggesting *C. minuta* and strain NSJ-44^T are from different genera. According to the phylogenominc tree (Figure SD-2c), the ANIs and dDDH values of genomes between strain NSJ-44^T (NMDC accession number is NMDC60014002) and phylogenomically neighbored genomes were calculated, and the highest ANI and dDDH values are 66.63 and 18.3 %, respectively, between the query genome of *Gehongia tenuis* strain NSJ-53^T (NMDC60014005); and difference in % G+C between genomes of strain NSJ-44^T and *Gehongia tenuis* strain NSJ-53^T (NMDC60014005) is 1.16; OrthoANI heatmap (Figure SD-2d) shows the phylogenomic status of the corresponding neighbor species based on OrthoANI values. Cells of the strain NSJ-44^T are ovoids with spiky ends. The G+C content is around 61.02 mol %. The type species is *Luoshenia tenuis.*

Description of *Luoshenia tenuis* sp. nov. (te'nu.is. L. fem. adj. *tenuis*, thin, slim, referring to the predicted potential function of the strain in weight-loss)

Cells are strictly anaerobic are ovoids with spiky ends (1.3-1.6 µm long by 0.7-0.9 µm wide, Figure SD-2.1b); cells appear singly or in dividing pairs. Extremely small, pinpoint, whitish to semi-translucent, entire, convex colonies appear on modified mGAM agar plate after 3 days of incubation. Growth occurs at 37°C and at pH range of 7.0-7.5. The organism uses adonitol, D-arabitol, arbutin, D-cellobiose, dextrin, D-fructose, L-fucose, D-galactose, D-galacturonic acid, gentiobiose, D-gluconic acid, D-glucosaminic acid, α-D-glucose, glucose-6-phosphate, lactulose, maltose, maltotriose, D-mannose, D-melibiose, 3-melthyl-D-glucose, palatinose, L-rhamnose, turanose, glyoxylic acid, α-ketobutyric acid, α-ketovaleric acid, pyruvic acid, pyruvic acidmethyl ester, inosine and uridine as carbon source for growth, and weakly metabolizes amygdalin, α-cyclodextrin, dulcitol, i-erythritol, glycerol, m-inositol, α-D-lactose, α-methyl-D-galactoside, ß-methyl-D-galactoside, ß -methyl-D-glucoside, salicin, D-mannitol, D-melezitose, D-sorbitol, acetic acid, formic acid, L- malic acid, propionic acid and L-valine plus L-aspartic acid. The G+C content of the type strain NSJ-44^T is around 61.02 mol %. The type strain NSJ-44^T (=CGMCC 1.32817^T) was isolated from the faeces of a healthy adult.

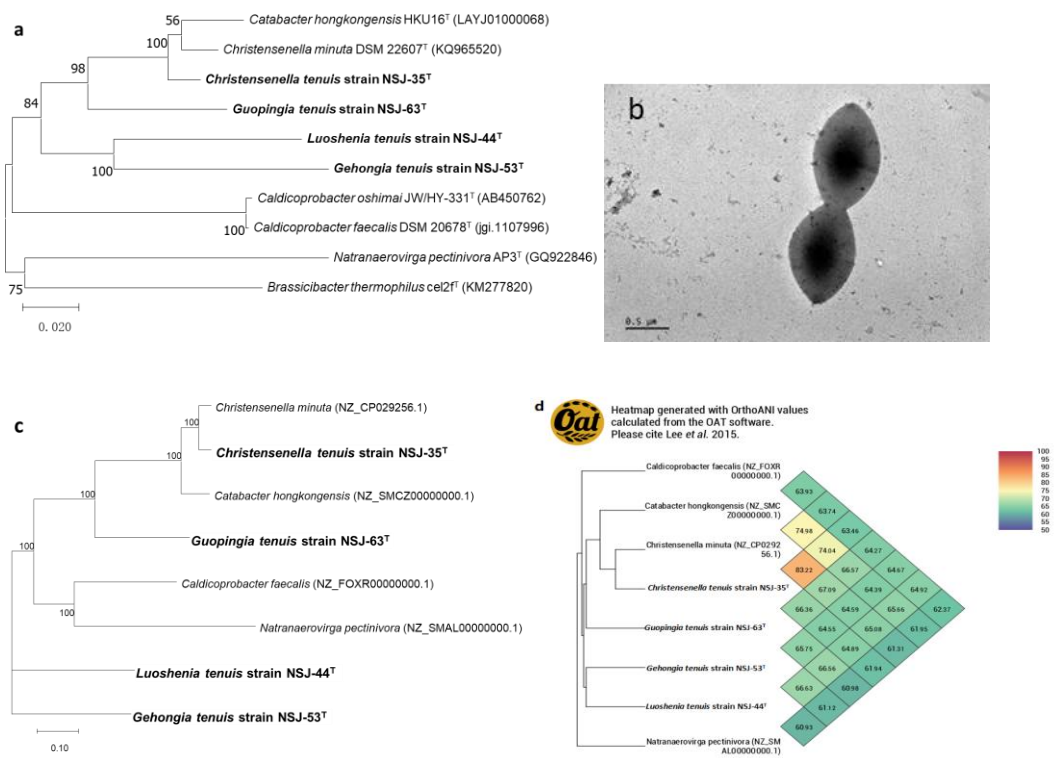

Figure SD-2. The Neighbour-joining phylogenetic tree based on 16S rRNA gene sequences (a) and the cell morphology (b) of strain NSJ-44^{T}. GenBank accession numbers are given in parentheses. Percentages of bootstrap support are shown at branch nodes. Bootstrap value was 1000. Bar: 0.020 substitutions per nucleotide position. The phylogenominc tree based on gemome-sequence-alignment (c) and OrthoANI heatmap (d) of strain NSJ-44^{T} and its neighbor species. Refseq ID of representative genomes are given in parentheses. Percentages of bootstrap support are shown at branch nodes. Bar: 0.10 substitutions per nucleotide position.

Taxon 3: *Feifania hominis* gen.nov.sp. nov.

Description of Feifaniaceae fam.nov.

Feifaniaceae (Fei.fa.ni.a.ce'ae. N.L. fem. n. *Feifania*, type genus of the family. -aceae, ending to denote a family. N. L. fem. pl. n. *Feifaniaceae*, family of the genus *Feifania*). This family is affiliated with the order *Clostridiales* in the class *Clostridia.*This family encompasses bacteria that divide by binary fission. Cells are rod shaped and mostly appear singly. Affiliation of new species to this family should depend on their phylogenetic position based on the 16S rRNA gene and genomic DNA idendity. The detailed description is as the same as that for type genus *Feifania*.

***Description of Feifania* gen.nov**

Feifania (Fei.fa'ni.a N.L. fem. n. *Feifania*, named after the Chinese microbiologist Feifan Tang)
The closest phylogenetic neighbor of Taxon 3 as represented by strain $BX7^T$, is *Papillibacter cinnamivorans* (Figure SD-3a). *P. cinnamivorans* and strain $BX7^T$ (16S rRNA sequence accession number in NMDC is NMDCN0000103) has 16S rRNA gene identity of 90.73 %, and their DNA G+C content is different by 3 %. The genome of strain $BX7^T$ was sequenced, and the NMDC accession number is NMDC60014003. Genome-based analysis on *P. cinnamivorans* strain and type strain $BX7^T$ reveals that the dDDH and ANI values between *P. cinnamivorans* DSM 12816^T (NZ FWXW00000000) and strain $BX7^T$ are 27.40 % and 65.50 %, respectively, and the POCP value is 34.96 %, suggesting *P. cinnamivorans* and strain $BX7^T$ are from different genera. According to the phylogenominc tree (Figure SD-3c), the ANIs and dDDH values of genomes between strain $BX7^T$ (NMDC accession number is NMDC60014003) and phylogenomically neighbored genomes were calculated, and the highest ANI and dDDH values are 66.89 and 22.2 %, respectively, between the query genome of *Lawsonibacter asaccharolyticus* (NZ_BFBT00000000.1); and difference in % G+C between genomes of strain $BX7^T$ and *Lawsonibacter asaccharolyticus* (NZ_BFBT00000000.1) is 0.47; OrthoANI heatmap (Figure SD-3d) shows the phylogenomic status of the corresponding neighbor species based on OrthoANI values. Cells of strain $BX7^T$ are rod shaped. The G+C content is around 58.8 mol %. The type species is *Feifania hominis.*

Description of *Feifania hominis* sp. nov.
Feifania hominis sp. nov. (ho'mi.nis. L. gen. masc. n. *hominis*, of a human being, referring to the human gut habitat)
Cells are strictly anaerobic rods (1.6-2.9 µm long by 0.9-1.1 µm wide, Figure SD-3b); cells appear singly. Growth occurs at 37°C and at pH range of 7.0-7.5. The G+C content of the type strain $BX7^T$ is 58.8 mol %. The type species is *Feifania hominis.* The type strain $BX7^T$ (=CGMCC 1.32862^T) was isolated from the faeces of a healthy adult.

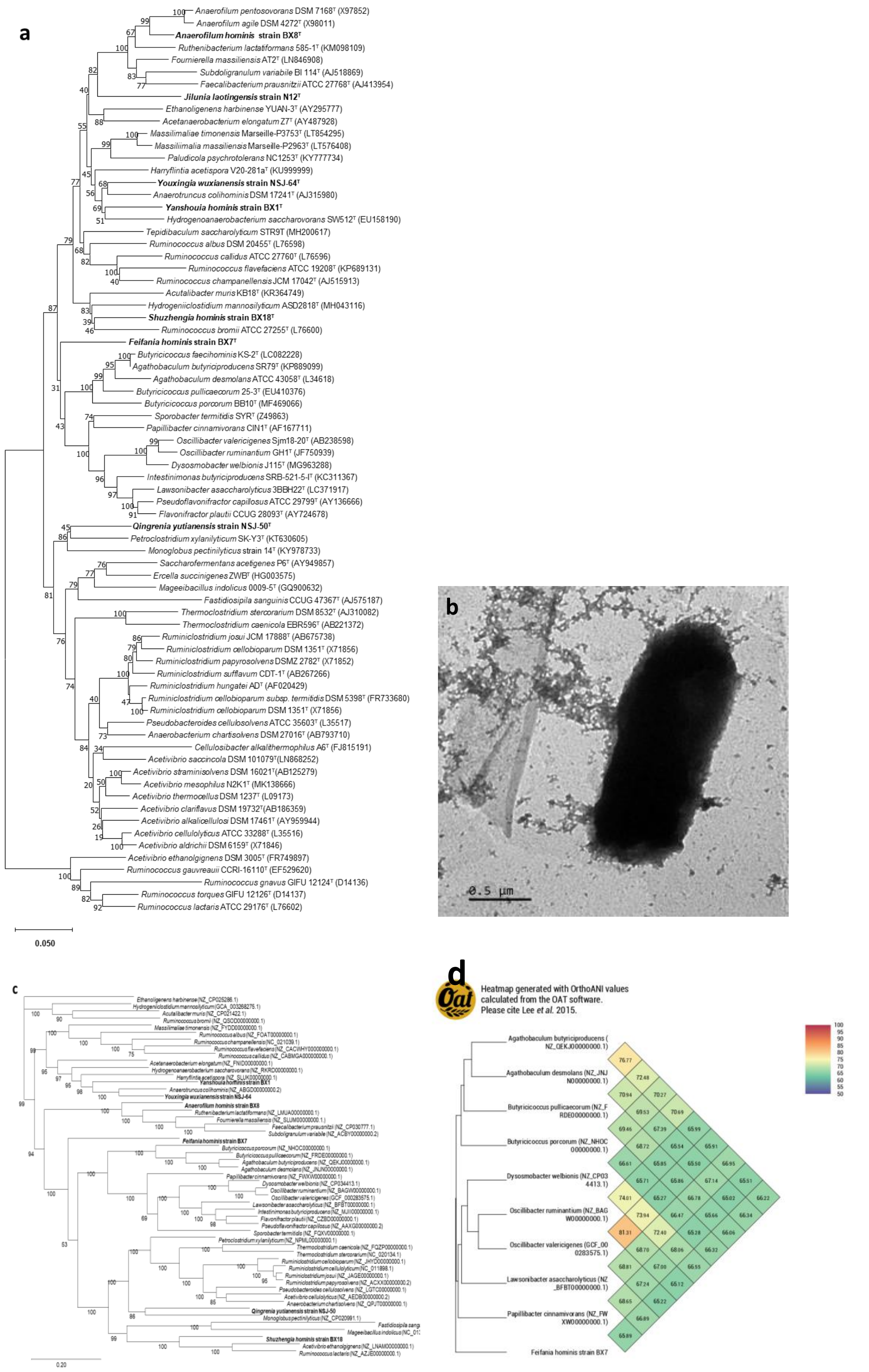

Figure SD-3. The Neighbour-joining phylogenetic tree based on 16S rRNA gene

sequences (a) and the cell morphology (b) of strain BX7^T. GenBank accession numbers are given in parentheses. Percentages of bootstrap support are shown at branch nodes. Bootstrap value was 1000. Bar: 0.05 substitutions per nucleotide position. The phylogenominc tree based on gemome-sequence-alignment (c) and OrthoANI heatmap (d) of strain BX7^T and its neighbor species. Refseq ID of representative genomes are given in parentheses. Percentages of bootstrap support are shown at branch nodes. Bar: 0.20 substitutions per nucleotide position.

Taxon 4: *Bianqueaceae* fam.nov.

Description of *Bianqueaceae* fam.nov.

Bianqueaceae (Bian.qu.a’ce.ae. N. L. neut. n. *Bianquea*, type genus of the family; -aceae, ending to denote a family; N. L. fem. pl. n. *Bianqueaceae*, family of the genus *Bianquea*).

This family is affiliated with the order *Clostridiales* in the class *Clostridia.*This family encompasses bacteria that divide by binary fission. Cells are rod shaped. Affiliation of new species to this family should depend on their phylogenetic position based on the 16S rRNA gene and genomic DNA identity. The type genus is *Bianquea*.

***Bianquea renquensis* gen. nov. sp. nov.**

Bianquea (Bian.que’a N.L. fem. n. *Bianquea*, named after the Chinese medical scientist Que Bian)

The closest phylogenetic neighbor of Taxon 4 as represented by strain NSJ-32^T, is *Abyssivirga alkaniphila* (Figure SD-4a). *A. alkaniphila* and strain NSJ-32^T (16S rRNA sequence accession number in NMDC is NMDCN0000104) has 16S rRNA gene identity of 87.63 %. The genome of strain NSJ-32^T was sequenced, the NMDC accession number is NMDC60014004, and their DNA G+C content is different by 30.12 %. The strain NSJ-32^T can not cluster with the members of genus *Abyssivirga* or any type species of genera within *Lachnospiraceae* family. Genome-based analysis on *A. alkaniphila* L81^T and type strain NSJ-32^T reveals that the dDDH and ANI values between *A. alkaniphila* L81^T and strain NSJ-32^T are 30.80 % and 67.19 %, respectively, and the POCP value is 9.8 %, suggesting *A. alkaniphila* and strain NSJ-32^T are from different genera. According to the phylogenominc tree (Figure SD-4c), the ANIs and dDDH values of genomes between strain NSJ-32^T (NMDC accession number is NMDC60014004) and phylogenomically neighbored genomes were calculated, and the highest ANI and dDDH values are 65.46 and 25.9 %, respectively, between the query genome of *Anaerotignum propionicum* (NZ_CP014223.1); and difference in % G+C between genomes of strain NSJ-32^T and *Anaerotignum propionicum* (NZ_CP014223.1) is 10.92; OrthoANI heatmap (Figure SD-4d) shows the phylogenomic status of the corresponding neighbor species based on OrthoANI values. Cells of the strain NSJ-32^T are straight or slightly curved rods. The G+C content is around 61.32 mol %. The type species is *Bianquea renquensis.*

Description of *Bianquea renquensis* sp. nov. (ren.qu.en'sis. N.L. fem. adj. *renquensis*, pertaining Renqiu county of China, the birthplace of Chinese medical scientist QueBian)

Cells are strictly anaerobic, straight or slightly curved rods (2.1-3.8 µm long by 0.7-0.9 µm wide, Figure SD-4b); cells appear singly or in dividing pairs. White to translucent, pinpoint colonies appear on modified mGAM agar plate after 2 days of incubation. Growth occurs range of 37 °C and at pH range of 7.0-7.5. The organism uses N-acetyl-D glucosamine, amygdalin, arbutin, D-fructose, L-fucose, D-galacturonic acid, gentiobiose, α-D-glucose, D-mannose, 3-melthyl-D-glucose, palatinose,L-rhamnose,

salicin, sucrose, glyoxylic acid, pyruvic acid and inosine as the carbon source for growth, and weakly metabolizes D-cellobiose, dextrin, D-galactose, D-gluconic acid, glucose-6-phosphate, maltose, D-raffinose, thymidine-5'-monophosphate and α-ketobutyric acid. The G+C content of the type strain NSJ-32^T is around 61.32 mol %. The type strain NSJ-32^T (=CGMCC 1.32805^T) was isolated from the faeces of a healthy adult.

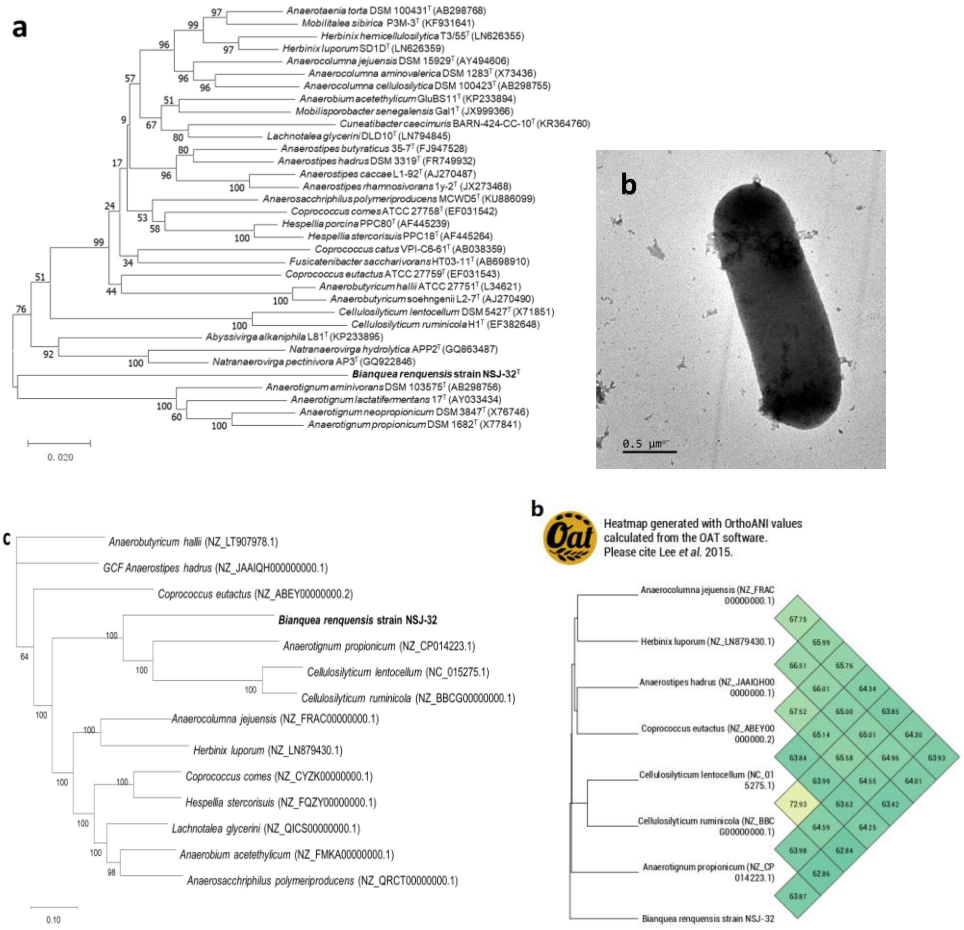

Figure SD-4. The Neighbour-joining phylogenetic tree based on 16S rRNA gene sequences (a) and the cell morphology (b) of strain NSJ-32^T. GenBank accession numbers are given in parentheses. Percentages of bootstrap support are shown at branch nodes. Bootstrap value was 1000. Bar: 0.02 substitutions per nucleotide position. The phylogenomin c tree based on gemome-sequence-alignment (c) and OrthoANI heatmap (d) of strain NSJ-32^T and its neighbor species. Refseq ID of representative genomes are given in parentheses. Percentages of bootstrap support are shown at branch nodes. Bar: 0.1 substitutions per nucleotide position.

Taxon 5: *Gehongia tenuis* gen. nov. sp. nov.

Description of *Gehongia* gen. nov. (Ge.hong'ia N.L. fem. n. *Gehongia*, named after Ge Hong (284-364 AD), a Chinese medical scientist, who for the first time described the use of fecal suspension by mouth for treatment of severe diarrhea or food poisoning in his writing "Zhou Hou Bei Ji Fang (A Handbook of Formulas for Emergencies)", which is known as the first literature describing the Fecal Microbiota Transplantation)

The closest phylogenetic neighbor of Taxon 5 as represented by strain NSJ-53^T, is *Christensenella minuta* (Figure SD-5a). The genome of strain NSJ-53^T was sequenced, the NMDC accession number is NMDC60014005. *Christensenella minuta* and strain NSJ-53^T (16S rRNA sequence accession number in NMDC is NMDCN0000105) has 16S rRNA gene identity of 86.82 %, and their DNA G+C content is different by 7.5 %. The strain NSJ-53^T can not cluster with the *Christensenella* members or any type species of genera in *Christensenellaceae* family. Genome-based analysis on *C. minuta* DSM 22607^T (NZ CP029256) and type strain NSJ-53^T reveals that the dDDH and ANI values between *C. minuta* DSM 22607^T and strain NSJ-53^T are 27.90 % and 71.09 %, respectively, and the POCP value is 32.23 %, suggesting *C. minuta* and strain NSJ-53^T are from different genera. According to the phylogenominc tree (Figure SD-5c), the ANIs and dDDH values of genomes between strain NSJ-53^T (NMDC accession number is NMDC60014005) and phylogenomically neighbored genomes were calculated, and the highest ANI and dDDH values are 66.63 and 18.3 %, respectively, between the query genome of *Luoshenia tenuis* strain NSJ-44^T (NMDC60014002); and difference in % G+C between genomes of strain NSJ-53^T and *Luoshenia tenuis* strain NSJ-44^T (NMDC60014002) is 1.16; OrthoANI heatmap (Figure SD-5d) shows the phylogenomic status of the corresponding neighbor species based on OrthoANI values.Cells of the strain NSJ-53^T are oval to short rods. The G+C content is around 59.0 mol %. The type species is *Gehongia tenuis.*

Description of *Gehongia tenuis* sp. nov. (te'nu.is L. masc./fem. adj. *tenuis*, thin, slim, referring to the predicted potential function of the strain in weight-loss)

Cells are strictly anaerobic, ovoid to short rod shaped (1.3-2.0 µm long by 0.7-0.9 µm wide, Figure SD-5b); cells appear singly or in dividing pairs. Circular, white to translucent, slightly raised pinpoint colonies appear on modified mGAM agar plate after 3 days of incubation. Growth occurs at 37°C and at pH range of 7.0-7.5. The organism uses N-acetyl-D galactosamine, N-acetyl-D glucosamine, N-acetyl-β-D mannosamine, adonitol, D-arabitol, ß-cyclodextrin, dextrin, i-erythritol, L-fucose, D-galacturonic acid, D-gluconic acid, D-glucosaminic acid, D-melezitose, L-rhamnose, D-sorbitol, sucrose, acetic acid, formic acid, D-lactic acidmethyl ester, D-malic acid, L-malic acid, L-alanyl-L-histidine, L-phenylalanine, L-serine, L-valine, L-valine plus L-aspartic acid, 2'-deoxyadenosine, inosine, thymidine and uridine as the carbon source for growth, and weakly metabolizes glycerol, 3-melthyl-D-glucose, ß-methyl-D-galactoside, α-methyl-D glucoside, glyoxylic acid, α-ketobutyric acid and α-ketovaleric acid. The G+C content of the type strain NSJ-53^T is around 59.00 mol %. The type strain NSJ-53^T (=CGMCC 1.32829^T =KCTC 25141^T) was isolated from the

faeces of a healthy adult.

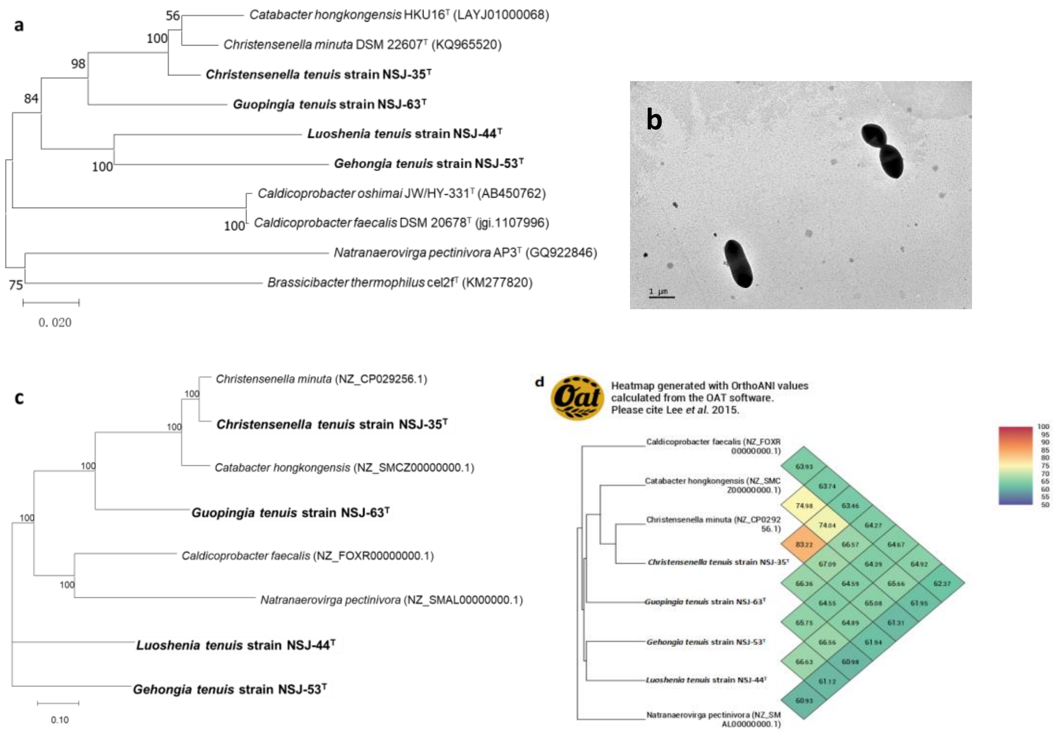

Figure SD-5. The Neighbour-joining phylogenetic tree based on 16S rRNA gene sequences (a) and the cell morphology (b) of strain NSJ-53^{T}. GenBank accession numbers are given in parentheses. Percentages of bootstrap support are shown at branch nodes. Bootstrap value was 1000. Bar: 0.02 substitutions per nucleotide position. The phylogenominc tree based on gemome-sequence-alignment (c) and OrthoANI heatmap (d) of *Gehongia tenuis* strain NSJ-53^{T} and its neighbor species. Refseq ID of representative genomes are given in parentheses. Percentages of bootstrap support are shown at branch nodes. Bar: 0.10 substitutions per nucleotide position.

Taxon 6: *Guopingia tenuis* gen. nov. sp. nov.

Description of *Guopingia* gen. nov.

Guopingia(Guo.ping'i.a. N.L. fem. n. *Guopingia*, named after the Chinese microbiologist Guoping Zhao)

The closest phylogenetic neighbor of Taxon 6 as represented by strain NSJ-63^T, is *Christensenella minuta*(Figure SD-6a). The genome of strain NSJ-63^T was sequenced, the NMDC accession number is NMDC60014006. *Christensenella minuta* and strain NSJ-63^T (16S rRNA sequence accession number in NMDC is NMDCN0000106) has 16S rRNA gene identity of 91.45 %, and their DNA G+C content is different by 1.8 %. The strain NSJ-63^T can not cluster with the *Christensenella* members or any type species of genera in *Christensenellaceae* family. Genome-based analysis on *C. minuta* DSM 22607^T (NZ CP029256) and type strain NSJ-63^T reveals that the dDDH and ANI values between *C. minuta* DSM 22607^T and strain NSJ-63^T are 22.50 % and 68.87 %, respectively, and the POCP value is 38.48 %, suggesting *C. minuta* and strain NSJ-63^T are from different genera. According to the phylogenominc tree (Figure SD-6c), the ANIs and dDDH values of genomes between strain NSJ-63^T (NMDC accession number is NMDC60014006) and phylogenomically neighbored genomes were calculated, and the highest ANI and dDDH values are 67.09 and 36.1 %, respectively, between the query genome of *Christensenella minuta* (NZ_CP029256.1); and difference in % G+C between genomes of strain NSJ-63^T and *Christensenella minuta* (NZ_CP029256.1) is 1.8; OrthoANI heatmap (Figure SD-6d) shows the phylogenomic status of the corresponding neighbor species based on OrthoANI values. Cells of the strain NSJ-63^T are spherical in shape. The G+C content is around 53.30 mol %. The type species is *Guopingia tenuis.*

Description of *Guopingia tenuis* sp. nov. (te'nu.is. L. fem. adj. tenuis, thin, slim, referring to the predicted potential function of the strain in weight-loss)

Cells are strictly anaerobic, spherical in shape (diameter 0.8-1.1µm, Figure SD-6b); cells appear singly or in dividing pairs. Irregular, pinpoint, ivory, moist, convex colonies appear on modified mGAM agar plate after 6 days of incubation. Growth occurs at 37°C and at pH range of 7.0-7.5. The organism uses D-fructose, L-fucose, D-galactose, D-galacturonic acid, α-D-glucose, glycerol, D-mannose, 3-melthyl-D-glucose, palatinose, L-rhamnose, glyoxylic acid and pyruvic acid, and weakly metabolizes gentiobiose, glucose-6-phosphate and D-melibiose. The G+C content of the type strain NSJ-63^T is around 53.30 mol %. The type strain NSJ-63^T (=CGMCC 1.32839^T) was isolated from the faeces of a healthy adult.

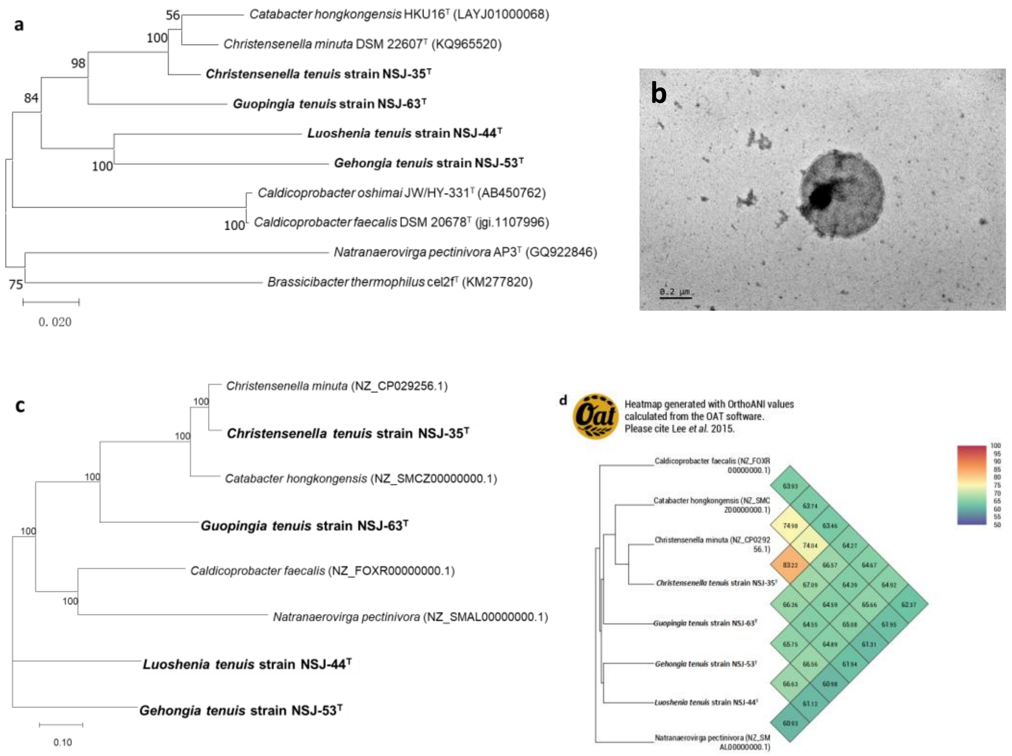

Figure SD-6. The Neighbour-joining phylogenetic tree based on 16S rRNA gene sequences (a) and the cell morphology (b) of strain NSJ-63^{T}. GenBank accession numbers are given in parentheses. Percentages of bootstrap support are shown at branch nodes. Bootstrap value was 1000. Bar: 0.020 substitutions per nucleotide position. The phylogenominc tree based on gemome-sequence-alignment (c) and OrthoANI heatmap (d) of strain NSJ-63^{T} and its neighbor species. Refseq ID of representative genomes are given in parentheses. Percentages of bootstrap support are shown at branch nodes. Bar: 0.10 substitutions per nucleotide position.

Taxon 7: *Ligaoa zhengdingensis* gen. nov. sp. nov.

Description of *Ligaoa* gen. nov.

Ligaoa (Li.gao'a N.L. fem. n. *Ligaoa*, named in honour of the Chinese medical scientis Gao Li)

The closest phylogenetic neighbor of Taxon 7 as represented by strain NSJ-31^T, is *Hydrogenoanaerobacterium saccharovorans* (Figure SD-7a). *H. saccharovorans* and strain NSJ-31^T (16S rRNA sequence accession number in NMDC is NMDCN0000107) has 16S rRNA gene identity of 92.16 %, and their DNA G+C content is different by 22.17 %. The genome of strain NSJ-31^T was sequenced, and the NMDC accession number is NMDC60014007. Genome-based analysis on *H. saccharovorans* strain and type strain NSJ-31^T reveals that the dDDH and ANI values between *H. saccharovorans* DSM 24774^T (NZ RKRD00000000) and strain NSJ-31^T are 18.40 % and 76.89 %, respectively, and the POCP value is 52.54 %, suggesting *H. saccharovorans* and strain NSJ-31^T are from different genera of the family *Oscillospiraceae*. According to the phylogenominc tree (Figure SD-7c), the ANIs and dDDH values of genomes between strain NSJ-31^T (NMDC accession number is NMDC60014007) and phylogenomically neighbored genomes were calculated, and the highest ANI and dDDH values are 70.77 and 18.5 %, respectively, between the query genome of *Hydrogenoanaerobacterium saccharovorans* (NZ_RKRD00000000.1); and difference in % G+C between genomes of strain NSJ-31^T and *Hydrogenoanaerobacterium saccharovorans* (NZ_RKRD00000000.1) is 14.35; OrthoANI heatmap (Figure SD-7d) shows the phylogenomic status of the corresponding neighbor species based on OrthoANI values. Cells of the strain NSJ-31^T are spherical in shape. The G+C content is around 64.87 mol %. The type species is *Ligaoa zhengdingensis.*

Description of *Ligaoa zhengdingensis* sp. nov.

***Ligaoa zhengdingensis* sp. nov.** (zheng.ding.en'sis. N.L. fem. adj. *zhengdingensis*, referring to Zhengding county of China, the birthplace of Li Gao)

Cells are strictly anaerobic, spherical in shape (diameter 0.9-1.2 µm, Figure SD-7b); cells appear singly. Growth occurs at 37°C and at pH 7.0-7.5. The organism uses D-cellobiose, dextrin, D-fructose, L-fucose, D-galactose, D-galacturonic acid, pyruvic acidmethyl ester, gentiobiose, α-D-glucose, glucose-1-phosphate, glucose-6-phosphate, α-D-lactose, lactulose, maltotriose, D-mannose, D-melibiose, 3-melthyl-D-glucose, ß-methyl-D-galactoside, palatinose, L-rhamnose, turanose, glyoxylic acid, α-ketobutyric acid, α-ketovaleric acid, D-lactic acidmethyl ester,pyruvic acid, L-glutamic acid, L-serine, L-valine plus L-aspartic acid, 2'-deoxyadenosine, inosine, thymidine, uridine and uridine-5'-monophosphate as the carbon source for growth, and weakly metabolizes D-gluconic acid, glycerol, maltose, D-melezitose, α-methyl-D-galactoside, D-sorbitol, formic acid, L-malic acid, propionic acid, L-alanyl-L-glutamine, L-phenylalanine and thymidine-5'-monophosphate. The G+C content of the type strain NSJ-31^T is around 64.87 mol %. The type strain NSJ-31^T

(=CGMCC 1.32804^T) was isolated from the faeces of a healthy adult.

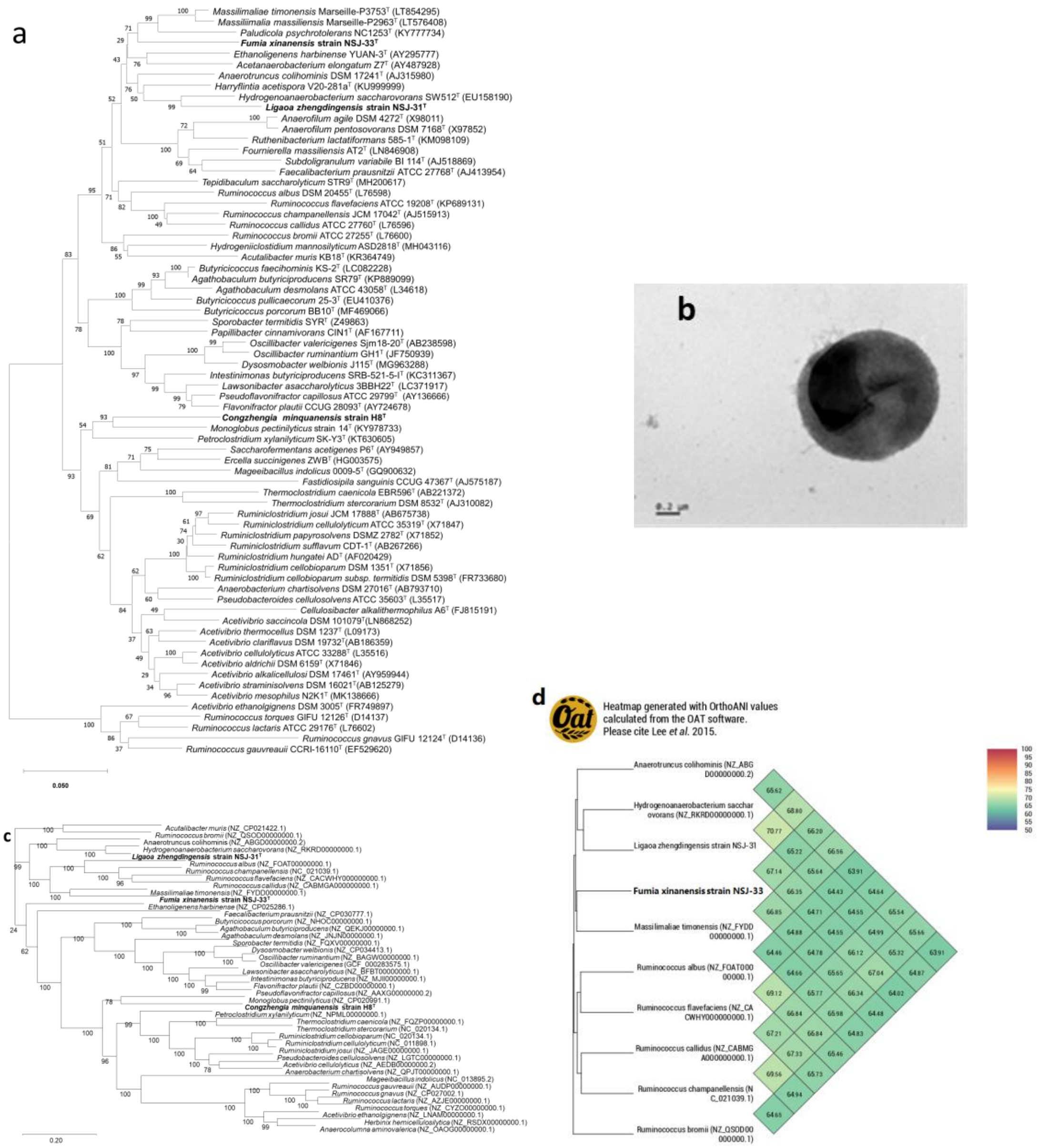

Figure SD-7. The Neighbour-joining phylogenetic tree based on 16S rRNA gene sequences (a) and the cell morphology (b) of strain NSJ-31^T. GenBank accession numbers are given in parentheses. Percentages of bootstrap support are shown at branch nodes. Bootstrap value was 1000. Bar: 0.05 substitutions per nucleotide position. The phylogenominc tree based on gemome-sequence-alignment (c) and OrthoANI heatmap (d) of strain NSJ-31^T and its neighbor species. Refseq ID of representative genomes are given in parentheses. Percentages of bootstrap support are shown at branch nodes. Bar: 0.20 substitutions per nucleotide position.

Taxon 8: *Congzhengia minquanensis* gen. nov. sp. nov.

Description of *Congzhengia* gen. nov.

Congzhengia (Cong.zheng'i.a. N.L. fem. n. *Congzhengia*, named after the Chinese medical scientist Congzheng Zhang)
The closest phylogenetic neighbor of Taxon 8 as represented by strain H8^T, is *Monoglobus pectinilyticus* (Figure SD-8a). *M. pectinilyticus* and strain H8^T (16S rRNA sequence accession number in NMDC is NMDCN0000108) has 16S rRNA gene identity of 90.28 %, and their DNA G+C content is different by 12.79 %. The genome of strain H8^T was sequenced, and the NMDC accession number is NMDC60014008. Genome-based analysis on *M. pectinilyticus* strain and type strain H8^T reveals that the dDDH and ANI values between *M. pectinilyticus* DSM 14^T (NZ CP020991) and strain H8^T are 31.30 % and 64.89 %, respectively, and the POCP value is 34.91 %, suggesting *M. pectinilyticus* and strain H8^T are from different genera of the family *Oscillospiraceae*. According to the phylogenominc tree (Figure SD-8c), the ANIs and dDDH values of genomes between strain H8^T (NMDC accession number is NMDC60014008) and phylogenomically neighbored genomes were calculated, and the highest ANI and dDDH values are 65.11 and 31.3 %, respectively, between the query genome of *Monoglobus pectinilyticus* (NZ_CP020991.1); and difference in % G+C between genomes of strain H8^T and *Monoglobus pectinilyticus* (NZ_CP020991.1) is 7.53; OrthoANI heatmap (Figure SD-8d) shows the phylogenomic status of the corresponding neighbor species based on OrthoANI values. Cells of strain H8^T are spherical or short rod shaped. The G+C content is around 50.19 mol %. The type species is *Congzhengia minquanensis.*

Description of *Congzhengia minquanensis* sp. nov.

***Congzhengia minquanensis* sp. nov.** (min.quan.en'sis. N.L. fem. adj. *minquanensis*, referring to Minquan county of China, the birthplace of Congzheng Zhang)
Cells are strictly anaerobic, spherical or short rod shaped (diameter 0.6-1.17 µm, Figure SD-8b); cells appear singly or in (dividing) pairs. Growth occurs at 37°C and at pH 7.0-7.5. The organism uses arbutin, D-cellobiose, dextrin, D-fructose, L-fucose, D-galactose, D-galacturonic acid, gentiobiose, D-gluconic acid, D-glucosaminic acid, α-D-glucose, glucose-6-phosphate, glycerol, lactulose, maltotriose, D-mannose, D-melibiose, 3-melthyl-D-glucose, α-methyl-D-galactoside, palatinose, D-raffinose, L-rhamnose, salicin, stachyose, sucrose, turanose, glyoxylic acid, α-ketobutyric acid, α-ketovaleric acid, propionic acid, pyruvic acid, pyruvic acidmethyl ester, L-alaninamide, L-methionine, L-phenylalanine, L-valine, L-valine plus and L-aspartic acid as carbon source for growth, and weakly metabolizes N-acetyl-D galactosamine, N-acetyl-D glucosamine, N-acetyl-β-D mannosamine, adonitol, amygdalin, D-arabitol, i-erythritol, α-D-lactose, ß-methyl-D-galactoside, D-sorbitol, D-trehalose, acetic acid, formic acid, L-malic acid, D-saccharic acid, m-tartaric acid and L-serine. The G+C content of the type strain H8^T is around 50.19 mol %. The type strain H8^T (=CGMCC

1.32875^{T}) was isolated from the faeces of a healthy adult.

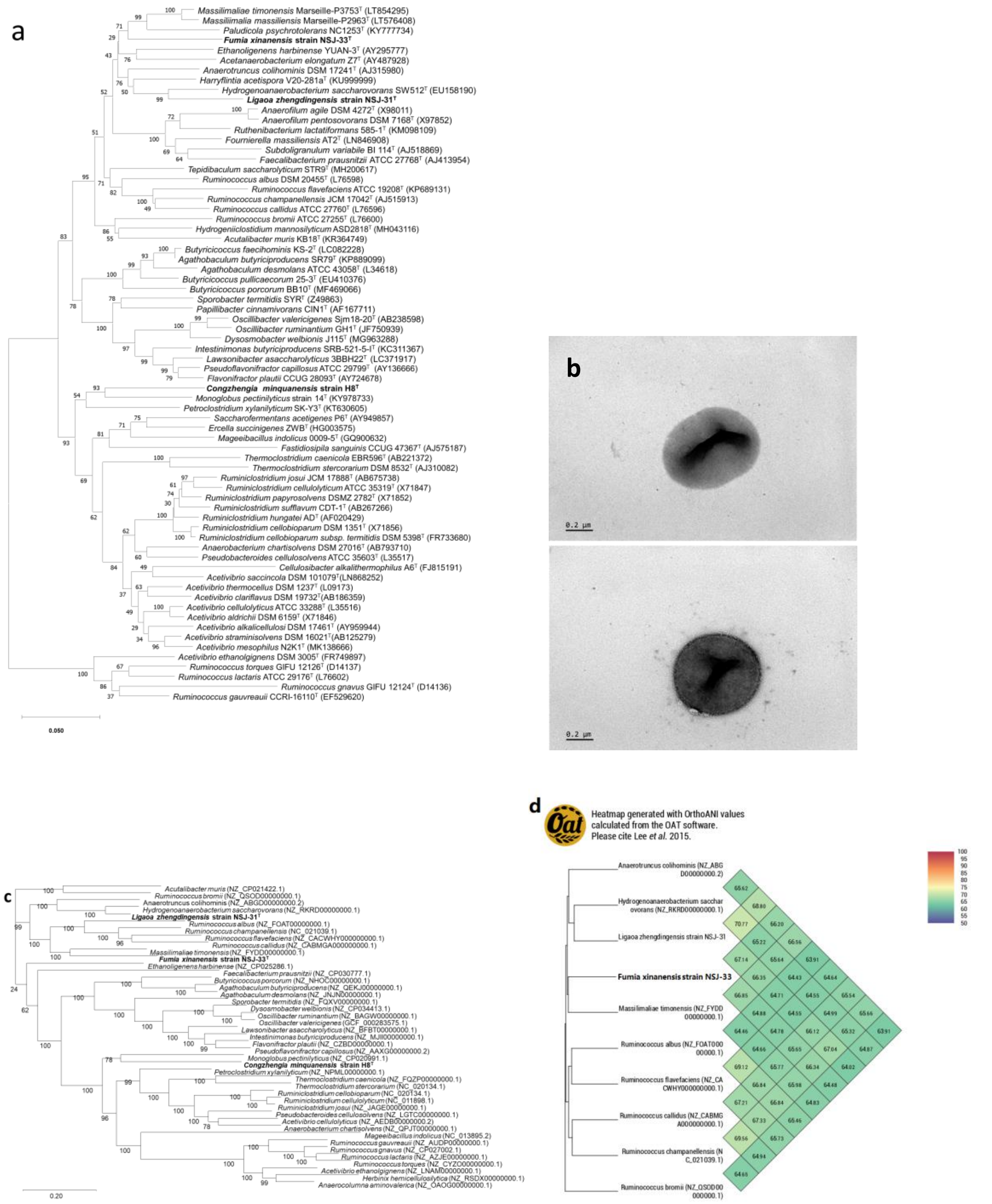

Figure SD-8. The Neighbour-joining phylogenetic tree based on 16S rRNA gene sequences (a) and the cell morphology (b) of strain H8^{T}. GenBank accession numbers are given in parentheses. Percentages of bootstrap support are shown at branch nodes. Bootstrap value was 1000. Bar: 0.05 substitutions per nucleotide position. The phylogenominc tree based on gemome-sequence-alignment (c) and OrthoANI heatmap (d) of strain H8^{T} and its neighbor species. Refseq ID of representative genomes are given in parentheses. Percentages of bootstrap support are shown at branch nodes. Bar: 0.20 substitutions per nucleotide position.

Taxon 9: *Fumia xinanensis* gen. nov. sp. nov.

Description of *Fumia* gen. nov. (Fu.mi’a N.L. fem. n. *Fumia*, named in honour of the Chinese medical scientis Fumi Huang)

The closest phylogenetic neighbor of Taxon 9 as represented by strain NSJ-33^T, is *Massiliimalia timonensis* (Figure SD-9a). *M. timonensis* and strain NSJ-33^T (16S rRNA sequence accession number in NMDC is NMDCN0000109) has 16S rRNA gene identity of 90.95 %, and their DNA G+C content is different by 12.79 %. The genome of strain NSJ-33^T was sequenced, and the NMDC accession number is NMDC60014009. Genome-based analysis on *M. timonensis* strain and type strain NSJ-33^T reveals that the dDDH and ANI values between *M. timonensis* Marseille-P3753^T (NZ ABGD00000000) and strain NSJ-33^T are 39.40 % and 69.57 %, respectively, and the POCP value is 33.96 %, suggesting *M. timonensis* and strain NSJ-33^T are from different genera of the family *Oscillospiraceae*. According to the phylogenominc tree (Figure SD-9c), the ANIs and dDDH values of genomes between strain NSJ-33^T (NMDC accession number is NMDC60014009) and phylogenomically neighbored genomes were calculated, and the highest ANI and dDDH values are 67.14 and 27 %, respectively, between the query genome of strain NSJ-31^T (NMDC60014007); and difference in % G+C between genomes of strain NSJ-33^T and *Ligaoa zhengdingensis* strain NSJ-31^T (NMDC60014007) is 7.53; OrthoANI heatmap (Figure SD-9d) shows the phylogenomic status of the corresponding neighbor species based on OrthoANI values. Cells of the strain NSJ-33^T are oval to short rod shaped. The G+C content is around 51.32 mol %. The type species is *Fumia xinanensis.*

Description of *Fumia xinanensis* sp. nov. (xin.an.en'sis. N.L. fem. adj. *xinanensis*, referring to Xin'an county where Fumi Huang was born)

Cells are strictly anaerobic, oval to short rod shaped (1.4-2.2 µm long by 0.8-1.0 µm wide, Figure SD-9b); cells mostly appear singly. Tiny, circular, cream-colored, moist, raised colonies appear on modified mGAM agar plate after 1 day of incubation. Growth occurs at 37°C and at pH 7.0-7.5. The organism uses N-acetyl-D galactosamine, N-acetyl-D glucosamine, N-acetyl-β-D mannosamine, amygdalin, arbutin, dextrin, D-fructose, L-fucose, D-galactose, D-galacturonic acid, gentiobiose, D-gluconic acid, α-D-glucose, glucose-6-phosphate, α-D-lactose, lactulose, D-mannose, D-melezitose, D-melibiose, 3-melthyl-D-glucose, α-methyl-D-galactoside, ß-methyl-D-galactoside, α-methyl-D glucoside, palatinose, D-raffinose, L-rhamnose, salicin, turanose, glyoxylic acid, α-ketobutyric acid, α-ketovaleric acid, pyruvic acid, pyruvic acidmethyl ester, L-alaninamide, L-alanine, L-methionine, L-phenylalanine, L-serine, L-threonine, L-valine, L-valine plus L-aspartic acid, inosine and uridine as the carbon source for growth, and weakly metabolizes D-cellobiose, ß-cyclodextrin, maltose, ß-methyl-D-glucoside and glycyl-L-proline. The G+C content of the type strain NSJ-33^T is around 51.32 mol %. The type strain NSJ-33^T (=CGMCC 1.32806^T) was isolated from the faeces of a healthy adult.

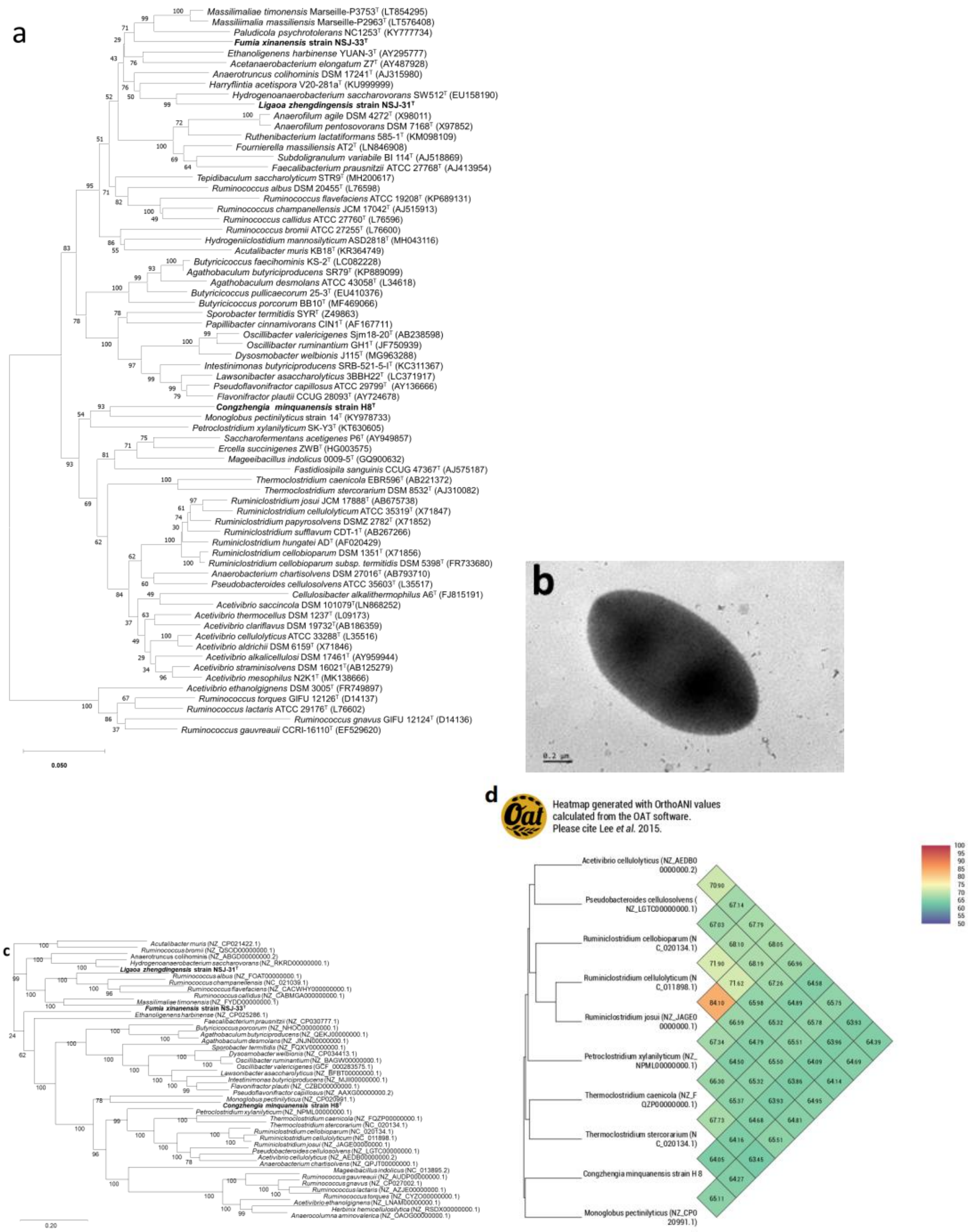

Figure SD-9. The Neighbour-joining phylogenetic tree based on 16S rRNA gene sequences (a) and the cell morphology (b) of strain NSJ-33^T. GenBank accession numbers are given in parentheses. Percentages of bootstrap support are shown at branch nodes. Bootstrap value was 1000. Bar: 0.05 substitutions per nucleotide position. The phylogenominc tree based on gemome-sequence-alignment (c) and OrthoANI heatmap (d) of strain NSJ-33^T and its neighbor species. Refseq ID of representative genomes are given in parentheses. Percentages of bootstrap support are shown at branch nodes. Bar: 0.20 substitutions per nucleotide position.

Taxon 10: *Wujia chipingensis* gen. nov. sp. nov.

Description of *Wujia* gen. nov. (Wu.ji'a N.L. fem. n. *Wujia*, named in honour of the Chinese medical scientis Wuji Cheng)

The closest phylogenetic neighbor of Taxon 10 as represented by strain NSJ-4^T, is *Coprococcus eutactus* (Figure SD-10a). *C. eutactus* and strain NSJ-4^T (16S rRNA sequence accession number in NMDC is NMDCN000010A) has 16S rRNA gene identity of 95.13 %, and their DNA G+C content is different by 0.82 %. The genome of strain NSJ-4^T was sequenced, and the NMDC accession number is NMDC60014010, and genome-based analysis on *Coprococcus eutactus* strain and type strain NSJ-4^T reveals that the dDDH and ANI values between *Coprococcus eutactus* ATCC 27759^T (NZ_ABEY00000000.2) and strain NSJ-4^T are 22.80 % and 71.79 %, respectively, suggesting *Coprococcus eutactus* and strain NSJ-4^T are from different genera in *Lachnospiraceae* family. According to the phylogenominc tree (Figure SD-10c), the ANIs and dDDH values of genomes between strain NSJ-4^T (NMDC accession number is NMDC60014010) and phylogenomically neighbored genomes were calculated, and the highest ANI and dDDH values are 71.45 and 33 %, respectively, between the query genome of *Coprococcus eutactus* (NZ ABEY00000000.2); and difference in % G+C between genomes of strain NSJ-4^T and *Coprococcus eutactus* (NZ ABEY00000000.2) is 0.83; OrthoANI heatmap (Figure SD-10d) shows the phylogenomic status of the corresponding neighbor species based on OrthoANI values. Cells of strain NSJ-4^T are oval or short rod shaped with flagella. The G+C content is around 43.92 mol %. The type species is *Wujia chipingensis.*

Description of *Wujia chipingensis* sp. nov. (chi.ping'en.sis. N.L. fem. adj. chipingensis, referring to Chiping county of China, the birthplace of the Chinese medical scientist Wuji Cheng)

Cells are strictly anaerobic, oval or short rod shaped with flagella (1.2-1.6 µm long by 0.8-1.0 µm wide, Figure SD-10b); cells appear singly or in dividing pairs. Growth occurs at 37°C and at pH range of 7.0-7.5. The organism uses D-fructose, L-fucose, D-galacturonic acid, 3-melthyl-D-glucose, palatinose 6-O-D-fructofuranose as the carbon source for growth, and weakly metabolizes dextrin, D-mannose, L-rhamnose, pyruvic acid and pyruvic acid methyl ester. The G+C content of the type strain NSJ-4^T is around 43.92 mol %. The type strain NSJ-4^T (=CGMCC 1.5256^T) was isolated from the faeces of a healthy adult.

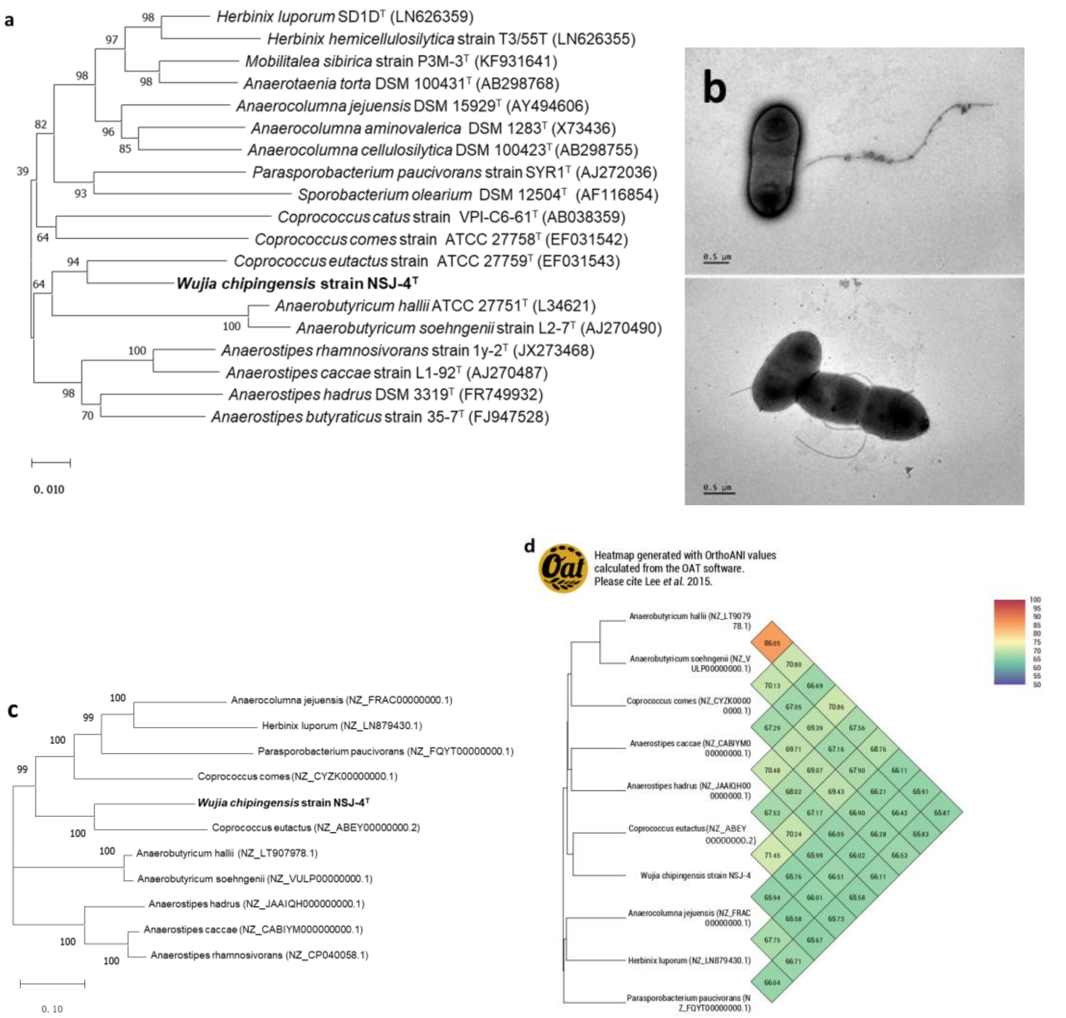

Figure SD-10. The Neighbour-joining phylogenetic tree based on 16S rRNA gene sequences (a) and the cell morphology (b) of strain NSJ-4^T. GenBank accession numbers are given in parentheses. Percentages of bootstrap support are shown at branch nodes. Bootstrap value was 1000. Bar: 0.01 substitutions per nucleotide position. The phylogenominc tree based on gemome-sequence-alignment (c) and OrthoANI heatmap (d) of strain NSJ-4^T and its neighbor species. Refseq ID of representative genomes are given in parentheses. Percentages of bootstrap support are shown at branch nodes. Bar: 0.1 substitutions per nucleotide position.

Taxon 11: *Simiaoa sunii* gen. nov. sp. nov.

Description of *Simiaoa* gen. nov. (Si.miao'a. N.L. fem. n. *Simiaoa*, named after the Chinese medical scientist Sun Simiao)

The closest phylogenetic neighbor of Taxon 11 as represented by strain NSJ-8^T, is *Kineothrix alysoides* (Figure SD-11a). *K. alysoides* and strain NSJ-8^T (16S rRNA sequence accession number in NMDC is NMDCN000010B) has 16S rRNA gene identity of 92.62 %, and their DNA G+C content is different by 3.21 %. The genome of strain NSJ-8^T was sequenced, and the NMDC accession number is NMDC60014011. and genome-based analysis on *K. alysoides* strain and type strain NSJ-8^T reveals that the dDDH and ANI values between *K. alysoides* KNHs209^T (NZ JPNB00000000) and strain NSJ-8^T are 21.50 % and 73.06 %, respectively, and the POCP value is 42.67 %, suggesting *K. alysoides* and strain NSJ-8^T are from different genera of the family *Lachnospiraceae*. According to the phylogenominc tree (Figure SD-11c), the ANIs and dDDH values of genomes between strain NSJ-8^T (NMDC accession number is NMDC60014011) and phylogenomically neighbored genomes were calculated, and the highest ANI and dDDH values are 72.6 and 29.1 %, respectively, between the query genome of *Simiaoa hominis* strain H15^T (NMDC60014012); and difference in % G+C between genomes of strain NSJ-8^T and *Simiaoa hominis* strain H15^T (NMDC60014012) is 0.57; OrthoANI heatmap (Figure SD-11d) shows the phylogenomic status of the corresponding neighbor species based on OrthoANI values. Cells of the strain NSJ-8^T are curved thin rods with flagella. The G+C content is around 45.91 mol %. The type species is *Simiaoa sunii*.

Description of *Simiaoa sunii* sp. nov. (sun'i.i. N.L. gen. n. *sunii*, named after the family name of the Chinese medical scientist Simiao Sun)

Cells are strictly anaerobic, straight rods with flagella (0.6-3.1 µm long by 0.4-0.7 µm wide, Figure SD-11b); cells appear in dividing pairs. Tiny, pinpoint, pale yellow, convex colonies appear on modified mGAM agar plate after 3 days of incubation. Growth occurs at 37°C and at pH range of 7.0-7.5. The organism uses arbutin, D-fructose, L-fucose, D-galactose, D-galacturonic acid, gentiobiose, α-D-glucose, glucose-6-phosphate, D-mannose, D-melibiose, 3-melthyl-D-glucose, palatinose, L-rhamnose, pyruvic acid and pyruvic acidmethyl ester as the carbon source for growth, and weakly metabolizes glyoxylic acid. The G+C content of the type strain NSJ-8^T is around 45.91 mol %. The type strain NSJ-8^T (=CGMCC 1.5284^T) was isolated from the faeces of a healthy adult.

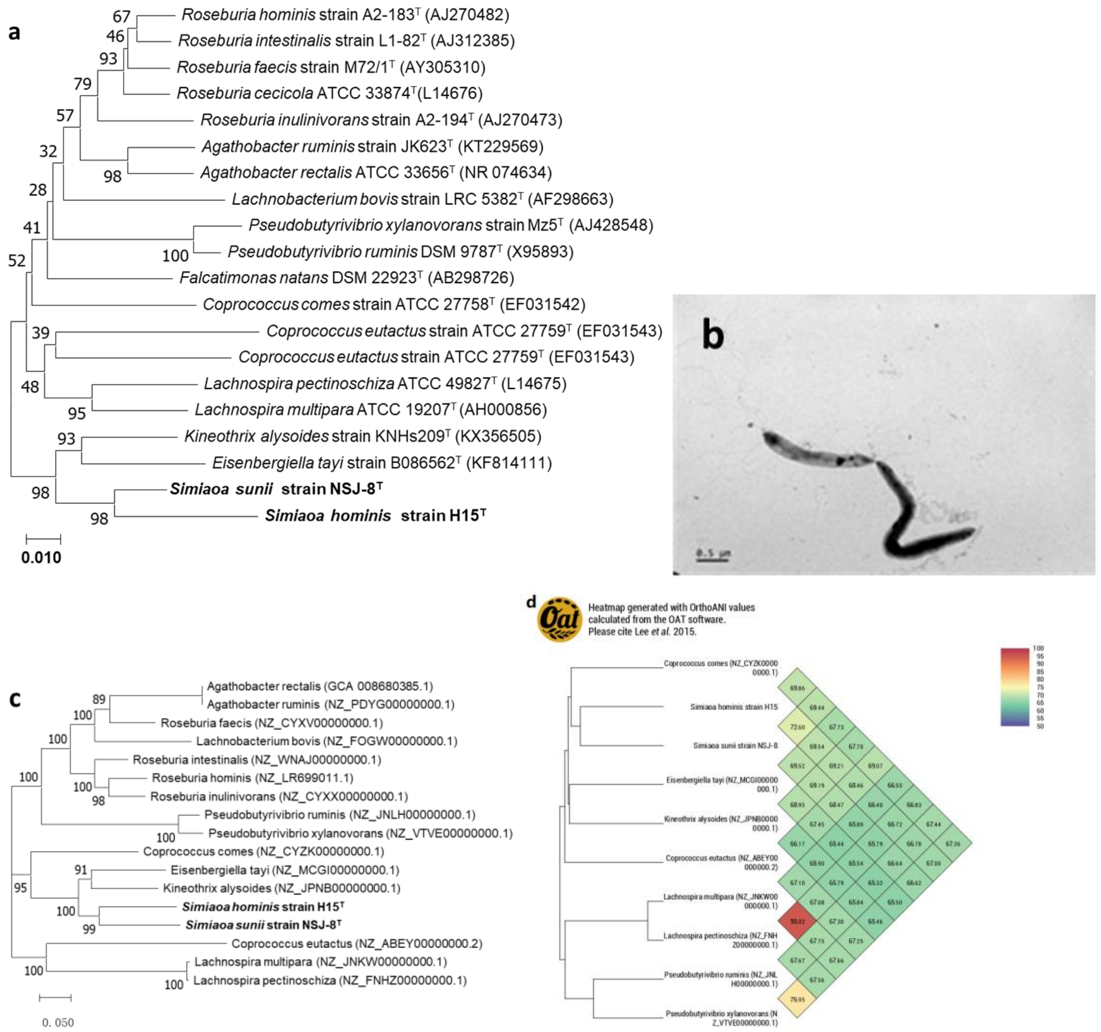

Figure SD-11. The Neighbour-joining phylogenetic tree based on 16S rRNA gene sequences (a) and the cell morphology (b) of strain NSJ-8^T. GenBank accession numbers are given in parentheses. Percentages of bootstrap support are shown at branch nodes. Bootstrap value was 1000. Bar: 0.01 substitutions per nucleotide position. The phylogenominc tree based on gemome-sequence-alignment (c) and OrthoANI heatmap (d) of strain NSJ-8^T and its neighbor species. Refseq ID of representative genomes are given in parentheses. Percentages of bootstrap support are shown at branch nodes. Bar: 0.050 substitutions per nucleotide position.

Taxon 12: *Simiaoa hominis* sp. nov.

.

Description of *Simiaoa hominis* sp. nov. (ho'mi.nis. L. gen. masc. n. *hominis*, of a human being, referring to the human gut habitat)

The validly published, closest phylogenetic neighbor of Taxon 12 as represented by strain H15^T, is *Kineothrix alysoides*, and Taxon 12 and *Simiaoa sunii* (Taxon 11) is phylogenetically closely clustered (Figure SD-11a). The genome of strain H15^T was sequenced, and the NMDC accession number is NMDC60014012. *K. alysoides* and strain H15^T (16S rRNA sequence accession number in NMDC is NMDCN000010C) has 16S rRNA gene identity of 93.16 %, and their DNA G+C content is different by 3.21 %; The genome of strain H15^T was sequenced, and genome-based analysis on *K. alysoides* strain and type strain H15^T reveals that the dDDH and ANI values between *K. alysoides* KNHs209^T (NZ JPNB00000000) and strain H15^T are 21.00 % and 68.74 %, respectively, and the POCP value is 38.71 %, suggesting *K. alysoides* and strain NSJ-8^T are from different genera of the family *Lachnospiraceae*, and *Simiaoa sunii* and strain NSJ-8^T (16S rRNA sequence accession number in NMDC is NMDCN000010B) that we proposed as a new genus shares 16S rRNA gene identity of 94.91 %. According to the phylogenominc tree (Figure SD-12c), the ANIs and dDDH values of genomes between strain H15^T (NMDC accession number is NMDC60014012) and phylogenomically neighbored genomes were calculated, and the highest ANI and dDDH values are 72.6 and 29.1 %, respectively, between the query genome of *Simiaoa sunii* strain NSJ-8^T (NMDC60014011); and difference in % G+C between genomes of strain H15^T and *Simiaoa sunii* strain NSJ-8^T (NMDC60014011) is 0.57; OrthoANI heatmap (Figure SD-12d) shows the phylogenomic status of the corresponding neighbor species based on OrthoANI values. Thus, Taxon 12 is considered as a new member of the genera we proposed as *Simiaoa* (Taxon 11). The G+C content is around 46.50 mol %. The type species is *Simiaoa hominis.*

Cells are strictly anaerobic, straight or slightly curved rods with flagella (0.8-2.6 µm long by 0.4-0.8 µm wide, Figure SD-12b); cells appear singly or in dividing pairs. Tiny, pinpoint, pale yellow, convex colonies appear on modified mGAM agar plate after 3 days of incubation. Growth occurs at temperature range of 30-37 °C and at pH range of

7.0-7.5. The organism uses arbutin, D-fructose, L-fucose, D-galactose, D-galacturonic acid, gentiobiose, α-D-glucose, glucose-6-phosphate, D-mannose, D-melibiose, 3-melthyl-D-glucose, palatinose, L-rhamnose, pyruvic acid and pyruvic acidmethyl ester as the carbon source for growth, and weakly metabolizes glyoxylic acid. The G+C content of the type strain H15^T is around 46.50 mol %. The type strain H15^T (=CGMCC 1.32863^T) was isolated from the faeces of a healthy adult.

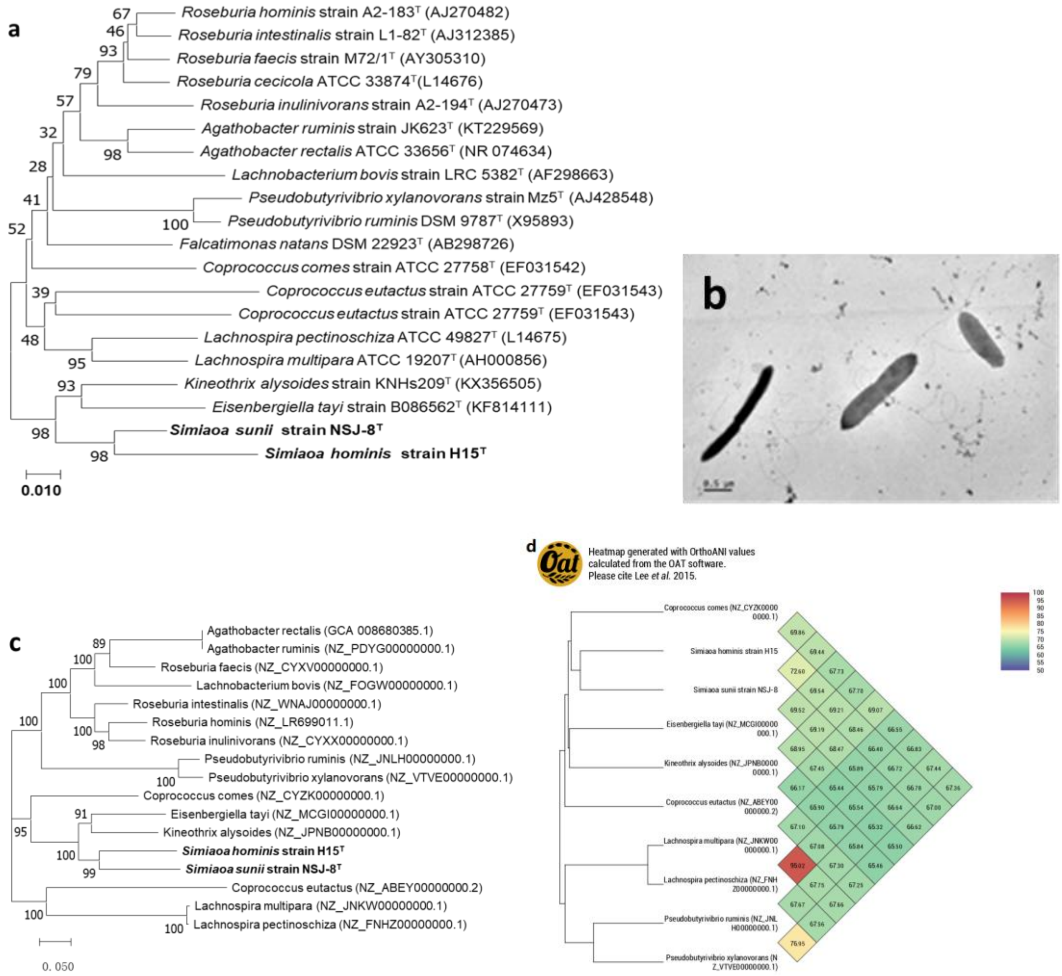

Figure SD-12. The Neighbour-joining phylogenetic tree based on 16S rRNA gene sequences (a) and the cell morphology (b) of strain H15^T. GenBank accession numbers are given in parentheses. Percentages of bootstrap support are shown at branch nodes. Bootstrap value was 1000. Bar: 0.010 substitutions per nucleotide position. The phylogenominc tree based on gemome-sequence-alignment (c) and OrthoANI heatmap (d) of strain H15^T and its neighbor species. Refseq ID of representative genomes are given in parentheses. Percentages of bootstrap support are shown at branch nodes. Bar: 0.050 substitutions per nucleotide position.

Taxon 13: *Jutongia hominis* sp. nov.

Description of *Jutongia hominis* sp. nov. (ho'mi.nis L. gen. masc. n. hominis, of a human being, referring to the human gut habitat)
The closest phylogenetic neighbor of Taxon 13 as represented by strain BX3^T, is *Eubacterium ventrios* with that *E. ventriosum* and strain BX3^T (16S rRNA sequence accession number in NMDC is NMDCN000010D) shares 16S rRNA gene identity of 91.7 %, and their DNA G+C content is different by 1.45 %. The genome of strain BX3^T was sequenced, and the NMDC accession number is NMDC60014013.
Genome-based analysis on *E. ventriosum* strain and type strain BX3^T reveals that the dDDH and ANI values between *E. ventriosum* ATCC 27560^T (NZ_AAVL00000000) and strain BX3^T are 71.02 % and 39.30 %, respectively, and the POCP value is 47.84 %, suggesting *E. ventriosum* and strain BX3^T are from different genera. According to the phylogenominc tree (Figure SD-13c), the ANIs and dDDH values of genomes between strain BX3^T (NMDC accession number is NMDC60014013) and phylogenomically neighbored genomes were calculated, and the highest ANI and dDDH values are 69.7547 and 23.1 %, respectively, between the query genome of *Jutongia huaianensis* strain NSJ-37^T (NMDC60014014); and difference in % G+C between genomes of strain BX3^T and *Jutongia huaianensis* strain NSJ-37^T (NMDC60014014) is 5.49; OrthoANI heatmap (Figure SD-13d) shows the phylogenomic status of the corresponding neighbor species based on OrthoANI values. And strain BX3^T and strain NSJ-37^T (16S rRNA sequence accession number in NMDC is NMDCN000010E) we proposed as a novel genus (*Jutongia huaianensis* gen. nov. sp. nov.) shares 16S rRNA gene identity of 95.73 %, and their DNA G+C content is different by 1.40 %, ANI values is differented by 71.59 %, phylogenetic tree based on 16S rRNA gene was generated as below (Figure SD-13a). Based on these results, we concluded that the strain BX3^T represents of new species of the genus *Jutongia*, and the name *Jutongia hominis* sp. nov. is proposed.
Cells are strictly anaerobic, club shaped rods with single blunt end (1.1-2.9 µm long by 0.5-1.0 µm wide, Figure SD-13b); cells appear singly. The G+C content of the type strain BX3^T is 38.6 mol %. The type strain BX3^T (=CGMCC 1.32876^T) was isolated from the faeces of a healthy adult.

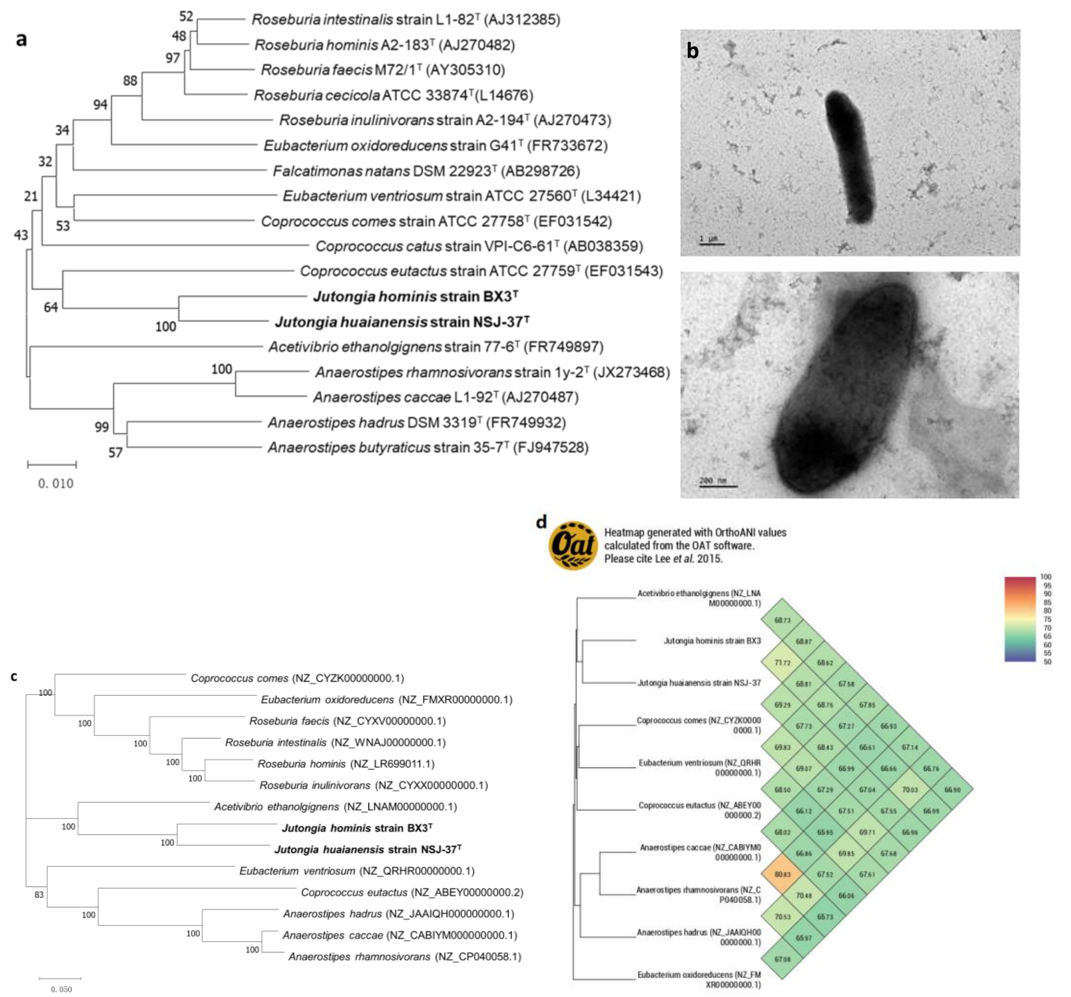

Figure SD-13. The Neighbour-joining phylogenetic tree based on 16S rRNA gene sequences (a) and the cell morphology (b) of strain BX3^T. GenBank accession numbers are given in parentheses. Percentages of bootstrap support are shown at branch nodes. Bootstrap value was 1000. Bar: 0.01 substitutions per nucleotide position. The phylogenomic tree based on gemome-sequence-alignment (c) and OrthoANI heatmap (d) of strain BX3^T and its neighbor species. Refseq ID of representative genomes are given in parentheses. Percentages of bootstrap support are shown at branch nodes. Bar: 0.050 substitutions per nucleotide position.

Taxon 14: *Jutongia huaianensis* gen. nov. sp. nov.

Description of *Jutongia* gen. nov. (Ju.tong'ia, L. adj. fem. *Jutongia*, in honor of the Chinese medical scientist Jutong Wu)

The closest phylogenetic neighbor of Taxon 14 as represented by strain NSJ-37^T, is *Roseburia inulinivorans* (Figure SD-14a). *R. inulinivorans* and strain NSJ-37^T (16S rRNA sequence accession number in NMDC is NMDCN000010E) has 16S rRNA gene identity of 92 %, and their DNA G+C content is different by 10.21 %. The strain NSJ-37^T can not cluster with any species of genus *Roseburia* or the members of the *Lachnospiraceae* family. The genome of strain NSJ-37^T was sequenced, and the NMDC accession number is NMDC60014014. Genome-based analysis on *R. inulinivorans* strain and type strain NSJ-37^T reveals that the dDDH and ANI values between *R. inulinivorans* A2-194^T (NZ ACFY00000000) and strain NSJ-37^T are 28.90 % and 78.9 2 %, respectively, and the POCP value is 35.98 %, suggesting *R. inulinivorans* and strain NSJ-37^T are from different genera of the family *Lachnospiraceae*. According to the phylogenominc tree (Figure SD-14c), the ANIs and dDDH values of genomes between strain NSJ-37^T (NMDC accession number is NMDC60014014) and phylogenomically neighbored genomes were calculated, and the highest ANI and dDDH values are 69.98 and 23.1 %, respectively, between the query genome of *Jutongia hominis* strain BX3^T (NMDC60014013); and difference in % G+C between genomes of strain NSJ-37^T and *Jutongia hominis* strain BX3^T (NMDC60014013) is 5.49; OrthoANI heatmap (Figure SD-14d) shows the phylogenomic status of the corresponding neighbor species based on OrthoANI values. Cells of the strain NSJ-37^T are straight rods with flagella. The G+C content is around 51.41 mol %. The type species is *Jutongia huaianensis*.

Description of *Jutongia huaianensis* sp. nov.

***Jutongia huaianensis* sp. nov.** (huai.an'en.sis. N.L. fem. adj. *huaianensis*, referring to huai'an county of China, the birthplace of the Chinese medical scientist Jutong Wu)

Cells are strictly anaerobic, slightly curved or straight rods with flagella(1.8-2.8 µm long by 0.7–0.9 µm wide, Figure SD-14b) with flagellum. Cells appear singly or in (dividing) pairs. Circular, cream-color to whitish, convex, entire colonies appear on modified mGAM agar plate after 3 days of incubation. Growth occurs at 37°C and at pH range of 7.0-7.5. The organism uses N-acetyl-D galactosamine, glucose-6-phosphate and pyruvic acid methyl ester as the carbon source for growth, and weakly metabolizes D-melibiose and D-lactic acid methyl ester. The G+C content of the type strain NSJ-37^T is around 51.41 mol %. The type strain NSJ-37^T (=CGMCC 1.32810^T) was isolated from the faeces of a healthy adult.

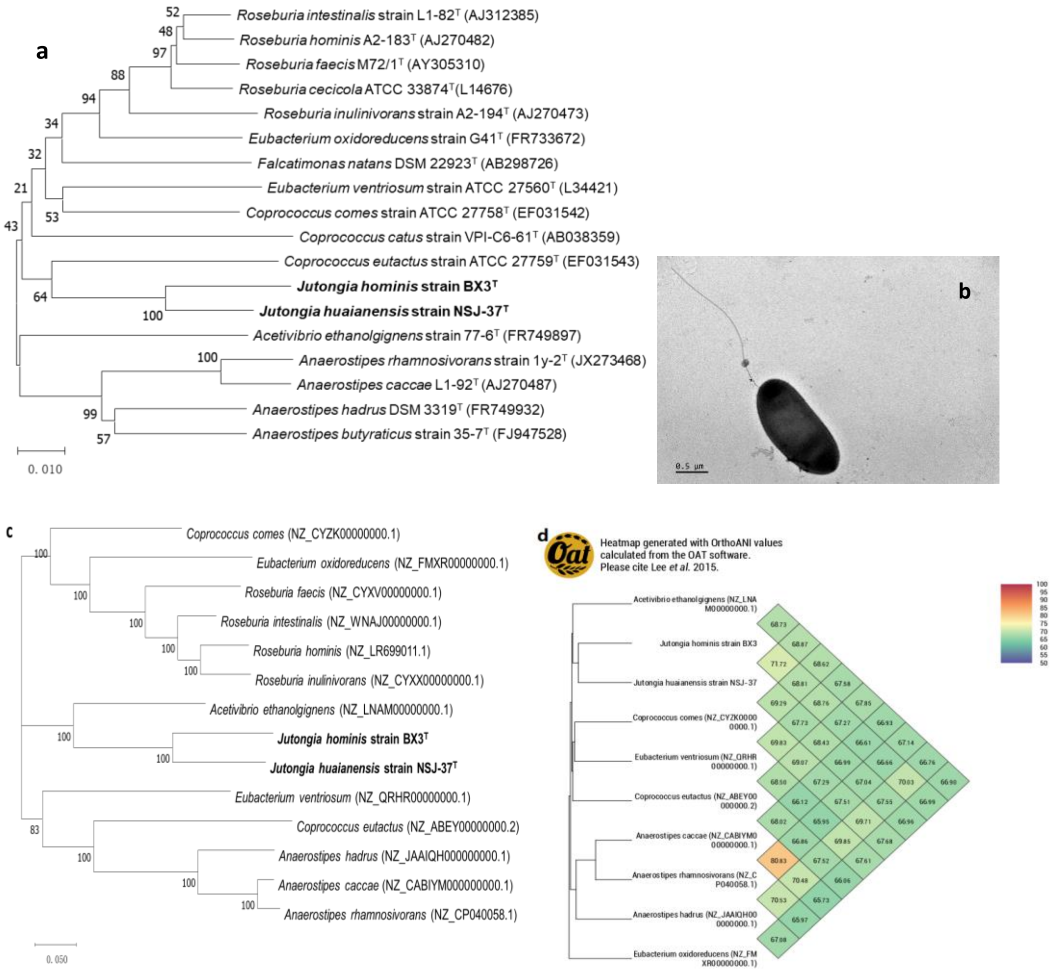

Figure SD-14. The Neighbour-joining phylogenetic tree based on 16S rRNA gene sequences (a) and the cell morphology (b) of strain NSJ-37^{T}. GenBank accession numbers are given in parentheses. Percentages of bootstrap support are shown at branch nodes. Bootstrap value was 1000. Bar: 0.01 substitutions per nucleotide position. The phylogenominc tree based on gemome-sequence-alignment (c) and OrthoANI heatmap (d) of strain NSJ-37^{T} and its neighbor species. Refseq ID of representative genomes are given in parentheses. Percentages of bootstrap support are shown at branch nodes. Bar: 0.05 substitutions per nucleotide position.

Taxon 15: *Qiania dongpingensis* gen. nov. sp. nov.

Description of *Qiania* gen. nov. (Qian'i.a. N.L. fem. n. *Qiania*, named after the Chinese medical scientist Yi Qian)

The closest phylogenetic neighbor of Taxon 15 as represented by strain NSJ-38^T, is *Enterocloster aldensis* (Figure SD-78a). *E. aldensis* and strain NSJ-38^T (16S rRNA sequence accession number in NMDC is NMDCN000010F) has 16S rRNA gene identity of 93 %, and their DNA G+C content is different by 0.48 %. The genome of strain NSJ-38^T was sequenced, and the NMDC accession number is NMDC60014015. Genome-based analysis on *E. aldensis* strain and type strain NSJ-38^T reveals that the dDDH and ANI values between *E. aldensis* RMA 9741^T (NZ QSGP00000000) and strain NSJ-38^T are 21.60 % and 74.12 %, respectively, and the POCP value is 38.48 %, suggesting *E. aldensis* and strain NSJ-38^T are from different genera of the family *Lachnospiraceae*. According to the phylogenominc tree (Figure SD-15c), the ANIs and dDDH values of genomes between strain NSJ-38^T (NMDC accession number is NMDC60014015) and phylogenomically neighbored genomes were calculated, and the highest ANI and dDDH values are 68.53 and 35.2 %, respectively, between the query genome of *Fusicatenibacter saccharivorans* (NZ_CYYV00000000.1); and difference in % G+C between genomes of strain NSJ-38^T and *Fusicatenibacter saccharivorans* (NZ_CYYV00000000.1) is 2.11; OrthoANI heatmap (Figure SD-15d) shows the phylogenomic status of the corresponding neighbor species based on OrthoANI values. Cells of the strain NSJ-38^T are oval to rod shaped with tapered ends. The G+C content is around 49.22 mol %. The type species is *Qiania dongpingensis.*

Description of *Qiania dongpingensis* sp. nov.

***Qiania dongpingensis* sp. nov.** (dong.ping'en'sis. N.L. fem. adj. *dongpingensis*, referring to Dongping county of China, the birthplace of Yi Qian)

Cells are strictly anaerobic, oval to rod shaped with tapered ends (2.2–7.6 µm long by 0.9–1.1 µm wide, Figure SD-15b); cells appear singly, in dividing pairs or in chains. Irregular, cream-color, smooth, dry, translucent colonies appear on modified mGAM agar plate after 3 days of incubation. Growth occurs at 37°C and at pH range of 7.0-7.5. The organism uses D-mannose, D-fructose, L-fucose, D-galactose, D-galacturonic acid, gentiobiose, D-glucosaminic acid, α-D-glucose, glucose-6-phosphate, lactulose, D-melibiose, 3-melthyl-D-glucose, palatinose and L-rhamnose as the carbon source for growth, and weakly metabolizes β-cyclodextrin, i-erythritol, D-glucosaminic acid, m-Inositol, L-asparagine and L-glutamine. The G+C content of the type strain NSJ-38^T is around 49.22 mol %. The type strain NSJ-38^T (=CGMCC 1.32811^T) was isolated from the faeces of a healthy adult.

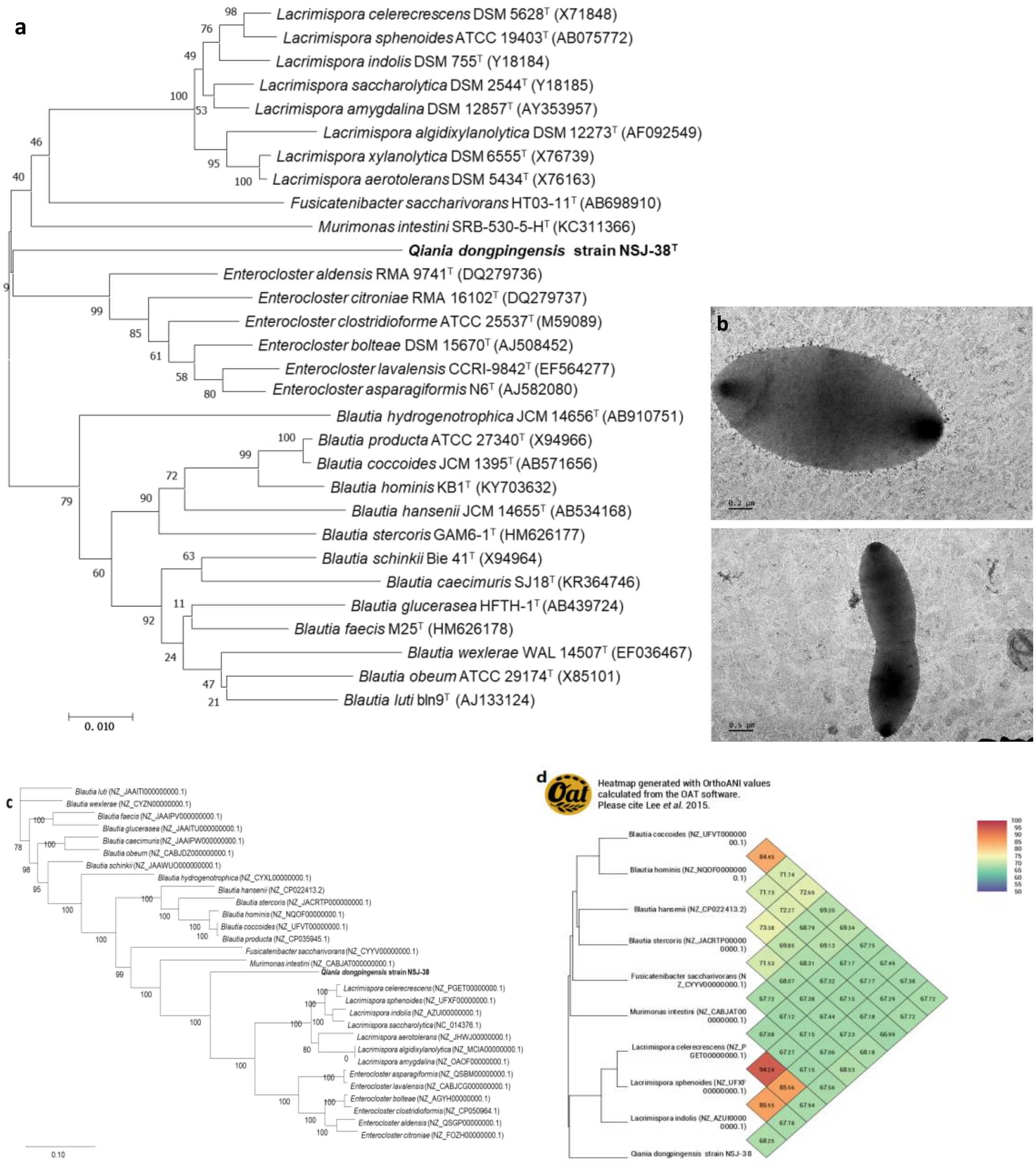

Figure SD-15. The Neighbour-joining phylogenetic tree based on 16S rRNA gene sequences (a) and the cell morphology (b) of strain NSJ-38^T. GenBank accession numbers are given in parentheses. Percentages of bootstrap support are shown at branch nodes. Bootstrap value was 1000. Bar: 0.01 substitutions per nucleotide position. The phylogenominc tree based on gemome-sequence-alignment (c) and OrthoANI heatmap (d) of strain NSJ-38^T and its neighbor species. Refseq ID of representative genomes are given in parentheses. Percentages of bootstrap support are shown at branch nodes. Bar: 0.10 substitutions per nucleotide position.

Taxon 16: *Zhenhengia yiwuensis* gen. nov. sp. nov.

Description of *Zhenhengia* gen. nov. (Zhen.heng'i.a. N.L. fem. n. *Zhenhengia*, named after the Chinese medical scientist Zhenheng Zhu)

The closest phylogenetic neighbor of Taxon 16 as represented by strain NSJ-12^T, is *Cellulosilyticum lentocellum* (Figure SD-16a). *C. lentocellum* and strain NSJ-12^T (16S rRNA sequence accession number in NMDC is NMDCN000010G) has 16S rRNA gene identity of 92 %, and their DNA G+C content is different by 18.89 %. The genome of strain NSJ-12^T was sequenced, and the NMDC accession number is NMDC60014016. Genome-based analysis on *C. lentocellum* strain and type strain NSJ-12^T reveals that the dDDH and ANI values between *C. lentocellum* DSM 5427^T (NC 015275) and strain NSJ-12^T are 22.0 % and 81.80 %, respectively, and the POCP value is 42.70 %, suggesting *C. lentocellum* and strain NSJ-12^T are from different genera of the family *Lachnospiraceae*. According to the phylogenominc tree (Figure SD-16c), the ANIs and dDDH values of genomes between strain NSJ-12^T (NMDC accession number is NMDC60014016) and phylogenomically neighbored genomes were calculated, and the highest ANI and dDDH values are 69.81 and 19.7 %, respectively, between the query genome of *Cellulosilyticum ruminicola* (NZ_BBCG00000000.1); and difference in % G+C between genomes of strain NSJ-12^T and Cellulosilyticum ruminicola (NZ_BBCG00000000.1) is 1.89; OrthoANI heatmap (Figure SD-16d) shows the phylogenomic status of the corresponding neighbor species based on OrthoANI values. Cells of the strain NSJ-12^T are rod shaped. The G+C content is around 53.19 mol %. The type species is *Zhenhengia yiwuensis.*

Description of *Zhenhengia yiwuensis* sp. nov. (yi.wu.en'sis. N.L. fem. adj. *yiwuensis*, referring to Yiwu city of China, where Zhenheng Zhu was born)

Cells are strictly anaerobic, straight rods (2.3-4.0 µm long by 0.8-1.2 µm wide, Figure SD-16b); cells appear singly or in dividing pairs. Cream-colored, tiny, pinpoint colonies appear on modified mGAM agar plate after 3 days of incubation. Growth occurs at 37°C and at pH range of 7.0-7.5. The organism uses D-fructose, L-fucose, D-galactose D-galacturonic acid, gentiobiose, 3-melthyl-D-glucose, palatinose, L-rhamnose, pyruvic acid and pyruvic acid as the carbon source for growth, and weakly metabolizes arbutin, glucose-6-phosphate, salicin and a-ketovaleric acid. The G+C content of the type strain NSJ-12^T is around 53.19 mol %. The type strain NSJ-12^T (=CGMCC 1.32465^T=KCTC 15954^T) was isolated from the faeces of a healthy adult.

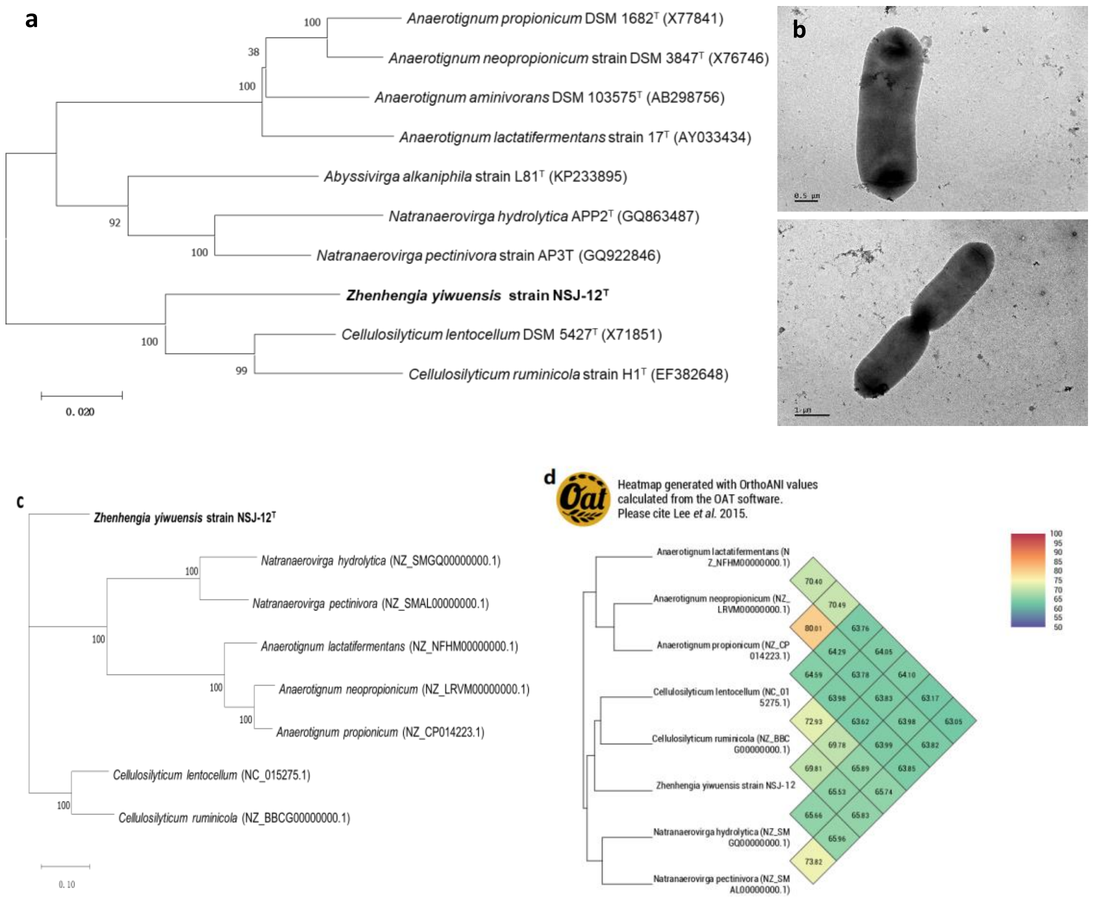

Figure SD-16. The Neighbour-joining phylogenetic tree based on 16S rRNA gene sequences (a) and the cell morphology (b) of strain NSJ-12^T. GenBank accession numbers are given in parentheses. Percentages of bootstrap support are shown at branch nodes. Bootstrap value was 1000. Bar: 0.02 substitutions per nucleotide position. The phylogenominc tree based on gemome-sequence-alignment (c) and OrthoANI heatmap (d) of strain NSJ-12^T and its neighbor species. Refseq ID of representative genomes are given in parentheses. Percentages of bootstrap support are shown at branch nodes. Bar: 0.10 substitutions per nucleotide position.

Taxon 17: *Jingyaoa shaoxingensis* gen. nov. sp. nov.

Description of *Jingyaoa* gen. nov. (Jing.yao'a. N.L. fem. n. *Jingyaoa*, named after the Chinese medical scientist Jingyao Zhang.)
The closest phylogenetic neighbor of Taxon 17 as represented by strain NSJ-46^T, is *Lacrimispora amygdalina* by *L. amygdalina* and strain NSJ-46^T shares 16S rRNA gene identity of 93.4 %, and their DNA G+C content is different by 4.42 %. The strain NSJ-46^T (16S rRNA sequence accession number in NMDC is NMDCN000010H) can not cluster with any species of *Lacrimispora*. The genome of strain NSJ-46^T was sequenced, and the NMDC accession number is NMDC60014017, and genome-based analysis on *L. amygdalina* strain and type strain NSJ-46^T reveals that the dDDH and ANI values between *L. amygdalina* DSM 12857^T (NZ OAOF00000000) and strain NSJ-46^T are 22.80 % and 74.08 %, respectively, and the POCP value is 35.49 %, and phylogenetic tree based on 16S rRNA sequence shows that strain NSJ-46^T dooes not cluster with any other members of the genus *Lacrimispora*(Figure SD-17a), suggesting *L. amygdalina* and strain NSJ-46^T are from different genera. According to the phylogenominc tree (Figure SD-17c), the ANIs and dDDH values of genomes between strain NSJ-46^T (NMDC accession number is NMDC60014017) and phylogenomically neighbored genomes were calculated, and the highest ANI and dDDH values are 71.24 and 31.2 %, respectively, between the query genome of *Fusicatenibacter saccharivorans* (NZ_CYYV00000000.1); and difference in % G+C between genomes of strain NSJ-46^T and *Fusicatenibacter saccharivorans* (NZ_CYYV00000000.1) is 4.4; OrthoANI heatmap (Figure SD-17d) shows the phylogenomic status of the corresponding neighbor species based on OrthoANI values. Cells of the strain NSJ-46^T are spherical or oval or short rod shaped. The G+C content is around 54.52 mol %. The type species is *Jingyaoa shaoxingensis.*

Description of *Jingyaoa shaoxingensis* sp. nov. (shao.xing'en.sis. N.L. fem. adj. *shaoxingensis*, referring to Shaoxing city of China, where Jingyao Zhang was born)
Cells are strictly anaerobic, spherical or oval or short rod shaped (1.8-2.6 μm long by 1.2-2.1 μm wide, Figure SD-17b); cells appear singly or in dividing pairs. Growth occurs at 37°C and at pH range of 7.0-7.5. The organism uses N-acetyl-D galactosamine, N-acetyl-D glucosamine, N-acetyl-β -D mannosamine, adonitol, amygdalin, D-arabitol, arbutin, D-cellobiose, dextrin,i-erythritol, D-fructose, L-fucose, D-galactose, D-galacturonic acid, gentiobiose, D-gluconic acid, D-glucosaminic acid, α-D-glucose, glucose-1-phosphate, glucose-6-phosphate, glycerol, D,L-α-glycerol phosphate, m-inositol, α-D-lactose, lactulose, maltose, maltotriose, D-mannose, D-melibiose, 3-melthyl-D-glucose, α-methyl-D-galactoside, ß -methyl-D-galactoside, α-methyl-D glucoside,ß -methyl-D-glucoside, D-raffinose, L-rhamnose, D-sorbitol, stachyose, turanose, acetic acid, formic acid, fumaric acid, glyoxylic acid, α-hydroxybutyric acid, ß -hydroxybutyric acid, D,L-lactic acid, L-lactic acid, D-lactic acidmethyl ester,D-malic acid, L- malic acid, propionic acid, pyruvic acidmethyl ester, D-saccharic acid, succinamic acid, succinic acid, succinic acid mono-methyl ester, m-tartaric acid, urocanic acid, L-alaninamide, L-alanine, L-alanyl-L-glutamine,

L-alanyl-L-histidine, L-alanyl-L-threonine, L-asparagine, L-glutamic acid, glycyl-L-aspartic acid, glycyl-L-methionine, glycyl-L-proline, L-methionine, L-phenylalanine, L-serine, L-threonine, L-valine plus L-aspartic acid, 2’-deoxyadenosine, inosine, thymidine, uridine and uridine-5’-monophosphate as the carbon source for growth, and weakly metabolizes sucrose. The G+C content of the type strain NSJ-46^T is around 54.52 mol %. The type strain NSJ-46^T (=CGMCC 1.32819^T) was isolated from the faeces of a healthy adult.

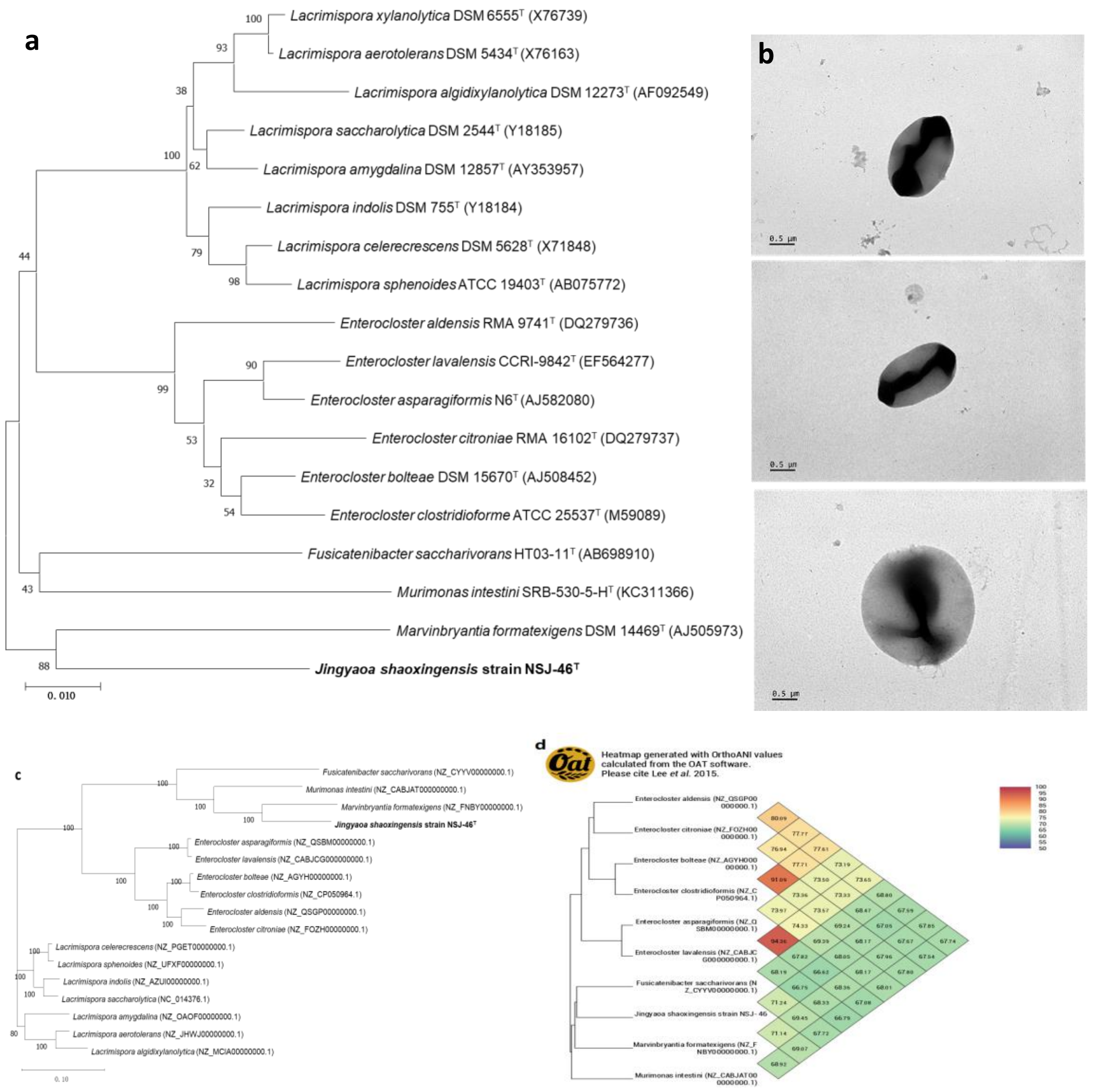

Figure SD-17. The Neighbour-joining phylogenetic tree based on 16S rRNA gene sequences (a) and the cell morphology (b) of strain NSJ-46^T. GenBank accession numbers are given in parentheses. Percentages of bootstrap support are shown at branch nodes. Bootstrap value was 1000. Bar: 0.01 substitutions per nucleotide position. The phylogenominc tree based on gemome-sequence-alignment (c) and OrthoANI heatmap (d) of strain NSJ-46^T and its neighbor species. Refseq ID of representative genomes are given in parentheses. Percentages of bootstrap support are shown at branch nodes. Bar: 0.10 substitutions per nucleotide position.

Taxon 18: *Wansuia hejianensis* gen. nov. sp. nov.

Description of *Wansuia* gen. nov. (Wan.su'i.a. N.L. adj. fem. *Wansuia*, in honor of the Chinese medical scientist Wansu Liu**)**

The closest phylogenetic neighbor of Taxon 18 as represented by strain NSJ-29^T, is *Blautia hominis* (Figure SD-18a). *B. hominis* and strain NSJ-29^T (16S rRNA sequence accession number in NMDC is NMDCN000010I) has 16S rRNA gene identity of 93.7 %, and their DNA G+C content is different by 13.23 %. The type strain NSJ-29^T can not cluster with any species of *Blautia*. The genome of strain NSJ-29^T was sequenced, and the NMDC accession number is NMDC60014018. genome-based analysis on *B. hominis* strain and type strain NSJ-29^T reveals that the dDDH and ANI values between *B. hominis* KB1^T (NZ NQOF00000000) and strain NSJ-29^T are 44.10 % and 70.75 %, respectively, and the POCP value is 41.77 %, suggesting *B. hominis* and strain NSJ-29^T are from different genera of the family *Lachnospiraceae*. According to the phylogenominc tree (Figure SD-18c), the ANIs and dDDH values of genomes between strain NSJ-29^T (NMDC accession number is NMDC60014018) and phylogenomically neighbored genomes were calculated, and the highest ANI and dDDH values are 70.22 and 23.6 %, respectively, between the query genome of *Fusicatenibacter saccharivorans* (NZ_CYYV00000000.1); and difference in % G+C between genomes of strain NSJ-29^T and *Fusicatenibacter saccharivorans* (NZ_CYYV00000000.1) is 2.23; OrthoANI heatmap (Figure SD-18d) shows the phylogenomic status of the corresponding neighbor species based on OrthoANI values. Cells of strain NSJ-29^T are oval to rod in shape with spiky ends. The G+C content is around 59.53 mol %. The type species is *Wansuia hejianensis.*

Description of *Wansuia hejianensis* sp. nov. (he.jian.en'sis, N.L. fem. adj. *hejianesis*, referring to Hejian county of China, the birthplace of the Chinese medical scientist Wansu Liu)

Cells are strictly anaerobic, oval to rod in shape with spiky ends (1.2-2.0 μm long, 0.8-1.0 μm wide, Figure SD-18b); cells appear singly or in dividing pairs. White to light yellow, circular, translucent, slightly peaked colonies appear on modified mGAM agar plate after 6 days of incubation. Growth occurs at 37°C and at pH of 7.0-7.5. The organism uses N-acetyl-D glucosamine, dextrin, i-erythritol, D-fructose, L-fucose, D-galactose, D-galacturonic acid, gentiobiose, α-D-glucose, glycerol, glucose-1-phosphate, glucose-6-phosphate, D-mannitol, D-mannose, D-melibiose, 3-melthyl-D-glucose, palatinose, L-rhamnose, D-sorbitol, formic acid, fumaric acid, glyoxylic acid, α-ketobutyric acid, α-ketovaleric acid, D-lactic acidmethyl ester, L-malic acid, pyruvic acid, pyruvic acidmethyl ester, L-alanine, L-alanyl-L-glutamine, L-alanyl-L-threonine, L-glutamic acid, L-serine, L-threonine, L-valine plus L-aspartic acid, 2'-deoxyadenosine, inosine, thymidine and uridine as the carbon source for growth, and weakly metabolizes N-acetyl-β-D mannosamine, D-arabitol, D-cellobiose, D-gluconic acid, lactulose, maltotriose, turanose, acetic acid, propionic acid, m-tartaric acid, L-glutamine, L-valine and uridine-5'-monophosphate. The G+C content of the type strain NSJ-29^T is around 59.53 mol %. The type strain NSJ-29^T (=CGMCC 1.32802^T =KCTC 25078^T) was isolated from the faeces of a healthy adult.

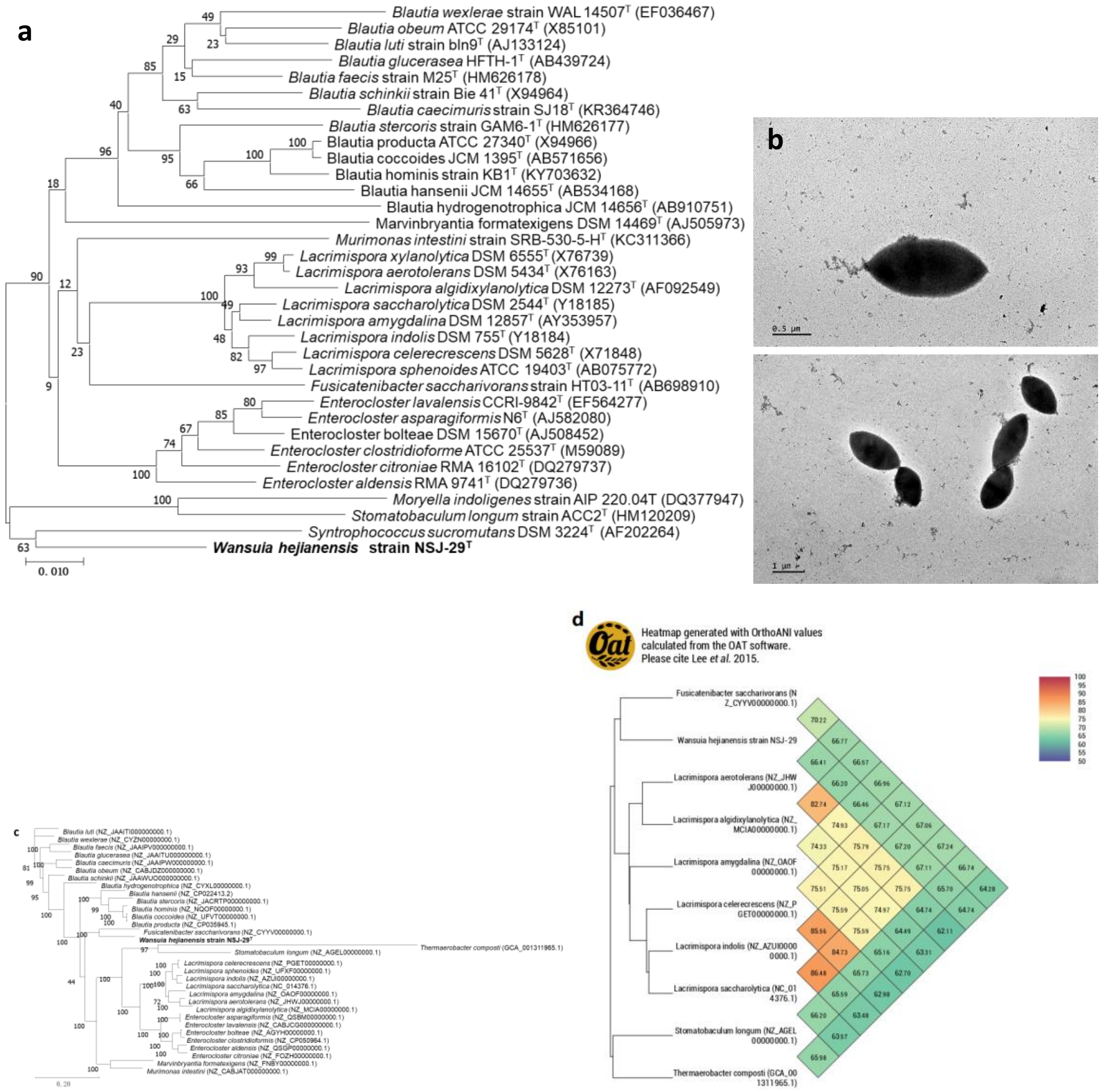

Figure SD-18. The Neighbour-joining phylogenetic tree based on 16S rRNA gene sequences (a) and the cell morphology (b) of strain NSJ-29^T. GenBank accession numbers are given in parentheses. Percentages of bootstrap support are shown at branch nodes. Bootstrap value was 1000. Bar: 0.01 substitutions per nucleotide position. The phylogenominc tree based on gemome-sequence-alignment (c) and OrthoANI heatmap (d) of strain NSJ-29^T and its neighbor species. Refseq ID of representative genomes are given in parentheses. Percentages of bootstrap support are shown at branch nodes. Bar: 0.20 substitutions per nucleotide position.

Taxon 19: *Zhenpiania hominis* gen. nov. sp. nov.

Description of *Zhenpiania* gen. nov. (Zhen.pian'i.a. N.L. fem. n. *Zhenpiania*, named after the Chinese medical scientist Zhenpian Li)

The closest phylogenetic neighbor of Taxon 19 as represented by strain BX12^T, is *Aminipila butyrica* (Figure SD-19a). *A. butyrica* and strain BX12^T (16S rRNA sequence accession number in NMDC is NMDCN000010J) has 16S rRNA gene identity of 92.5 %, and their DNA G+C content is different by 2.6 %. The genome of strain BX12^T was sequenced, and the NMDC accession number is NMDC60014019. Genome-based analysis on *A. butyrica* strain and type strain BX12^T reveals that the dDDH and ANI values between *A. butyrica* FH042^T (NZ CP048649) and strain BX12^T are 67.93 % and 25.00 %, respectively, and the POCP value is 39.40 %, suggesting *A. butyrica* and strain BX12^T are from different genera of the order *Eubacteriales*. According to the phylogenominc tree (Figure SD-19c), the ANIs and dDDH values of genomes between strain BX12^T (NMDC accession number is NMDC60014019) and phylogenomically neighbored genomes were calculated, and the highest ANI and dDDH values are 68.86 and 24.1 %, respectively, between the query genome of *Lentihominibacter faecis* strain BX16 (NMDC60014020); and difference in % G+C between genomes of strain BX12^T and *Lentihominibacter faecis* strain BX16^T (NMDC60014020) is 0.61; OrthoANI heatmap (Figure SD-19d) shows the phylogenomic status of the corresponding neighbor species based on OrthoANI values. Cells of strain BX12^T are straight or slightly curved rods. The G+C content is around 47.50 mol %. The type species is *Zhenpiania hominis.*

Description of *Zhenpiania hominis* sp. nov. (ho'mi.nis. L. gen. masc. n. *hominis*, of a human being, referring to the human gut habitat)

Cells are strictly anaerobic rods (1.2-2.1 µm long by 0.7-1.0 µm wide, Figure SD-19b); cells appear singly or in dividing pairs. The G+C content of the type strain BX12^T is around 47.50 mol %. The type strain BX12^T (=CGMCC 1.32877^T) was isolated from the faeces of a healthy adult.

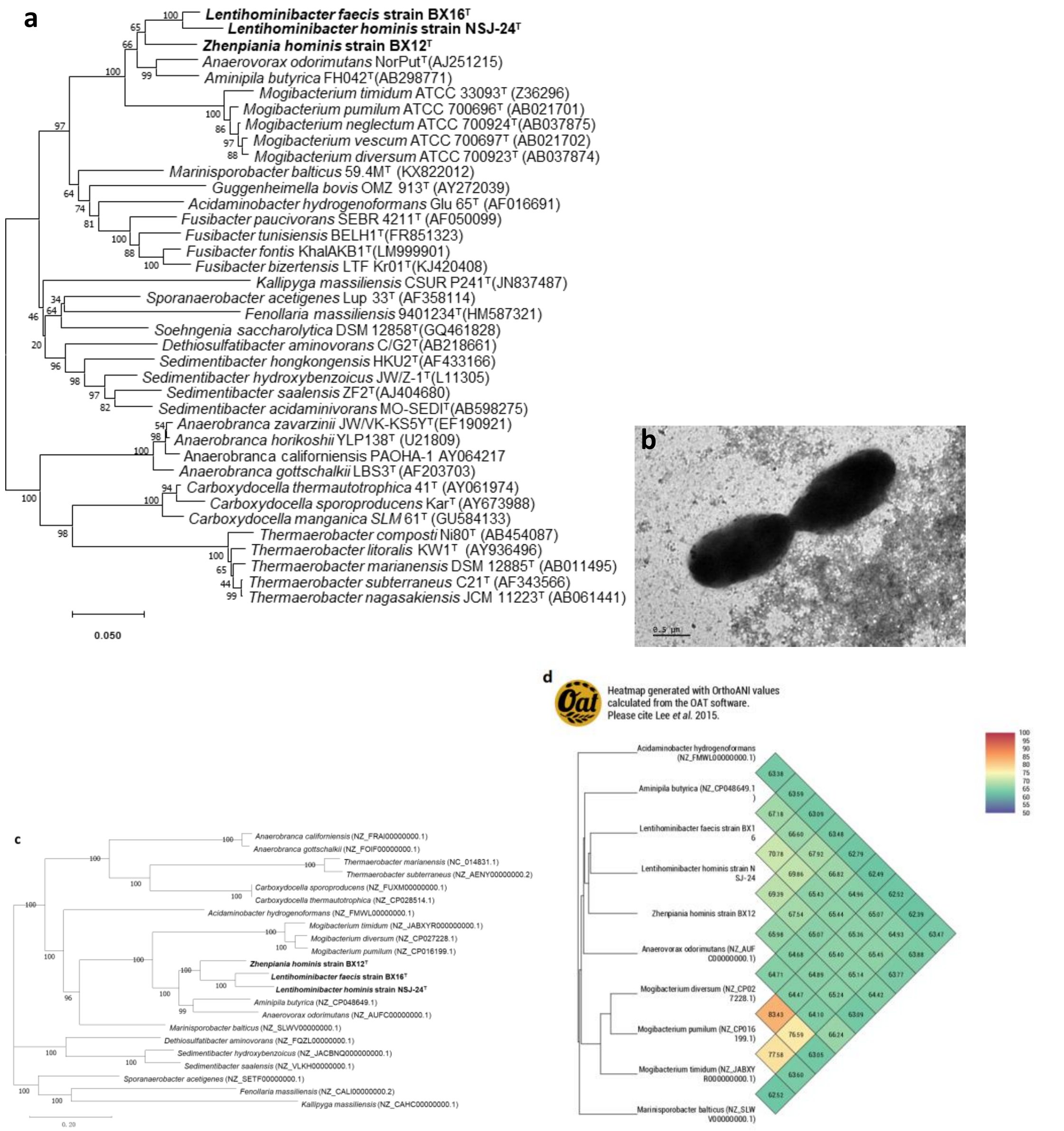

Figure SD-19. The Neighbour-joining phylogenetic tree based on 16S rRNA gene sequences (a) and the cell morphology (b) of strain BX12^{T}. GenBank accession numbers are given in parentheses. Percentages of bootstrap support are shown at branch nodes. Bootstrap value was 1000. Bar: 0.05 substitutions per nucleotide position. The phylogenominc tree based on gemome-sequence-alignment (c) and OrthoANI heatmap (d) of strain BX12^{T} and its neighbor species. Refseq ID of representative genomes are given in parentheses. Percentages of bootstrap support are shown at branch nodes. Bar: 0.2 substitutions per nucleotide position.

Taxon 20: *Lentihominibacter faecis* sp. nov.

Description of *Lentihominibacter faecis* sp. nov. (L. gen. fem. n. *faecis*, of faeces, from which the organism was isolated)

The closest phylogenetic neighbor of Taxon 20 as represented by strain BX16^T, is *Anaerovorax odorimutans* (Figure SD-20a). *A. odorimutans* and strain BX16^T (16S rRNA sequence accession number in NMDC is NMDCN000010K) has 16S rRNA gene identity of 92.8 %, and their DNA G+C content is different by 16.10 %. The genome of strain BX16^T was sequenced, and the NMDC accession number is NMDC60014020. Genome-based analysis on *A. odorimutans* strain and type strain BX16^T reveals that the dDDH and ANI values between *A. odorimutans* NorPut^T (NZ AUFC00000000) and strain BX16^T are 64.83 % and 21.90 %, respectively, and the POCP value is 34.64 %, suggesting *A. odorimutans* and strain BX16^T are from different genera of the order *Eubacteriales*. According to the phylogenominc tree (Figure SD-20c), the ANIs and dDDH values of genomes between strain BX16^T (NMDC accession number is NMDC60014020) and phylogenomically neighbored genomes were calculated, and the highest ANI and dDDH values are 68.02 and 24.1 %, respectively, between the query genome of *Zhenpiania hominis* strain BX12^T (NMDC60014019); and difference in % G+C between genomes of strain BX16^T and *Zhenpiania hominis* strain BX12^T (NMDC60014019) is 0.61; OrthoANI heatmap (Figure SD-20d) shows the phylogenomic status of the corresponding neighbor species based on OrthoANI values. Strain BX16^T and *Lentihominibacter hominis* strain NSJ-24^T shares 16S rRNA gene identity of 96.4 %, thus we conclude the strain is a new species of the genus *Lentihominibacter*.

Cells of strain BX16^T are straight or slightly curved rods. The G+C content is around 47.60 mol %. Cells are strictly anaerobic rods (1.6-2.4 µm long by 0.6-0.8 µm wide, Figure SD-20b); cells appear singly. The G+C content of the type strain BX16^T is around 47.60 mol %. The type strain BX16^T (=CGMCC 1.32878^T) was isolated from the faeces of a healthy adult.

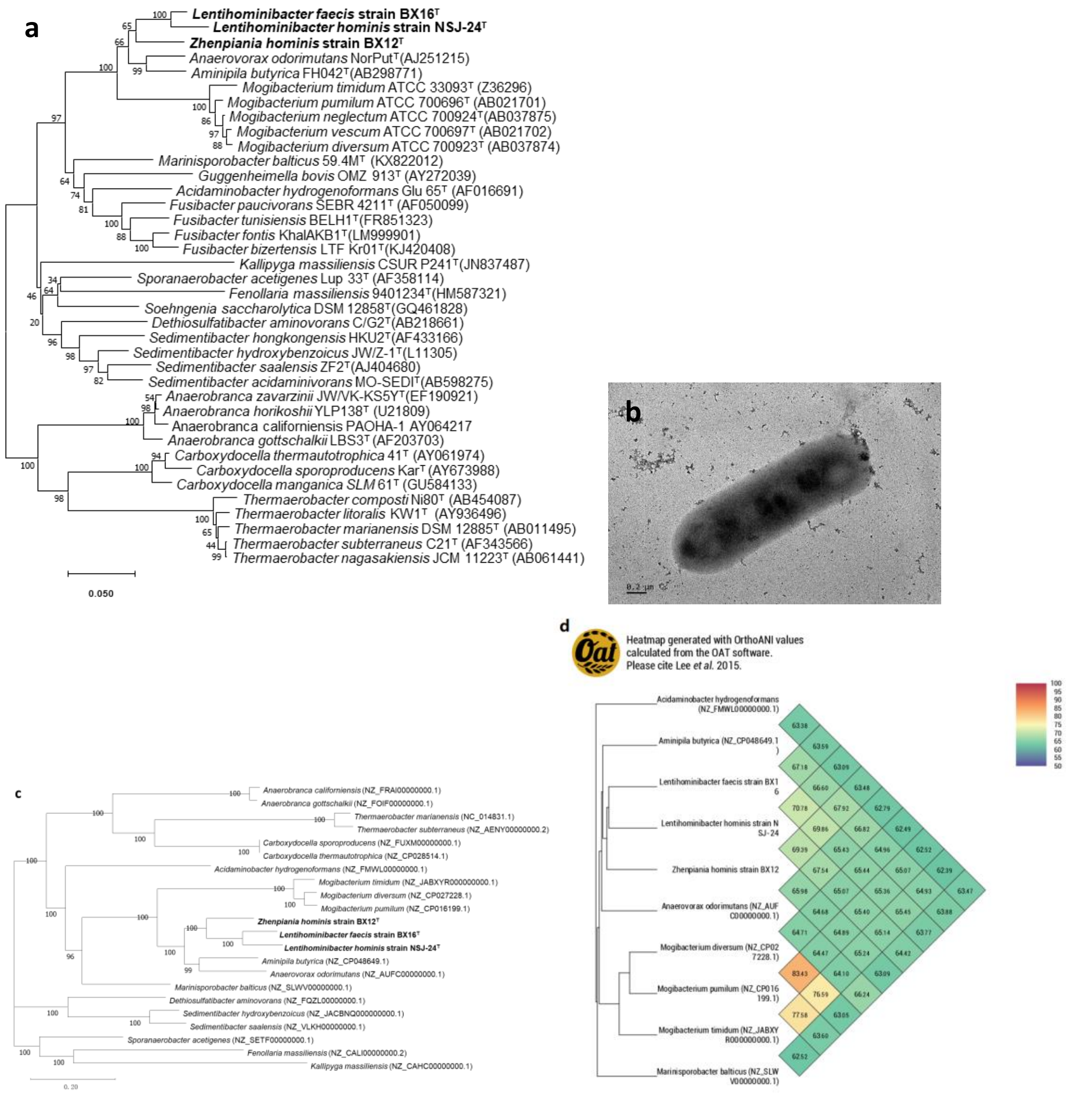

Figure SD-20. The Neighbour-joining phylogenetic tree based on 16S rRNA gene sequences (a) and the cell morphology (b) of strain BX16^{T}. GenBank accession numbers are given in parentheses. Percentages of bootstrap support are shown at branch nodes. Bootstrap value was 1000. Bar: 0.05 substitutions per nucleotide position. The phylogenominc tree based on gemome-sequence-alignment (c) and OrthoANI heatmap (d) of strain BX16^{T} and its neighbor species. Refseq ID of representative genomes are given in parentheses. Percentages of bootstrap support are shown at branch nodes. Bar: 0.20 substitutions per nucleotide position.

Taxon 21: *Lentihominibacter hominis* gen. nov. sp. nov.

Description of *Lentihominibacter* gen. nov. (Len.ti.ho.mi.ni.bac'ter. L. masc. n. lentus, slow. L. masc. n. bacter, a rod. N.L. masc. n. *Lentihominibacter*, slowly growing rod-shaped bacterium)
The closest phylogenetic neighbor of Taxon 21 as represented by strain NSJ-24^T, is *Anaerovorax odorimutans* (Figure SD-21a). *A. odorimutans* and strain NSJ-24^T (16S rRNA sequence accession number in NMDC is NMDCN000010L) has 16S rRNA gene identity of 91.8 %, and their DNA G+C content is different by 17.65 %. The genome of strain NSJ-24^T was sequenced, and the NMDC accession number is NMDC60014021. Genome-based analysis on *A. odorimutans* strain and type strain NSJ-24^T reveals that the dDDH and ANI values between *A. odorimutans* NorPut^T (NZ AUFC00000000) and strain NSJ-24^T are 21.90 % and 68.74 %, respectively, and the POCP value is 42.57 %, suggesting *A. odorimutans* and strain NSJ-24^T are from different genera of the order *Eubacteriales*. According to the phylogenominc tree (Figure SD-21c), the ANIs and dDDH values of genomes between strain NSJ-24^T (NMDC accession number is NMDC60014021) and phylogenomically neighbored genomes were calculated, and the highest ANI and dDDH values are 70.76 and 19.6 %, respectively, between the query genome of *Lentihominibacter faecis* strain BX16^T (NMDC60014020); and difference in % G+C between genomes of strain NSJ-24^T and *Lentihominibacter faecis* strain BX16^T (NMDC60014020) is 7.73; OrthoANI heatmap (Figure SD-21d) shows the phylogenomic status of the corresponding neighbor species based on OrthoANI values. Cells of the strain NSJ-24^T are straight or slightly curved rods. The G+C content is around 49.12 mol %. The type species is *Lentihominibacter hominis.*

Description of *Lentihominibacter hominis* sp. nov. (ho'mi.nis. L. gen. masc. n. *hominis*, of a human being, referring to the human gut habitat)
Cells are strictly anaerobic, straight or slightly curved rods (1.3-2.7 μm long by 0.6–1.2 μm wide, Figure SD-21b); cells appear singly or in dividing pairs. Irregular, pinpoint, ivory, convex colonies appear on modified mGAM agar plate after 3 days of incubation. Growth occurs at 37°C and at pH of 7.0-7.5. The organism uses D-fructose, L-fucose, D-galactose, D-galacturonic acid, gentiobiose, D-glucosaminic acid, α-D-glucose, glucose-6-phosphate, D-mannose, D-melibiose, 3-melthyl-D-glucose, palatinose, L-rhamnose and pyruvic acid as the carbon source for growth, and weakly metabolizes lactulose and maltose. The G+C content of the type strain NSJ-24^T is around 49.12 mol %. The type strain NSJ-24^T (=CGMCC 1.32824^T) was isolated from the faeces of a healthy adult.

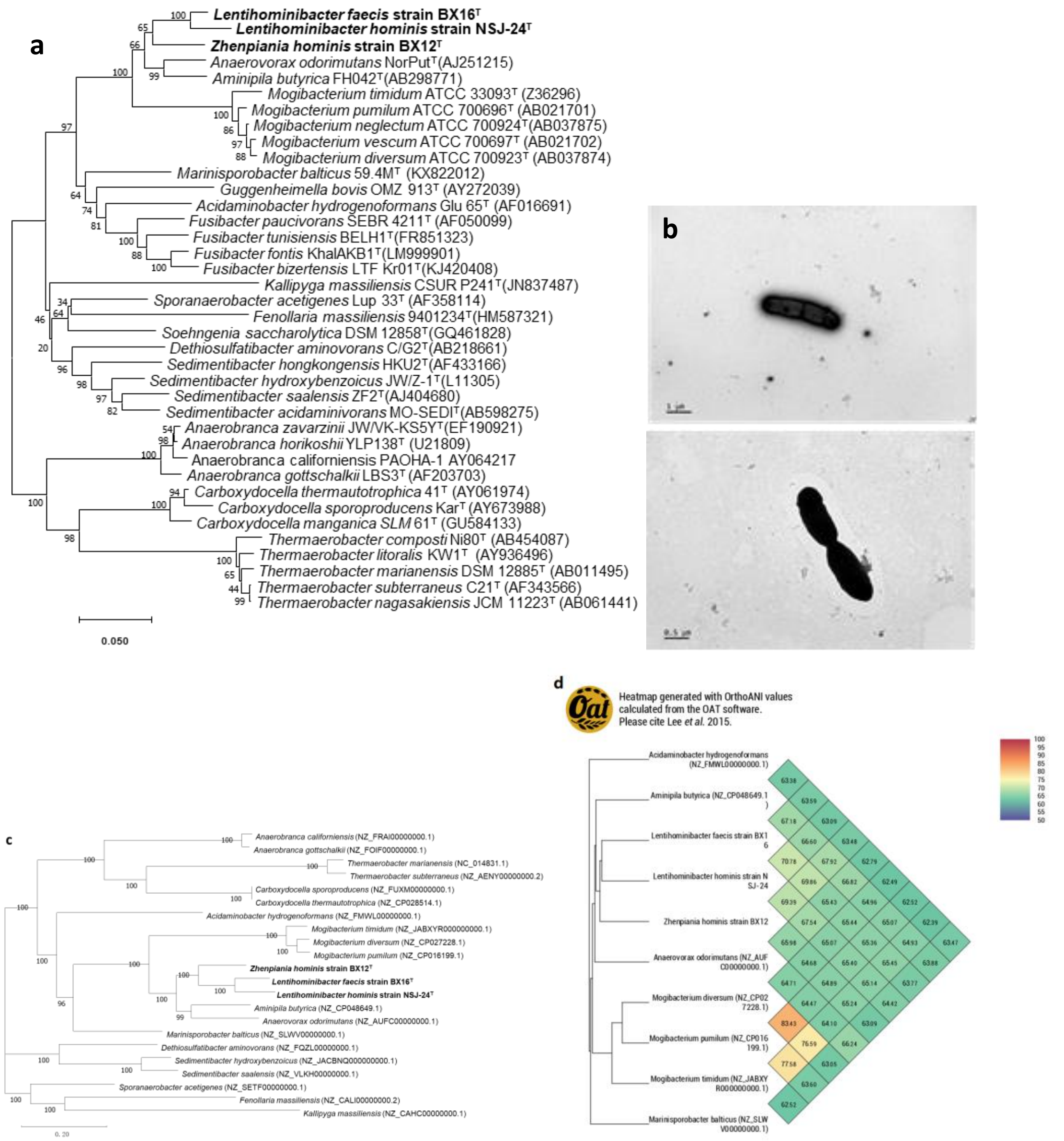

Figure SD-21. The Neighbour-joining phylogenetic tree based on 16S rRNA gene sequences (a) and the cell morphology (b) of strain NSJ-24^T. GenBank accession numbers are given in parentheses. Percentages of bootstrap support are shown at branch nodes. Bootstrap value was 1000. Bar: 0.05 substitutions per nucleotide position. The phylogenominc tree based on gemome-sequence-alignment (c) and OrthoANI heatmap (d) of strain NSJ-24^T and its neighbor species. Refseq ID of representative genomes are given in parentheses. Percentages of bootstrap support are shown at branch nodes. Bar: 0.2 substitutions per nucleotide position.

Taxon 22: *Yanshouia hominis* gen. nov. sp. nov.

Description of *Yanshouia* gen. nov. (Yan.shou'ia N.L. fem. n. *Yanshouia*, named after the Chinese microbiologist Yanshou Wei)

The closest phylogenetic neighbor of Taxon 22 as represented by strain $BX1^T$, is *Anaerotruncus colihominis* (Figure SD-22a). *A. colihominis* and strain $BX1^T$ (16S rRNA sequence accession number in NMDC is NMDCN000010M) has 16S rRNA gene identity of 91.7 %, and their DNA G+C content is different by 2.10 %. The genome of strain $BX1^T$ was sequenced, and the NMDC accession number is NMDC60014022. Genome-based analysis on *A. colihominis* strain and type strain $BX1^T$ reveals that the dDDH and ANI values between *A. colihominis* DSM 17241^T (NZ ABGD00000000) and strain $BX1^T$ are 68.75 % and 25.70 %, respectively, and the POCP value is 34.92 %. According to the phylogenominc tree (Figure SD-22c), the ANIs and dDDH values of genomes between strain $BX1^T$ (NMDC accession number is NMDC60014022) and phylogenomically neighbored genomes were calculated, and the highest ANI and dDDH values are 69.3 and 25.7 %, respectively, between the query genome of *Anaerotruncus colihominis* (NZ_ABGD00000000.2); and difference in % G+C between genomes of strain $BX1^T$ and *Anaerotruncus colihominis* (NZ_ABGD00000000.2) is 2.11; OrthoANI heatmap (Figure SD-22d) shows the phylogenomic status of the corresponding neighbor species based on Ortho-ANI values. These results suggested *A. colihominis* and strain $BX1^T$ are from different genera of the family *Oscillospiraceae*. Cells of strain $BX1^T$ are rod-shaped, and some are in shape of curved rods. The G+C content is around 56.30 mol %. The type species is *Yanshouia hominis.*

Description of *Yanshouia hominis* sp. nov. (ho'mi.nis. L. gen. masc. n. *hominis*, of a human being, referring to the human gut habitat)

Cells mostly are in shape of rods, and some are in shape of curved rods. (1.2-2.9 µm long by 0.9-1.2 µm wide, Figure SD-22b); cells appear singly or in dividing pairs. Growth occurs at 37°C and at pH range of 7.0-7.5. The G+C content of the type strain $BX1^T$ is 56.30 mol %. The type species is *Yanshouia hominis.* The type strain $BX1^T$ (=CGMCC 1.32879^T) was isolated from the faeces of a healthy adult.

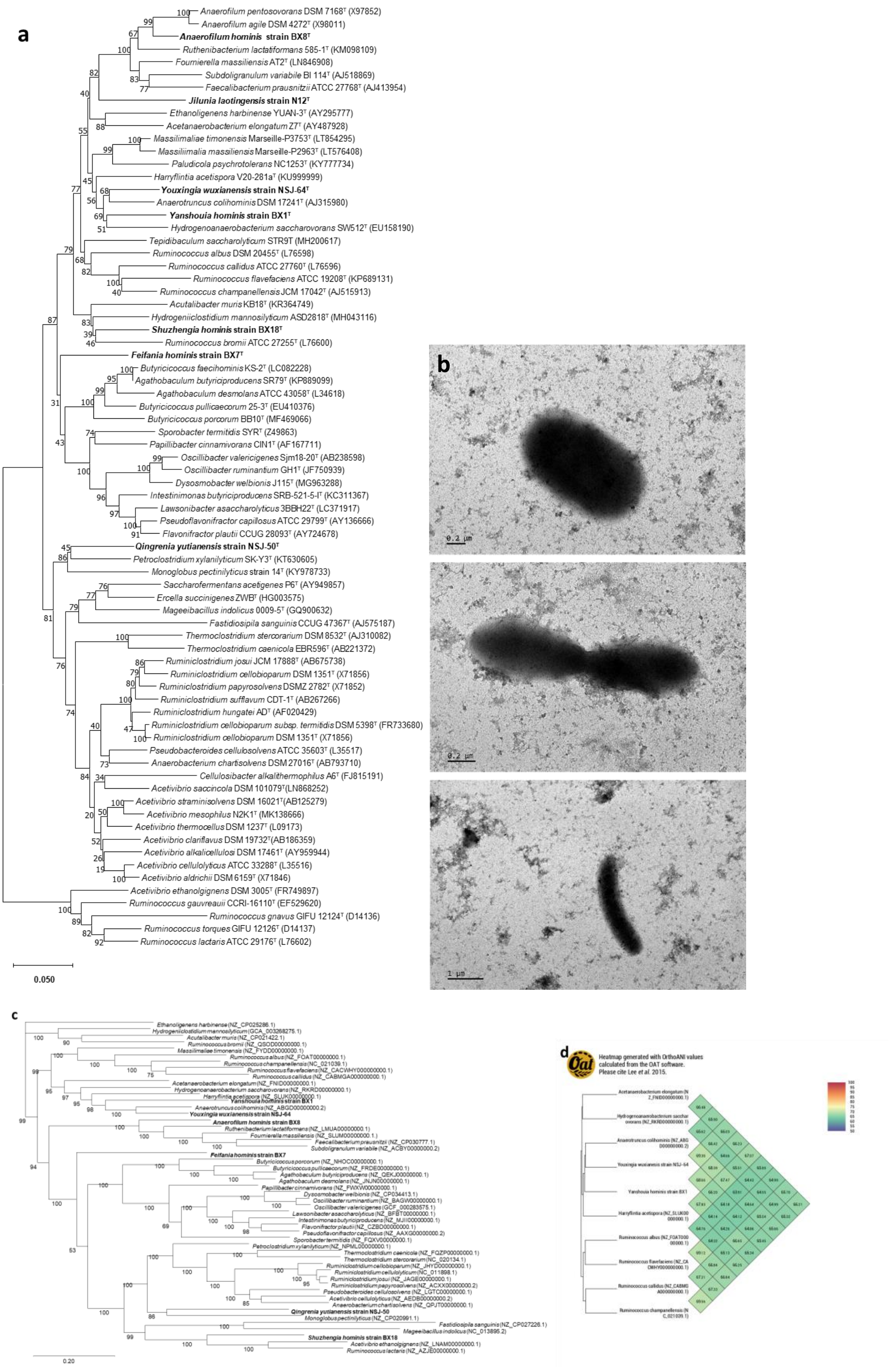

Figure SD-22. The Neighbour-joining phylogenetic tree based on 16S rRNA gene sequences (a) and the cell morphology (b) of strain BX1^T. GenBank accession numbers are given in parentheses. Percentages of bootstrap support are shown at

branch nodes. Bootstrap value was 1000. Bar: 0.05 substitutions per nucleotide position. The phylogenomininc tree based on gemome-sequence-alignment (c) and OrthoANI heatmap (d) of strain BX1^{T} and its neighbor species. Refseq ID of representative genomes are given in parentheses. Percentages of bootstrap support are shown at branch nodes. Bar: 0.2 substitutions per nucleotide position.

Taxon 23: *Shuzhengia hominis* gen. nov. sp. nov.

Description of *Shuzhengia* gen. nov.

Shuzhengia (Shu.zheng'i.a. N.L. fem. n. *Shuzhengia*, named after the Chinese microbiologist Shuzheng Zhang)

The closest phylogenetic neighbor of Taxon 23 as represented by strain BX18^T, is *Hydrogeniiclostridium mannosilyticum* (Figure SD-23a). *H. mannosilyticum* and strain BX18^T (16S rRNA sequence accession number in NMDC is NMDCN000010N) has 16S rRNA gene identity of 91.9 %, and their DNA G+C content is different by 1.0 %. The genome of strain BX18^T was sequenced, and the NMDC accession number is NMDC60014023. Genome-based analysis on *H. mannosilyticum* strain and type strain BX18^T reveals that the dDDH and ANI values between *H. mannosilyticum* ASD2818^T (NZ_JADPHD000000000.1) and strain BX18^T are 64.39 % and 22.70 %, respectively, and the POCP value is 21.63 %, suggesting *H. mannosilyticum* and strain BX18^T are from different genera of the family *Oscillospiraceae*. According to the phylogenominc tree (Figure SD-23c), the ANIs and dDDH values of genomes between strain BX18^T (NMDC accession number is NMDC60014023) and phylogenomically neighbored genomes were calculated, and the highest ANI and dDDH values are 68.54 and 32.1 %, respectively, between the query genome of *Ruminococcus lactaris* (NZ_AZJE00000000.1); and difference in % G+C between genomes of strain BX18^T and *Ruminococcus lactaris* (NZ_AZJE00000000.1) is 2.81; OrthoANI heatmap (Figure SD-23d) shows the phylogenomic status of the corresponding neighbor species based on OrthoANI values. Cells of strain BX18^T are straight rods. The G+C content is around 45.40 mol %. The type species is *Shuzhengia hominis.*

Description of *Shuzhengia hominis* sp. nov. (ho'mi.nis. L. gen. masc. n. *hominis*, of a human being, referring to the human gut habitat)

Cells are strictly anaerobic straight rods (1.4-2.1 µm long by 0.8-1.0 µm wide, Figure SD-23b); cells appear singly. Growth occurs at 37°C and at pH range of 7.0-7.5. The G+C content of the type strain BX18^T is 45.40 mol %. The type species is *Shuzhengia hominis.* The type strain BX18^T (=CGMCC 1.32880^T) was isolated from the faeces of a healthy adult.

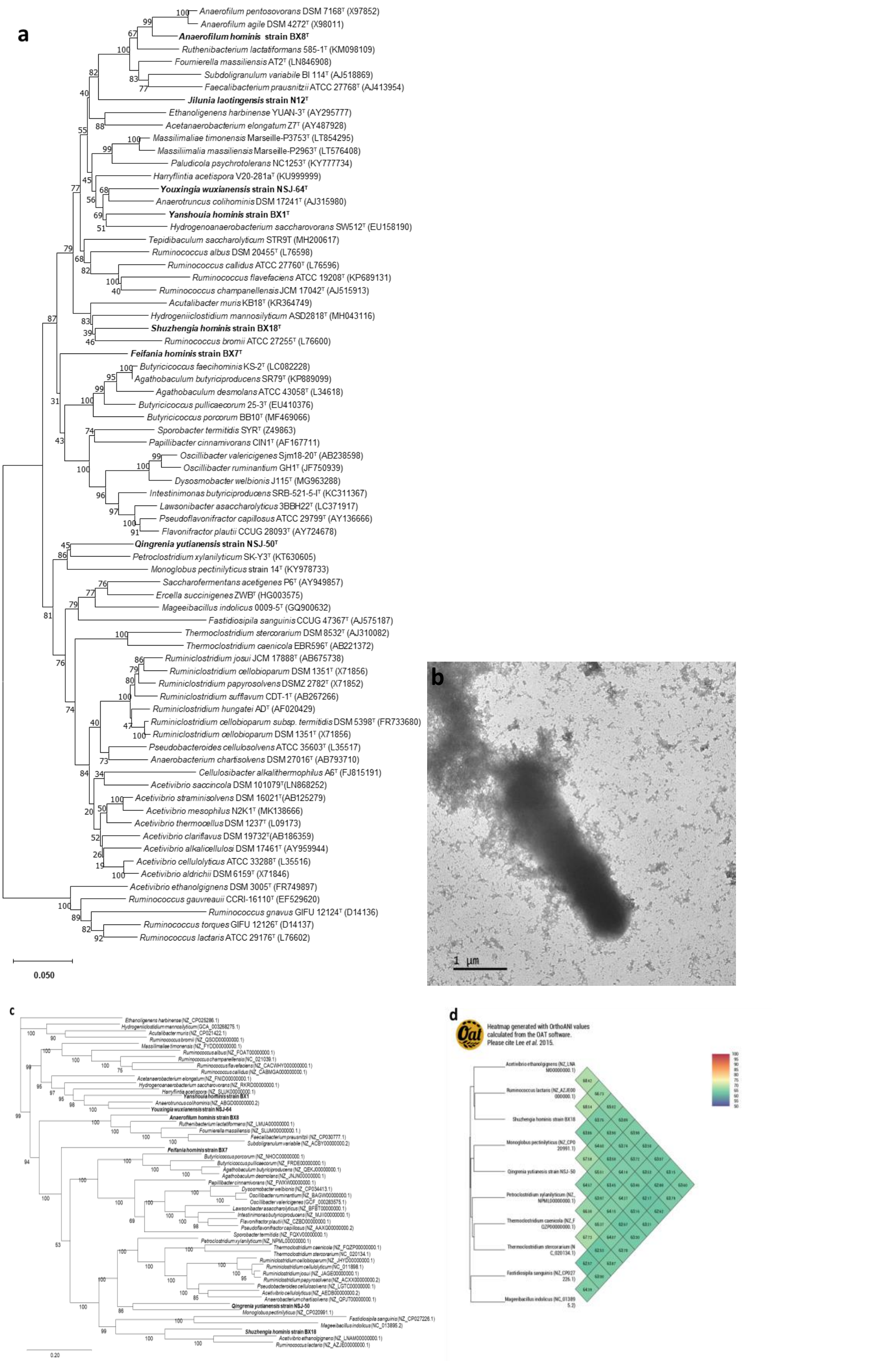

Figure SD-23. The Neighbour-joining phylogenetic tree based on 16S rRNA gene sequences (a) and the cell morphology (b) of strain BX18^{T}. GenBank accession numbers are given in parentheses. Percentages of bootstrap support are shown at

branch nodes. Bootstrap value was 1000. Bar: 0.05 substitutions per nucleotide position. The phylogenominc tree based on gemome-sequence-alignment (c) and OrthoANI heatmap (d) of strain BX18^{T} and its neighbor species. Refseq ID of representative genomes are given in parentheses. Percentages of bootstrap support are shown at branch nodes. Bar: 0.20 substitutions per nucleotide position.

Taxon 24 : *Anaerofilum hominis* sp. nov.

Description of *Anaerofilum hominis* sp. nov. (ho'mi.nis L. gen. masc. n. *hominis*, of a human being, referring to the human gut habitat)

The taxon 24, represented by strain BX8^T, is closest to *Anaerofilum pentosovorans*, with 16S rRNA gene identity of 94.6 %. Phylogenetic tree shows that strain BX8^T (16S rRNA sequence accession number in NMDC is NMDCN000010O) clusters with other members of the genus *Anaerofilum* (Figure SD-24a), suggesting that strain BX8^T is a new member of the genus *Anaerofilum.* The genome of strain BX8^T was sequenced and the NMDC accession number is NMDC60014024. Genome-based analysis on *A. pentosovorans* strain Fae^T (NZ NFJT00000000) and type strain BX8^T reveals that the ANI value is 70.44 % and the dDDH estimation is 19.10 %. According to the phylogenominc tree (Figure SD-24c), the ANIs and dDDH values of genomes between strain BX8^T (NMDC accession number is NMDC60014024) and phylogenomically neighbored genomes were calculated, and the highest ANI and dDDH values are 69.87 and 19.2 %, respectively, between the query genome of *Fournierella massiliensis* (NZ_SLUM00000000.1; and difference in % G+C between genomes of strain BX8^T and *Fournierella massiliensis* (NZ_SLUM00000000.1 is 4.8; OrthoANI heatmap (Figure SD-24d) shows the phylogenomic status of the corresponding neighbor species based on OrthoANI values. Based on these results, we concluded that the strain BX8^T represents a new species of the genus *Anaerofilum*, and the name *Anaerofilum hominis* sp. nov. is proposed.

Cells are strictly anaerobic straight rods (1.2-2.6 µm long by 0.8-1.1 µm wide, Figure SD-74b); cells appear singly, in dividing pairs or in chains. Growth occurs at 37°C and at pH range of 7.0-7.5. The G+C content of the type strain BX8^T is 61.60 mol %. The type species is *Anaerofilum hominis.* The type strain BX8^T (=CGMCC 1.32881^T) was isolated from the faeces of a healthy adult.

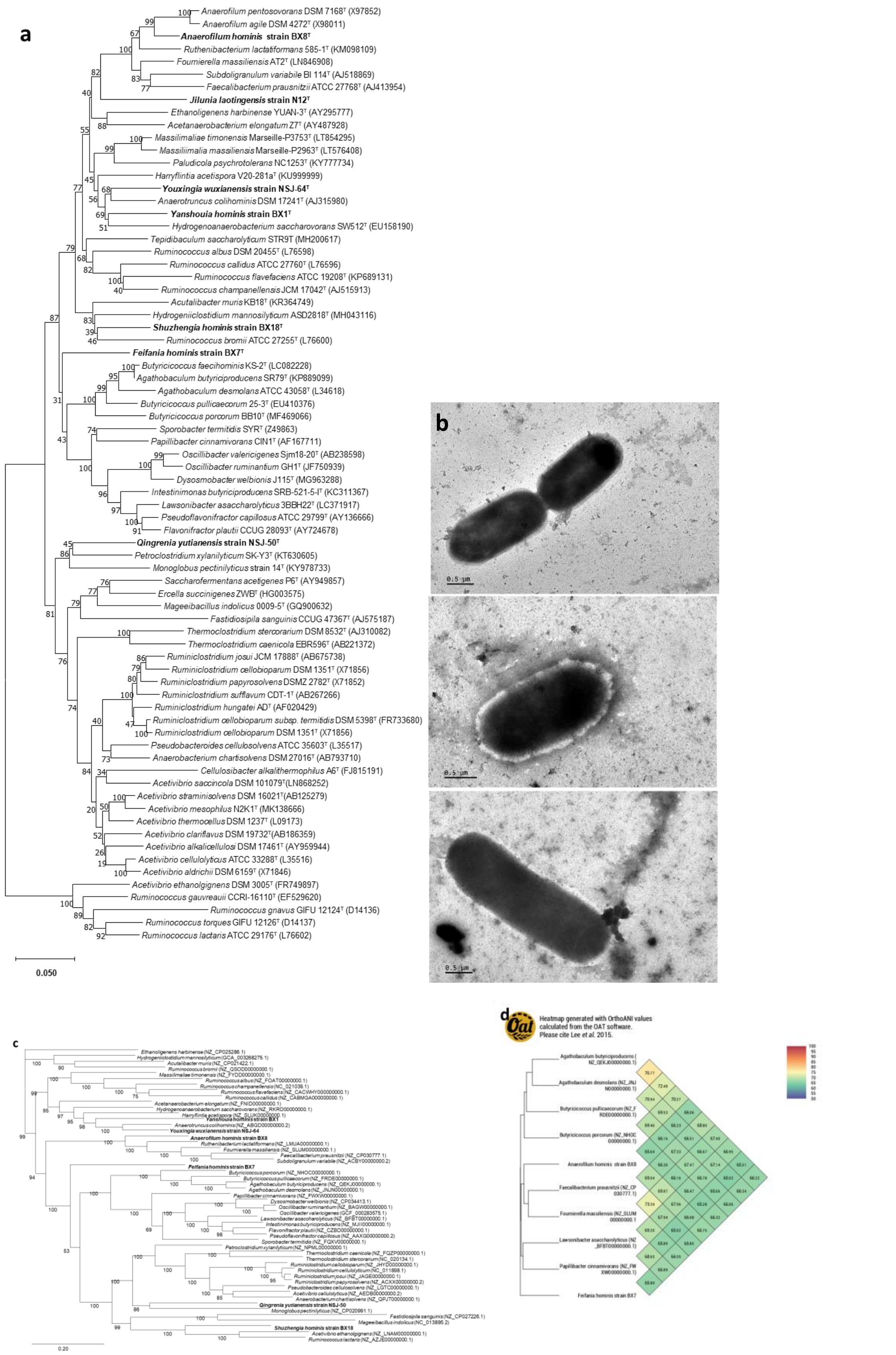

Figure SD-24. The Neighbour-joining phylogenetic tree based on 16S rRNA gene sequences (a) and the cell morphology (b) of strain BX8^{T}. GenBank accession numbers are given in parentheses. Percentages of bootstrap support are shown at

branch nodes. Bootstrap value was 1000. Bar: 0.05 substitutions per nucleotide position. The phylogenomiNC tree based on gemome-sequence-alignment (c) and OrthoANI heatmap (d) of strain BX8^T and its neighbor species. Refseq ID of representative genomes are given in parentheses. Percentages of bootstrap support are shown at branch nodes. Bar: 0.20 substitutions per nucleotide position.

Taxon 25 : *Zongyangia hominis* gen. nov. sp. nov.

Description of *Zongyangia* gen. nov. (Zong.yang'i.a. N.L. fem. n. *Zongyangia*, named after the Chinese medical scientist Zongyang Yang)

The closest phylogenetic neighbor of Taxon 25 as represented by strain NSJ-54^T, is *Harryflintia acetispora* (Figure SD-25a). *Harryflintia acetispora* and strain NSJ-54^T (16S rRNA sequence accession number in NMDC is NMDCN000010P) has 16S rRNA gene identity of 92.52 %, and their DNA G+C content is different by 2.6 %. The genome of strain NSJ-54^T was sequenced, the NMDC accession number is NMDC60014025, and genome-based analysis on *Harryflintia acetispora* strain and type strain NSJ-54^T reveals that the dDDH and ANI values between *Harryflintia acetispora* DSM 100433^T (NZ SLUK00000000) and strain NSJ-54^T are 68.55 % and 18.00 %, respectively, and the POCP value is 46.41 %, suggesting *Harryflintia acetispora* and strain NSJ-54^T are from different genera of the family *Oscillospiraceae.* According to the phylogenominc tree (Figure SD-25c), the ANIs and dDDH values of genomes between strain NSJ-54^T (NMDC accession number is NMDC60014025) and phylogenomically neighbored genomes were calculated, and the highest ANI and dDDH values are 68.92 and 18 %, respectively, between the query genome of *Harryflintia acetispora* (NZ_SLUK00000000.1); and difference in % G+C between genomes of strain NSJ-54^T and *Harryflintia acetispora* (NZ_SLUK00000000.1) is 3.66; OrthoANI heatmap (Figure SD-25d) shows the phylogenomic status of the corresponding neighbor species based on OrthoANI values. Cells of the strain NSJ-54^T are straight or slightly curved rods. The G+C content is around 56.40 mol %. The type species is *Zongyangia hominis*

Description of *Zongyangia hominis* sp. nov. (ho'mi.nis L. gen. masc. n. *hominis*, of a human being, referring to the human gut habitat)

The taxon 25, represented by strain NSJ-54^T, is closest to *Harryflintia acetispora*, with 16S rRNA gene identity of 92.52 %. Phylogenetic tree shows that strain NSJ-54^T clusters with other members of the genus *Dialister* (Figure SD-25a), suggesting that strain NSJ-54^T is a member of the genus *Dialister.* The genome of strain NSJ-54^T was sequenced and the NMDC accession number is. Genome-based analysis on *Harryflintia acetispora* strain V20-281a^T (ASM434012v1) and type strain NSJ-54^T reveals that the ANI value is 68.55 % and the dDDH estimation is 18.00 %. Based on these results, we concluded that the strain NSJ-54^T represents a new species of the genus *Zongyangia*, and the name *Zongyangia hominis sp. nov.* is proposed.

Cells are strictly anaerobic, straight rods with spiky ends in various sizes (0.8-6.0 µm long by 0.6-0.8 µm wide, Figure SD-25b); cells appear singly, in dividing pairs or in dividing chains. Growth occurs at 37°C and at pH 7.0-7.5. The organism uses amygdalin, D-cellobiose, dextrin, D-fructose, L-fucose, D-galactose, D-galacturonic acid, gentiobiose, α-D-glucose, glucose-6-phosphate, lactulose, maltotriose, D-mannose, D-melibiose, 3-melthyl-D-glucose, palatinose, D-raffinose, L-rhamnose, stachyose, sucrose, turanose, glyoxylic acid, α-ketobutyric acid, a-ketovaleric acid, pyruvic acid, pyruvic acid methyl ester, L-valine, L-valine plus L-aspartic acid, inosine,

thymidine and uridine as the carbon source for growth, and weakly metabolizes D-gluconic acid, D-glucosaminic acid, glycerol, maltose, D-sorbitol, glycyl-L-glutamine, L-phenylalanine and 2'-deoxy adenosine. The DNA G+C content of the type strain NSJ-54^T is 56.40 mol %. The type strain is NSJ-54^T (=CGMCC 1.32830^T=KCTC 25132^T), which was isolated from the faeces of a healthy adult.

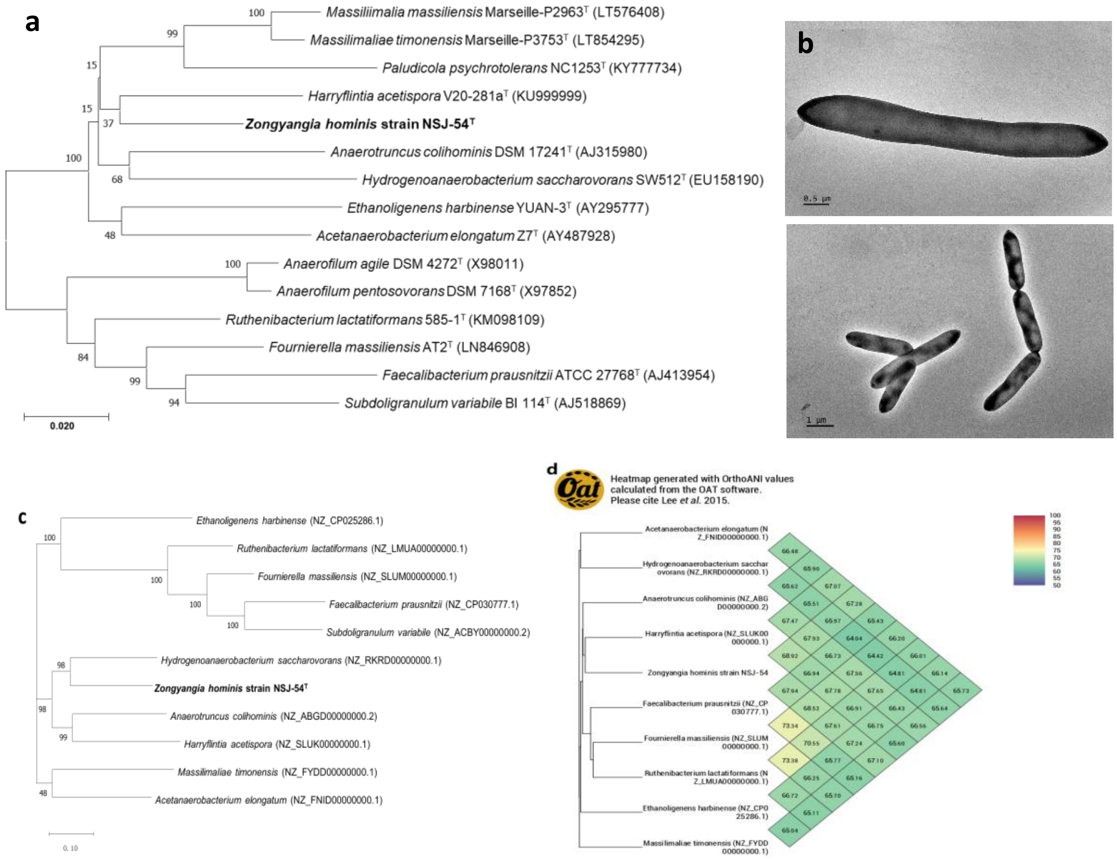

Figure SD-25. The Neighbour-joining phylogenetic tree based on 16S rRNA gene sequences (a) and the cell morphology (b) of strain NSJ-54^T. GenBank accession numbers are given in parentheses. Percentages of bootstrap support are shown at branch nodes. Bootstrap value was 1000. Bar: 0.01 substitutions per nucleotide position. The phylogenominc tree based on gemome-sequence-alignment (c) and OrthoANI heatmap (d) of strain NSJ-54^T and its neighbor species. Refseq ID of representative genomes are given in parentheses. Percentages of bootstrap support are shown at branch nodes. Bar: 0.10 substitutions per nucleotide position.

Taxon 26: *Youxingia wuxianensis* gen. nov. sp. nov.

Description of *Youxingia* gen. nov.

Youxingia (You.xing'i.a. N.L. fem. n. *Youxingia*, named after the Chinese medical scientist Youxing Wu)

The closest phylogenetic neighbor of Taxon 26 as represented by strain NSJ-64^T, is *Anaerotruncus colihominis* (Figure SD-26a). *A. colihominis* and strain NSJ-64^T (16S rRNA sequence accession number in NMDC is NMDCN000010Q) has 16S rRNA gene identity of 93 %,and their DNA G+C content is different by 4.21 %. The genome of strain NSJ-64^T was sequenced and the NMDC accession number is NMDC60014026. Genome-based analysis on *A. colihominis* DSM 17241^T (NZ DS544194) and type strain NSJ-64^T reveals that the dDDH and ANI values between *A. colihominis* DSM 17241^T and strain NSJ-64^T are 26.70 % and 76.04 %, respectively, and the POCP value is 48.29 %, suggesting *A. colihominis* and strain NSJ-64^T are from different genera. According to the phylogenominc tree (Figure SD-26c), the ANIs and dDDH values of genomes between strain NSJ-64^T (NMDC accession number is NMDC60014026) and phylogenomically neighbored genomes were calculated, and the highest ANI and dDDH values are 68.66 and 39.8 %, respectively, between the query genome of *Yanshouia hominis* strain BX1^T (NMDC60014022); and difference in % G+C between genomes of strain NSJ-64^T and *Yanshouia hominis* strain BX1^T (NMDC60014022) is 8.64; OrthoANI heatmap (Figure SD-26d) shows the phylogenomic status of the corresponding neighbor species based on OrthoANI values. Cells of the strain NSJ-64^T are strictly anaerobic rods with tapered ends. The G+C content is around 58.41 mol %. The type species is *Youxingia wuxianensis.*

Description of *Youxingia wuxianensis* sp. nov. (wu.xian.en'sis. N.L. fem. adj. *wuxianensis*, referring to the Wuxian county of China, where Youxing Wu was born)

Cells are strictly anaerobic rods with tapered ends in various sizes (0.6-3.1 µm long by 0.5-0.8 µm wide, Figure SD-26b); cells appear singly, in dividing pairs or in chains. Tiny, circular, whitish, raised colonies appear on modified mGAM agar plate after 3 days of incubation. Growth occurs at 37°C and at pH range of 7.0-7.5. The organism uses D-galacturonic acid, α-D-glucose and succinic acid mono-methyl ester as the carbon source for growth, and weakly metabolizes dextrin, D-mannose, sucrose, turanose, L-lactic acid, L-serine, 2’-deoxy adenosine, thymidine. The G+C content of the type strain NSJ-64^T is 58.41 mol %. The type species is *Youxingia wuxianensis.* The type strain NSJ-64^T (=CGMCC 1.32840^T =KCTC 25128^T) was isolated from the faeces of a healthy adult.

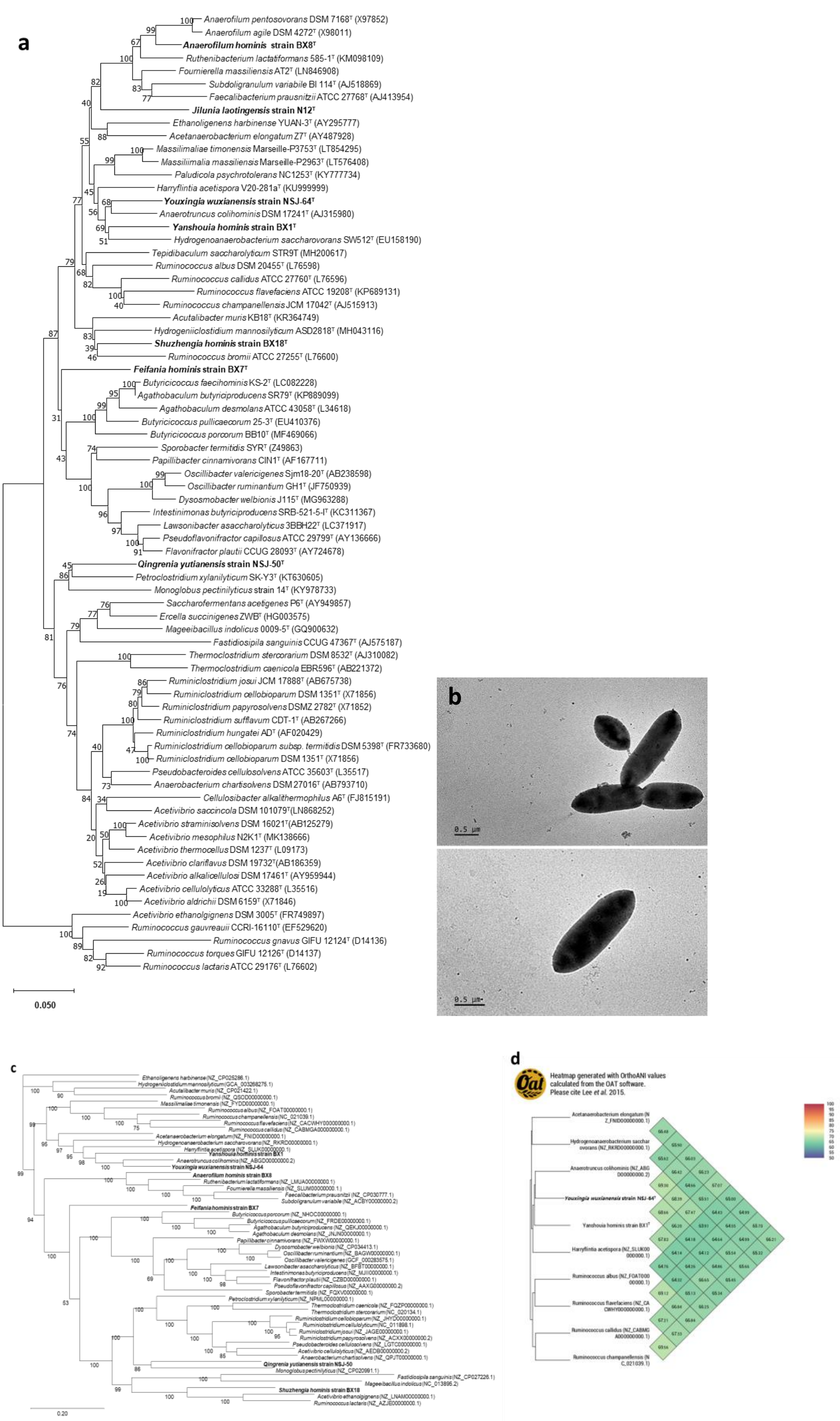

Figure SD-26. The Neighbour-joining phylogenetic tree based on 16S rRNA gene sequences (a) and the cell morphology (b) of strain NSJ-64^{T}. GenBank accession

numbers are given in parentheses. Percentages of bootstrap support are shown at branch nodes. Bootstrap value was 1000. Bar: 0.05 substitutions per nucleotide position. The phylogenominc tree based on gemome-sequence-alignment (c) and OrthoANI heatmap (d) of strain NSJ-64^{T} and its neighbor species. Refseq ID of representative genomes are given in parentheses. Percentages of bootstrap support are shown at branch nodes. Bar: 0.20 substitutions per nucleotide position.

Taxon 27: *Qingrenia yutianensis* gen. nov. sp. nov.

Description of *Qingrenia* gen. nov. (Qing.re'ni.a N.L. fem. n. *Qingrenia*, named after the Chinese medical scientist Qingren Wang)
The closest phylogenetic neighbor of Taxon 27 as represented by strain NSJ-50^T, is *Petroclostridium xylanilyticum* (Figure SD-27a). *P. xylanilyticum* and strain NSJ-50^T (16S rRNA sequence accession number in NMDC is NMDCN000010R) has 16S rRNA gene identity of 91 %, and their DNA G+C content is different by 18.06 %. The genome of strain NSJ-50^T was sequenced, the NMDC accession number is NMDC60014027, and genome-based analysis on *P. xylanilyticum* strain and type strain NSJ-50^T reveals that the dDDH and ANI values between *P. xylanilyticum* SK-Y3^T (NZ NPML00000000) and strain NSJ-50^T are 30.70 % and 66.37 %, respectively, and the POCP value is 30.70 %, suggesting *P. xylanilyticum* and strain NSJ-50^T are from different genera of the family *Oscillospiraceae*. According to the phylogenominc tree (Figure SD-27c), the ANIs and dDDH values of genomes between strain NSJ-50^T (NMDC accession number is NMDC60014027) and phylogenomically neighbored genomes were calculated, and the highest ANI and dDDH values are 67.58 and 36.2 %, respectively, between the query genome of *Monoglobus pectinilyticus* (NZ_CP020991.1); and difference in % G+C between genomes of strain NSJ-50^T and *Monoglobus pectinilyticus* (NZ_CP020991.1) is 5.48; OrthoANI heatmap (Figure SD-27d) shows the phylogenomic status of the corresponding neighbor species based on OrthoANI values. Cells of the strain NSJ-50^T are oval shaped with spiky ends. The G+C content is around 55.26 mol %. The type species is *Qingrenia yutianensis.*
Description of *Qingrenia yutianensis* sp. nov. (yu.tian.en'sis. N.L. fem. adj. *yutianensis*, referring to Yutian county of China, where Qingren Wang was born)
Cells are strictly anaerobic and oval shaped with spiky ends (1.5-2.8 μm long by 0.9-1.1 μm wide, Figure SD-27b); cells appear singly, in dividing pairs or in chains. Pinpoint, whitish, slightly raised tiny colonies appear on modified mGAM agar plate after3 days of incubation. Growth occurs at 37°C and at pH range of 7.0-7.5. The organism uses D-fructose, L-fucose, D-galactose, D-galacturonic acid, gentiobiose, α-D-glucose, glucose-6-phosphate, D-mannose, D-melibiose, 3-melthyl-D-glucose, palatinose, L-rhamnose, glyoxylic acid, α-ketobutyric acid and pyruvic acid as the carbon source for growth, and weakly metabolizes dextrin and pyruvic acidmethyl ester. The G+C content of the type strain NSJ-50^T is 55.26 mol %. The type species is *Qingrenia yutianensis.* The type strain NSJ-50^T (=CGMCC 1.32823^T) was isolated from the faeces of a healthy adult.

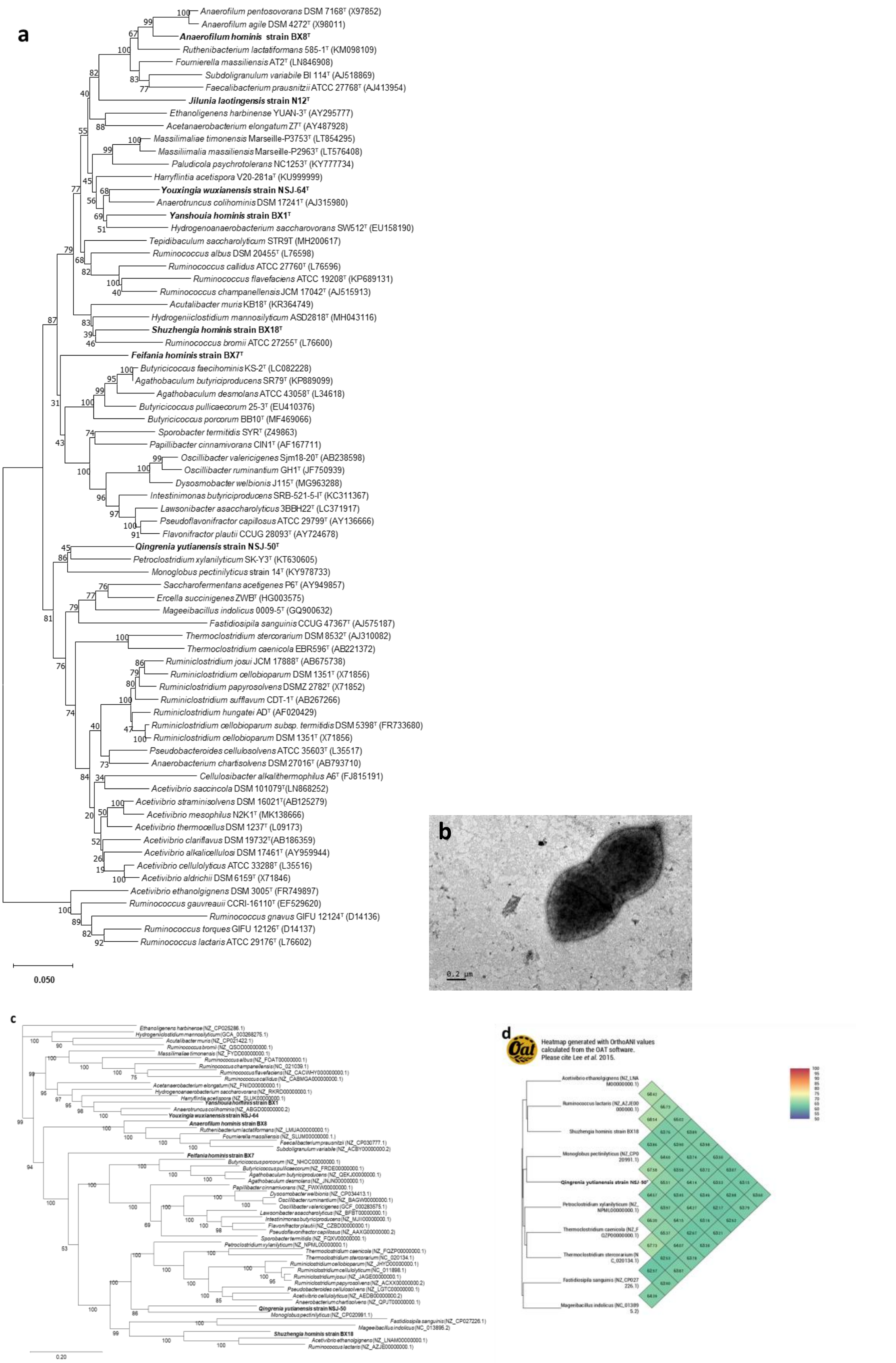

Figure SD-27. The Neighbour-joining phylogenetic tree based on 16S rRNA gene sequences (a) and the cell morphology (b) of strain NSJ-50^T. GenBank accession numbers are given in parentheses. Percentages of bootstrap support are shown at

branch nodes. Bootstrap value was 1000. Bar: 0.05 substitutions per nucleotide position. The phylogenominc tree based on gemome-sequence-alignment (c) and OrthoANI heatmap (d) of strain NSJ-50^{T} and its neighbor species. Refseq ID of representative genomes are given in parentheses. Percentages of bootstrap support are shown at branch nodes. Bar: 0.20 substitutions per nucleotide position.

Taxon 28: *Jilunia laotingensis* gen. nov. sp. nov.

Description of *Jilunia* gen. nov. (lao.ting.en'sis. N.L. fem. adj. *laotingensis*, referring to the Laoting county where Jilun Li was born)

The taxon 28 as represented by strain N12^T (16S rRNA sequence accession number in NMDC is NMDCN000010S) and *Anaerotruncus colihominis* has 16S rRNA gene identity of 89.13 %,and their DNA G+C content is different by 12.56 %. The phylogenetic tree based on 16S rRNA sequence revealed that the strain N12^T (16S rRNA sequence accession number in NMDC is NMDCN000010S) does not cluster with any species of the genus *Anaerotruncus* or the members of any other genus within the family *Oscillospiraceae*.(Figure SD-28a). The genome of strain N12^T was sequenced and the NMDC accession number is NMDC60014028. Genome-based analysis on *A. colihominis* DSM 17241^T (NZ DS544194) and type strain N12^T reveals that the dDDH and ANI values between *A. colihominis* DSM 17241^T and strain N12^T are 22.30 % and 77.42 %, respectively, and the POCP value is 9.8 %, suggesting *A. colihominis* and strain N12^T are from different genera. Based on further genomic DNA analysis, we concluded strain N12^T represents a novel genera of the family Bacteroides, and the phylogenetic tree based on 16S rRNA sequence was constructed(Figure SD-28b). According to the phylogenominc tree (Figure SD-28c), the ANIs and dDDH values of genomes between strain N12^T (NMDC accession number is NMDC60014028) and phylogenomically neighbored genomes were calculated, and the highest ANI and dDDH values are 77.27 and 22.1 %, respectively, between the query genome of *Bacteroides salyersiae* (NZ_AGXV00000000.1); and difference in % G+C between genomes of strain N12^T and *Bacteroides salyersiae* (NZ_AGXV00000000.1) is 0.73; OrthoANI heatmap (Figure SD-28d) shows the phylogenomic status of the corresponding neighbor species based on OrthoANI values. Cells of strain N12^T are strictly anaerobic rods with tapered ends. The G+C content is around 41.64 mol %. The type species is *Jilunia laotingensis.*

Description of *Jilunia laotingensis* sp. nov. (lao.ting.en'sis. N.L. fem. adj. *laotingensis*, referring to the Laoting county where Jilun Li was born)

Cells are strictly anaerobic, oval, spherical or short rod shaped (1.2-2.7μm long by 0.9-1.2 μm wide, Figure SD-28c). Irregular, cream-colored, smooth, glossy, convex, entire colonies appear on modified mGAM agar plate after 3 days of incubation. Growth occurs at range 37 °C and at pH range of 7.0-7.5. The organism uses D-fructose, L-fucose, D-galacturonic acid, palatinose, D-malic acid, succinamic acid, succinic acid and succinic acid mono-methyl ester as the carbon source for growth, and weakly metabolizes D-galactose, D-mannose, α-ketobutyric acid, L-malic acid and pyruvic acid. The G+C content of the type strain N12^T is 41.64 mol %. The type strain N12^T (CGMCC 1.32860^T) was isolated from the faeces of a healthy adult.

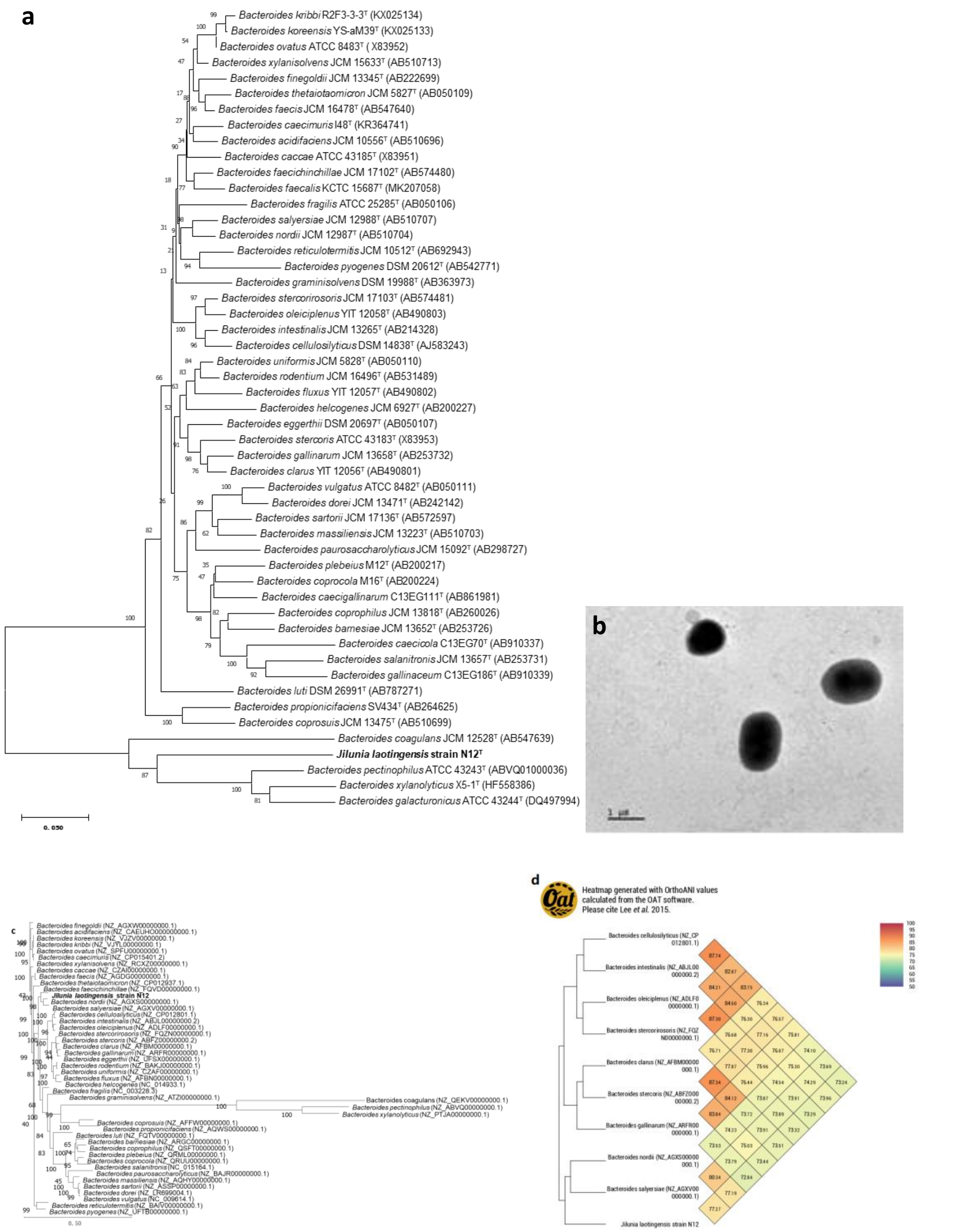

Figure SD-28. The Neighbour-joining phylogenetic tree based on 16S rRNA gene sequences (a, b) and the cell morphology (c) of strain N12T. GenBank accession numbers are given in parentheses. Percentages of bootstrap support are shown at branch nodes. Bootstrap value was 1000. Bar: 0.05 substitutions per nucleotide position. The phylogenominc tree based on gemome-sequence-alignment (c) and OrthoANI heatmap (d) of Jilunia laotingensis strain N12T and its neighbor species. Refseq ID of representative genomes are given in parentheses. Percentages of bootstrap support are shown at branch nodes. Bar: 0.50 substitutions per nucleotide position.

Taxon 29: *Paratissierella segnis* gen. nov. sp. nov.

Description of *Paratissierella* gen. nov. (Pa.ra.tis.sier.el'la. Gr. prep. para, beside. N.L. fem. dim. n. *Tissierella*, a genus name. N.L. masc. n. *Paratissierella*, resembling the genus *Tissierella*)

The closest phylogenetic neighbor of Taxon 29 as represented by strain BX21^T, is *Tissierella creatinini* (Figure SD-29a). *T. creatinini* and strain BX21^T (16S rRNA sequence accession number in NMDC is NMDCN000010T) has 16S rRNA gene identity of 92 %, and their DNA G+C content is different by 2.4 %. The genome of strain BX21^T was sequenced, the NMDC accession number is NMDC60014029, and genome-based analysis on *T. creatinini* strain and type strain BX21^T reveals that the dDDH and ANI values between *T. creatinini* DSM 9508^T (NZ SUSS00000000) and strain BX21^T are 69.09 % and 16.70 %, respectively, and the POCP value is 47.84 %, suggesting *T. creatinini* DSM 9508^T and strain BX21^T are from different genera of the *Tissierellaceae* family. According to the phylogenominc tree (Figure SD-29c), the ANIs and dDDH values of genomes between strain BX21^T (NMDC accession number is NMDC60014029) and phylogenomically neighbored genomes were calculated, and the highest ANI and dDDH values are 70.68 and 17.8 %, respectively, between the query genome of *Tissierella praeacuta* (NZ_UHHZ00000000.1); and difference in % G+C between genomes of strain BX21^T and *Tissierella praeacuta* (NZ_UHHZ00000000.1) is 3.18; OrthoANI heatmap (Figure SD-29d) shows the phylogenomic status of the corresponding neighbor species based on OrthoANI values. Cells of strain BX21^T are rod shaped. The G+C content is around 33.30 mol %. The type species is *Paratisierella segnis.*

Description of *Paratisierella segnis* sp. nov. (L. fem. adj. *segnis*, slow, inactive, lazy, referring the slow growth of the strain)

Cells are strictly anaerobic rods (1.3-3.2 µm long by 0.7-1.1 µm wide, Figure SD-29b); cells appear singly or in dividing pairs. The G+C content of the type strain BX21^T is around 33.30 mol %. The type strain BX21^T (=CGMCC 1.32882^T) was isolated from the faeces of a healthy adult.

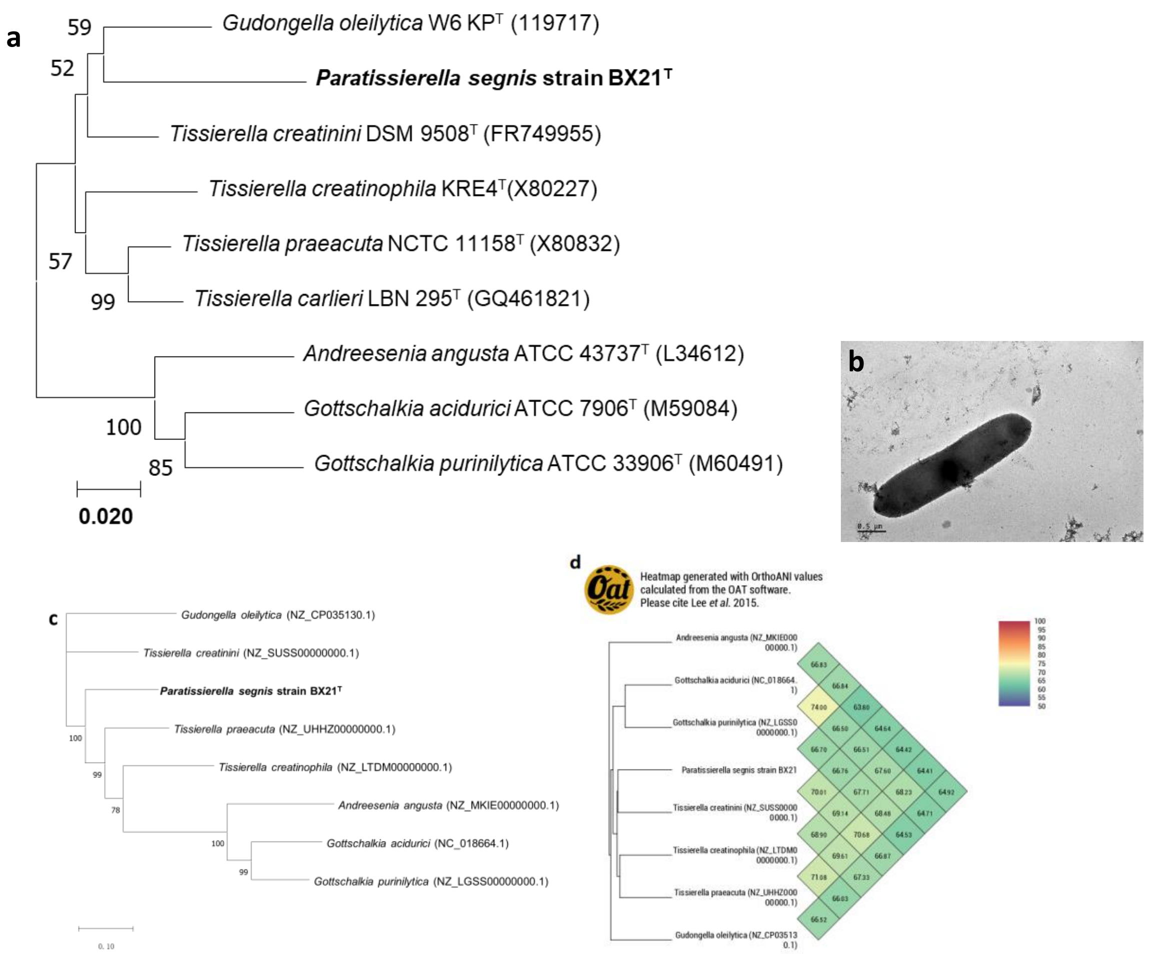

Figure SD-29. The Neighbour-joining phylogenetic tree based on 16S rRNA gene sequences (a) and the cell morphology (b) of strain BX21^T. GenBank accession numbers are given in parentheses. Percentages of bootstrap support are shown at branch nodes. Bootstrap value was 1000. Bar: 0.02 substitutions per nucleotide position. The phylogenominc tree based on gemome-sequence-alignment (c) and OrthoANI heatmap (d) of strain BX21^T and its neighbor species. Refseq ID of representative genomes are given in parentheses. Percentages of bootstrap support are shown at branch nodes. Bar: 0.10 substitutions per nucleotide position.

Taxon 30: *Bittarella massiliensis* sp.

Description of *Bittarella* gen. (N.L. fem. dim. n. *Bittarella*, in honour of Dr. Bittar, a French microbiologist)

The closest phylogenetic neighbor of Taxon 30 as represented by strain NSJ-19^T, is *Harryflintia acetispora* (Figure SD-30a). *Harryflintia acetispora* and strain NSJ-19^T (16S rRNA sequence accession number in NMDC is NMDCN000010U) has 16S rRNA gene identity of 89.11 %, and their DNA G+C content is different by 11.2 %. The genome of strain NSJ-19^T was sequenced, the NMDC accession number is NMDC60014030, and genome-based analysis on *Harryflintia acetispora* strain DSM 100433 and type strain NSJ-19^T reveals that the dDDH and ANI values between *Harryflintia acetispora* V20-281a^T (NZ SLUK00000000) and strain NSJ-19^T are 70.76 % and 21.30 %, respectively, and the POCP value is 37.75 %, suggesting *Harryflintia acetispora* strain DSM 100433 and strain NSJ-19^T are from different genera. According to the phylogenominc tree (Figure SD-30c), the ANIs and dDDH values of genomes between strain NSJ-19^T (NMDC accession number is NMDC60014030) and phylogenomically neighbored genomes were calculated, and the highest ANI and dDDH values are 68.6336 and 22.9 %, respectively, between the query genome of *Fournierella massiliensis* (NZ_SLUM00000000.1); and difference in % G+C between genomes of strain NSJ-19^T and *Fournierella massiliensis* (NZ_SLUM00000000.1) is 6.19; OrthoANI heatmap (Figure SD-30d) shows the phylogenomic status of the corresponding neighbor species based on OrthoANI values. Cells of the strain NSJ-19^T are rod shaped. The G+C content is around 71.15 mol %. The type species is *Bittarella massiliensis.*

Description of *Bittarella massiliensis* sp. (mas.sil.i.en'sis L. masc./fem. adj. *massiliensis*, of Massilia, the Latin name of Marseille where the strain was for the first time isolated, and *Bittarella massiliensis* is the type species of the genus *Bittarella*)

Cells are strictly anaerobic, straight or slightly curved long rods in various sizes (3.0-7.8 µm long by 0.6-0.8µm wide, Figure SD-30b). Circular, yellow to white, semi-translucent, raised, entire colonies appear on modified mGAM agar plate after 6 days of incubation. Growth occurs at temperature range of 30-37 °C and at pH range of 7.0-7.5. The organism uses D-fructose, L-fucose, D-galactose, D-galacturonic acid, gentiobiose, α-D-glucose, glucose-6-phosphate, D-mannose, D-melibiose, 3-melthyl-D-glucose, palatinose and L-rhamnose as the carbon source for growth, and weakly metabolizes D-cellobiose, dextrin, D-glucosaminic acid, lactulose, turanose. The DNA G+C content of the type strain NSJ-19^T is 71.15 mol %. The type strain NSJ-19^T (=CGMCC 1.32824^T=KCTC 25133^T) was isolated from the faeces of a healthy adult.

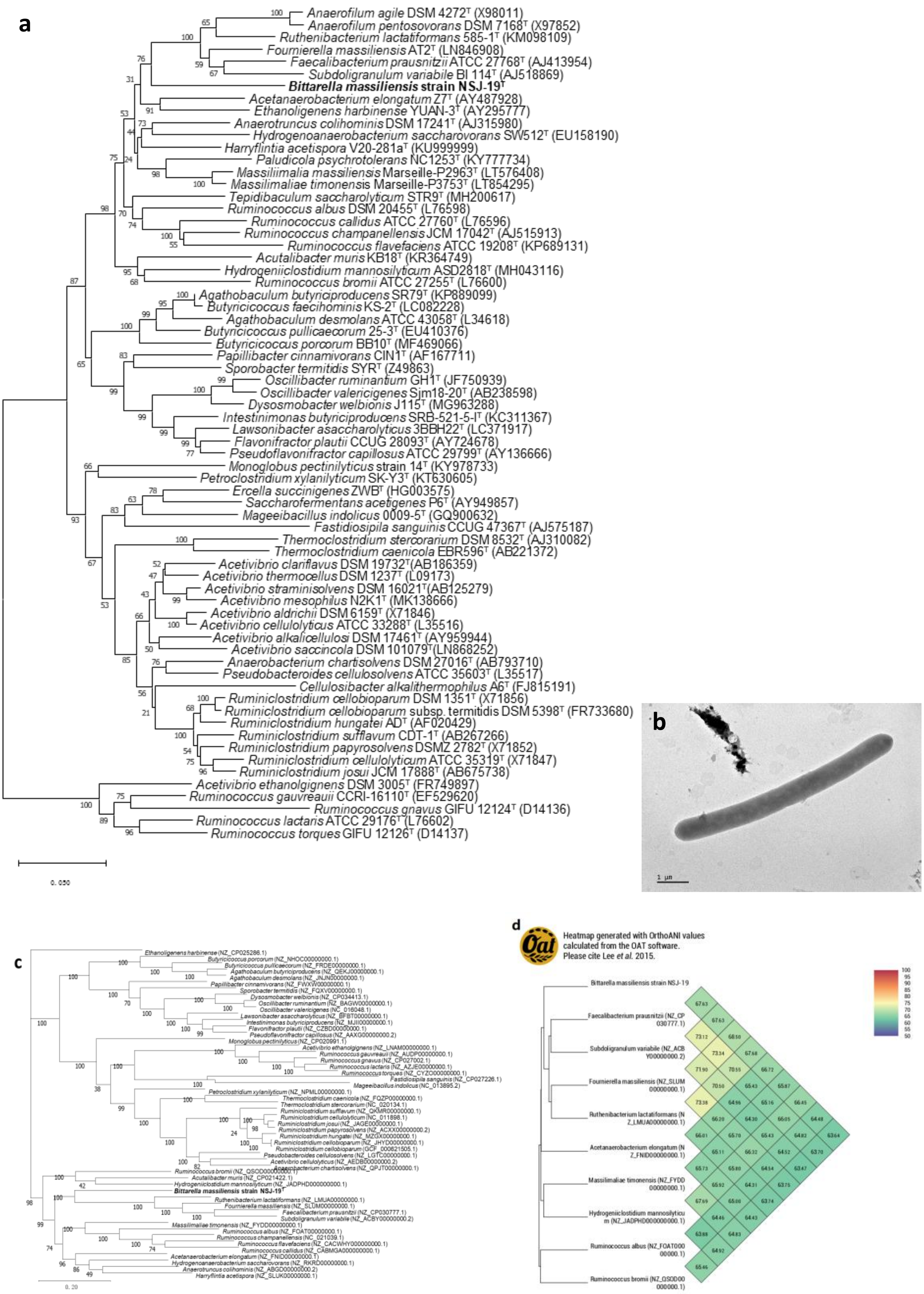

Figure SD-30. The Neighbour-joining phylogenetic tree based on 16S rRNA gene sequences (a) and the cell morphology (b) of strain NSJ-19T. GenBank accession numbers are given in parentheses. Percentages of bootstrap support are shown at branch nodes. Bootstrap value was 1000. Bar: 0.05 substitutions per nucleotide position. The phylogenomic tree based on gemome-sequence-alignment (c) and OrthoANI heatmap (d) of strain NSJ-19T and its neighbor species. Refseq ID of

representative genomes are given in parentheses. Percentages of bootstrap support are shown at branch nodes. Bar: 0.2 substitutions per nucleotide position.

Taxon 32: *Eggerthella hominis* sp. nov.

Description *of Eggerthella hominis* sp. nov. (ho'mi.nis L. gen. masc. n. *hominis*, of a human being, referring to the human gut habitat)

The taxon 32, respresented by strain NSJ-70^T (16S rRNA sequence accession number in NMDC is NMDCN000010V), is phylogenetically closest to *Eggerthella sinensis*, with 16S rRNA gene identity of 97.90 %. Phylogenetic tree shows that strain NSJ-70^T clusters with other members of the genus *Eggerthella,* (Figure SD-32a), suggesting strain NSJ-70^T is a member of the genus *Eggerthella*. The genome of strain NSJ-70^T was sequenced and the NMDC accession number is NMDC60014031. Genome-based analysis showed that the ANI value of genomes of strain NSJ-70^T and *Eggerthella sinensis* HKU14^T (NZ PPTT00000000) is 85.62 % and the dDDH estimation is 30.30 %. According to the phylogenominc tree (Figure SD-32c), the ANIs and dDDH values of genomes between strain NSJ-70^T (NMDC accession number is NMDC60014031) and phylogenomically neighbored genomes were calculated, and the highest ANI and dDDH values are 89.41 and 38.1 %, respectively, between the query genome of *Eggerthella lenta* (NZ_CP021140.1); and difference in % G+C between genomes of strain NSJ-70^T and *Eggerthella lenta* (NZ_CP021140.1) is 2.02; OrthoANI heatmap (Figure SD-32d) shows the phylogenomic status of the corresponding neighbor species based on OrthoANI values. Based on these results, we concluded that the strain NSJ-70^T represents of new species of the genus *Eggerthella*, and the name *Eggerthella hominis* sp. nov. is proposed.

Cells are strictly anaerobic straight rods (1.2-2.5 μm long by 0.6-0.8 μm wide, Figure SD-32b); cells appear singly or in dividing pairs. Cream-colored, flat-to-raised, pinpoint colonies appear on modified mGAM agar plate after 3 days of incubation. Growth occurs at 37°C and at pH 7.0-7.5. The organism uses D-arabitol, D-galactose, D-galacturonic acid, ß-hydroxybutyric acid, α-methyl-D-galactoside, D-gluconic acid, α-D-glucose, glycerol, 3-melthyl-D-glucose, ß-methyl-D-glucoside, palatinose, turanose, D,L-lactic acid, D-lactic acidmethyl ester, succinamic acid, m-tartaric acid, L-alaninamide and L-serine as the carbon source for growth, and weakly metabolizes N-acetyl-D galactosamine, N-acetyl-D glucosamine, D-fructose, glucose-6-phosphate, fumaric acid, α-hydroxybutyric acid, L-lactic acid, L-malic acid, pyruvic acidmethyl ester, urocanic acid, L-alanine, L-alanyl-L-threonine, L-asparagine, L-valine plus and L-aspartic acid. The DNA G+C content of the type strain NSJ-70^T is 70.44 mol %. The type strain NSJ-70^T (=CGMCC 1.32846^T =KCTC 25139^T) was isolated from the faeces of a healthy adult.

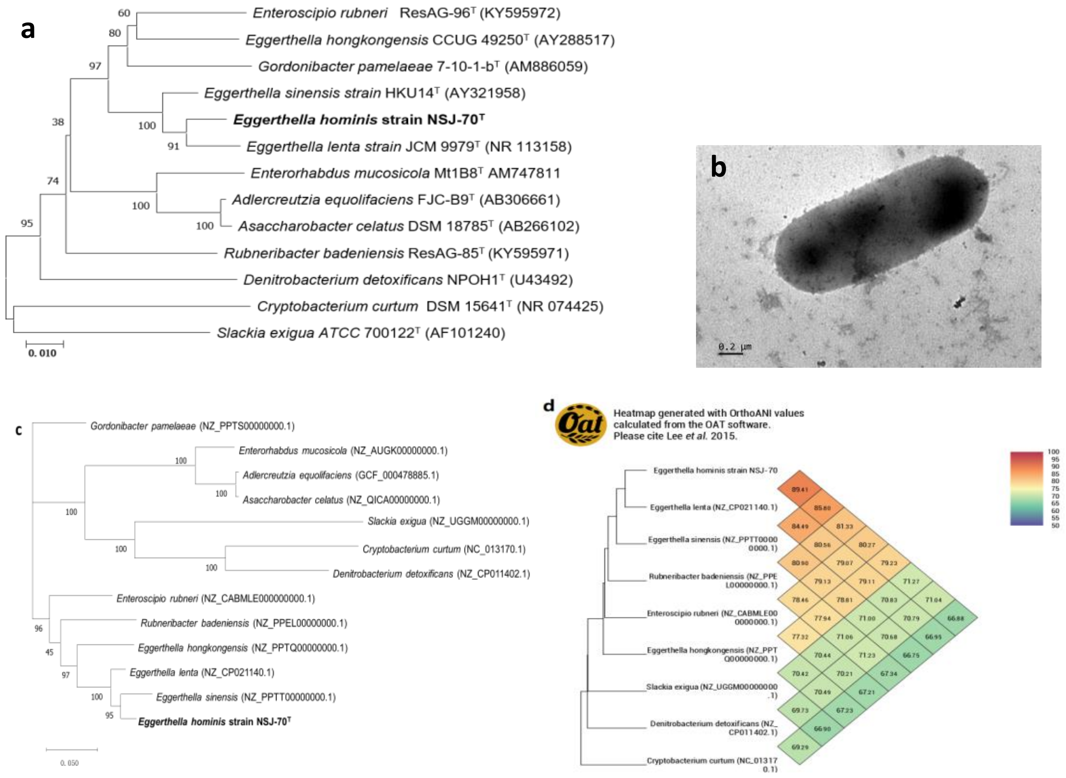

Figure SD-32. The Neighbour-joining phylogenetic tree based on 16S rRNA gene sequences (a) and the cell morphology (b) of strain NSJ-70^{T}. GenBank accession numbers are given in parentheses. Percentages of bootstrap support are shown at branch nodes. Bootstrap value was 1000. Bar: 0.01 substitutions per nucleotide position. The phylogenominc tree based on gemome-sequence-alignment (c) and OrthoANI heatmap (d) of strain NSJ-70^{T} and its neighbor species. Refseq ID of representative genomes are given in parentheses. Percentages of bootstrap support are shown at branch nodes. Bar: 0.050 substitutions per nucleotide position.

Taxon 33: *Gordonibacter massiliensis* sp.

Description of *Gordonibacter massiliensis* sp. (mas.si.li.en'sis. L. adj. masc. *massiliensis*, of Massilia, Marseilli, where the bacterium was for the first time isolated)

The taxon 33, represented by strain NSJ-58^T (16S rRNA sequence accession number in NMDC is NMDCN0000110), is closest to *Gordonibacter lactaris*, with 16S rRNA gene identity of 95.2 %. Phylogenetic tree shows that strain NSJ-58^T clusters with other members of the genus *Gordonibacter* (Figure SD-33a), suggesting that strain NSJ-58^T is a member of the genus *Gordonibacter.* The genome of strain NSJ-58^T was sequenced and the NMDC accession number is NMDC60014032. Genome-based analysis on *Gordonibacter urolithinfaciens* strain DSM 27213^T (NZ LT900218) and type strain NSJ-58^T reveals that the ANI value is 80.88 % and the dDDH estimation is 30.30 %. According to the phylogenominc tree (Figure SD-33c), the ANIs and dDDH values of genomes between strain NSJ-58^T (NMDC accession number is NMDC60014032) and phylogenomically neighbored genomes were calculated, and the highest ANI and dDDH values are 85.92 and 32 %, respectively, between the query genome of *Gordonibacter pamelaeae* (NZ_PPTS00000000.1); and difference in % G+C between genomes of strain NSJ-58^T and *Gordonibacter pamelaeae* (NZ_PPTS00000000.1) is 0.13; OrthoANI heatmap (Figure SD-33d) shows the phylogenomic status of the corresponding neighbor species based on OrthoANI values. Based on these results, we concluded that the strain NSJ-58^T represents a new species of the genus *Gordonibacter*, and the name *Gordonibacter massiliensis* sp.nov. is proposed by Traore. Thus here we present the characterization and identification of the strain to validate the name *Gordonibacter massiliensis*.

Cells are strictly anaerobic, short rods (1.3-2.9 µm long by 0.6-0.8 µm wide, Figure SD-33b); cells appear singly or in dividing pairs. Tiny, semi-translucent, pinpoint colonies appear on GAM agar plate after 3 days of incubation. Growth occurs at range 37 °C and at pH range of 7.0-7.5. The organism uses N-acetyl-D galactosamine, acetyl-D glucosamine, N-acetyl-β-D mannosamine, D-fructose, L-fucose, D-galactose, D-galacturonic acid, glycerol, D, L-α-glycerol phosphate, α-methyl-D-galactoside, ß-methyl-D-galactoside, α-methyl-D glucoside, ß-methyl-D-glucoside, palatinose, L-rhamnose, turanose, fumaric acid, glyoxylic acid, D-malic acid, L-malic acid, pyruvic acid, succinic acid mono-methyl ester, urocanic acid, L-alaninamide, L-alanyl-L-histidine, L-alanyl-L-threonine, L-valine plus and L-aspartic acid as the carbon source for growth, and weakly metabolizes adonitol, glucose-6-phosphate, m-Inositol, 3-melthyl-D-glucose, formic acid, ß-hydroxybutyric acid, succinamic acid, succinic acid, L-glutamic acid, glycyl-L-aspartic acid, L-serine, L-threonine. The DNA G+C content of the type strain NSJ-58^T is 76.43 mol %. The type strain is NSJ-58^T (=CGMCC 1.32834^T =KCTC 25146^T), which was isolated from the faeces of a healthy adult.

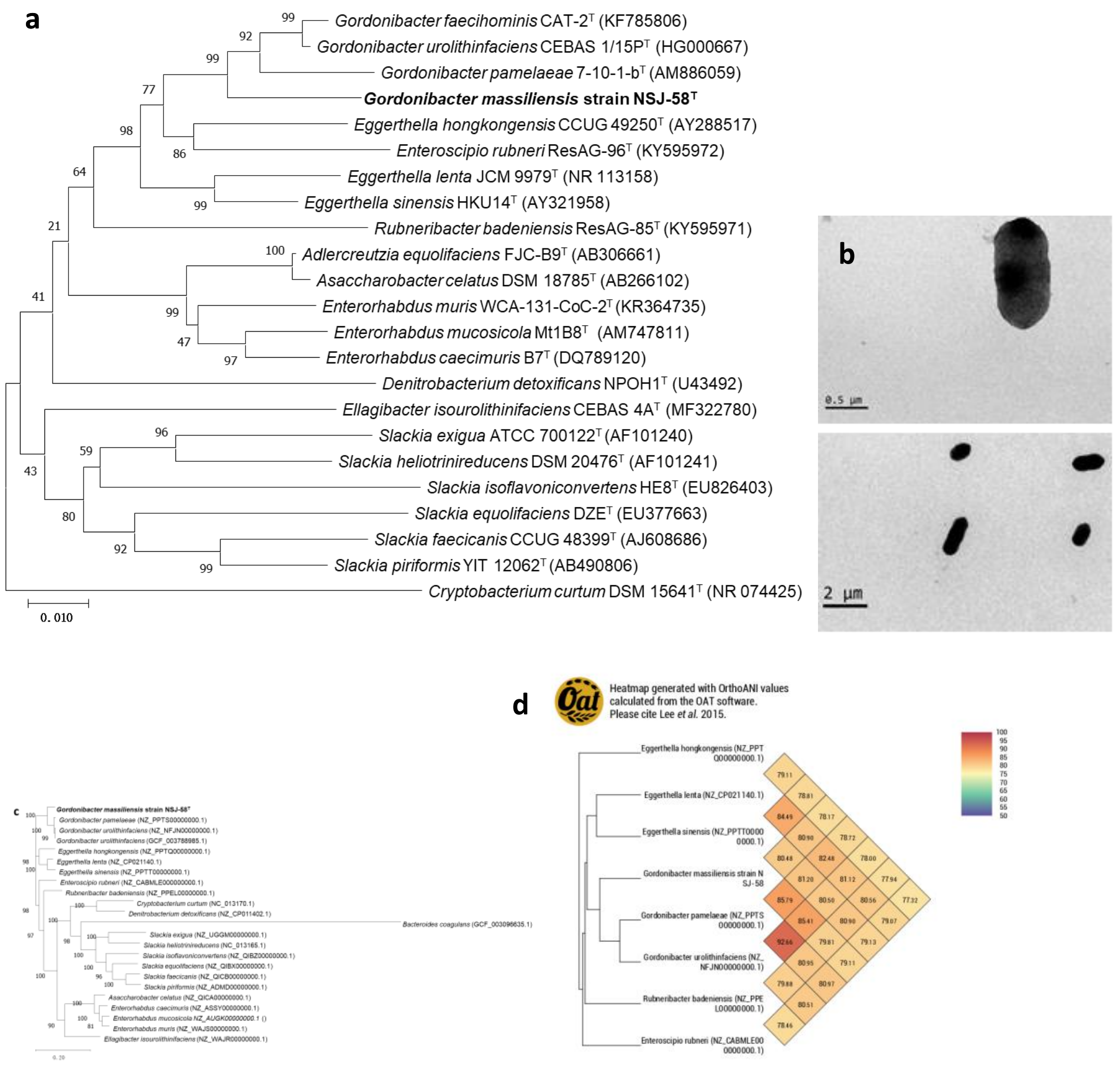

Figure SD-33. The Neighbour-joining phylogenetic tree based on 16S rRNA gene sequences (a) and the cell morphology (b) of strain NSJ-58^{T}. GenBank accession numbers are given in parentheses. Percentages of bootstrap support are shown at branch nodes. Bootstrap value was 1000. Bar: 0.01 substitutions per nucleotide position. The phylogenominc tree based on gemome-sequence-alignment (c) and OrthoANI heatmap (d) of strain NSJ-58^{T} and its neighbor species. Refseq ID of representative genomes are given in parentheses. Percentages of bootstrap support are shown at branch nodes. Bar: 0.20 substitutions per nucleotide position.

Taxon 35: *Bacteroides multiformis* sp. nov.

Description *of Bacteroides multiformis* sp. nov. (mul.ti.for'mis. L. masc. adj. *multiformis*, many-shaped, multiform, referring to the various size and shape of the strain)

The taxon 35, respresented by strain L5^T, is phylogenetically closest to *Bacteroides cellulosilyticus*, with 16S rRNA gene identity of 98.65 %. Phylogenetic tree shows that strain L5^T clusters with other members of the genus *Bacteroides,* (Figure SD-35a), suggesting strain L5^T is a member of the genus *Bacteroides*. The genome of strain L5^T was sequenced and the NMDC accession number is NMDC60014034. Genome-based analysis showed that the ANI value of genomes of strain L5^T and *Bacteroides cellulosilyticus* DSM 14838^T (NZ CP012801) is 89.44 % and the dDDH estimation is 41.20 %. According to the phylogenominc tree (Figure SD-35c), the ANIs and dDDH values of genomes between strain L5^T (NMDC accession number is NMDC60014034) and phylogenomically neighbored genomes were calculated, and the highest ANI and dDDH values are 93.68 and 57.3 %, respectively, between the query genome of *Bacteroides intestinalis* (NZ_ABJL00000000.2); and difference in % G+C between genomes of strain L5^T and *Bacteroides intestinalis* (NZ_ABJL00000000.2) is 0.24; OrthoANI heatmap (Figure SD-35d) shows the phylogenomic status of the corresponding neighbor species based on OrthoANI values. Based on these results, we concluded that the strain L5^T represents of new species of the genus *Bacteroides*, and the name *Bacteroides multiformis* sp. nov. is proposed.

Cells are strictly anaerobic, oval or rods (0.9-3.2 µm long by 0.6-1.3 µm wide, Figure SD-35b); cells appear singly. Tiny, circular, clear, smooth, light whitish, translucent, raised colonies appear on modified mGAM agar plate after 2 days of incubation. Growth occurs at 37°C and at pH of 7.0-7.5. The organism uses arbutin, D-cellobiose, dextrin, D-fructose, L-fucose, D-galactose, D-galacturonic acid, gentiobiose, α-D-glucose, glucose-1-phosphate, glucose-6-phosphate, lactulose, D-mannose, D-melibiose, 3-melthyl-D-glucose, palatinose, L-rhamnose, D-sorbitol, stachyose, sucrose, turanose, acetic acid, glyoxylic acid, α-hydroxybutyric acid, α-ketobutyric acid, α-ketovaleric acid, D, L-lactic acid, L-lactic acid, D-lactic acid methyl ester, D-malic acid, pyruvic acid, pyruvic acid methyl ester, succinamic acid, succinic acid, succinic acid mono-methyl ester, m-tartaric acid, glycyl-L-aspartic acid, glycyl-L-glutamine, glycyl-L-methionine, glycyl-L-proline, inosine, thymidine, uridine and uridine-5'-monophophate as the carbon source for growth, and weakly metabolizes N-acetyl-D galactosamine, amygdalin, α-cyclodextrin, β-cyclodextrin, D-gluconic acid, D-glucosaminic acid, α-D-lactose, maltotriose, D-melezitose, formic acid, ß-hydroxybutyric acid, L-malic acid, propionic acid, D-saccharic acid, L-alanyl-L-histidine, L-glutamic acid, L-glutamine, L-methionine, L-phenylalanine, L-valine plus L-aspartic acid, 2'-deoxy adenosine and thymidine-5'-monophosphate. The DNA G+C content of the type strain L5^T is 56.50 mol %. The type strain L5^T (=CGMCC 1.32865^T) was isolated from the faeces of a healthy adult.

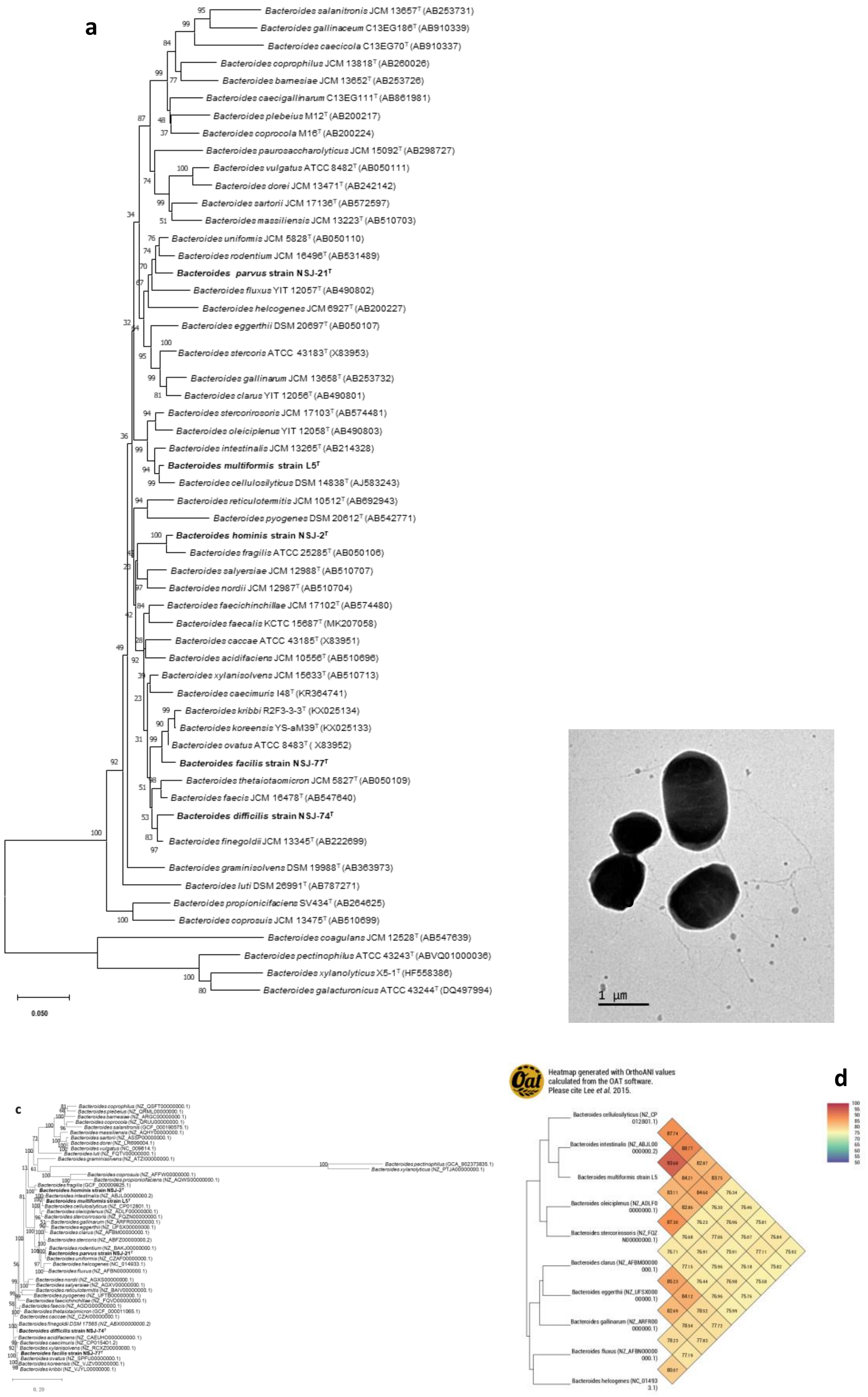

Figure SD-35. The Neighbour-joining phylogenetic tree based on 16S rRNA gene sequences (a) and the cell morphology (b) of strain L5^{T}. GenBank accession

numbers are given in parentheses. Percentages of bootstrap support are shown at branch nodes. Bootstrap value was 1000. Bar: 0.05 substitutions per nucleotide position. The phylogenominc tree based on gemome-sequence-alignment (c) and OrthoANI heatmap (d) of strain L5^{T} and its neighbor species. Refseq ID of representative genomes are given in parentheses. Percentages of bootstrap support are shown at branch nodes. Bar: 0.20 substitutions per nucleotide position.

Taxon 36: *Bacteroides facilis* sp. nov.

Description *of Bacteroides facilis* sp. nov. (L. masc. adj. *facilis*, easy, without difficulty, referring that the type strain is easily cultured)
The taxon 36, respresented by strain NSJ-77^T (16S rRNA sequence accession number in NMDC is NMDCN0000113), is phylogenetically closest to *Bacteroides ovatus*, with 16S rRNA gene identity of 98.24 %. Phylogenetic tree shows that strain NSJ-77^T clusters with other members of the genus *Bacteroides,* (Figure SD-36a), suggesting strain NSJ-77^T is a member of the genus *Bacteroides*. The genome of strain NSJ-77^T was sequenced and the NMDC accession number is NMDC60014035. Genome based analysis showed that the ANI value of genomes of strain NSJ-77^T and *Bacteroides ovatus* ATCC 8483^T (NZ CP012938) is 93.86 % and the dDDH estimation is 40.40 %. According to the phylogenominc tree (Figure SD-36c), the ANIs and dDDH values of genomes between strain NSJ-77^T (NMDC accession number is NMDC60014035) and phylogenomically neighbored genomes were calculated, and the highest ANI and dDDH values are 93.69 and 56.9 %, respectively, between the query genome of *Bacteroides koreensis* (NZ_VJZV00000000.1); and difference in % G+C between genomes of strain NSJ-77^T and *Bacteroides koreensis* (NZ_VJZV00000000.1) is 0.32; OrthoANI heatmap (Figure SD-36d) shows the phylogenomic status of the corresponding neighbor species based on OrthoANI values. Based on these results, we concluded that the strain NSJ-77^T represents of new species of the genus *Bacteroides*, and the name *Bacteroides facilis* sp. nov. is proposed.
Cells are strictly anaerobic, rods in various sizes (1.4-4.1 µm long by 1.0-1.3 µm wide, Figure SD-36b); cells appear singly. Circular to round, cream-colored, moist, entire, convex colonies appear on modified mGAM agar plate after 3 days of incubation. Growth occurs at 37°C and at pH 7.0-7.5. The organism uses D-fructose, L-fucose, D-galacturonic acid, 3-melthyl-D-glucose, palatinose,glyoxylic acid, α-hydroxybutyric acid, α-ketovaleric acid, D,L-lactic acid, L-lactic acid, D-malic acid, L-malic acid, pyruvic acid, succinamic acid, succinic acid and succinic acid mono-methyl ester as the carbon source for growth, and weakly metabolizes D-malic acid, L-malic acid, pyruvic acid, succinamic acid, succinic acid and m-tartaric acid. The DNA G+C content of the type strain NSJ-77^T is 41.74 mol %. The type strain NSJ-77^T (=CGMCC 1.32853^T=KCTC 25155^T) was isolated from the faeces of a healthy adult.

a

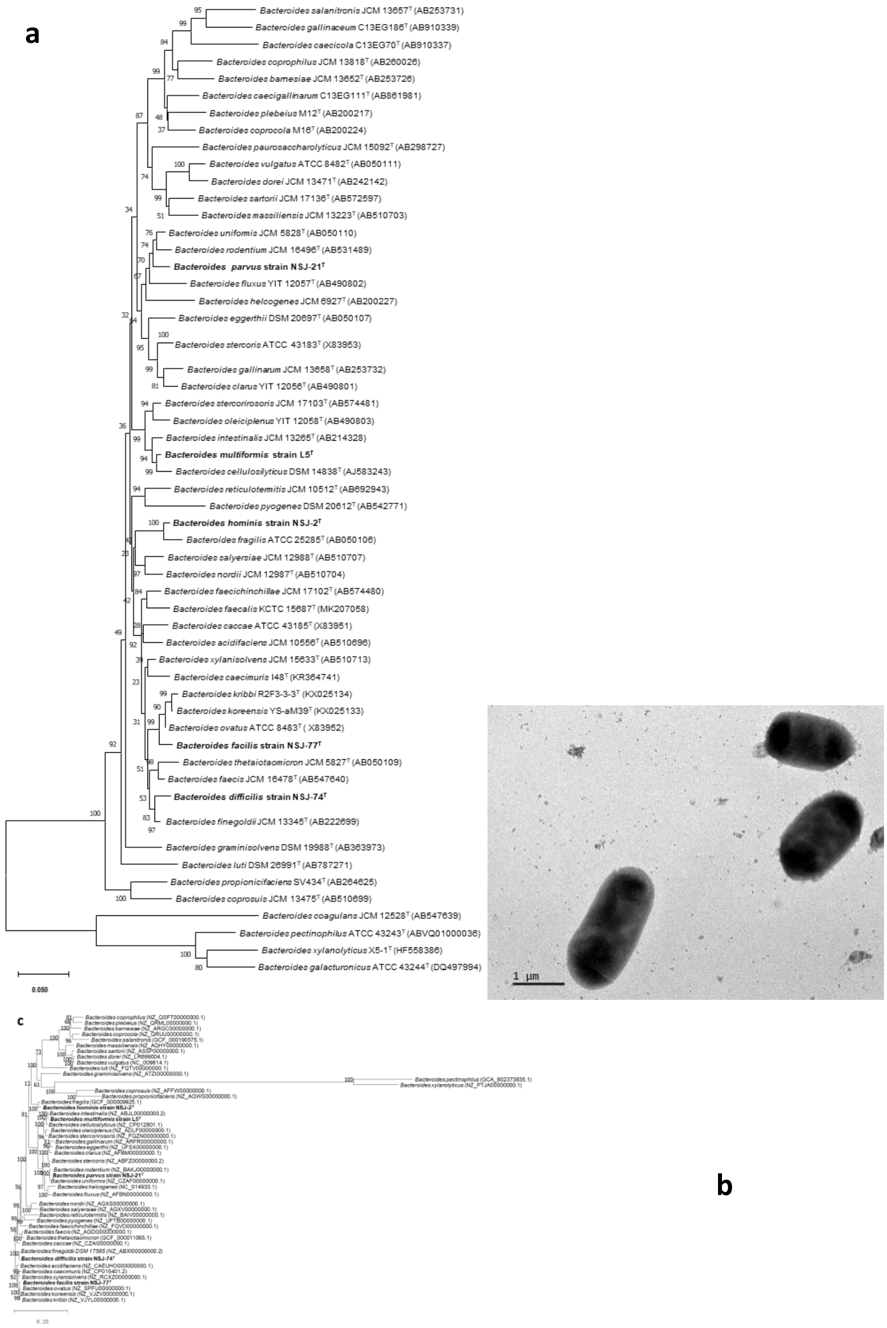

c

b

Figure SD-36. The Neighbour-joining phylogenetic tree based on 16S rRNA gene sequences (a) and the cell morphology (b) of strain NSJ-77T. GenBank accession numbers are given in parentheses. Percentages of bootstrap support are shown at branch nodes. Bootstrap value was 1000. Bar: 0.05 substitutions per nucleotide position. The phylogenominc tree based on gemome-sequence-alignment (c) and OrthoANI heatmap (d) of strain NSJ-77T and its neighbor species. Refseq ID of representative genomes are given in parentheses. Percentages of bootstrap support are shown at branch nodes. Bar: 0.20 substitutions per nucleotide position.

Taxon 38: *Bacteroides difficilis* sp. nov.

Description of *Bacteroides difficilis* sp. nov. (diff'i.cil.is L. masc. adj. *difficilis*, difficult, because it is difficult to cultivate)

The taxon 3, represented by strain NSJ-74^T (16S rRNA sequence accession number in NMDC is NMDCN0000115), is closest to *Bacteroides faecis*, with 16S rRNA gene identity of 97.01 %. Phylogenetic tree shows that strain NSJ-74^T clusters with other members of the genus *Bacteroides* (Figure SD-38a), suggesting that strain NSJ-74^T is a member of the genus *Bacteroides.* The genome of strain NSJ-74^T was sequenced and the NMDC accession number is NMDC60014037. Genome-based analysis on *B. faecis* strain MAJ27^T (NZ AGDG00000000) and type strain NSJ-74^T reveals that the ANI value is 80.88 % and the dDDH estimation is 25.70 %. According to the phylogenominc tree (Figure SD-38c), the ANIs and dDDH values of genomes between strain NSJ-74^T (NMDC accession number is NMDC60014037) and phylogenomically neighbored genomes were calculated, and the highest ANI and dDDH values are 95.22 and 65 %, respectively, between the query genome of *Bacteroides finegoldii* DSM 17565^T (NZ_ABXI00000000.2); and difference in % G+C between genomes of strain NSJ-74^T and *Bacteroides finegoldii* DSM 17565^T (NZ_ABXI00000000.2) is 0.59; OrthoANI heatmap (Figure SD-38d) shows the phylogenomic status of the corresponding neighbor species based on OrthoANI values. Based on these results, we concluded that the strain NSJ-74^T represents a new species of the genus *Bacteroides*, and the name *Bacteroides difficilis* sp. nov. is proposed.

Cells are strictly anaerobic, spherical, oval or short rod shaped (1.1-2.4 µm long by 1.0-1.3 µm wide, Figure SD-38b); cells appear singly or in (dividing) pairs. Tiny, circular to irregular, whitish to semi-translucent, smooth, convex, entire colonies appear on PYG agar plate after 5 days of incubation. Growth occurs at 37°C and at pH 7.0-7.5. The organism uses m-tartaric acid and urocanic acid as carbon source for growth, and weakly metabolizes α-cyclodextrin, ß-cyclodextrin, sucrose, D-trehalose, α-ketovaleric acid, pyruvic acid, glycyl-L glutamine. The DNA G+C content of the type strain NSJ-74^T is 52.58 mol %. The type strain is NSJ-74^T (=CGMCC 1.32850^T =KCTC KCTC 25155^T), which was isolated from the faeces of a healthy adult.

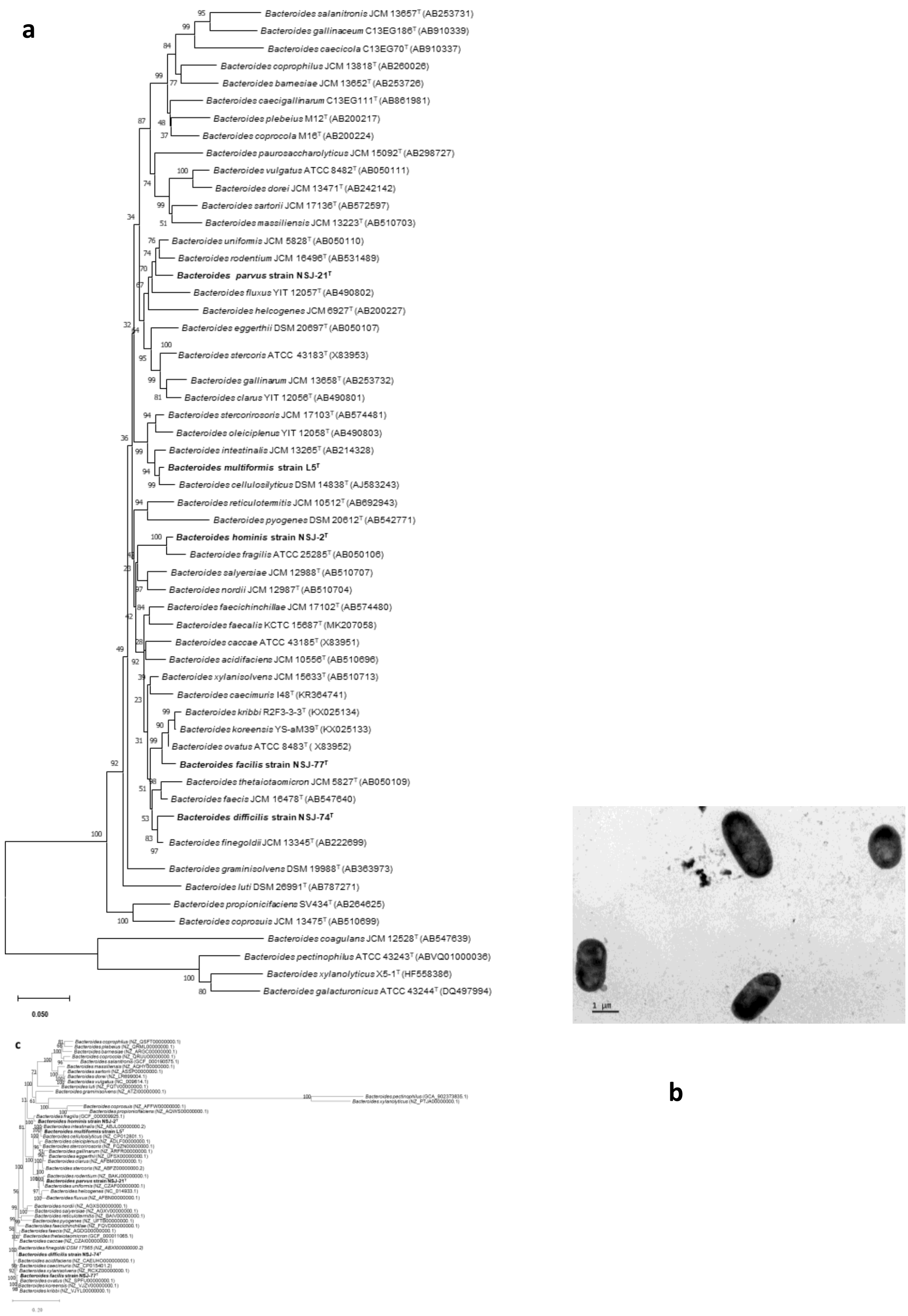

Figure SD-38. The Neighbour-joining phylogenetic tree based on 16S rRNA gene sequences (a) and the cell morphology (b) of strain NSJ-74^{T}. GenBank accession numbers are given in parentheses. Percentages of bootstrap support are shown at branch nodes. Bootstrap value was 1000. Bar: 0.05 substitutions per nucleotide position. The phylogenominc tree based on gemome-sequence-alignment (c) and OrthoANI heatmap (d) of strain NSJ-74^{T} and its neighbor species. Refseq ID of representative genomes are given in parentheses. Percentages of bootstrap support are shown at branch nodes. Bar: 0.20 substitutions per nucleotide position.

Taxon 39: *Bacteroides hominis* sp. nov.

Description of *Bacteroides hominis* sp. nov. (ho’mi.nis L. gen. masc. n. hominis, of a human being, referring to the human gut habitat)

The taxon 39, represented by strain NSJ-2^T (16S rRNA sequence accession number in NMDC is NMDCN0000116), is closest to *Bacteroides fragilis*, with 16S rRNA gene identity of 98.14 %. Phylogenetic tree shows that strain NSJ-2^T clusters with other members of the genus *Bacteroides* (Figure SD-39a), suggesting that strain NSJ-2^T is a member of the genus *Bacteroides.* The genome of strain NSJ-2^T was sequenced and the NMDC accession number is NMDC60014038. Genome-based analysis on *B. fragilis* strain NCTC 9343^T (NC 003228.3) and type strain NSJ-2^T reveals that the ANI value is 86.71 % and the dDDH estimation is 32.2 %. According to the phylogenominc tree (Figure SD-39c), the ANIs and dDDH values of genomes between strain NSJ-2^T (NMDC accession number is NMDC60014038) and phylogenomically neighbored genomes were calculated, and the highest ANI and dDDH values are 86.55 and 20.4 %, respectively, between the query genome of *Bacteroides fragilis* (GCF_000009925.1); and difference in % G+C between genomes of strain NSJ-2^T and *Bacteroides fragilis* (GCF_000009925.1) is 0.81; OrthoANI heatmap (Figure SD-39d) shows the phylogenomic status of the corresponding neighbor species based on OrthoANI values. Based on these results, we concluded that the strain NSJ-2^T represents a new species of the genus *Bacteroides*, and the name *Bacteroides hominis* sp. nov. is proposed.

Cells are strictly anaerobic, spherical, oval or short rod shaped (1.3-2.1 µm long by 1.0-1.2 µm wide, Figure SD-39b); cells appear singly or in (dividing) pairs. Tiny, circular, whitish to semi-translucent, smooth, convex, entire colonies appear on modified mGAM agar plate after 6 days of incubation. Growth occurs at 37°C and at pH 7.0-7.5. The organism uses D-fructose, L-fucose, D-galacturonic acid, palatinose, L-rhamnose, α-hydroxybutyric acid, D, L-lactic acid, L-lactic acid, D-malic acid, succinamic acid, succinic acid and succinic acid mono-methyl ester as the carbon source for growth, and weakly metabolizes arbutin, 3-melthyl-D-glucose, glyoxylic acid, α-ketovaleric acid, pyruvic acid and pyruvic acid methyl ester. The DNA G+C content of the type strain NSJ-2^T is 48.67 mol %. The type strain is NSJ-2^T (=CGMCC 1.31481^T =KCTC 15964^T), which was isolated from the faeces of a healthy adult.

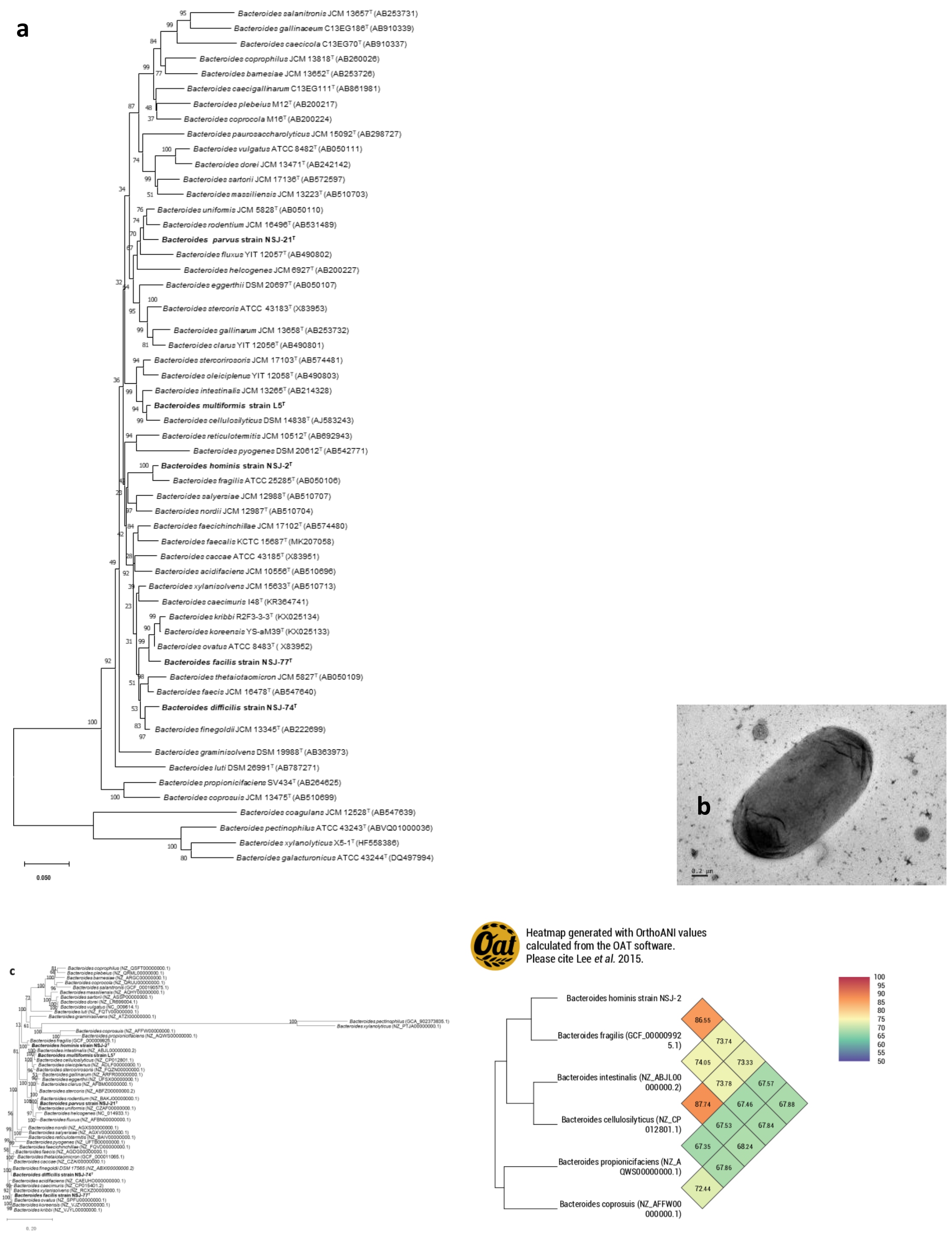

Figure SD-39. The Neighbour-joining phylogenetic tree based on 16S rRNA gene sequences (a) and the cell morphology (b) of strain NSJ-2^T. GenBank accession numbers are given in parentheses. Percentages of bootstrap support are shown at branch nodes. Bootstrap value was 1000. Bar: 0.05 substitutions per nucleotide position. The phylogenominc tree based on gemome-sequence-alignment (c) and OrthoANI heatmap (d) of strain NSJ-2^T and its neighbor species. Refseq ID of representative genomes are given in parentheses. Percentages of bootstrap support are shown at branch nodes. Bar: 0.2 substitutions per nucleotide position.

Taxon 40: *Bacteroides parvus* sp. nov.

Description of *Bacteroides parvus* sp. nov. (par' vus. L. masc. adj. *parvus,* small, referring to the fact that its colonies on modified mGAM agar media are significantly small)

The taxon 40, represented by strain NSJ-21^T (16S rRNA sequence accession number in NMDC is NMDCN0000117), is closest to *Bacteroides uniformis*, with 16S rRNA gene identity of 97.8 %. Phylogenetic tree shows that strain NSJ-21^T clusters with other members of the genus *Bacteroides* (Figure SD-40a), suggesting that strain NSJ-21^T is a member of the genus *Bacteroides.* The genome of strain NSJ-21^T was sequenced and the NMDC accession number is NMDC60014039. Genome-based analysis on *B. uniformis* strain ATCC 8492^T (NZ AAYH00000000) and type strain NSJ-21^T reveals that the ANI value is 95.73 % and the dDDH estimation is 65.80 %. According to the phylogenominc tree (Figure SD-40c), the ANIs and dDDH values of genomes between strain NSJ-21^T (NMDC accession number is NMDC60014039) and phylogenomically neighbored genomes were calculated, and the highest ANI and dDDH values are 95.45 and 65.8 %, respectively, between the query genome of *Bacteroides uniformis* (NZ_CZAF00000000.1); and difference in % G+C between genomes of strain NSJ-21^T and *Bacteroides uniformis* (NZ_CZAF00000000.1) is 0.12; OrthoANI heatmap (Figure SD-40d) shows the phylogenomic status of the corresponding neighbor species based on OrthoANI values. Based on these results, we concluded that the strain NSJ-21^T represents a new species of the genus *Bacteroides*, and the name *Bacteroides parvus* sp. nov. is proposed.

Cells are strictly anaerobic, oval to rod shaped with round or blunt ends (1.1-2.6 µm long by 0.8-1.2 µm wide, Figure SD-40b). Pinpoint or tiny circular, whitish, smooth, convex, entire colonies appear on modified mGAM agar plate after 6 days of incubation. Growth occurs at 37°C and at pH 7.0-7.5. The organism uses arbutin, D-cellobiose, α-cyclodextrin, β-cyclodextrin, dextrin, D-fructose, L-fucose, D-galactose, D-galacturonic acid, gentiobiose, D-gluconic acid, D-glucosaminic acid, α-D-glucose, glucose-1-phosphate, glucose-6-phosphate, α-D-lactose, lactulose, maltose, maltotriose, D-mannose, D-melibiose, 3-melthyl-D-glucose palatinose, D-raffinose, L-rhamnose, stachyose, sucrose, turanose, fumaric acid, glyoxylic acid, α-hydroxybutyric acid, α-ketobutyric acid, α-ketovaleric acid, L-lactic acid, D-lactic acid methyl ester, D-malic acid, L-malic acid, pyruvic acid, pyruvic acid methyl ester, succinamic acid, succinic acid, succinic acid mono-methyl ester, m-tartaric acid, L-alaninamide, L-asparagine, L-alanyl-L-threonine L-alanine, L-alanyl-L-glutamine, L-alanyl-L-histidine, L-serine, L-glutamic acid, L-glutamine, glycyl-L-aspartic acid, glycyl-L-glutamine, glycyl-L-methionine, L-methionine, L-threonine, L-valine, L-valine plus L-aspartic acid, 2'-deoxy adenosine, inosine, thymidine, uridine-5'-monophophate, thymidine-5'-monophosphate and uridine as the carbon source for growth, and weakly N-acetyl-D galactosamine, acetyl-D glucosamine, amygdalin, D-melezitose, α-methyl-D-galactoside, ß-methyl-D-galactoside, ß-methyl-D-glucoside, salicin, D-sorbitol, D-trehalose, L-lactic acid, D-saccharic acid, urocanic acid, L-phenylalanine. The DNA G+C content of the type strain NSJ-21^T is

46.32 mol %. The type strain is NSJ-21^{T} (=CGMCC 1.31612^{T}=KCTC 25073^{T}), which was isolated from the faeces of a healthy adult.

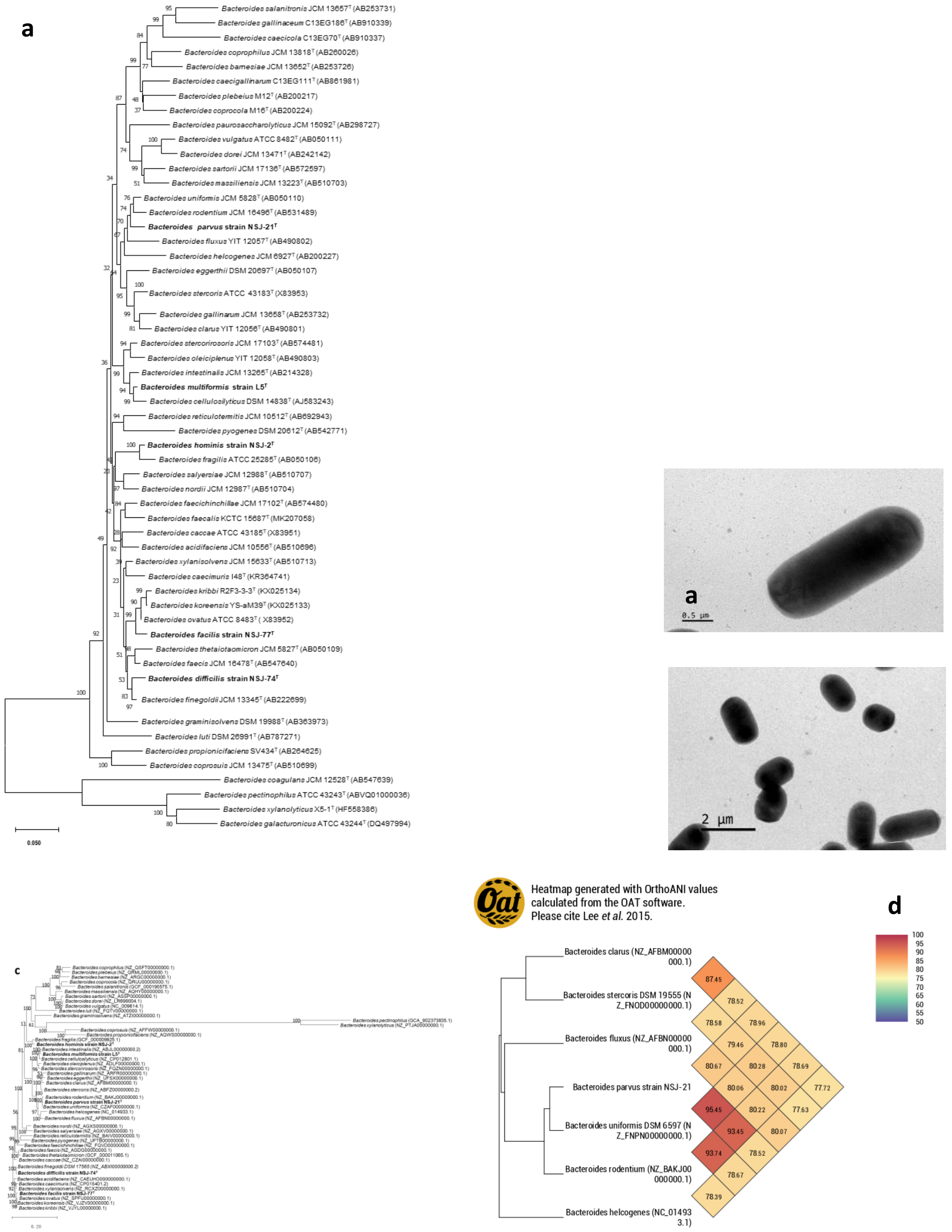

Figure SD-40. The Neighbour-joining phylogenetic tree based on 16S rRNA gene sequences (a) and the cell morphology (b) of strain NSJ-21^T. GenBank accession numbers are given in parentheses. Percentages of bootstrap support are shown at branch nodes. Bootstrap value was 1000. Bar: 0.05 substitutions per nucleotide position. The phylogenomine tree based on gemome-sequence-alignment (c) and OrthoANI heatmap (d) of strain NSJ-21^T and its neighbor species. Refseq ID of representative genomes are given in parentheses. Percentages of bootstrap support are shown at branch nodes. Bar: 0.20 substitutions per nucleotide position.

Taxon 41: *Barnesiella faecis* sp. nov.

Description *of Barnesiella faecis* sp. nov. (L. gen. fem. n. *faecis*, of faeces, from which the organism was isolated)

The taxon 41, respresented by strain BX6^T (16S rRNA sequence accession number in NMDC is NMDCN0000118), is phylogenetically closest to *Barnesiella intestinihominis*, with 16S rRNA gene identity of 98.6 %. Phylogenetic tree shows that strain BX6^T clusters with other members of the genus *Barnesiella* (Figure SD-41a), suggesting strain BX6^T is a member of the genus *Barnesiella.* The genome of strain BX6^T was sequenced and the NMDC accession number is NMDC60014105. Genome-based analysis showed that the ANI value of genomes of strain BX6^T and *Barnesiella intestinihominis* YIT 11860^T (NZ_ADLE00000000) is 61.04 % and the dDDH estimation is 12.90 %. According to the phylogenominc tree (Figure SD-41c), the ANIs and dDDH values of genomes between strain BX6^T (NMDC accession number is NMDC60014105) and phylogenomically neighbored genomes were calculated, and the highest ANI and dDDH values are 63.24 and 17.8 %, respectively, between the query genome of *Paramuribaculum intestinale* (NZ_PUBV00000000.1); and difference in % G+C between genomes of strain BX6^T and *Paramuribaculum intestinale* (NZ_PUBV00000000.1) is 13.01; OrthoANI heatmap (Figure SD-41d) shows the phylogenomic status of the corresponding neighbor species based on OrthoANI values. Based on these results, we concluded that the strain BX6^T (=CGMCC 1.32883^T) represents of new species of the genus *Barnesiella*, and the name *Barnesiella faecis* sp. nov. is proposed.

Cells are strictly anaerobic, straight or slightly curved rods (1.4-2.6 µm long by 0.8-1.2 µm wide, Figure SD-41b); cells appear singly or in dividing pairs. The DNA G+C content of the type strain BX6^T is 66.00 mol %. The type strain is BX6^T (=CGMCC 1.32883^T), which was isolated from the faeces of a healthy adult.

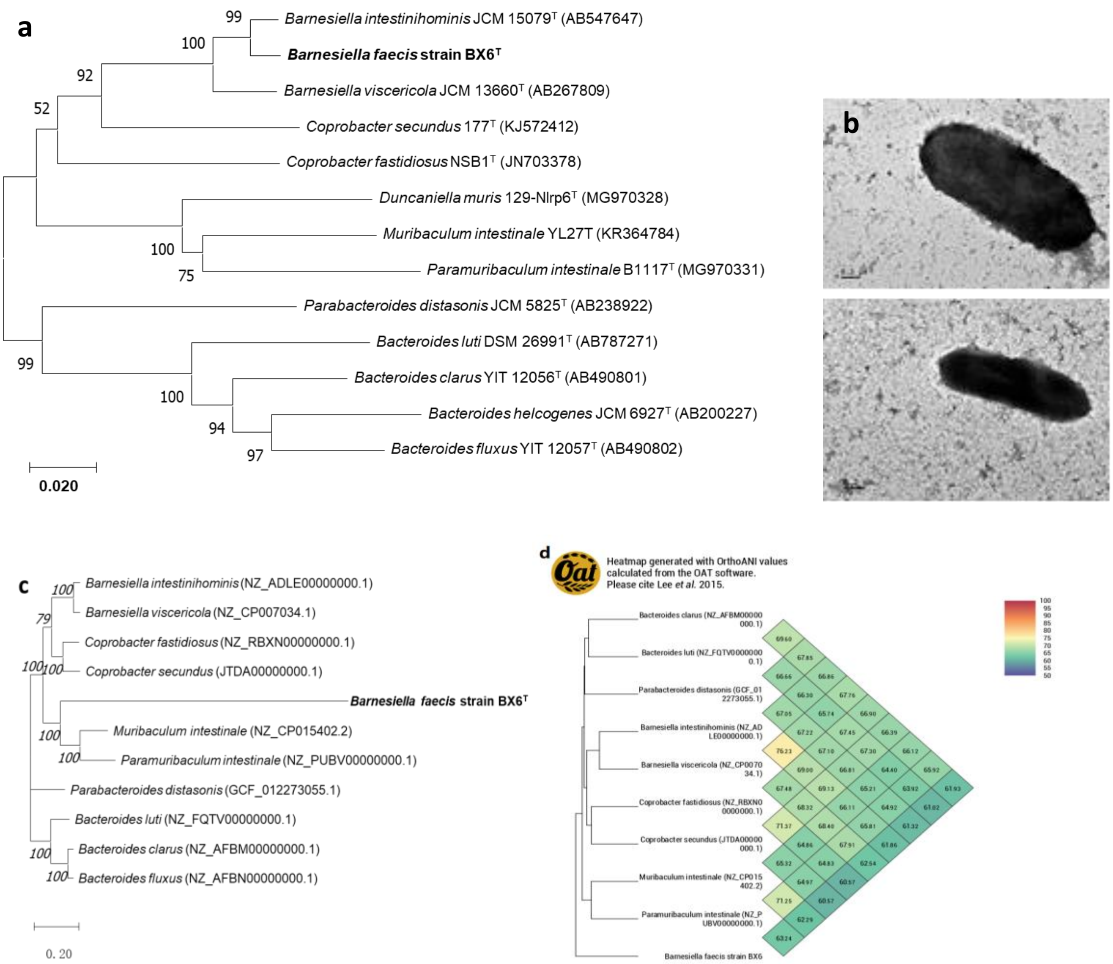

Figure SD-41. The Neighbour-joining phylogenetic tree based on 16S rRNA gene sequences (a) and the cell morphology (b) of strain BX6^{T}. GenBank accession numbers are given in parentheses. Percentages of bootstrap support are shown at branch nodes. Bootstrap value was 1000. Bar: 0.02 substitutions per nucleotide position. The phylogenominc tree based on gemome-sequence-alignment (c) and OrthoANI heatmap (d) of strain BX6^{T} and its neighbor species. Refseq ID of representative genomes are given in parentheses. Percentages of bootstrap support are shown at branch nodes. Bar: 0.20 substitutions per nucleotide position.

Taxon 42: *Butyricimonas hominis* sp. nov.

Description *of Butyricimonas hominis* sp. nov. (ho'mi.nis L. gen. masc. n. hominis, of a human being, referring to the human gut habitat)

The taxon 42, respresented by strain NSJ-56^T (16S rRNA sequence accession number in NMDC is NMDCN0000119), is phylogenetically closest to *Anaerostipes rhamnosivorans*, with 16S rRNA gene identity of 98.1 %. Phylogenetic tree shows that strain NSJ-56^T clusters with other members of the genus *Anaerostipes,* (Figure SD-42a), suggesting strain NSJ-56^T is a member of the genus *Anaerostipes*. The genome of strain NSJ-56^T was sequenced and the NMDC accession number is NMDC60014040. Genome-based analysis showed that the ANI value of genomes of strain NSJ-56^T and *Anaerostipes rhamnosivorans* 1y-2^T (NZ CP040058) is 92.07 % and the dDDH estimation is 48.40 %. According to the phylogenominc tree (Figure SD-42c), the ANIs and dDDH values of genomes between strain NSJ-56^T (NMDC accession number is NMDC60014040) and phylogenomically neighbored genomes were calculated, and the highest ANI and dDDH values are 82.43 and 26.1 %, respectively, between the query genome of *Butyricimonas synergistica* (NZ_ARBK00000000.1); and difference in % G+C between genomes of strain NSJ-56^T and *Butyricimonas synergistica* (NZ_ARBK00000000.1) is 0.35; OrthoANI heatmap (Figure SD-42d) shows the phylogenomic status of the corresponding neighbor species based on OrthoANI values. Based on these results, we concluded that the strain NSJ-56^T represents of new species of the genus *Anaerostipes*, and the name *Anaerostipes hominis* sp. nov. is proposed.

Cells are strictly anaerobic, short rods (1.6–2.4 µm long by 0.8–1.2 µm wide, Figure SD-42b); cells appear singly. Growth occurs at 37°C and at pH 7.0-7.5. The organism uses D-fructose, L-fucose, D-galacturonic acid, palatinose, succinamic acid, succinic acid, succinic acid mono-methyl ester and urocanic acid as the carbon source for growth, and weakly metabolizes D-galactose, α-D-glucose, glucose-6-phosphate, D-mannose, 3-melthyl-D-glucose, L-rhamnose, D-trehalose, glyoxylic acid, L-lactic acid, D-malic acid, pyruvic acid and m-tartaric acid. The DNA G+C content of the type strain NSJ-56^T is 60.54 mol %. The type strain NSJ-56^T (=CGMCC 1.32832^T) was isolated from the faeces of a healthy adult.

a

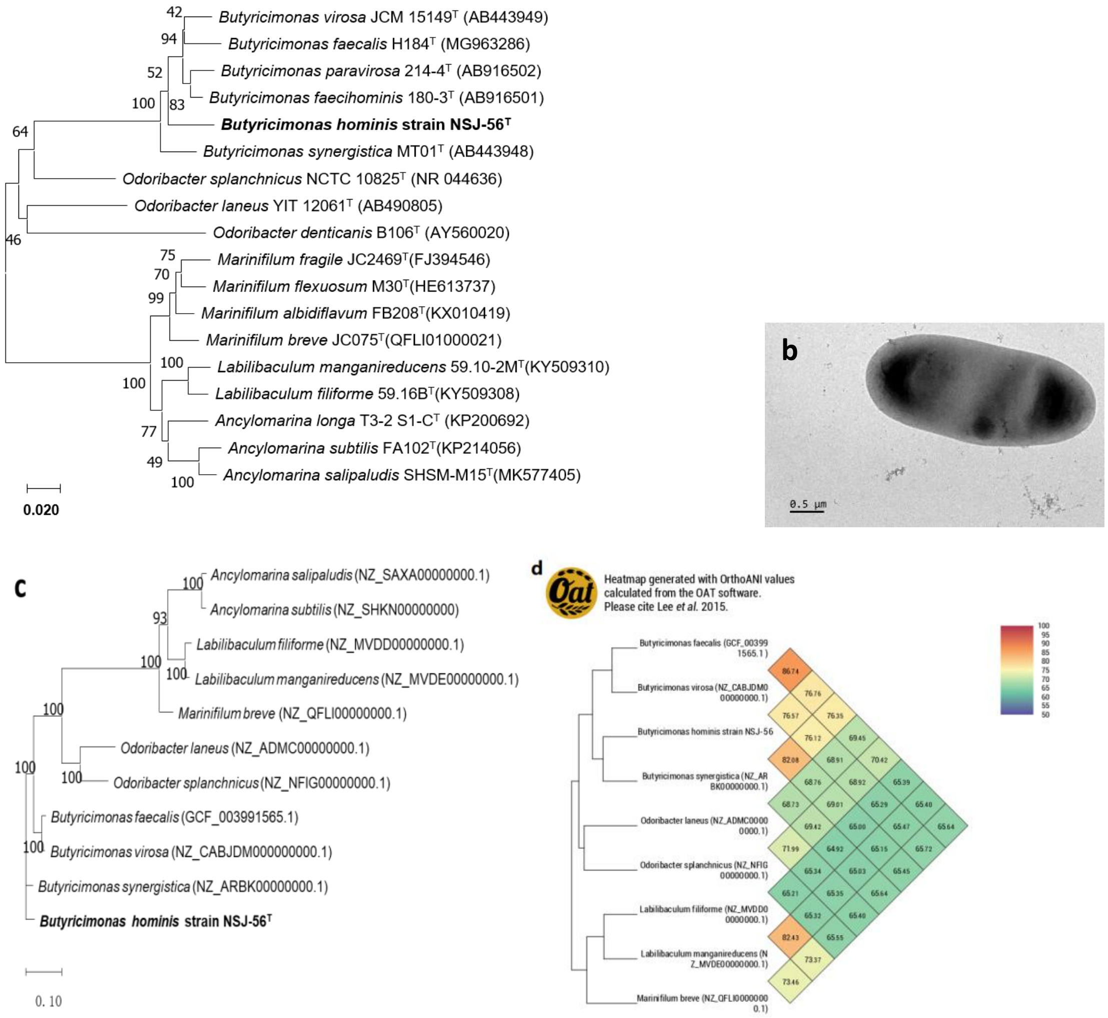

Figure SD-42. The Neighbour-joining phylogenetic tree based on 16S rRNA gene sequences (a) and the cell morphology (b) of strain NSJ-56^{T}. GenBank accession numbers are given in parentheses. Percentages of bootstrap support are shown at branch nodes. Bootstrap value was 1000. Bar: 0.02 substitutions per nucleotide position. The phylogenominc tree based on gemome-sequence-alignment (c) and OrthoANI heatmap (d) of strain NSJ-56^{T} and its neighbor species. Refseq ID of representative genomes are given in parentheses. Percentages of bootstrap support are shown at branch nodes. Bar: 0.10 substitutions per nucleotide position.

Taxon 44: *Parabacteroides segnis* sp. nov.

Description *of Parabacteroides segnis* sp. nov. (L. masc. adj. segnis, slow, inactive, lazy, referring the slow growth of the strain)

The taxon 44, respresented by strain BX2^T (16S rRNA sequence accession number in NMDC is NMDCN000011B), is phylogenetically closest to *Parabacteroides goldsteinii*, with 16S rRNA gene identity of 97.8 %. Phylogenetic tree shows that strain BX2^T clusters with other members of the genus *Parabacteroides* (Figure SD-44a), suggesting strain BX2^T is a member of the genus *Parabacteroides.* The genome of strain BX2^T was sequenced and the NMDC accession number is NMDC60014041. Genome-based analysis showed that the ANI value of genomes of strain BX2^T and *P. merdae* DSM 19448^T (NZ UFTK00000000) is 93.99 % and the dDDH estimation is 62.70 %. According to the phylogenominc tree (Figure SD-44c), the ANIs and dDDH values of genomes between strain BX2^T (NMDC accession number is NMDC60014041) and phylogenomically neighbored genomes were calculated, and the highest ANI and dDDH values are 94.76 and 62.7 %, respectively, between the query genome of *Parabacteroides goldsteinii* (NZ_UYXJ00000000.1); and difference in % G+C between genomes of strain BX2^T and *Parabacteroides goldsteinii* (NZ_UYXJ00000000.1) is 0.37; OrthoANI heatmap (Figure SD-44d) shows the phylogenomic status of the corresponding neighbor species based on OrthoANI values. Based on these results, we concluded that the strain BX2^T represents of new species of the genus *Parabacteroides*, and the name *Parabacteroides segnis* sp. nov. is proposed.

Cells are strictly anaerobic short rods (1.1-2.3 μm long by 0.9-1.2 μm wide, Figure SD-44b); cells appear singly. Growth occurs at 37°C and at pH of 7.0-7.5. The DNA G+C content of the type strain BX2^T is 43.00 mol %. The type strain BX2^T (=CGMCC 1.32884^T) was isolated from the faeces of a healthy adult.

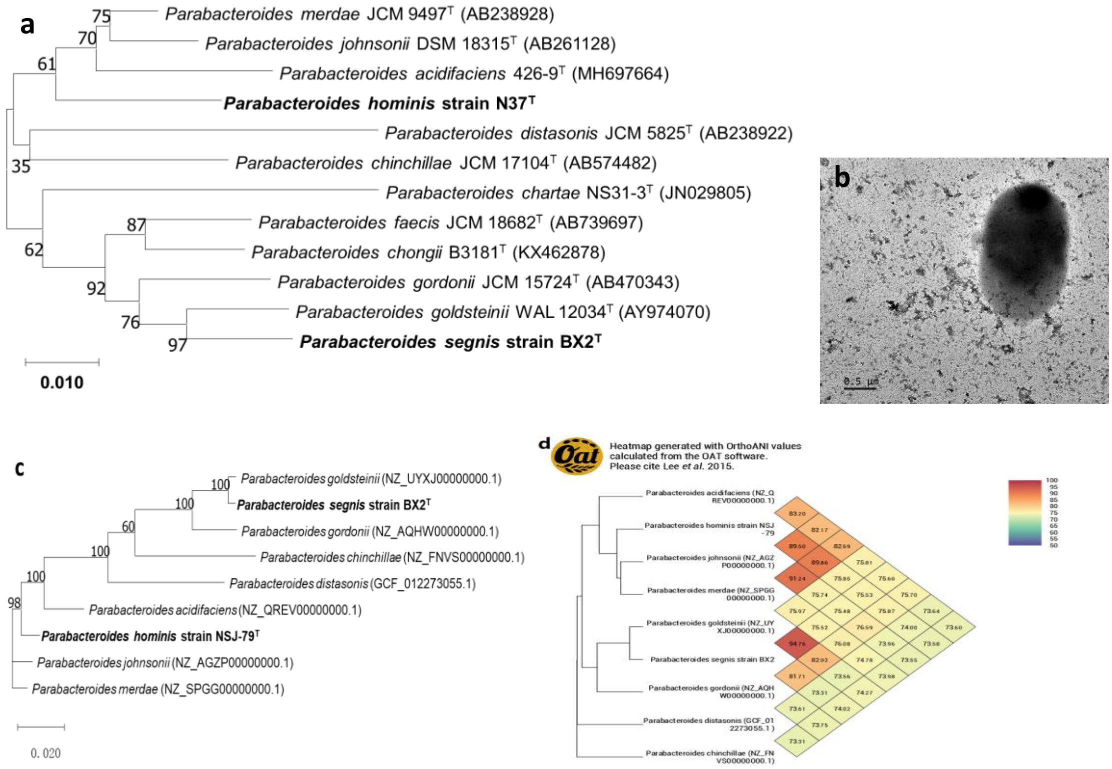

Figure SD-44. The Neighbour-joining phylogenetic tree based on 16S rRNA gene sequences (a) and the cell morphology (b) of strain BX2^T. GenBank accession numbers are given in parentheses. Percentages of bootstrap support are shown at branch nodes. Bootstrap value was 1000. Bar: 0.01 substitutions per nucleotide position. The phylogenominc tree based on gemome-sequence-alignment (c) and OrthoANI heatmap (d) of strain BX2^T and its neighbor species. Refseq ID of representative genomes are given in parentheses. Percentages of bootstrap support are shown at branch nodes. Bar: 0.020 substitutions per nucleotide position.

Taxon 45: *Parabacteroides hominis* sp. nov.

Description *of Parabacteroides hominis* sp. nov. (ho'mi.nis L. gen. masc. n. hominis, of a human being, referring to the human gut habitat)

The taxon 45, respresented by strain NSJ-79^T (16S rRNA sequence accession number in NMDC is NMDCN000011C), is phylogenetically closest to *Parabacteroides merdae*, with 16S rRNA gene identity of 96.4 %. Phylogenetic tree shows that strain NSJ-79^T clusters with other members of the genus *Parabacteroides* (Figure SD-45a), suggesting strain NSJ-79^T is a member of the genus *Parabacteroides.* The genome of strain NSJ-79^T was sequenced and the NMDC accession number is NMDC60014042. Genome-based analysis showed that the ANI value of genomes of strain NSJ-79^T and *P. merdae* ATCC 43184^T (NZ UFTK00000000) is 89.91 % and the dDDH estimation is 40.40 %. According to the phylogenominc tree (Figure SD-45c), the ANIs and dDDH values of genomes between strain NSJ-79^T (NMDC accession number is NMDC60014042) and phylogenomically neighbored genomes were calculated, and the highest ANI and dDDH values are 89.86 and 40.1 %, respectively, between the query genome of *Parabacteroides merdae* (NZ_SPGG00000000.1); and difference in % G+C between genomes of strain NSJ-79^T and *Parabacteroides merdae* (NZ_SPGG00000000.1) is 0.92; OrthoANI heatmap (Figure SD-45d) shows the phylogenomic status of the corresponding neighbor species based on OrthoANI values. Based on these results, we concluded that the strain NSJ-79^T represents of new species of the genus *Parabacteroides*, and the name *Parabacteroides hominis* sp. nov. is proposed.

Cells are strictly anaerobic, short rods (1.1-2.1 µm long by 0.6-0.8 µm wide, Figure SD-45b); cells appear singly or in (dividing) pairs, cell size varies. Round, whitish, glossy, convex colonies appear on modified mGAM agar plate after 3 days of incubation. Growth occurs at 37°C and at pH of 7.0-7.5. The organism uses D-galacturonic acid, L-fucose, palatinose, L-rhamnose, D, L-lactic acid, D-malic acid, succinamic acid, succinic acid and succinic acid mono-methyl ester as the carbon source for growth, and weakly metabolizes D-fructose, 3-melthyl-D-glucose, α-hydroxybutyric acid, L-lactic acid, D-lactic acid methyl ester, pyruvic acid, pyruvic acid methyl ester. The DNA G+C content of the type strain NSJ-79^T is 59.37 mol %. The type strain NSJ-79^T (=CGMCC 1.32855^T =KCTC 25129^T) was isolated from the faeces of a healthy adult.

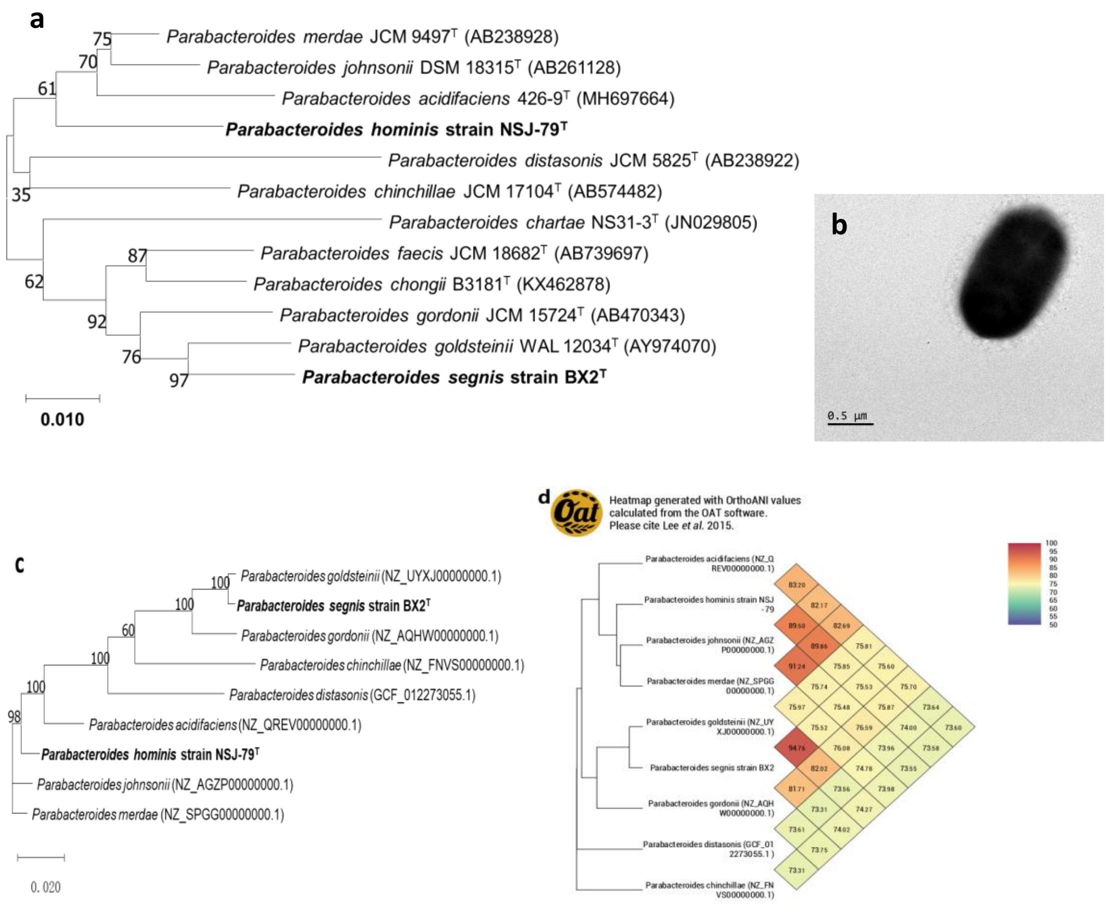

Figure SD-45. The Neighbour-joining phylogenetic tree based on 16S rRNA gene sequences (a) and the cell morphology (b) of strain NSJ-79^T. GenBank accession numbers are given in parentheses. Percentages of bootstrap support are shown at branch nodes. Bootstrap value was 1000. Bar: 0.01 substitutions per nucleotide position. The phylogenominc tree based on gemome-sequence-alignment (c) and OrthoANI heatmap (d) of strain NSJ-79^T and its neighbor species. Refseq ID of representative genomes are given in parentheses. Percentages of bootstrap support are shown at branch nodes. Bar: 0.020 substitutions per nucleotide position.

Taxon 46: *Alistipes hominis* sp. nov.

Description of *Alistipes hominis* sp. nov. (ho'mi.nis L. gen. masc. n. hominis, of a human being, referring to the human gut habitat)

The taxon 46, represented by strain New-7^T (16S rRNA sequence accession number in NMDC is NMDCN000011D), is closest to *Alistipes indistinctus*, with 16S rRNA gene identity of 96.13 %. Phylogenetic tree shows that strain New-7^T clusters with other members of the genus *Alistipes* (Figure SD-46a), suggesting that strain New-7^T is a member of the genus *Alistipes.* The genome of strain New-7^T was sequenced and the NMDC accession number is NMDC60014043. Genome-based analysis on *A. indistinctus* strain YIT 12060^T (NZ ADLD00000000) and type strain New-7^T reveals that the ANI value is 75.0 % and the dDDH estimation is 21.0 %. According to the phylogenominc tree (Figure SD-46c), the ANIs and dDDH values of genomes between strain New-7^T (NMDC accession number is NMDC60014043) and phylogenomically neighbored genomes were calculated, and the highest ANI and dDDH values are 75.778 and 20.4 %, respectively, between the query genome of *Alistipes ihumii* (NZ_CAPH00000000.1); and difference in % G+C between genomes of strain New-7^T and *Alistipes ihumii* (NZ_CAPH00000000.1) is 3.36; OrthoANI heatmap (Figure SD-46d) shows the phylogenomic status of the corresponding neighbor species based on OrthoANI values. Based on these results, we concluded that the strain New-7^T represents a new species of the genus *Alistipes*, and the name *Alistipes hominis* sp. nov. is proposed.

Cells are strictly anaerobic, ovoid to short rods in shape (1.2-1.8 µm long by 0.6-0.8 µm wide, Figure SD-46b). Tiny, cream-colored, smooth, raised pinpoint colonies appear on modified mGAM agar plate after 6-20 days of incubation at 37°C. Growth occurs at 37°C and at pH 7.0-7.5. The organism uses N-acetyl-D galactosamine, acetyl-D glucosamine, amygdalin, D-cellobiose, dextrin, D-fructose, D-galactose, D-galacturonic acid, α-D-glucose, glucose-6-phosphate, lactulose, maltose, maltotriose, D-mannose, D-melezitose, D-melibiose, 3-melthyl-D-glucose, α-methyl-D-galactoside, ß-methyl-D-galactoside, α-methyl-D glucoside, palatinose, D-raffinose and L-rhamnose as the carbon source for growth, and weakly metabolizes N-acetyl-β-D mannosamine, L-fucose, glucose-1-phosphate, a-D-lactose, D-sorbitol, stachyose, fumaric acid and pyruvic acid. The DNA G+C content of the type strain is 58.63 mol %. The type strain is New-7^T (=CGMCC 1.31637^T=KCTC 15866^T), which was isolated from the faeces of a healthy adult.

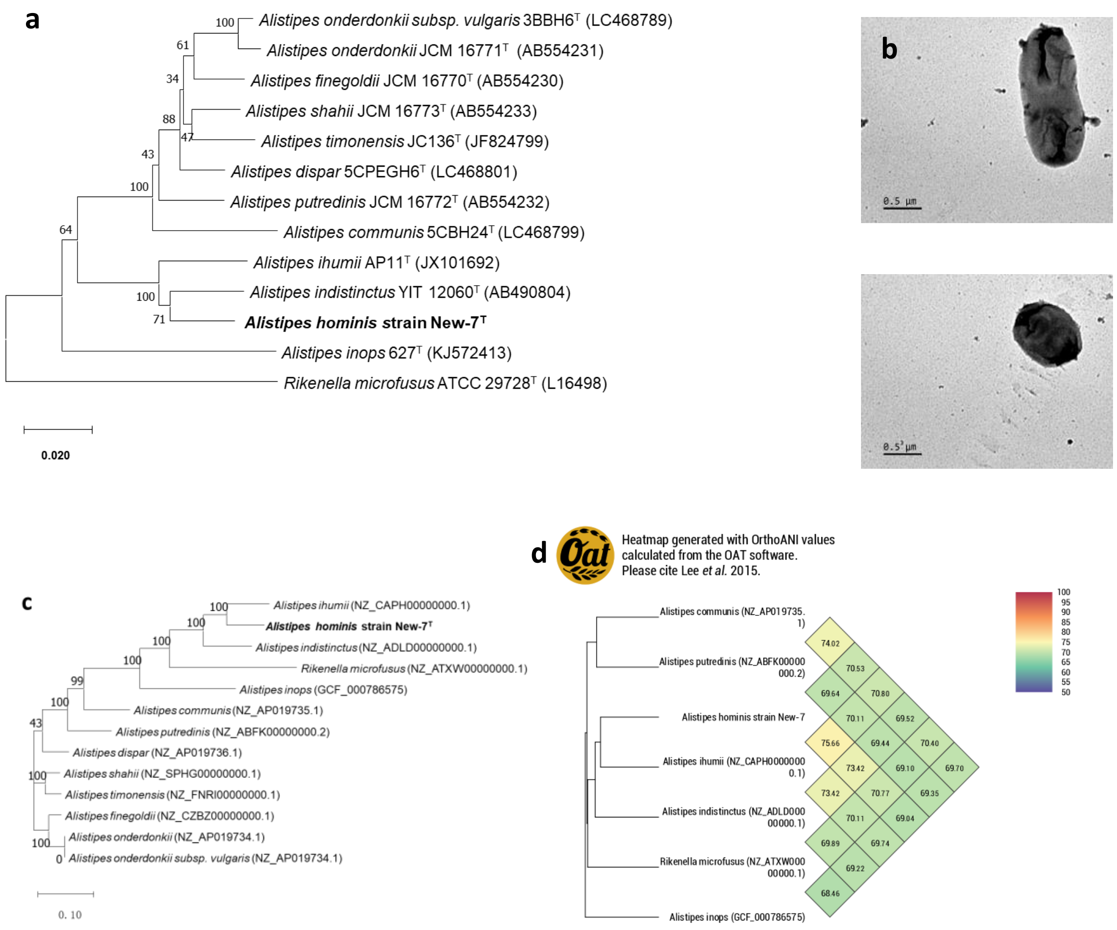

Figure SD-46. The Neighbour-joining phylogenetic tree based on 16S rRNA gene sequences (a) and the cell morphology (b) of strain New-7^T. GenBank accession numbers are given in parentheses. Percentages of bootstrap support are shown at branch nodes. Bootstrap value was 1000. Bar: 0.02 substitutions per nucleotide position. The phylogenominc tree based on gemome-sequence-alignment (c) and OrthoANI heatmap (d) of strain New-7^T and its neighbor species. Refseq ID of representative genomes are given in parentheses. Percentages of bootstrap support are shown at branch nodes. Bar: 0.10 substitutions per nucleotide position.

Taxon 47: *Ornithinibacillus hominis* sp. nov.

Description *of Ornithinibacillus hominis* sp. nov. (ho'mi.nis L. gen. masc. n. hominis, of a human being, referring to the human gut habitat)

The taxon 47, respresented by strain BX22^T (16S rRNA sequence accession number in NMDC is NMDCN000011E), is phylogenetically closest to *Ornithinibacillus scapharcae*, with 16S rRNA gene identity of 98.6 %. Phylogenetic tree shows that strain BX22^T clusters with other members of the genus *Ornithinibacillus* (Figure SD-47a), suggesting strain BX22^T is a member of the genus *Ornithinibacillus.* The genome of strain BX22^T was sequenced and the NMDC accession number is NMDC60014044. Genome-based analysis showed that the ANI value of genomes of strain BX22^T and *Ornithinibacillus scapharcae* TW25^T (NZ AEWH00000000) is 74.47 % and the dDDH estimation is 19.70 %. According to the phylogenominc tree (Figure SD-47c), the ANIs and dDDH values of genomes between strain BX22^T (NMDC accession number is NMDC60014044) and phylogenomically neighbored genomes were calculated, and the highest ANI and dDDH values are 74.99 and 19.7 %, respectively, between the query genome of *Ornithinibacillus scapharcae* (NZ_AEWH00000000.1); and difference in % G+C between genomes of strain BX22^T and *Ornithinibacillus scapharcae* (NZ_AEWH00000000.1) is 0.37; OrthoANI heatmap (Figure SD-47d) shows the phylogenomic status of the corresponding neighbor species based on OrthoANI values. Based on these results, we concluded that the strain BX22^T represents of new species of the genus *Ornithinibacillus*, and the name *Ornithinibacillus hominis* sp. nov. is proposed.

Cells are strictly anaerobic, straight or slightly curved rods (1.2-2.8 µm long by 0.6-1.0 µm wide, Figure SD-47b); cells appear singly or in dividing pairs, cell size varies. Growth occurs at 37°C and at pH of 7.0-7.5.DNA G+C content of the type strain BX22^T is 37.10 mol %. The type strain BX22^T (=CGMCC 1.32885^T) was isolated from the faeces of a healthy adult.

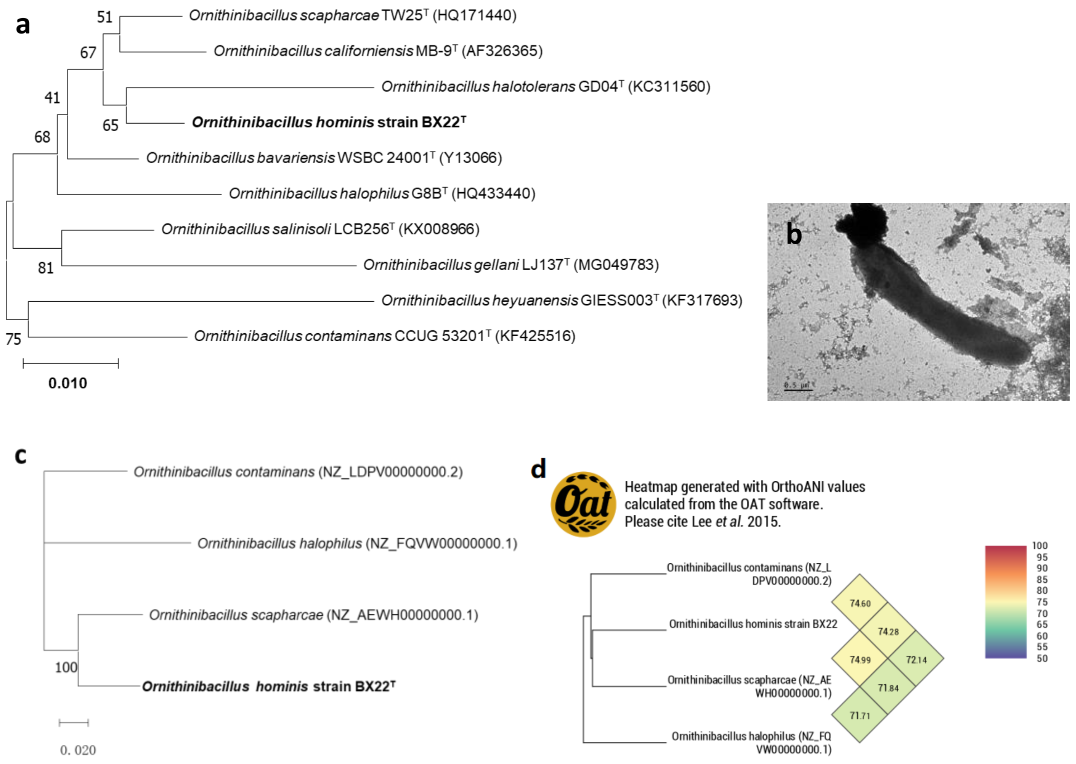

Figure SD-47. The Neighbour-joining phylogenetic tree based on 16S rRNA gene sequences (a) and the cell morphology (b) of strain BX22^T. GenBank accession numbers are given in parentheses. Percentages of bootstrap support are shown at branch nodes. Bootstrap value was 1000. Bar: 0.01 substitutions per nucleotide position. The phylogenominc tree based on gemome-sequence-alignment (c) and OrthoANI heatmap (d) of strain BX22^T and its neighbor species. Refseq ID of representative genomes are given in parentheses. Percentages of bootstrap support are shown at branch nodes. Bar: 0.020 substitutions per nucleotide position.

Taxon 48: *Streptococcus hominis* sp. nov.

Description *of Streptococcus hominis* sp. nov. (ho'mi.nis L. gen. masc. n. hominis, of a human being, referring to the human gut habitat)

The taxon 48, respresented by strain NSJ-17^T (16S rRNA sequence accession number in NMDC is NMDCN000011F), is phylogenetically closest to *Streptococcus anginosus subsp. anginosus*, with 16S rRNA gene identity of 98.3 %. Phylogenetic tree shows that strain NSJ-17^T clusters with other members of the genus *Streptococcus* (Figure SD-48a), suggesting strain NSJ-17^T is a member of the genus *Streptococcus.* The genome of strain NSJ-17^T was sequenced and the NMDC accession number is NMDC60014045. Genome-based analysis showed that the ANI value of genomes of strain NSJ-17^T and *Streptococcus anginosus subsp. anginosus* DSM 20563^T(NC 022239) is 94.31 % and the dDDH estimation is 57.50 %. According to the phylogenominc tree (Figure SD-48c), the ANIs and dDDH values of genomes between strain NSJ-17^T (NMDC accession number is NMDC60014045) and phylogenomically neighbored genomes were calculated, and the highest ANI and dDDH values are 94.38 and 57.6 %, respectively, between the query genome of *Streptococcus anginosus* (NZ_CP012805.1); and difference in % G+C between genomes of strain NSJ-17^T and *Streptococcus anginosus* (NZ_CP012805.1) is 0.07; OrthoANI heatmap (Figure SD-48d) shows the phylogenomic status of the corresponding neighbor species based on OrthoANI values. Based on these results, we concluded that the strain NSJ-17^T represents of new species of the genus *Streptococcus*, and the name *Streptococcus hominis* sp. nov. is proposed

Cells are strictly anaerobic, oval to short rods (0.9-1.7 µm long by 0.7-1.0 µm wide,Figure SD-48b); cells appear singly, in dividing pairs or in short chains. Pinpoint, yellow to whitish, translucent, raised, colonies appear on modified mGAM agar plate after 3 days of incubation. Growth occurs at 37°C and at pH of 7.0-7.5. The organism uses N-acetyl-D galactosamine, acetyl-D glucosamine, N-acetyl-β-D mannosamine, amygdalin, arbutin, D-cellobiose, α-cyclodextrin, β-cyclodextrin, dextrin, D-fructose, L-fucose, D-galactose, D-galacturonic acid, gentiobiose, D-gluconic acid, D-glucosaminic acid, α-D-glucose, glucose-1-phosphate, glucose-6-phosphate, glycerol, m-Inositol, α-D-lactose, lactulose, maltose, maltotriose, D-mannose, D-melezitose, D-melibiose, 3-melthyl-D-glucose, α-methyl-D-galactoside, ß-methyl-D-galactoside, α-methyl-D glucoside, ß-methyl-D-glucoside, palatinose, D-raffinose, L-rhamnose, salicin, D-sorbitol, stachyose, sucrose, D-trehalose, turanose, acetic acid, formic acid, glyoxylic acid, α-ketobutyric acid, α-ketovaleric acid, L-lactic acid, D-lactic acid methyl ester, propionic acid, pyruvic acid methyl ester, m-tartaric acid, L-methionine, L-phenylalanine, L-serine, 2'-deoxy adenosine, inosine, thymidine, uridine and uridine-5'-monophophate as the carbon source for growth, and weakly metabolizes glycyl-L-methionine and thymidine-5'-monophosphate. The DNA G+C content of the type strain NSJ-17^T is 49.41 mol %. The type strain NSJ-17^T (=CGMCC 1.32470^T =KCTC 15949^T) was isolated from the faeces of a healthy adult.

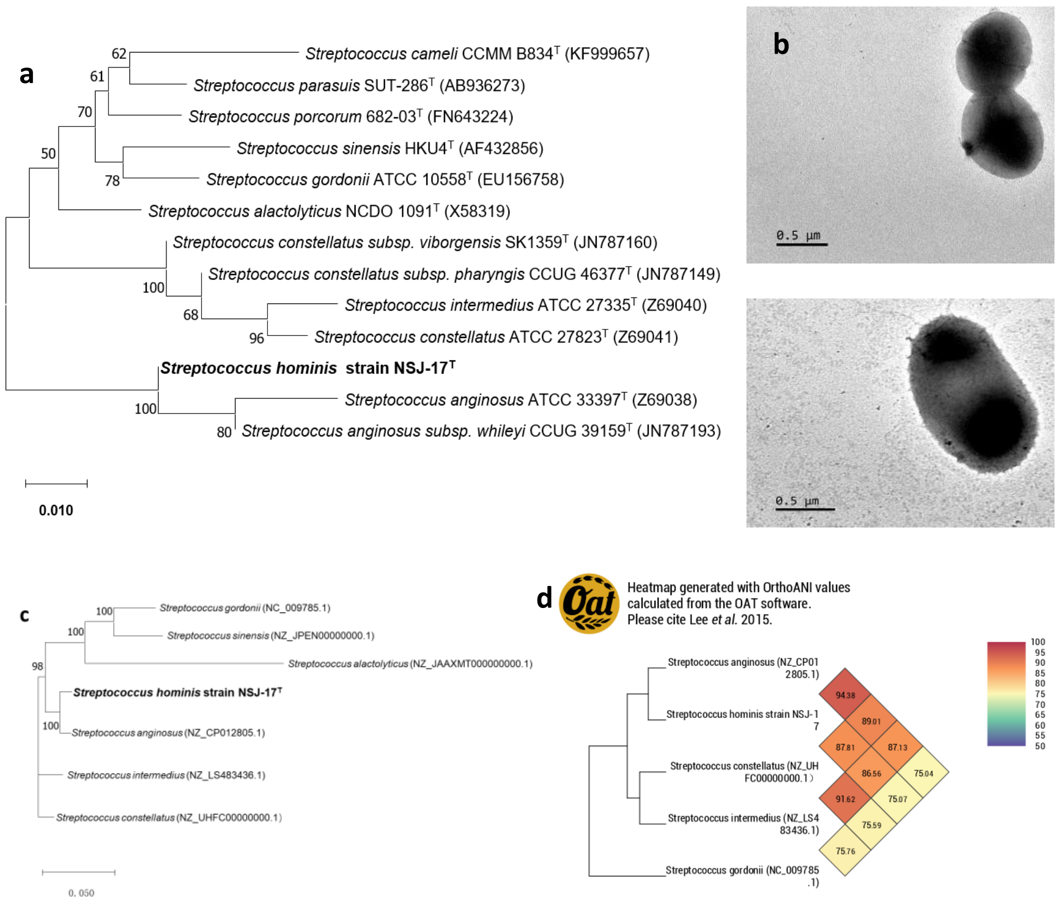

Figure SD-48. The Neighbour-joining phylogenetic tree based on 16S rRNA gene sequences (a) and the cell morphology (b) of strain NSJ-17^{T}. GenBank accession numbers are given in parentheses. Percentages of bootstrap support are shown at branch nodes. Bootstrap value was 1000. Bar: 0.01 substitutions per nucleotide position. The phylogenominc tree based on gemome-sequence-alignment (c) and OrthoANI heatmap (d) of strain NSJ-17^{T} and its neighbor species. Refseq ID of representative genomes are given in parentheses. Percentages of bootstrap support are shown at branch nodes. Bar: 0.050 substitutions per nucleotide position.

Taxon 50: *Christensenella tenuis* sp. nov.

Description *of Christensenella tenuis* sp. nov.

Christensenella tenuis sp. nov. (te'nu.is L. masc./fem. adj. *tenuis*, thin, slim, referring to the predicted potential function of the strain in weight-loss.)

The taxon 50, respresented by strain NSJ-35^T (16S rRNA sequence accession number in NMDC is NMDCN000011H), is phylogenetically closest to *Christensenella minuta*, with 16S rRNA gene identity of 97.5 %. Phylogenetic tree shows that strain NSJ-35^T clusters with other members of the genus *Christensenella,* (Figure SD-50a), suggesting strain NSJ-35^T is a member of the genus *Christensenella*. The genome of strain NSJ-35^T was sequenced and the NMDC accession number is NMDC60014047. Genome-based analysis showed that the ANI value of genomes of strain NSJ-35^T and *Christensenella minuta* DSM 22607^T (NZ LWGY00000000) is 83.25 % and the dDDH estimation is 27.40 %. According to the phylogenominc tree (Figure SD-50c), the ANIs and dDDH values of genomes between strain NSJ-35^T (NMDC accession number is NMDC60014047) and phylogenomically neighbored genomes were calculated, and the highest ANI and dDDH values are 83.22 and 27.2 %, respectively, between the query genome of *Christensenella minuta* (NZ_CP029256.1); and difference in % G+C between genomes of strain NSJ-35^T and *Christensenella minuta* (NZ_CP029256.1) is 2.58; OrthoANI heatmap (Figure SD-50d) shows the phylogenomic status of the corresponding neighbor species based on OrthoANI values.Based on these results, we concluded that the strain NSJ-35^T represents of new species of the genus *Christensenella*, and the name *Christensenella tenuis* sp. nov. is proposed.

Cells are strictly anaerobic, rod-shaped with spiky ends (1.2-1.6 µm long by 0.6-0.8 µm wide); cells appear singly or in (dividing) pairs. Circular, cream-colored, pinpoint, moist, convex colonies appear on modified mGAM agar plate after 2 days of incubation. Growth occurs at 37°C and at pH of 7.0-7.5. The organism uses amygdalin, arbutin, dextrin, D-fructose, L-fucose, gentiobiose, D-mannose, palatinose, sucrose, turanose and inosine as the carbon source for growth, and weakly metabolizes β-cyclodextrin, D-galacturonic acid, α-D-glucose, glucose-6-phosphate, α-D-lactose, maltotriose, ß-methyl-D-glucoside and thymidine. The DNA G+C content of the type strain NSJ-35^T is 55.54 mol %. The type strain NSJ-35^T (=CGMCC 1.32808^T) was isolated from the faeces of a healthy adult.

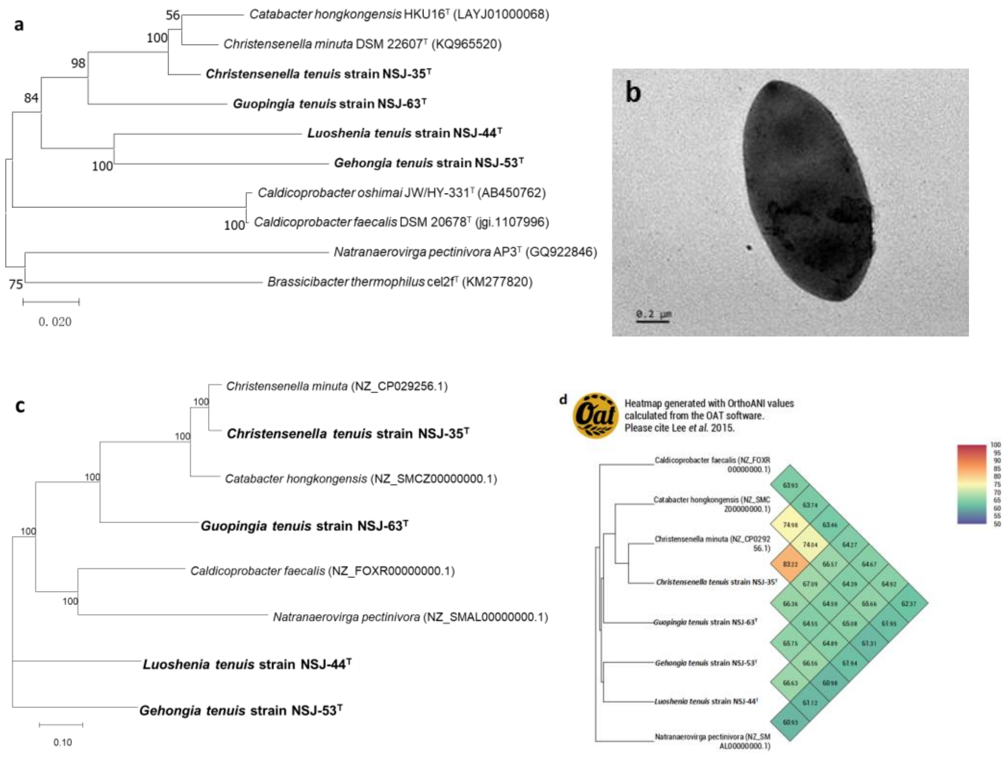

Figure SD-50. The Neighbour-joining phylogenetic tree based on 16S rRNA gene sequences (a) and the cell morphology (b) of strain NSJ-35^T. GenBank accession numbers are given in parentheses. Percentages of bootstrap support are shown at branch nodes. Bootstrap value was 1000. Bar: 0.01 substitutions per nucleotide position. The phylogenominc tree based on gemome-sequence-alignment (c) and OrthoANI heatmap (d) of strain NSJ-35^T and its neighbor species. Refseq ID of representative genomes are given in parentheses. Percentages of bootstrap support are shown at branch nodes. Bar: 0.10 substitutions per nucleotide position.

Taxon 51: *Clostridium hominis* sp. nov.

Description *of Clostridium hominis* sp. nov. (ho'mi.nis. L. gen. masc. n. *hominis*, of a human being, referring to the human gut habitat)

The taxon 51, respresented by strain NSJ-6^T (16S rRNA sequence accession number in NMDC is NMDCN000011I), is phylogenetically closest to *Clostridium celatum*, with 16S rRNA gene identity of 97.88 %. Phylogenetic tree shows that strain NSJ-6^T clusters with other members of the genus *Clostridium,* (Figure SD-51a), suggesting strain NSJ-6^T is a member of the genus *Clostridium*. The genome of strain NSJ-6^T was sequenced and the NMDC accession number is NMDC60014048. Genome-based analysis showed that the ANI value of genomes of strain NSJ-6^T and *Clostridium celatum* JCM 1394^T (NZ AMEZ00000000) is 76.62 % and the dDDH estimation is 21.50 %. According to the phylogenominc tree (Figure SD-51c), the ANIs and dDDH values of genomes between strain NSJ-6^T (NMDC accession number is NMDC60014048) and phylogenomically neighbored genomes were calculated, and the highest ANI and dDDH values are 87.08 and 33.2 %, respectively, between the query genome of *Clostridium disporicum* (NZ_CYZX00000000.1); and difference in % G+C between genomes of strain NSJ-6^T and *Clostridium disporicum* (NZ_CYZX00000000.1) is 0.28; OrthoANI heatmap (Figure SD-51d) shows the phylogenomic status of the corresponding neighbor species based on OrthoANI values. Based on these results, we concluded that the strain NSJ-6^T represents of new species of the genus *Clostridium*, and the *Clostridium hominis* sp. nov. is proposed. Cells are strictly anaerobic, oval to rod-shaped (1.7-4.0 μm long by 1.1-1.8 wide, Figure SD-51b); cells appear singly, in dividing pairs or in chains. Irregular, white to yellow, moist, smooth, and convex colonies appear on modified mGAM agar plate after 3 days of incubation. Growth occurs at 37℃ and at pH of 7.0-7.5. The organism uses α-cyclodextrin, dextrin, D-fructose, L-fucose, D-galacturonic acid, D-galactose, gentiobiose, D-glucosaminic acid, α-D-glucose, glucose-1-phosphate, glucose-6-phosphate, lactulose, D-mannose, D-melibiose, 3-melthyl-D-glucose, palatinose, L-rhamnose, D-trehalose, turanose and inosine as the carbon source for growth, and weakly metabolizes acetyl-D glucosamine, N-acetyl-β-D mannosamine, D-arabitol, D-cellobiose, maltose, maltotriose, D-melezitose, α-methyl-D-galactoside, D-raffinose, salicin, pyruvic acid, pyruvic acid methyl ester, glycyl-L-methionine and uridine. The DNA G+C content of the type strain NSJ-6^T is 53.42 mol %. The type strain NSJ-6^T (=CGMCC 1.32461^T =KCTC 15960^T) was isolated from the faeces of a healthy adult.

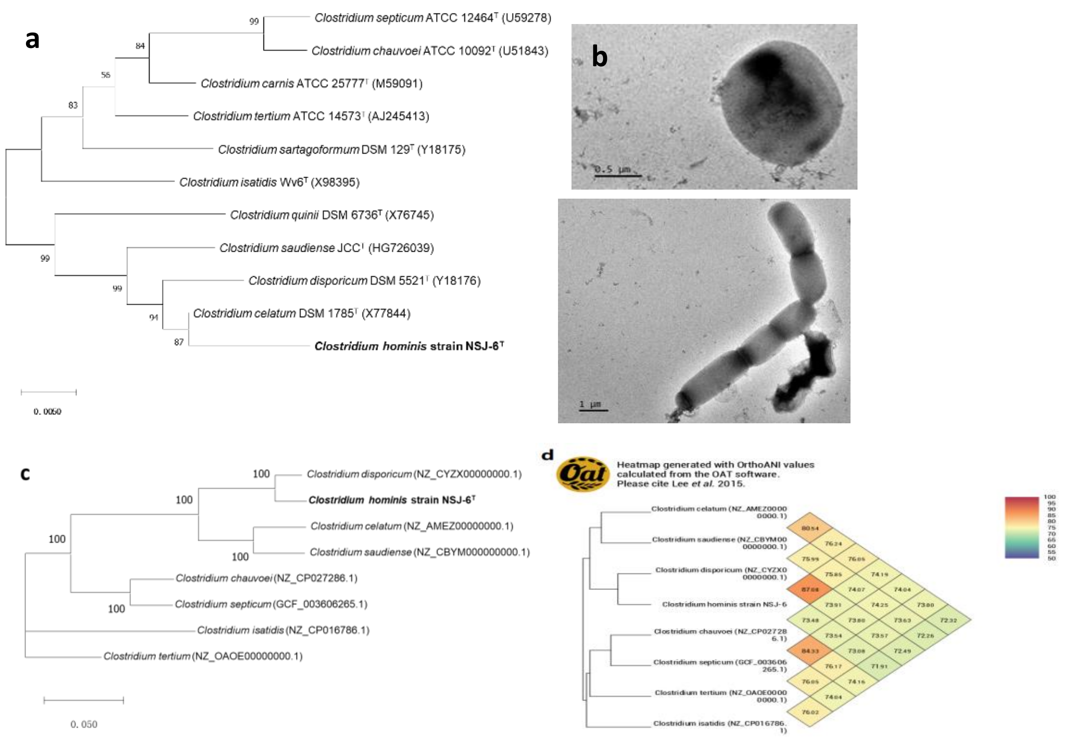

Figure SD-51. The Neighbour-joining phylogenetic tree based on 16S rRNA gene sequences (a) and the cell morphology (b) of strain NSJ-6^T. GenBank accession numbers are given in parentheses. Percentages of bootstrap support are shown at branch nodes. Bootstrap value was 1000. Bar: 0.005 substitutions per nucleotide position. The phylogenominc tree based on gemome-sequence-alignment (c) and OrthoANI heatmap (d) of strain NSJ-6^T and its neighbor species. Refseq ID of representative genomes are given in parentheses. Percentages of bootstrap support are shown at branch nodes. Bar: 0.050 substitutions per nucleotide position.

Taxon 53: *Clostridium lentum* sp. nov.

Description *of Clostridium lentum* sp. nov. (len'tum. L. neut. adj. *lentum*, slow, referring to the slow growth of the type strain)

The taxon 53, respresented by strain NSJ-42^T (16S rRNA sequence accession number in NMDC is NMDCN000011K), is phylogenetically closest to *Clostridium saudiense*, with 16S rRNA gene identity of 96.3 %. Phylogenetic tree shows that strain NSJ-42^T clusters with other members of the genus *Clostridium,* (Figure SD-53a), suggesting strain NSJ-42^T is a member of the genus *Clostridium*. The genome of strain NSJ-42^T was sequenced and the NMDC accession number is NMDC60014050. Genome-based analysis showed that the ANI value of genomes of strain NSJ-42^T and *Clostridium saudiense* JCC^T (NZ CBYM000000000) is 75.86 % and the dDDH estimation is 22.40 %. According to the phylogenominc tree (Figure SD-53c), the ANIs and dDDH values of genomes between strain NSJ-42^T (NMDC accession number is NMDC60014050) and phylogenomically neighbored genomes were calculated, and the highest ANI and dDDH values are 75.7251 and 21.3 %, respectively, between the query genome of *Clostridium disporicum* (NZ_CYZX00000000.1); and difference in % G+C between genomes of strain NSJ-42^T and *Clostridium disporicum* (NZ_CYZX00000000.1) is 0.01; OrthoANI heatmap (Figure SD-53d) shows the phylogenomic status of the corresponding neighbor species based on OrthoANI values. Based on these results, we concluded that the strain NSJ-42^T represents of new species of the genus *Clostridium*, and the name *Clostridium lentum* sp. nov. is proposed.

Cells are strictly anaerobic rods with lophotrichous flagella (2.5-5.1 μm long by 1.2-1.6 μm wide, Figure SD-53b). Irregular, white to grey, flat colonies appear on modified mGAM agar plate after 6 days of incubation. Growth occurs at 37°C and at pH 7.0-7.5. The organism uses D-fructose, L-fucose, D-galactose, D-galacturonic acid, gentiobiose, α-D-glucose, glucose-6-phosphate, D-mannose, D-melibiose, 3-melthyl-D-glucose, palatinose, L-rhamnose, glyoxylic acid, pyruvic acid and pyruvic acidmethyl ester as the carbon source for growth, and weakly metabolizes dextrin and α-ketovaleric acid. The DNA G+C content of the type strain NSJ-42^T is 52.68 mol %. The type strain NSJ-42^T (=CGMCC 1.32815^T) was isolated from the faeces of a healthy adult.

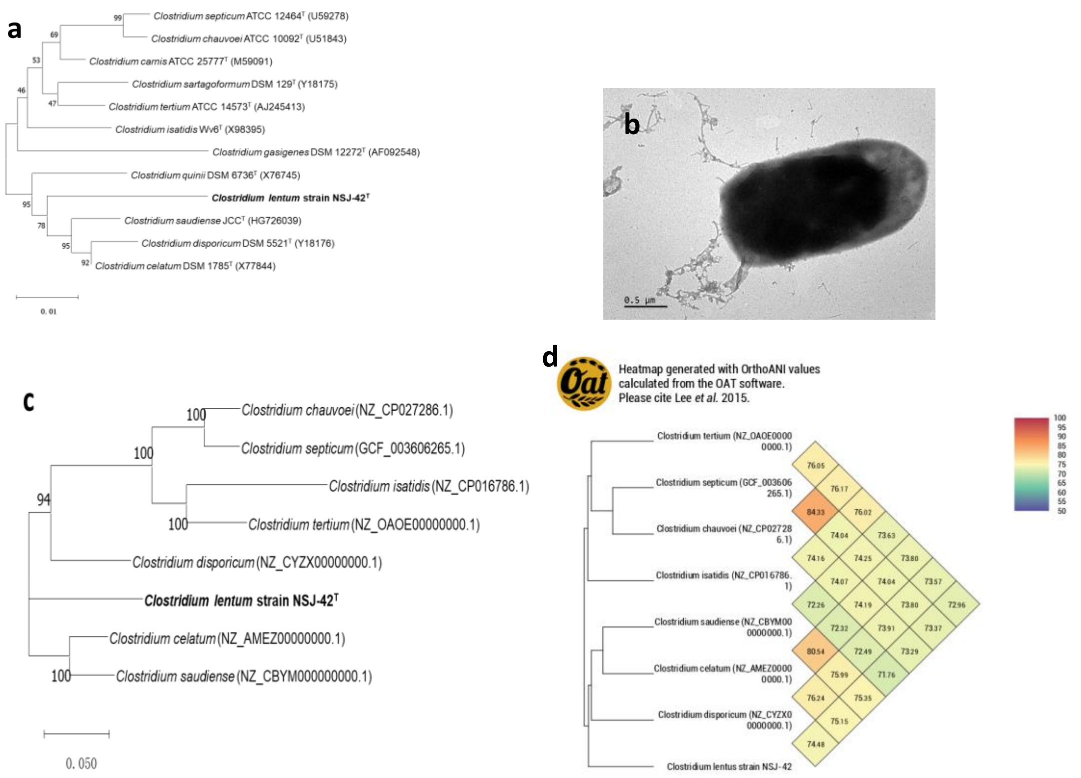

Figure SD-53. The Neighbour-joining phylogenetic tree based on 16S rRNA gene sequences (a) and the cell morphology (b) of strain NSJ-42^{T}. GenBank accession numbers are given in parentheses. Percentages of bootstrap support are shown at branch nodes. Bootstrap value was 1000. Bar: 0.01 substitutions per nucleotide position. The phylogenominc tree based on gemome-sequence-alignment (c) and OrthoANI heatmap (d) of strain NSJ-42^{T} and its neighbor species. Refseq ID of representative genomes are given in parentheses. Percentages of bootstrap support are shown at branch nodes. Bar: 0.050 substitutions per nucleotide position.

Taxon 54: *Clostridium facile* sp. nov.

Description *of Clostridium facile* sp. nov. (fa'ci.le. L. neut. adj. *facile*, easy, without difficulty, referring that the type strain is easily cultured)

The taxon 54, respresented by strain NSJ-27^T (16S rRNA sequence accession number in NMDC is NMDCN000011L), is phylogenetically closest to *Clostridium methylpentosum*, with 16S rRNA gene identity of 96.3 %. Phylogenetic tree shows that strain NSJ-27^T clusters with other members of the genus *Clostridium,* (Figure SD-54a), suggesting strain NSJ-27^T is a member of the genus *Clostridium*. The genome of strain NSJ-27^T was sequenced and the NMDC accession number is NMDC60014051. Genome-based analysis showed that the ANI value of genomes of strain NSJ-27^T and *Clostridium methylpentosum* DSM 5476^T (NZ ACEC00000000) is 69.33 % and the dDDH estimation is 25.60 %. According to the phylogenominc tree (Figure SD-54c), the ANIs and dDDH values of genomes between strain NSJ-27^T (NMDC accession number is NMDC60014051) and phylogenomically neighbored genomes were calculated, and the highest ANI and dDDH values are 67.62 and 25.6 %, respectively, between the query genome of *Clostridium methylpentosum* (GCF_902374025.1); and difference in % G+C between genomes of strain NSJ-27^T and *Clostridium methylpentosum* (GCF_902374025.1) is 12.35; OrthoANI heatmap (Figure SD-54d) shows the phylogenomic status of the corresponding neighbor species based on OrthoANI values. Based on these results, we concluded that the strain NSJ-27^T represents of new species of the genus *Clostridium*, and the name *Clostridium facile* sp. nov. is proposed.

Cells are strictly anaerobic, straight or curved rods (2.6-10.1 µm long by 0.5-0.9 µm wide, Figure SD-54b) in various sizes. Round, cream colored, smooth, convex colonies appear on modified mGAM agar plate after 3 days of incubation. Growth occurs at 37°C and at pH of 7.0-7.5. The organism uses D-cellobiose, dextrin, D-fructose, L-fucose, gentiobiose, α-D-glucose, glucose-6-phosphate, maltotriose, D-mannose, D-melezitose, 3-melthyl-D-glucose, palatinose, L-rhamnose, D-sorbitol, sucrose, D-trehalose, turanose, α-ketobutyric acid, α-ketovaleric acid and pyruvic acid as the carbon source for growth, and weakly metabolizes D-melibiose, glyoxylic acid. The DNA G+C content of the type strain is 51.75 mol %. The type strain is NSJ-27^T (=CGMCC 1.32800^T), which was isolated from the faeces of a healthy adult.

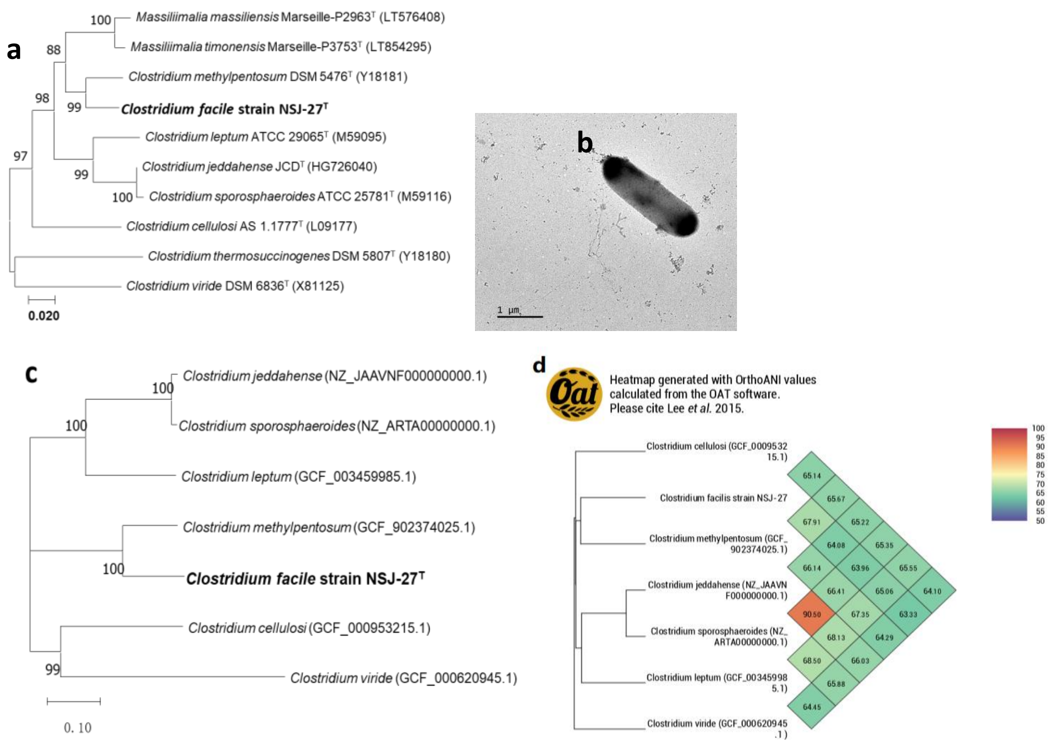

Figure SD-54. The Neighbour-joining phylogenetic tree based on 16S rRNA gene sequences (a) and the cell morphology (b) of strain NSJ-27^T. GenBank accession numbers are given in parentheses. Percentages of bootstrap support are shown at branch nodes. Bootstrap value was 1000. Bar: 0.02 substitutions per nucleotide position. The phylogenominc tree based on gemome-sequence-alignment (c) and OrthoANI heatmap (d) of strain NSJ-27^T and its neighbor species. Refseq ID of representative genomes are given in parentheses. Percentages of bootstrap support are shown at branch nodes. Bar: 0.10 substitutions per nucleotide position.

Taxon 55: ***Anaerosacchariphilus hominis*** **sp. nov.**

Description ***of Anaerosacchariphilus hominis*** **sp. nov.** (ho'mi.nis L. gen. masc. n. hominis, of a human being, referring to the human gut habitat)

The taxon 55, respresented by strain NSJ-68^T (16S rRNA sequence accession number in NMDC is NMDCN000011M), is phylogenetically closest to *Anaerosacchariphilus creatinophila*, with 16S rRNA gene identity of 94.8 %. Phylogenetic tree shows that strain NSJ-68^T clusters with other members of the genus *Anaerosacchariphilus,* (Figure SD-55a), suggesting strain NSJ-68^T is a member of the genus *Anaerosacchariphilus*. The genome of strain NSJ-68^T was sequenced and the NMDC accession number is NMDC60014052. Genome-based analysis showed that the ANI value of genomes of strain NSJ-68^T and *Anaerosacchariphilus polymeriproducens* MCWpalatinose^T (NZ ABYJ00000000) is 74.65 % and the dDDH estimation is 36.50 %. According to the phylogenominc tree (Figure SD-55c), the ANIs and dDDH values of genomes between strain NSJ-68^T (NMDC accession number is NMDC60014052) and phylogenomically neighbored genomes were calculated, and the highest ANI and dDDH values are 68.61 and 21 %, respectively, between the query genome of *Anaerosacchriphilus polymeriproducens* (NZ_QRCT00000000.1); and difference in % G+C between genomes of strain NSJ-68^T and *Anaerosacchriphilus polymeriproducens* (NZ_QRCT00000000.1) is 17.15; OrthoANI heatmap (Figure SD-55d) shows the phylogenomic status of the corresponding neighbor species based on OrthoANI values. Based on these results, we concluded that the strain NSJ-68^T represents a new species of the genus *Anaerosacchariphilus*, and the name *Anaerosacchariphilus hominis* sp. nov. is proposed.

Cells are strictly anaerobic, long, straight or slightly curved rods with tapered ends (3.5-6.0 µm long by 0.6-0.8 µm wide, Figure SD-55b); cells appear singly or in dividing pairs. Pinpoint, cream-colored, slightly raised colonies appear on modified mGAM agar plate after 6 days of incubation. Growth occurs at 37°C and at pH 7.0-7.5. The organism uses N-acetyl-D glucosamine, N-acetyl-β-D mannosamine, adonitol, amygdalin, D-arabitol, arbutin, D-cellobiose, dextrin, D-fructose, L-fucose, D-galactose, D-galacturonic acid, gentiobiose, D-gluconic acid, α-D-glucose, glucose-1-phosphate, glucose-6-phosphate, α-D-lactose, lactulose, maltose, maltotriose, D-mannose, D-melezitose, D-melibiose, 3-melthyl-D-glucose, ß-methyl-D-glucoside, palatinose, D-raffinose, L-rhamnose, salicin, turanose, glyoxylic acid, α-ketovaleric acid, D-lactic acidmethyl ester,pyruvic acid, pyruvic acidmethyl ester, L-alanine, L-alanyl-L-glutamine, L-alanyl-L-threonine, L-glutamic acid, L-glutamine, glycyl-L-glutamine, L-serine, L-threonine, L-valine plus L-aspartic acid, 2'-deoxyadenosine, inosine, thymidine, uridine and thymidine-5'-monophosphate as the carbon source for growth, and weakly metabolizes sucrose, m-tartaric acid and L-methionine. The DNA G+C content of the type strain NSJ-68^T is 54.45 mol %. The type strain NSJ-68^T (=CGMCC 1.32844^T =KCTC 25150^T) was isolated from the faeces of a healthy adult.

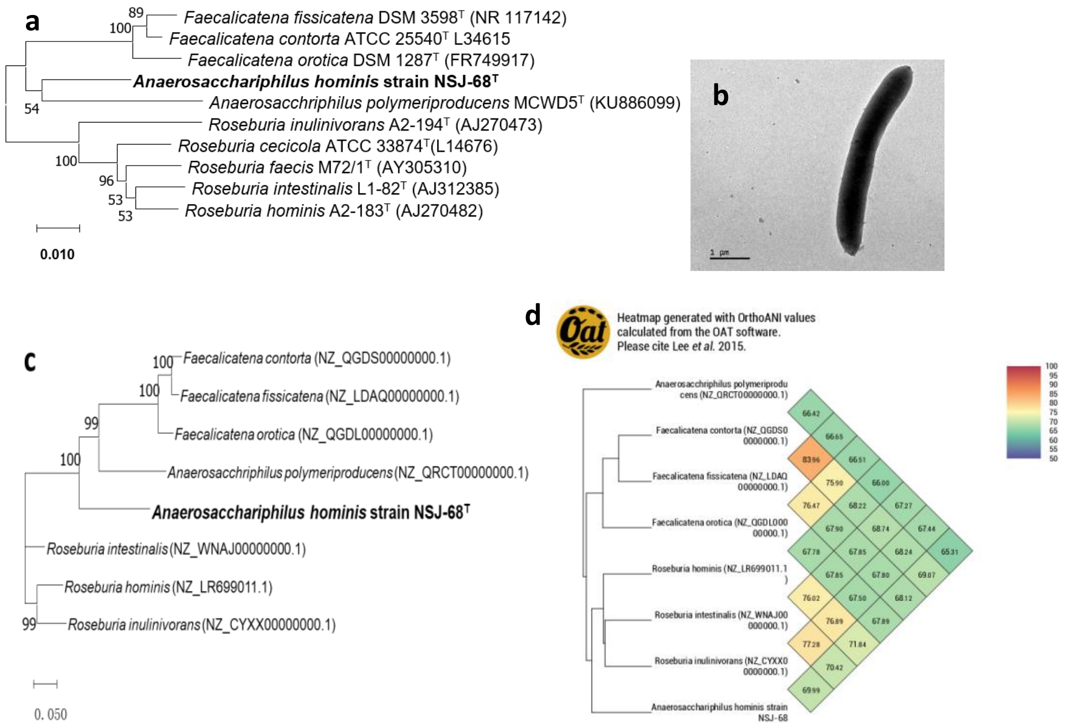

Figure SD-55. The Neighbour-joining phylogenetic tree based on 16S rRNA gene sequences (a) and the cell morphology (b) of strain NSJ-68^T. GenBank accession numbers are given in parentheses. Percentages of bootstrap support are shown at branch nodes. Bootstrap value was 1000. Bar: 0.01 substitutions per nucleotide position. The phylogenominc tree based on gemome-sequence-alignment (c) and OrthoANI heatmap (d) of strain NSJ-68^T and its neighbor species. Refseq ID of representative genomes are given in parentheses. Percentages of bootstrap support are shown at branch nodes. Bar: 0.050 substitutions per nucleotide position.

Taxon 56: *Anaerostipes hominis* sp. nov.

Description *of Anaerostipes hominis* sp. nov. (ho'mi.nis L. gen. masc. n. hominis, of a human being, referring to the human gut habitat)

The taxon 56, respresented by strain NSJ-7^T (16S rRNA sequence accession number in NMDC is NMDCN000011N), is phylogenetically closest to *Anaerostipes rhamnosivorans*, with 16S rRNA gene identity of 98.1 %. Phylogenetic tree shows that strain NSJ-7^T clusters with other members of the genus *Anaerostipes,* (Figure SD-56a), suggesting strain NSJ-7^T is a member of the genus *Anaerostipes*. The genome of strain NSJ-7^T was sequenced and the NMDC accession number is NMDC60014053. Genome-based analysis showed that the ANI value of genomes of strain NSJ-7^T and *Anaerostipes rhamnosivorans* 1y-2^T (NZ CP040058) is 92.07 % and the dDDH estimation is 48.40 %. According to the phylogenominc tree (Figure SD-56c), the ANIs and dDDH values of genomes between strain NSJ-7^T (NMDC accession number is NMDC60014053) and phylogenomically neighbored genomes were calculated, and the highest ANI and dDDH values are 92.05 and 48.4 %, respectively, between the query genome of *Anaerostipes caccae* (NZ_CP036345.1); and difference in % G+C between genomes of strain NSJ-7^T and *Anaerostipes caccae* (NZ_CP036345.1) is 0.89; OrthoANI heatmap (Figure SD-56d) shows the phylogenomic status of the corresponding neighbor species based on OrthoANI values. Based on these results, we concluded that the strain NSJ-7^T represents of new species of the genus *Anaerostipes*, and the name *Anaerostipes hominis* sp. nov. is proposed.

Cells are strictly anaerobic, straight rods in various sizes (1.8-4.7 μm long by 0.7-0.9 μm wide, Figure SD-56b); cells appear singly. Pinpoint, white, semi-translucent, convex, colonies appear on modified mGAM agar plate after 2 days of incubation. Growth occurs at 37°C and at pH of 7.0-7.5. The organism uses D-fructose, L-fucose, D-galacturonic acid, 3-melthyl-D-glucose, palatinose and L-rhamnose as the carbon source for growth, and weakly metabolizes D-galactose, gentiobiose, α-D-glucose, glucose-6-phosphate, glyoxylic acid, pyruvic acid and pyruvic acid methyl ester. The DNA G+C content of the type strain NSJ-7^T is 49.83 mol %. The type strain NSJ-7^T (=CGMCC=1.32462^T=KCTC 15959^T) was isolated from the faeces of a healthy adult.

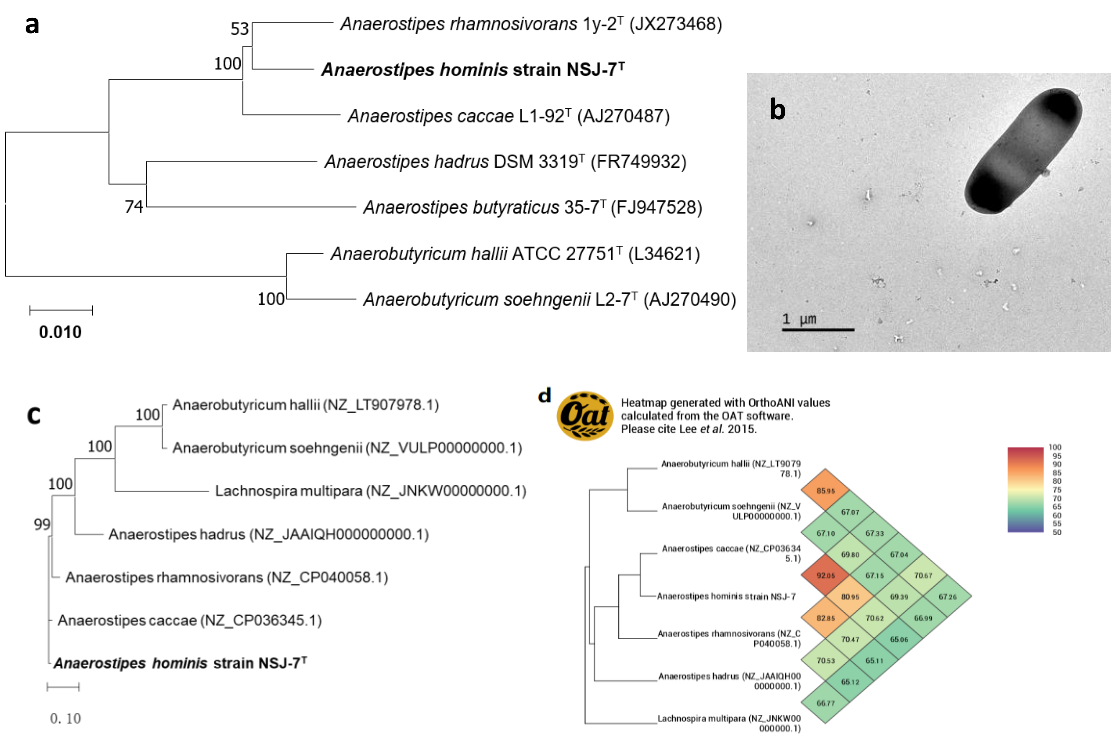

Figure SD-56. The Neighbour-joining phylogenetic tree based on 16S rRNA gene sequences (a) and the cell morphology (b) of strain NSJ-7^T. GenBank accession numbers are given in parentheses. Percentages of bootstrap support are shown at branch nodes. Bootstrap value was 1000. Bar: 0.01 substitutions per nucleotide position. The phylogenominc tree based on gemome-sequence-alignment (c) and OrthoANI heatmap (d) of strain NSJ-7^T and its neighbor species. Refseq ID of representative genomes are given in parentheses. Percentages of bootstrap support are shown at branch nodes. Bar: 0.1 substitutions per nucleotide position.

Taxon 57: *Blautia massiliensis* sp.

Description *of Blautia massiliensis* sp. (ma.si.li.e'n.sis. L. adj. masc. *massiliensis*, of Massilia, the Latin name of Marseill, where the bacteria was for the first time isolated, this name was given by Traore, et al.)

The taxon 57, respresented by strain 4-46^T (16S rRNA sequence accession number in NMDC is NMDCN000011O), is phylogenetically closest to *Blautia obeum*, with 16S rRNA gene identity of 96.6 %. Phylogenetic tree shows that strain 4-46^T clusters with other members of the genus *Blautia,* (Figure SD-57a), suggesting strain 4-46^T is a member of the genus *Blautia*. The genome of strain 4-46^T was sequenced and the NMDC accession number is NMDC60014054. Genome-based analysis showed that the ANI value of genomes of strain 4-46^T and *Blautia obeum* DSM ATCC 29174^T (NZ AAVO00000000) is 83.08 % and the dDDH estimation is 27.50 %. According to the phylogenominc tree (Figure SD-57c), the ANIs and dDDH values of genomes between strain 4-46^T (NMDC accession number is NMDC60014054) and phylogenomically neighbored genomes were calculated, and the highest ANI and dDDH values are 90.36 and 42.1 %, respectively, between the query genome of *Blautia luti* (NZ_JAAITI000000000.1); and difference in % G+C between genomes of strain 4-46^T and *Blautia luti* (NZ_JAAITI000000000.1) is 0.04; OrthoANI heatmap (Figure SD-57d) shows the phylogenomic status of the corresponding neighbor species based on OrthoANI values. Based on these results, we concluded that the strain 4-46^T represents of new species of the genus *Blautia*, and the name *Blautia massiliensis* sp. nov. is proposed by Traore. Thus we present the characterization and identification of the strain to validate the name *Blautia massiliensis.*

Cells are strictly anaerobic rods and some cells are with tapered ends (3.0-3.9 µm long by 0.4-0.9 µm wide, Figure SD-57b); cells appear singly, in dividing pairs or in chains. Irregular, brown, slightly raised colonies appear on modified mGAM agar plate after 3 days of incubation. Growth occurs at 37°C and at pH 7.0-7.5. The DNA G+C content of the type strain 4-46^T is 54.53 mol %. The type strain 4-46^T (=CGMCC 1.5283^T=NBRC 113773^T) was isolated from the faeces of a healthy adult.

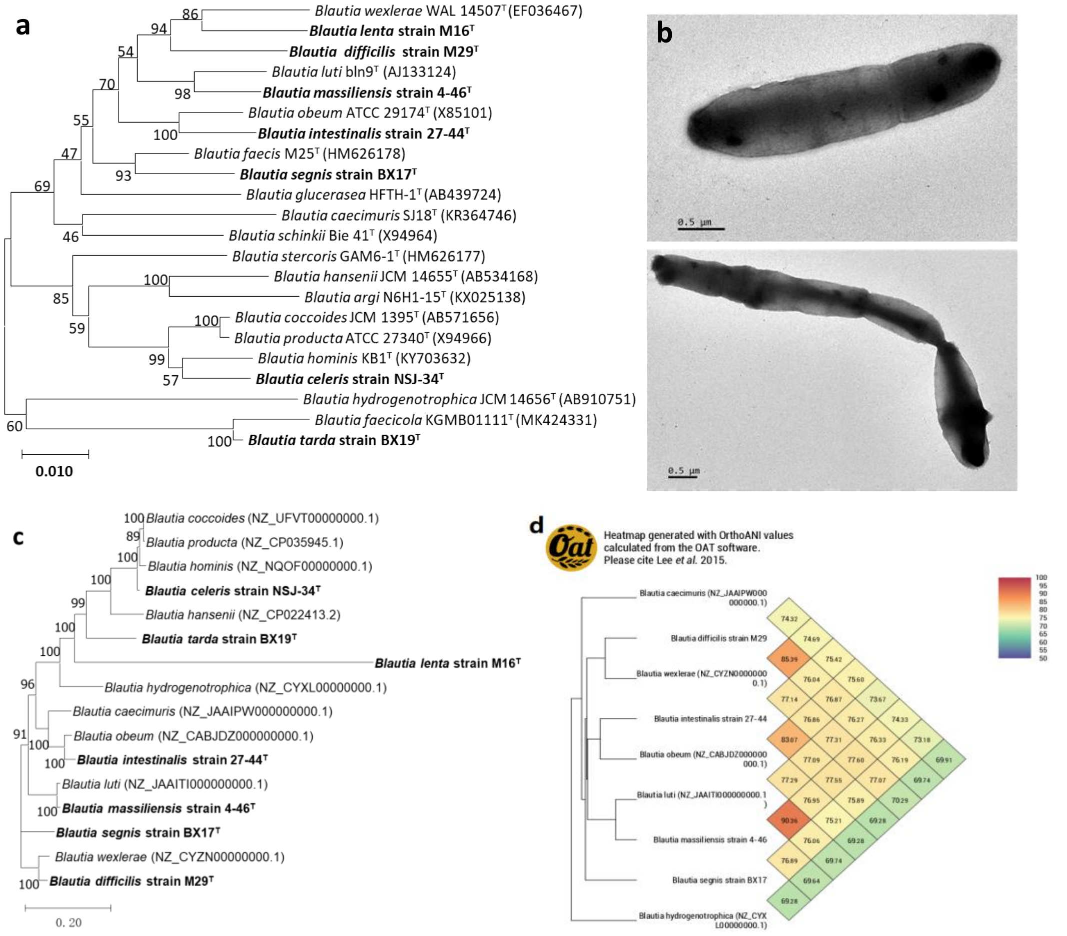

Figure SD-57. The Neighbour-joining phylogenetic tree based on 16S rRNA gene sequences (a) and the cell morphology (b) of strain 4-46ᵀ. GenBank accession numbers are given in parentheses. Percentages of bootstrap support are shown at branch nodes. Bootstrap value was 1000. Bar: 0.01 substitutions per nucleotide position. The phylogenominc tree based on gemome-sequence-alignment (c) and OrthoANI heatmap (d) of strain 4-46^{T} and its neighbor species. Refseq ID of representative genomes are given in parentheses. Percentages of bootstrap support are shown at branch nodes. Bar: 0.20 substitutions per nucleotide position.

Taxon 58: *Blautia intestinalis* sp. nov.

Description *of Blautia intestinalis* sp. nov. (in.tes.ti.na'lis N.L. fem. adj. *intestinalis*, pertaining to the intestines where the type strain inhabits)

The taxon 58, respresented by strain 27-44^T (16S rRNA sequence accession number in NMDC is NMDCN000011P), is phylogenetically closest to *Blautia obeum*, with 16S rRNA gene identity of 97.80 %. Phylogenetic tree shows that strain 27-44^T clusters with other members of the genus *Blautia,* (Figure SD-58a), suggesting strain 27-44^T is a member of the genus *Blautia*. The genome of strain 27-44^T was sequenced and the NMDC accession number is NMDC60014055. Genome-based analysis showed that the ANI value of genomes of strain 27-44^T and *Blautia obeum* DSM ATCC 29174^T (NZ AAVO00000000) is 83.08 % and the dDDH estimation is 27.50 %. According to the phylogenominc tree (Figure SD-58c), the ANIs and dDDH values of genomes between Blautia intestinalis strain 27-44 (NMDC accession number is NMDC60014055) and phylogenomically neighbored genomes were calculated, and the highest ANI and dDDH values are 83.07 and 27.5 %, respectively, between the query genome of *Blautia obeum* (NZ_CABJDZ000000000.1); and difference in % G+C between genomes of strain 27-44^T and *Blautia obeum* (NZ_CABJDZ000000000.1) is 0.77; OrthoANI heatmap (Figure SD-58d) shows the phylogenomic status of the corresponding neighbor species based on OrthoANI values.Based on these results, we concluded that the strain 27-44^T represents of new species of the genus *Blautia*, and the name *Blautia intestinalis* sp. nov. is proposed.

Cells are strictly anaerobic rods (2.1-2.8 µm long by 1.0-1.2 µm wide, Figure SD-58b); cells appear singly or in dividing pairs. Circular, whitish, slightly raised colonies appear on modified mGAM agar plate after 3 days of incubation. Growth occurs at 37°C and at pH 7.0-7.5. The organism uses N-acetyl-D glucosamine, N-acetyl-β-D mannosamine, amygdalin, arbutin, D-cellobiose, dextrin, D-galacturonic acid, dulcitol, D-fructose, L-fucose, D-galactose, gentiobiose, α-D-glucose, glucose-1-phosphate, glucose-6-phosphate, α-D-lactose, lactulose, maltose, maltotriose, D-mannose, D-melezitose, D-melibiose, 3-melthyl-D-glucose, α-methyl-D-galactoside, palatinose, ß-methyl-D-galactoside, α-methyl-D glucoside, ß-methyl-D-glucoside, D-raffinose, L-rhamnose, salicin, D-sorbitol, stachyose, sucrose, D-trehalose, turanose, glyoxylic acid, α-hydroxybutyric acid, L-serine, L-threonine, inosine and thymidine-5'-monophosphate, and weakly utilizes 3-melthyl-D-glucose and m-inositol. The DNA G+C content of the type strain 27-44^T is 54.62 mol %. The type strain 27-44^T (=CGMCC 1.5285^T =NBRC 113774^T) was isolated from the faeces of a healthy adult.

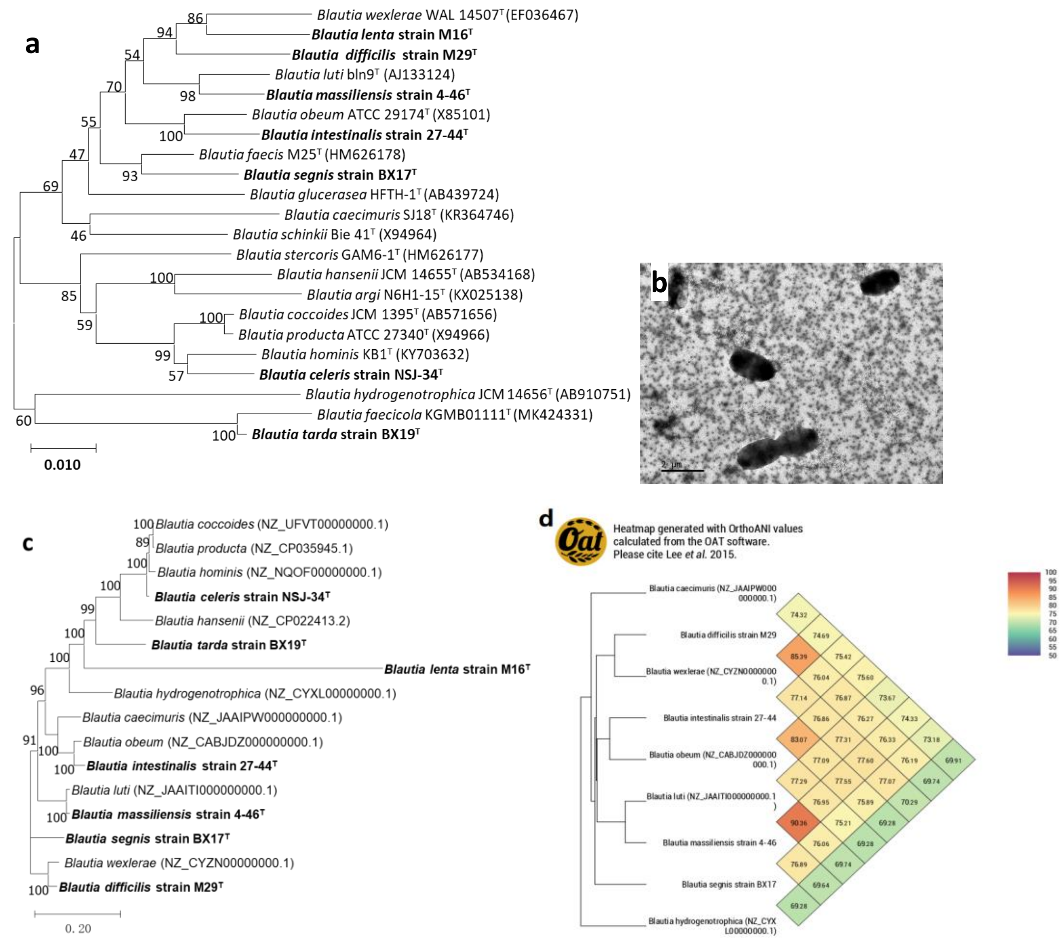

Figure SD-58. The Neighbour-joining phylogenetic tree based on 16S rRNA gene sequences (a) and the cell morphology (b) of strain 27-44^{T}. GenBank accession numbers are given in parentheses. Percentages of bootstrap support are shown at branch nodes. Bootstrap value was 1000. Bar: 0.01 substitutions per nucleotide position. The phylogenominc tree based on gemome-sequence-alignment (c) and OrthoANI heatmap (d) of strain 27-44^{T} and its neighbor species. Refseq ID of representative genomes are given in parentheses. Percentages of bootstrap support are shown at branch nodes. Bar: 0.20 substitutions per nucleotide position.

Taxon 59: *Blautia segnis* sp. nov.

Description *of Blautia segnis* sp. nov. (L. masc. adj. segnis, slow, inactive, lazy, referring the slow growth of the strain BX17^T)

The taxon 59, respresented by strain BX17^T (16S rRNA sequence accession number in NMDC is NMDCN000011Q), is phylogenetically closest to *Blautia faecis*, with 16S rRNA gene identity of 97.7 %. Phylogenetic tree shows that strain BX17^T clusters with other members of the genus *Blautia,* (Figure SD-59a), suggesting strain BX17^T is a member of the genus *Blautia*. The genome of strain BX17^T was sequenced and the NMDC accession number is NMDC60014056. Genome-based analysis showed that the ANI value of genomes of strain BX17^T and *Blautia faecis* M7-8^T (NZ_JAAIPV000000000.1) is 74.78 % and the dDDH estimation is 17.0 %. According to the phylogenominc tree (Figure SD-59c), the ANIs and dDDH values of genomes between strain BX17^T (NMDC accession number is NMDC60014056) and phylogenomically neighbored genomes were calculated, and the highest ANI and dDDH values are 77.07 and 29.6 %, respectively, between the query genome of *Blautia wexlerae* (NZ_CYZN00000000.1); and difference in % G+C between genomes of strain BX17^T and *Blautia wexlerae* (NZ_CYZN00000000.1) is 3.12; OrthoANI heatmap (Figure SD-59d) shows the phylogenomic status of the corresponding neighbor species based on OrthoANI values. Based on these results, we concluded that the strain BX17^T represents of new species of the genus *Blautia*, and the name *Blautia segnis* sp. nov. is proposed.

Cells are strictly anaerobic straight or slightly curved rods (2.6-4.6 μm long by 0.9-1.2 μm wide, Figure SD-59b); cells appear singly. Growth occurs at 37°C and at pH 7.0-7.5. The DNA G+C content of the type strain BX17^T is 44.2 mol %. The type strain BX17^T (=CGMCC 1.32886^T) was isolated from the faeces of a healthy adult.

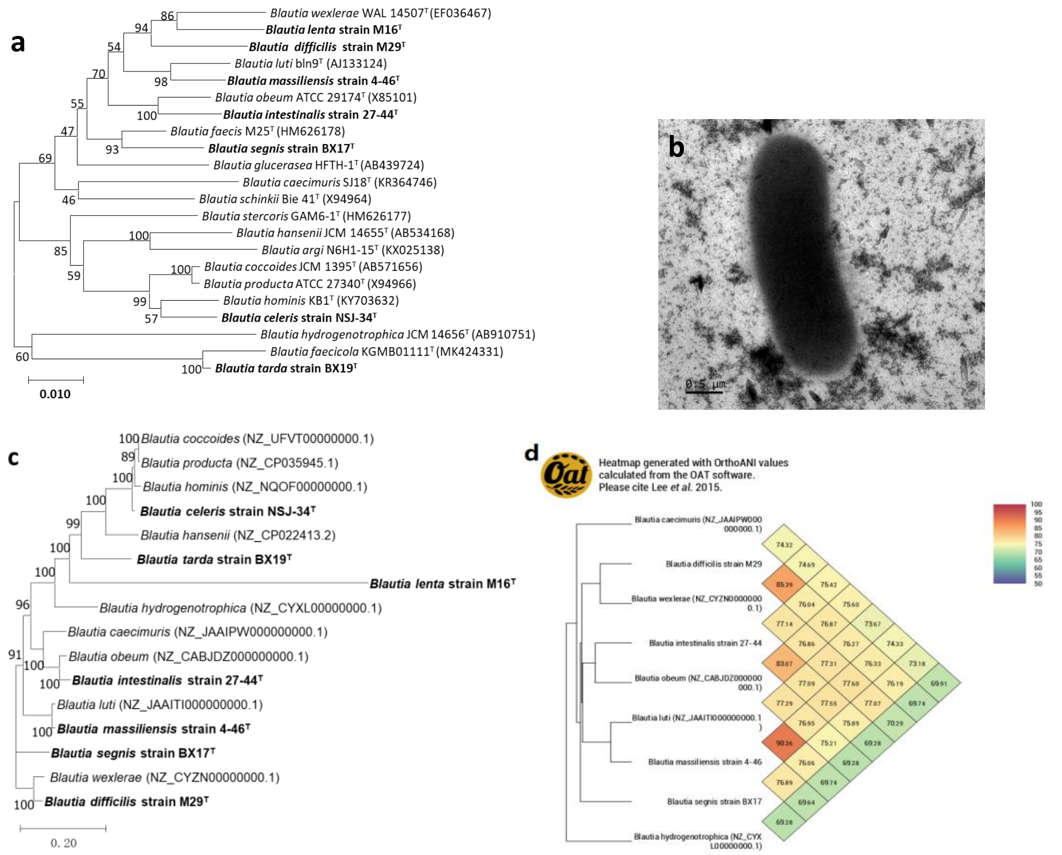

Figure SD-59. The Neighbour-joining phylogenetic tree based on 16S rRNA gene sequences (a) and the cell morphology (b) of strain BX17^T. GenBank accession numbers are given in parentheses. Percentages of bootstrap support are shown at branch nodes. Bootstrap value was 1000. Bar: 0.01 substitutions per nucleotide position. The phylogenominc tree based on gemome-sequence-alignment (c) and OrthoANI heatmap (d) of strain BX17^T and its neighbor species. Refseq ID of representative genomes are given in parentheses. Percentages of bootstrap support are shown at branch nodes. Bar: 0.20 substitutions per nucleotide position.

Taxon 60: *Blautia tarda* sp. nov.

Description *of Blautia tarda* sp. nov. (tar'da. L. fem. adj. *tarda*, slow, inactive, lazy, referring the slow growth of the strain)

The taxon 60, respresented by strain BX19^T (16S rRNA sequence accession number in NMDC is NMDCN000011R), is phylogenetically closest to *Blautia stercoris*, with 16S rRNA gene identity of 95.1 %. Phylogenetic tree shows that strain BX19^T clusters with other members of the genus *Blautia,* (Figure SD-60a), suggesting strain BX19^T is a member of the genus *Blautia*. The genome of strain BX19^T was sequenced and the NMDC accession number is NMDC60014057. Genome-based analysis showed that the ANI value of genomes of strain BX19^T and *Blautia stercoris* GAM6-1^T is 91.95 % and the dDDH estimation is 47.70 %. According to the phylogenominc tree (Figure SD-60c), the ANIs and dDDH values of genomes between strain BX19^T (NMDC accession number is NMDC60014057) and phylogenomically neighbored genomes were calculated, and the highest ANI and dDDH values are 70.62 and 24 %, respectively, between the query genome of *Blautia caecimuris* (NZ_JAAIPW000000000.1); and difference in % G+C between genomes of strain BX19^T and *Blautia caecimuris* (NZ_JAAIPW000000000.1) is 0.74; OrthoANI heatmap (Figure SD-60d) shows the phylogenomic status of the corresponding neighbor species based on OrthoANI values. Based on these results, we concluded that the strain BX19^T represents of new species of the genus *Blautia*, and the name *Blautia tarda* sp. nov. is proposed.

Cells are strictly anaerobic straight rods with tapered ends (2.5-5.4 µm long by 0.6-1.1 µm wide, Figure SD-60b); cells appear singly. Growth occurs at 37 °C and at pH 7.0-7.5. The DNA G+C content of the type strain BX19^T is 44.2 mol %. The type strain BX19^T (=CGMCC 1.32887^T) was isolated from the faeces of a healthy adult.

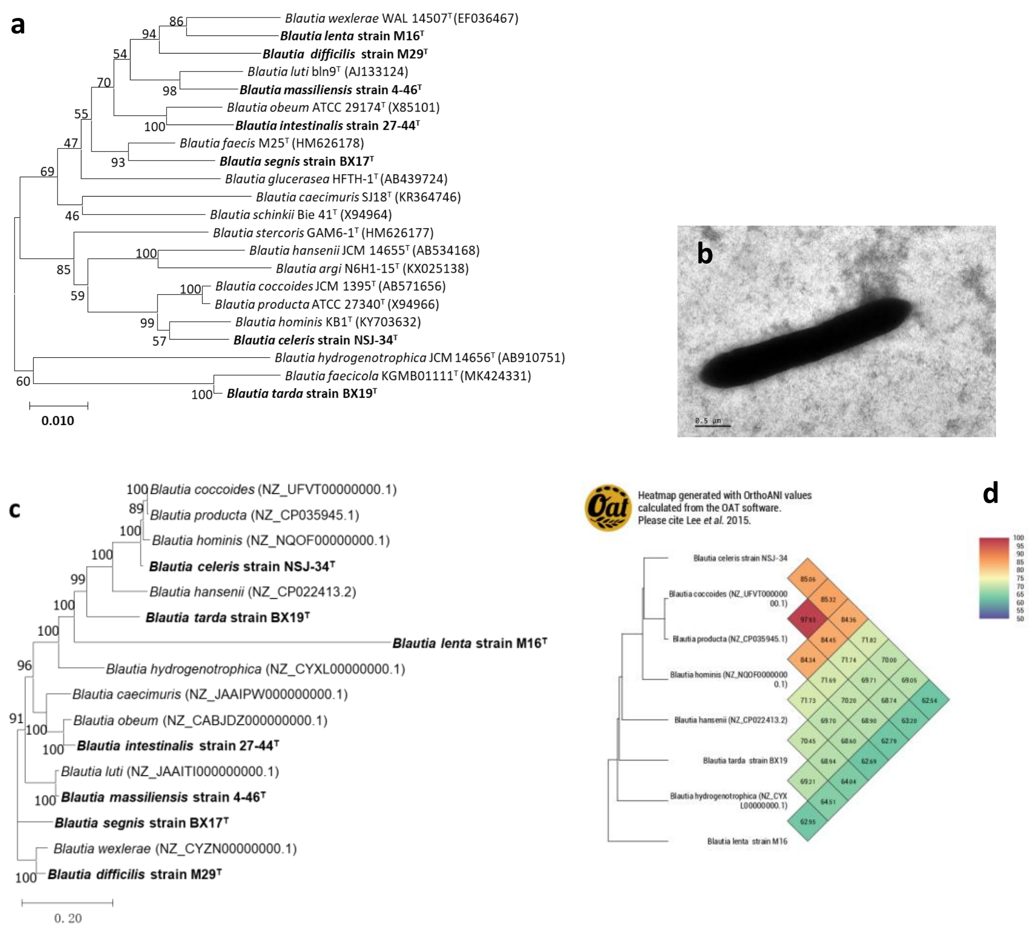

Figure SD-60. The Neighbour-joining phylogenetic tree based on 16S rRNA gene sequences (a) and the cell morphology (b) of strain BX19^T. GenBank accession numbers are given in parentheses. Percentages of bootstrap support are shown at branch nodes. Bootstrap value was 1000. Bar: 0.01 substitutions per nucleotide position. The phylogenomminc tree based on gemome-sequence-alignment (c) and OrthoANI heatmap (d) of strain BX19^T and its neighbor species. Refseq ID of representative genomes are given in parentheses. Percentages of bootstrap support are shown at branch nodes. Bar: 0.20 substitutions per nucleotide position.

Taxon 61: *Blautia celeris* sp. nov.

Description *of Blautia celeris* sp. nov. (ce'le.ris L. fem. adj. *celeris*, rapid, pertaining to fast growth of the strain)

The taxon 61, respresented by strain NSJ-34^T (16S rRNA sequence accession number in NMDC is NMDCN000011S), is phylogenetically closest to *Blautia hominis*, with 16S rRNA gene identity of 97.92 %. Phylogenetic tree shows that strain NSJ-34^T clusters with other members of the genus *Blautia,* (Figure SD-61a), suggesting strain NSJ-34^T is a member of the genus *Blautia*. The genome of strain NSJ-34^T was sequenced and the NMDC accession number is NMDC60014058. Genome-based analysis showed that the ANI value of genomes of strain NSJ-34^T and *Blautia hominis* Kdulcitol^T (NZ NQOF00000000) is 84.15 % and the dDDH estimation is 28.80 %. According to the phylogenominc tree (Figure SD-61c), the ANIs and dDDH values of genomes between strain NSJ-34^T (NMDC accession number is NMDC60014058) and phylogenomically neighbored genomes were calculated, and the highest ANI and dDDH values are 85.32 and 30.2 %, respectively, between the query genome of *Blautia producta* (NZ_CP035945.1); and difference in % G+C between genomes of strain NSJ-34^T and *Blautia producta* (NZ_CP035945.1) is 1; OrthoANI heatmap (Figure SD-61d) shows the phylogenomic status of the corresponding neighbor species based on OrthoANI values. Based on these results, we concluded that the strain NSJ-34^T represents of new species of the genus *Blautia*, and the name *Blautia celeris* sp. nov. is proposed.

Cells are strictly anaerobic straight rods with tapered ends (1.5-1.8 µm long by 0.7-1.2 µm wide, Figure SD-61b); cells appear singly or in dividing pairs. Irregular, yellow, raised, rough colonies appear on modified mGAM agar plate after 3 days of incubation. Growth occurs at 37°C and at pH 7.0-7.5. The organism uses D-fructose, L-fucose, D-galacturonic acid, palatinose and D-malic acid as the carbon source for growth, and weakly metabolizes D-galactose, L-rhamnose and pyruvic acid. The DNA G+C content of the type strain NSJ-34^T is 54.21 mol %. The type strain NSJ-34^T (=CGMCC 1.32807^T) was isolated from the faeces of a healthy adult.

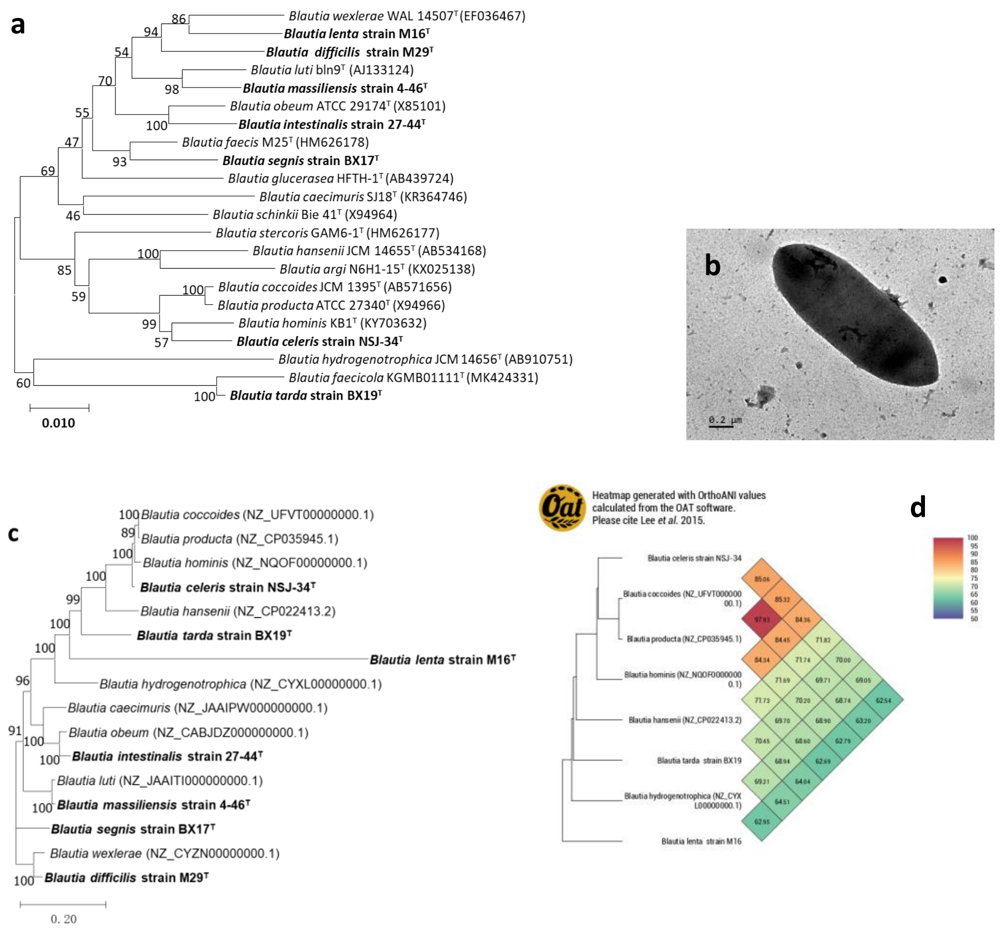

Figure SD-61. The Neighbour-joining phylogenetic tree based on 16S rRNA gene sequences (a) and the cell morphology (b) of strain NSJ-34^{T}. GenBank accession numbers are given in parentheses. Percentages of bootstrap support are shown at branch nodes. Bootstrap value was 1000. Bar: 0.01 substitutions per nucleotide position. The phylogenominc tree based on gemome-sequence-alignment (c) and OrthoANI heatmap (d) of strain NSJ-34^{T} and its neighbor species. Refseq ID of representative genomes are given in parentheses. Percentages of bootstrap support are shown at branch nodes. Bar: 0.20 substitutions per nucleotide position.

Taxon 62: *Blautia lenta* sp. nov.

Description *of Blautia lenta* sp. nov. (len'ta. L. fem. adj. lenta, slow, referring to the slow growth of the type strain)

The taxon 62, respresented by strain M16^T (16S rRNA sequence accession number in NMDC is NMDCN000011T), is phylogenetically closest to *Blautia luti*, with 16S rRNA gene identity of 98.29 %. Phylogenetic tree shows that strain M16^T clusters with other members of the genus *Blautia,* (Figure SD-62a), suggesting strain M16^T is a member of the genus *Blautia*. The genome of strain M16^T was sequenced and the NMDC accession number is NMDC60014059. Genome-based analysis showed that the ANI value of genomes of strain M16^T and *Blautia luti* DSM 14534^T (NZ WMBC00000000) is 66.1 % and the dDDH estimation is 41.50 %, DNA G+C content difference is 14.6 mol %. According to the phylogenominc tree (Figure SD-62c), the ANIs and dDDH values of genomes between strain M16^T (NMDC accession number is NMDC60014059) and phylogenomically neighbored genomes were calculated, and the highest ANI and dDDH values are 64.04 and 31.7 %, respectively, between the query genome of *Blautia hansenii* (NZ_CP022413.2); and difference in % G+C between genomes of strain M16^T and *Blautia hansenii* (NZ_CP022413.2) is 9.52; OrthoANI heatmap (Figure SD-62d) shows the phylogenomic status of the corresponding neighbor species based on OrthoANI values. Based on these results, we concluded that the strain M16^T represents of new species of the genus *Blautia*, and the name *Blautia lenta* sp. nov. is proposed.

Cells are strictly anaerobic, curved or straight rods in various sizes with round ends (1.5-5.9 µm long by 0.5-1.2 µm wide, Figure SD-62b); cells appear singly or in dividing pairs. Growth occurs at 37 °C and at pH 7.0-7.5. The organism uses D-fructose, L-fucose, D-galactose, D-galacturonic acid, α-D-glucose, D-mannose, 3-melthyl-D-glucose, palatinose, L-rhamnose and pyruvic aicd as the carbon source for growth, and weakly metabolizes dextrin, gentiobiose, glucose-6-phosphate, D-melibiose, turanose, glyoxylic acid, α-ketobutyric acid and pyruvic acidmethyl ester. The DNA G+C content of the type strain M16^T is 29.50 mol %. The type strain M16^T (=CGMCC 1.32888^T) was isolated from the faeces of a healthy adult.

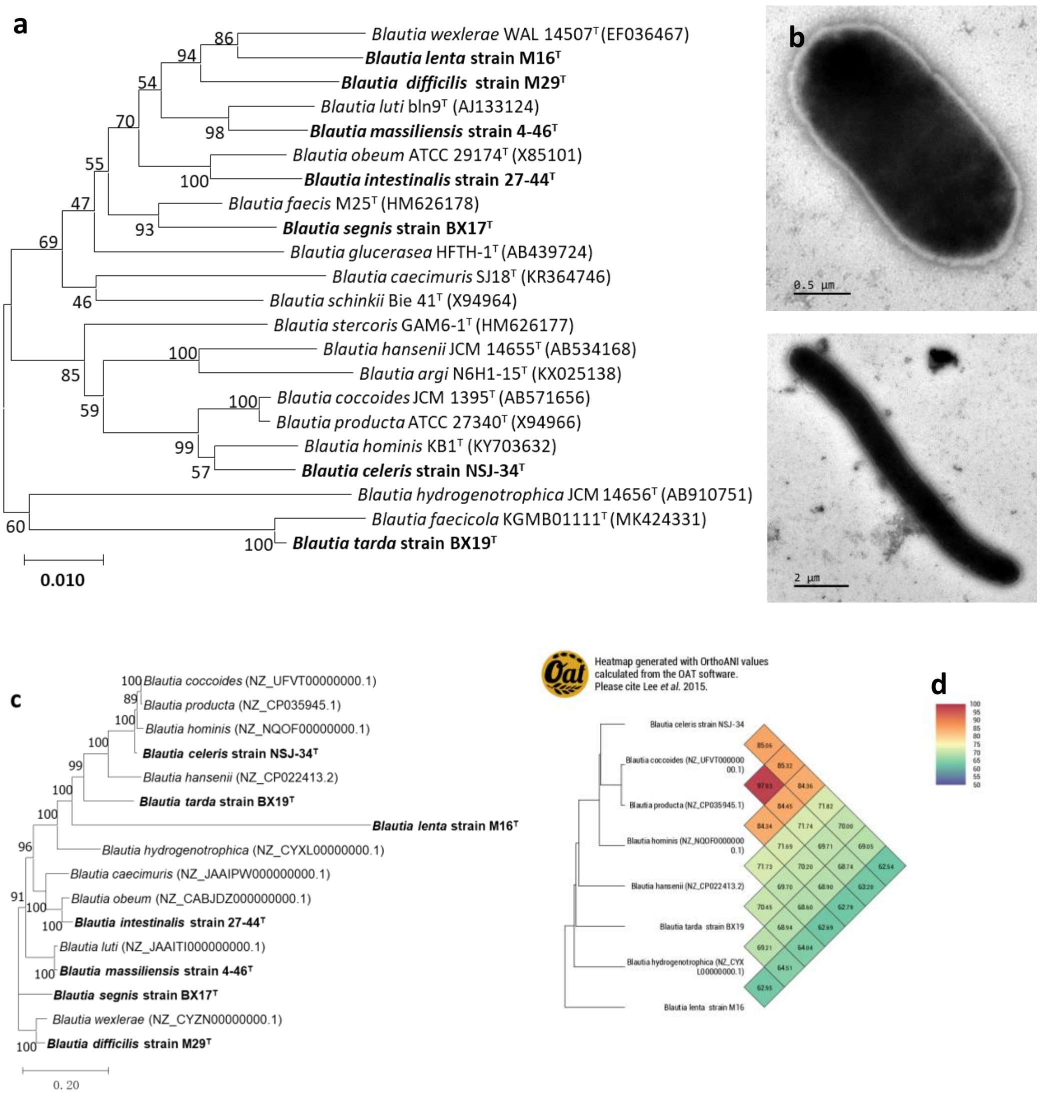

Figure SD-62. The Neighbour-joining phylogenetic tree based on 16S rRNA gene sequences (a) and the cell morphology (b) of strain M16^T. GenBank accession numbers are given in parentheses. Percentages of bootstrap support are shown at branch nodes. Bootstrap value was 1000. Bar: 0.01 substitutions per nucleotide position. The phylogenominc tree based on gemome-sequence-alignment (c) and OrthoANI heatmap (d) of strain M16^T and its neighbor species. Refseq ID of representative genomes are given in parentheses. Percentages of bootstrap support are shown at branch nodes. Bar: 0.20 substitutions per nucleotide position.

Taxon 63: *Blautia difficilis* sp. nov.

Description *of Blautia difficilis* sp. nov. (diff'i.cil.is L. masc. adj. difficilis, difficult, because it is difficult to cultivate)
The taxon 63, respresented by strain M29^T (16S rRNA sequence accession number in NMDC is NMDCN000011U), is phylogenetically closest to *Blautia luti*, with 16S rRNA gene identity of 97.6 %. Phylogenetic tree shows that strain M29^T clusters with other members of the genus *Blautia,* (Figure SD-63a), suggesting strain M29^T is a member of the genus *Blautia*. The genome of strain M29^T was sequenced and the NMDC accession number is NMDC60014060. Genome-based analysis showed that the ANI value of genomes of strain M29^T and *Blautia luti* DSM 14534^T (NZ WMBC00000000) is 80.07 % and the dDDH estimation is 28.10 %. According to the phylogenominc tree (Figure SD-63c), the ANIs and dDDH values of genomes between strain M29^T (NMDC accession number is NMDC60014060) and phylogenomically neighbored genomes were calculated, and the highest ANI and dDDH values are 85.39 and 37 %, respectively, between the query genome of *Blautia wexlerae* (NZ_CYZN00000000.1); and difference in % G+C between genomes of strain M29^T and *Blautia wexlerae* (NZ_CYZN00000000.1) is 0.39; OrthoANI heatmap (Figure SD-63d) shows the phylogenomic status of the corresponding neighbor species based on OrthoANI values. Based on these results, we concluded that the strain M29^T represents of new species of the genus *Blautia*, and the name *Blautia difficilis* sp. nov. is proposed.
Cells are strictly anaerobic, ovoid to short rods (1.2-2.0 µm long by 0.5-1.2 µm wide, Figure SD-63b); cells appear singly or in (dividing) pairs. Irregular and occasionally spreading, pale yellow, raised colonies appear on modified mGAM agar plate after3 days of incubation. Growth occurs at 37°C and at pH 7.0-7.5. The organism uses D-fructose, L-fucose,, glucose-6-phosphate, D-galactose, α-D-glucose, gentiobiose D-mannose, D-melibiose, 3-melthyl-D-glucose, palatinose, L-rhamnose, α-ketobutyric acid and pyruvic acid as carbon source for growth, and weakly metabolizes dextrin and glyoxylic acid. The DNA G+C content of the type strain M29^T is 54.51 mol %. The type strain M29^T (=CGMCC 1.32852^T) was isolated from the faeces of a healthy adult.

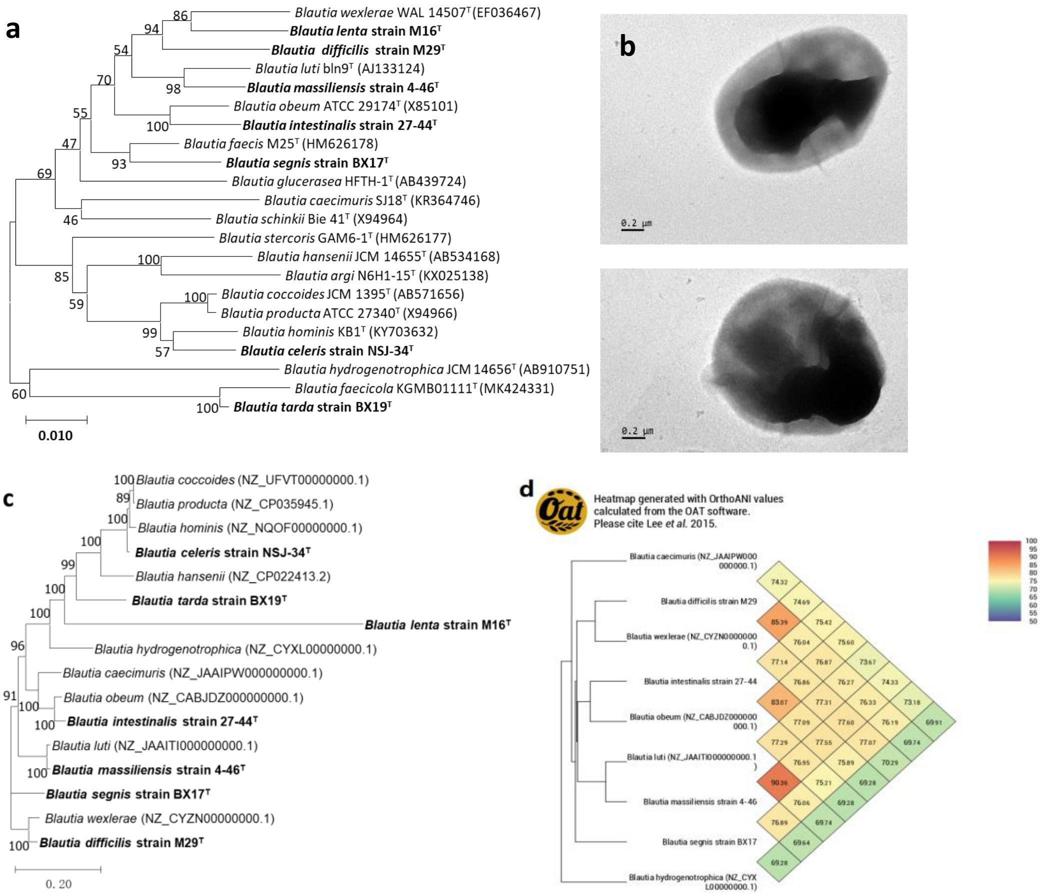

Figure SD-63. The Neighbour-joining phylogenetic tree based on 16S rRNA gene sequences (a) and the cell morphology (b) of strain M29^T. GenBank accession numbers are given in parentheses. Percentages of bootstrap support are shown at branch nodes. Bootstrap value was 1000. Bar: 0.01 substitutions per nucleotide position. The phylogenominc tree based on gemome-sequence-alignment (c) and OrthoANI heatmap (d) of strain M29^T and its neighbor species. Refseq ID of representative genomes are given in parentheses. Percentages of bootstrap support are shown at branch nodes. Bar: 0.2 substitutions per nucleotide position.

Taxon 64: *Clostridium segne* sp. nov.

Description *of Clostridium segne* sp. nov. (seg'ne. L. neut. adj. *segne*, slow, inactive, lazy, referring the slow growth of the strain)

The taxon 64, respresented by strain BX14^T (16S rRNA sequence accession number in NMDC is NMDCN000011V), is phylogenetically closest to *Clostridium symbiosum*, with 16S rRNA gene identity of 95.69 %. Phylogenetic tree shows that strain BX14^T clusters with other members of the genus *Clostridium,* (Figure SD-64a), suggesting strain BX14^T is a member of the genus *Clostridium*. The genome of strain BX14^T was sequenced and the NMDC accession number is NMDC60014061. Genome-based analysis showed that the ANI value of genomes of strain BX14^T and *Clostridium symbiosum* ATCC 14940^T (NZ ADLQ00000000) is 72.21 % and the dDDH estimation is 25.40 %. According to the phylogenominc tree (Figure SD-64c), the ANIs and dDDH values of genomes between strain BX14^T (NMDC accession number is NMDC60014061) and phylogenomically neighbored genomes were calculated, and the highest ANI and dDDH values are 70.4907 and 20.4 %, respectively, between the query genome of *Clostridium aminophilum* (NZ_FOZC00000000.1); and difference in % G+C between genomes of strain BX14^T and *Clostridium aminophilum* (NZ_FOZC00000000.1) is 2.23; OrthoANI heatmap (Figure SD-64d) shows the phylogenomic status of the corresponding neighbor species based on OrthoANI values. Based on these results, we concluded that the strain BX14^T represents of new species of the genus *Clostridium*, and the name *Clostridium segne* sp. nov. is proposed.

Cells are strictly anaerobic and are straight or slightly curverd rod shaped (1.2– 1.8 µm long by 0.8–1.2 µm wide, Figure SD-64b); cells appear singly. Growth occurs at 37℃ and at pH of 7.0-7.5. The DNA G+C content of the type strain BX14^T is 48.15 mol %. The type strain BX14^T (=CGMCC 1.32890^T) was isolated from the faeces of a healthy adult.

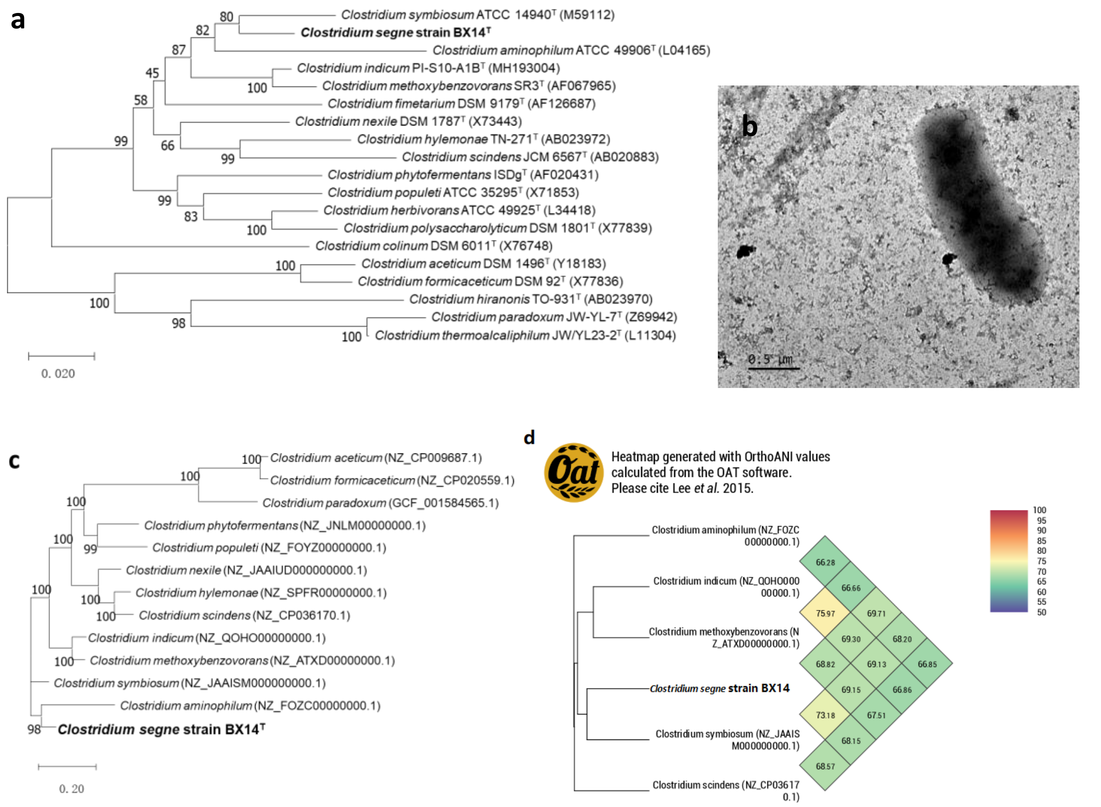

Figure SD-64. The Neighbour-joining phylogenetic tree based on 16S rRNA gene sequences (a) and the cell morphology (b) of strain BX14^T. GenBank accession numbers are given in parentheses. Percentages of bootstrap support are shown at branch nodes. Bootstrap value was 1000. Bar: 0.02 substitutions per nucleotide position. The phylogenominc tree based on gemome-sequence-alignment (c) and OrthoANI heatmap (d) of strain BX14^T and its neighbor species. Refseq ID of representative genomes are given in parentheses. Percentages of bootstrap support are shown at branch nodes. Bar: 0.20 substitutions per nucleotide position.

Taxon 65: *Coprococcus hominis* sp. nov.

Description *of Coprococcus hominis* sp. nov. (ho'mi.nis L. gen. masc. n. hominis, of a human being, referring to the human gut habitat)

The taxon 65, respresented by strain NSJ-10^T (16S rRNA sequence accession number in NMDC is NMDCN0000120), is phylogenetically closest to *Coprococcus eutactus*, with 16S rRNA gene identity of 96.0 %. Phylogenetic tree shows that strain NSJ-10^T clusters with other members of the genus *Coprococcus,* (Figure SD-65a), suggesting strain NSJ-10^T is a member of the genus *Coprococcus*. The genome of strain NSJ-10^T was sequenced and the NMDC accession number is NMDC60014062. Genome-based analysis showed that the ANI value of genomes of strain NSJ-10^T and *Coprococcus eutactus* ATCC 27759^T (NZ ABEY00000000) is 73.61 % and the dDDH estimation is 27.90 %. According to the phylogenominc tree (Figure SD-65c), the ANIs and dDDH values of genomes between strain NSJ-10^T (NMDC accession number is NMDC60014062) and phylogenomically neighbored genomes were calculated, and the highest ANI and dDDH values are 73.05 and 27.9 %, respectively, between the query genome of *Coprococcus eutactus* (NZ_ABEY00000000.2); and difference in % G+C between genomes of strain NSJ-10^T and *Coprococcus eutactus* (NZ_ABEY00000000.2) is 1.85; OrthoANI heatmap (Figure SD-65d) shows the phylogenomic status of the corresponding neighbor species based on OrthoANI values. Based on these results, we concluded that the strain NSJ-10^T represents of new species of the genus *Coprococcus*, and the name *Coprococcus hominis* sp. nov. is proposed.

Cells are strictly anaerobic and are oval shaped (1.2– 1.6 µm long by 0.8–1.3 µm wide, Figure SD-65b); cells appear singly or in dividing pairs. Tiny, circular, semi-translucent, slightly raised, entire colonies appear on modified mGAM agar slant medium after 2 days of incubation. Growth occurs at 37℃ and at pH of 7.0-7.5. The organism uses amygdalin, arbutin, D-cellobiose, dextrin, D-fructose, L-fucose, D-galactose, D-galacturonic acid, gentiobiose, α-D-glucose, glucose-1-phosphate, glucose-6-phosphate, α-D-lactose, lactulose, maltose, maltotriose, D-mannose, D-melibiose, 3-melthyl-D-glucose, α-methyl-D-galactoside, palatinose, D-raffinose, L-rhamnose, salicin, D-sorbitol, stachyose, sucrose, D-trehalose, turanose, α-ketobutyric acid, α-ketovaleric acid, D-lactic acid methyl ester, L-methionine, L-phenylalanine, L-valine, L-valine plus L-aspartic acid, inosine, uridine and uridine-5'-monophophate as the carbon source for growth, and weakly metabolizes D-melezitose. The DNA G+C content of the type strain NSJ-10^T is 51.02 mol %. The type strain NSJ-10^T (=CGMCC 1.32463^T) was isolated from the faeces of a healthy adult.

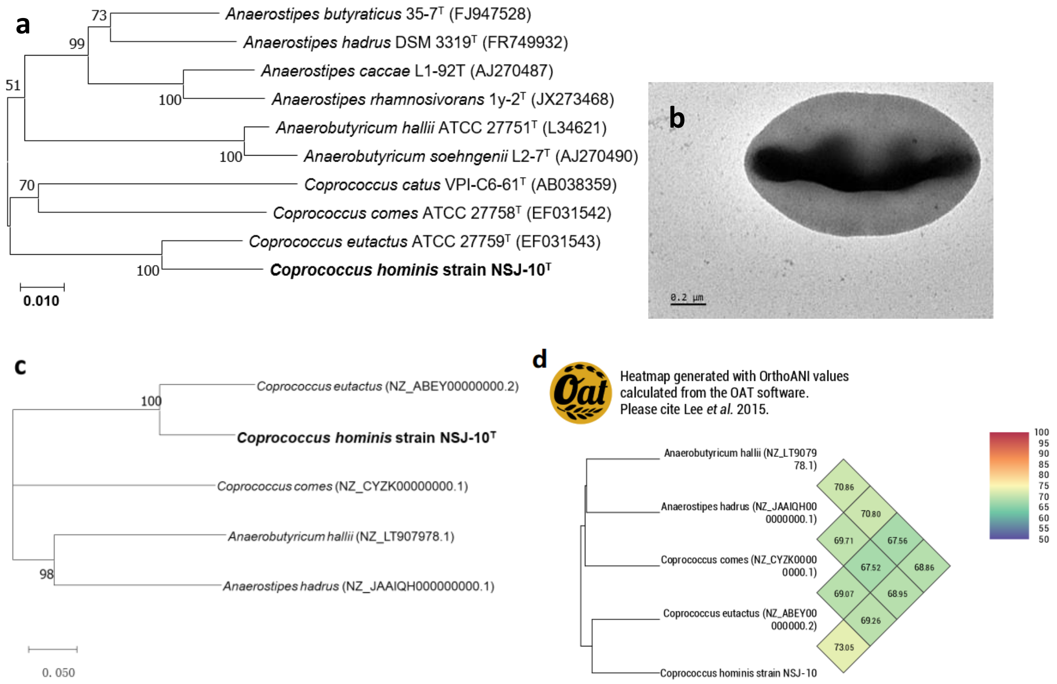

Figure SD-65. The Neighbour-joining phylogenetic tree based on 16S rRNA gene sequences (a) and the cell morphology (b) of strain NSJ-10^T. GenBank accession numbers are given in parentheses. Percentages of bootstrap support are shown at branch nodes. Bootstrap value was 1000. Bar: 0.01 substitutions per nucleotide position. The phylogenominc tree based on gemome-sequence-alignment (c) and OrthoANI heatmap (d) of strain NSJ-10^T and its neighbor species. Refseq ID of representative genomes are given in parentheses. Percentages of bootstrap support are shown at branch nodes. Bar: 0.050 substitutions per nucleotide position.

Taxon 66: *Dorea hominis* sp. nov.

Description *of Dorea hominis* sp. nov. (ho'mi.nis L. gen. masc. n. *hominis*, of a human being, referring to the human gut habitat)

The taxon 66, respresented by strain NSJ-36^T (16S rRNA sequence accession number in NMDC is NMDCN0000121), is phylogenetically closest to *Dorea longicatena*, with 16S rRNA gene identity of 97.5 %. Phylogenetic tree shows that strain NSJ-36^T clusters with other members of the genus *Dorea,* (Figure SD-66a), suggesting strain NSJ-36^T is a member of the genus *Dorea*. The genome of strain NSJ-36^T was sequenced and the NMDC accession number is NMDC60014063. Genome-based analysis showed that the ANI value of genomes of strain NSJ-36^T and *Dorea longicatena* DSM 13814^T (NZ AAXB00000000) is 83.25 % and the dDDH estimation is 27.40 %. According to the phylogenominc tree (Figure SD-66c), the ANIs and dDDH values of genomes between strain NSJ-36^T (NMDC accession number is NMDC60014063) and phylogenomically neighbored genomes were calculated, and the highest ANI and dDDH values are 76.2495 and 27.7 %, respectively, between the query genome of *Dorea formicigenerans* (NZ_QSFS00000000.1); and difference in % G+C between genomes of strain NSJ-36^T and *Dorea formicigenerans* (NZ_QSFS00000000.1) is 0.19; OrthoANI heatmap (Figure SD-66d) shows the phylogenomic status of the corresponding neighbor species based on OrthoANI values. Based on these results, we concluded that the strain NSJ-36^T represents of new species of the genus *Dorea*, and the name *Dorea hominis* sp. nov. is proposed.

Cells are strictly anaerobic, straight rods (1.6-2.4 µm long by 0.6–0.8 µm wide, Figure SD-66b); cells appear singly or in (dividing) pairs. Circular, cream-colored, flat-to-raised, entire colonies appear on modified mGAM agar plate after 3 days of incubation. Growth occurs at 37°C and at pH 7.0-7.5. The organism uses D-fructose, palatinose, L-rhamnose and glyoxylic acid as the carbon source for growth and weakly metabolizes α-D-glucose, maltotriose, 3-melthyl-D-glucose, turanose and L-serine. The DNA G+C content of the type strain NSJ-36^T is 51.43 mol %. The type strain NSJ-36^T (=CGMCC 1.32809^T) was isolated from the faeces of a healthy adult.

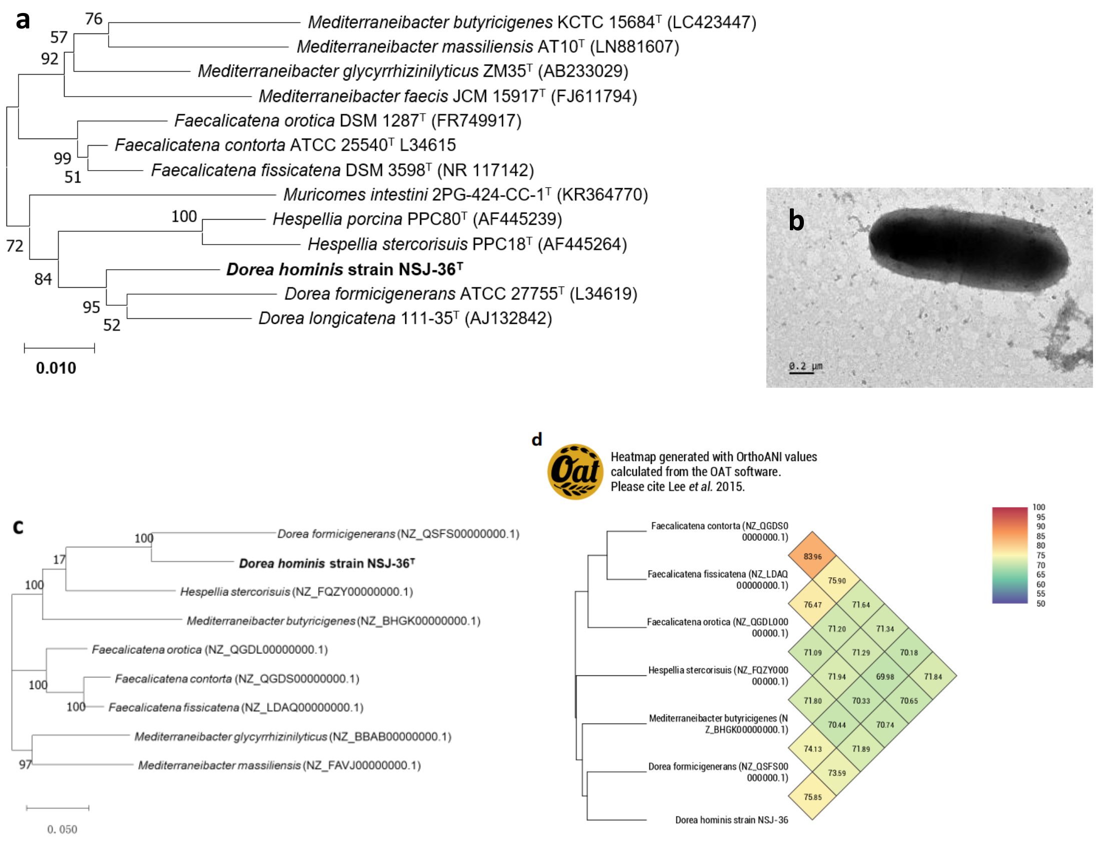

Figure SD-66. The Neighbour-joining phylogenetic tree based on 16S rRNA gene sequences (a) and the cell morphology (b) of strain NSJ-36^{T}. GenBank accession numbers are given in parentheses. Percentages of bootstrap support are shown at branch nodes. Bootstrap value was 1000. Bar: 0.01 substitutions per nucleotide position. The phylogenominc tree based on gemome-sequence-alignment (c) and OrthoANI heatmap (d) of strain NSJ-36^{T} and its neighbor species. Refseq ID of representative genomes are given in parentheses. Percentages of bootstrap support are shown at branch nodes. Bar: 0.050 substitutions per nucleotide position.

Taxon 67: *Enterocloster hominis* sp. nov.

Description *of Enterocloster hominis* sp. nov. (ho'mi.nis L. gen. masc. n. hominis, of a human being, referring to the human gut habitat)

The taxon 67, respresented by strain BX10^T (16S rRNA sequence accession number in NMDC is NMDCN0000122), is phylogenetically closest to *Enterocloster citroniae*, with 16S rRNA gene identity of 94.8 %. Phylogenetic tree shows that strain BX10^T clusters with other members of the genus *Enterocloster* (Figure SD-67a), suggesting strain BX10^T is a member of the genus *Enterocloster.* The genome of strain BX10^T was sequenced and the NMDC accession number is NMDC60014064. Genome-based analysis showed that the ANI value of genomes of strain BX10^T and *Enterocloster citroniae* RMA 16102^T (NZ ADLJ00000000) is 69.85 % and the dDDH estimation is 21.60 %. According to the phylogenominc tree (Figure SD-67c), the ANIs and dDDH values of genomes between strain BX10^T (NMDC accession number is NMDC60014064) and phylogenomically neighbored genomes were calculated, and the highest ANI and dDDH values are 70.7164 and 20.7 %, respectively, between the query genome of *Enterocloster aldensis* (NZ_QSGP00000000.1); and difference in % G+C between genomes of strain BX10^T and *Enterocloster aldensis* (NZ_QSGP00000000.1) is 3.44; OrthoANI heatmap (Figure SD-67d) shows the phylogenomic status of the corresponding neighbor species based on OrthoANI values. Based on these results, we concluded that the strain BX10^T represents of new species of the genus *Enterocloster*, and the name *Enterocloster hominis* sp. nov. is proposed.

Cells are strictly anaerobic, straight rods with peaked ends (1.4-2.6 µm long by 0.8–1.2 µm wide, Figure SD-66b); cells appear singly or in (dividing) pairs.Growth occurs at 37°C and at pH 7.0-7.5. The DNA G+C content of the type strain BX10^T is 52.60 mol %. The type strain BX10^T (=CGMCC 1.32891^T) was isolated from the faeces of a healthy adult.

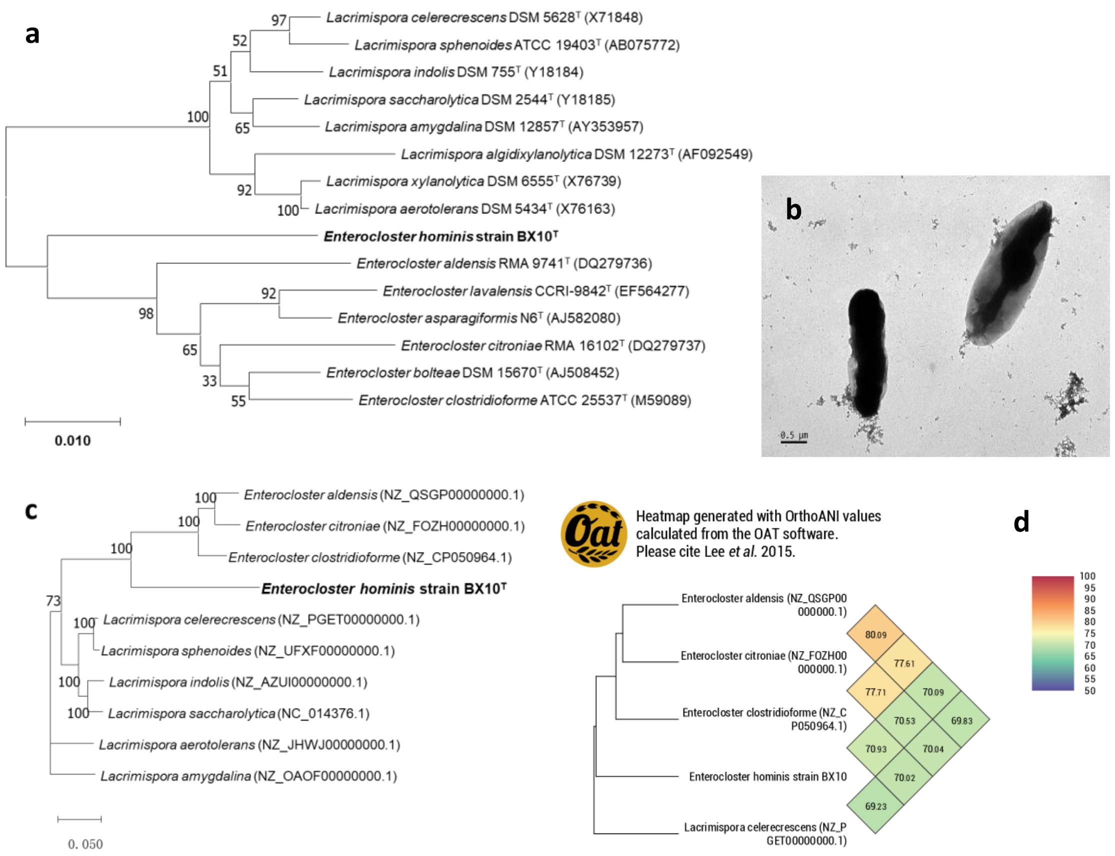

Figure SD-67. The Neighbour-joining phylogenetic tree based on 16S rRNA gene sequences (a) and the cell morphology (b) of strain BX10^T. GenBank accession numbers are given in parentheses. Percentages of bootstrap support are shown at branch nodes. Bootstrap value was 1000. Bar: 0.01 substitutions per nucleotide position. The phylogenominc tree based on gemome-sequence-alignment (c) and OrthoANI heatmap (d) of strain BX10^T and its neighbor species. Refseq ID of representative genomes are given in parentheses. Percentages of bootstrap support are shown at branch nodes. Bar: 0.050 substitutions per nucleotide position.

Taxon 68: *Eubacterium segne* sp. nov

Description of *Eubacterium segne* sp. nov. (seg'ne. L. neut. adj. *segne*, slow, inactive, lazy, referring the slow growth of the strain)
The taxon 68, represented by strain BX4^T (16S rRNA sequence accession number in NMDC is NMDCN0000123), is closest to *Eubacterium ventriosum*, with 16S rRNA gene identity of 97.1 %. Phylogenetic tree shows that strain BX4^T clusters with other members of the genus *Eubacterium* (Figure SD-68a), suggesting that strain BX4^T is a member of the genus *Eubacterium.* The genome of strain BX4^T was sequenced and the NMDC accession number is NMDC60014065. Genome-based analysis on *Eubacterium ventriosum* strain ATCC 27560^T (NZ AAVL00000000.) and type strain BX4^T reveals that the ANI value is 75.02 % and the dDDH estimation is 24.00 %. According to the phylogenominc tree (Figure SD-68c), the ANIs and dDDH values of genomes between strain BX4^T (NMDC accession number is NMDC60014065) and phylogenomically neighbored genomes were calculated, and the highest ANI and dDDH values are 75.39 and 24 %, respectively, between the query genome of *Eubacterium ventriosum* (NZ_QRHR00000000.1); and difference in % G+C between genomes of strain BX4^T and *Eubacterium ventriosum* (NZ_QRHR00000000.1) is 0.23; OrthoANI heatmap (Figure SD-68d) shows the phylogenomic status of the corresponding neighbor species based on OrthoANI values. Based on these results, we concluded that the strain BX4^T represents a new species of the genus *Eubacterium*, and the name *Eubacterium segne* sp.nov. is proposed.
Cells are strictly anaerobic, rod shaped (1.0-2.8 µm long by 0.7-0.9 µm wide, Figure SD-68b); cells appear singly. Growth occurs at 37°C and at pH 7.0-7.5. The DNA G+C content of the type strain BX4^T is 35.1 mol %. The type strain BX4^T (CGMCC 1.32892^T) was isolated from the faeces of a healthy adult.

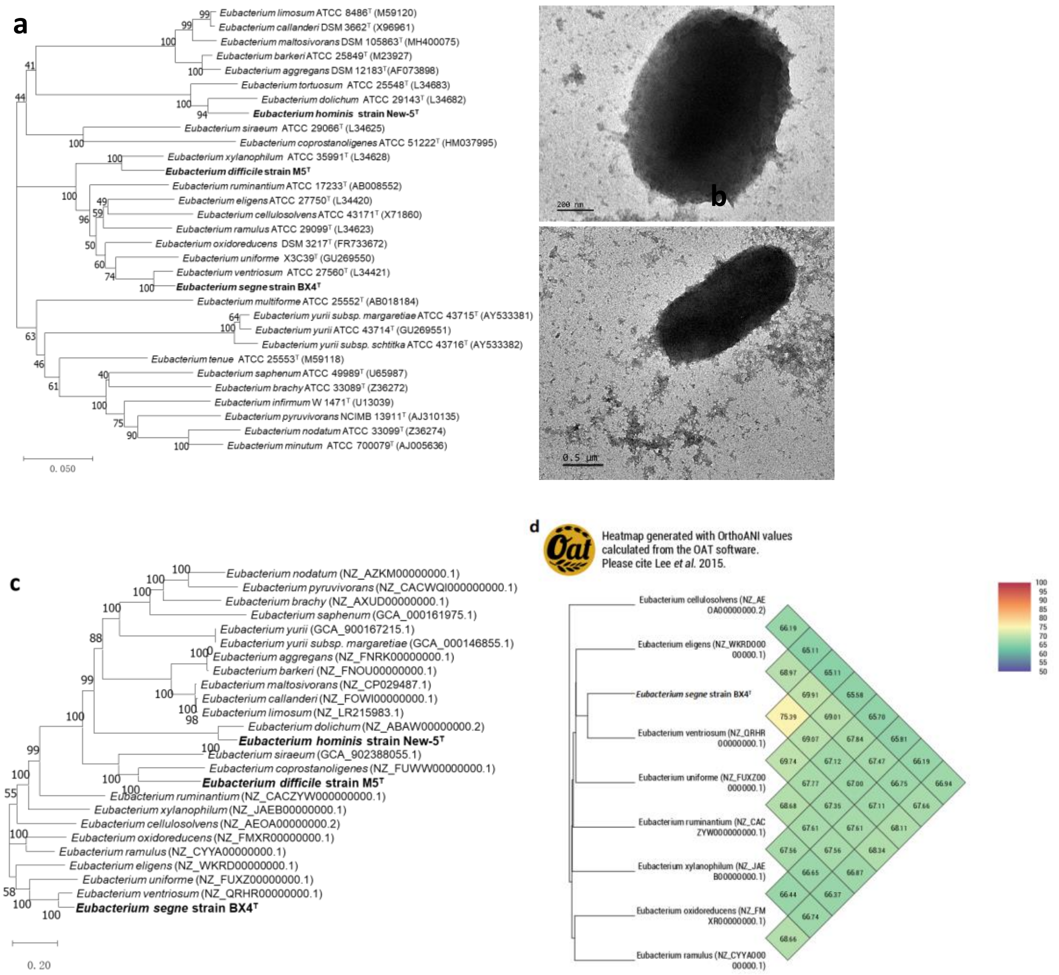

Figure SD-68. The Neighbour-joining phylogenetic tree based on 16S rRNA gene sequences (a) and the cell morphology (b) of strain BX4^T. GenBank accession numbers are given in parentheses. Percentages of bootstrap support are shown at branch nodes. Bootstrap value was 1000. Bar: 0.05 substitutions per nucleotide position. The phylogenominc tree based on gemome-sequence-alignment (c) and OrthoANI heatmap (d) of strain BX4^T and its neighbor species. Refseq ID of representative genomes are given in parentheses. Percentages of bootstrap support are shown at branch nodes. Bar: 0.20 substitutions per nucleotide position.

Taxon 69: *Eubacterium difficile* sp. nov.

Description of *Eubacterium difficile* sp. nov. (dif.fi'ci.le. L. neut. adj. *difficile*, difficult, referring the difficulty of culturing the strain)

The taxon 69, represented by strain M5^T (16S rRNA sequence accession number in NMDC is NMDCN0000124), is closest to *Eubacterium xylanophilum*, with 16S rRNA gene identity of 95.6 %. Phylogenetic tree shows that strain M5^T clusters with other members of the genus *Eubacterium* (Figure SD-69a), suggesting that strain M5^T is a member of the genus *Eubacterium.* The genome of strain M5^T was sequenced and the NMDC accession number is NMDC60014066. Genome-based analysis on *E. xylanophilum* strain ATCC 35991^T (NZ JAEB00000000) and type strain M5^T reveals that the ANI value is 62.98 % and the dDDH estimation is 60.30 %, the DNA G+C content difference is 3.65 mol %. According to the phylogenominc tree (Figure SD-69c), the ANIs and dDDH values of genomes between strain M5^T (NMDC accession number is NMDC60014066) and phylogenomically neighbored genomes were calculated, and the highest ANI and dDDH values are 65.36 and 18.1 %, respectively, between the query genome of *Eubacterium siraeum* (GCA_902388055.1); and difference in % G+C between genomes of strain M5^T and *Eubacterium siraeum* (GCA_902388055.1) is 6.34; OrthoANI heatmap (Figure SD-69d) shows the phylogenomic status of the corresponding neighbor species based on OrthoANI values. Based on these results, we concluded that the strain M5^T represents a new species of the genus *Eubacterium*, and the name *Eubacterium difficile* sp.nov. is proposed.

Cells are strictly anaerobic, curved or straight rods (2.5-3.6 µm long by 0.4-0.7 µm wide, Figure SD-69b). Growth occurs at 37°C and at pH 7.0-7.5. Cells appear singly or in dividing pairs. The organism uses amygdalin, dextrin, D-fructose, L-fucose, D-galactose, D-galacturonic acid, gentiobiose, D-gluconic acid, D-glucosaminic acid, α-D-glucose, glucose-6-phosphate, lactulose, D-mannose, glycerol, 3-melthyl-D-glucose, palatinose, L-rhamnose, turanose, acetic acid, glyoxylic acid, α-ketobutyric acid, pyruvic acid, pyruvic acidmethyl ester as the carbon source for growth, and weakly utilizes amygdalin, D-cellobiose, formic acid and D-saccharic acid. The DNA G+C content of the type strain M5^T is 51.20 mol %. The type strain is M5^T, which was isolated from the faeces of a healthy adult.

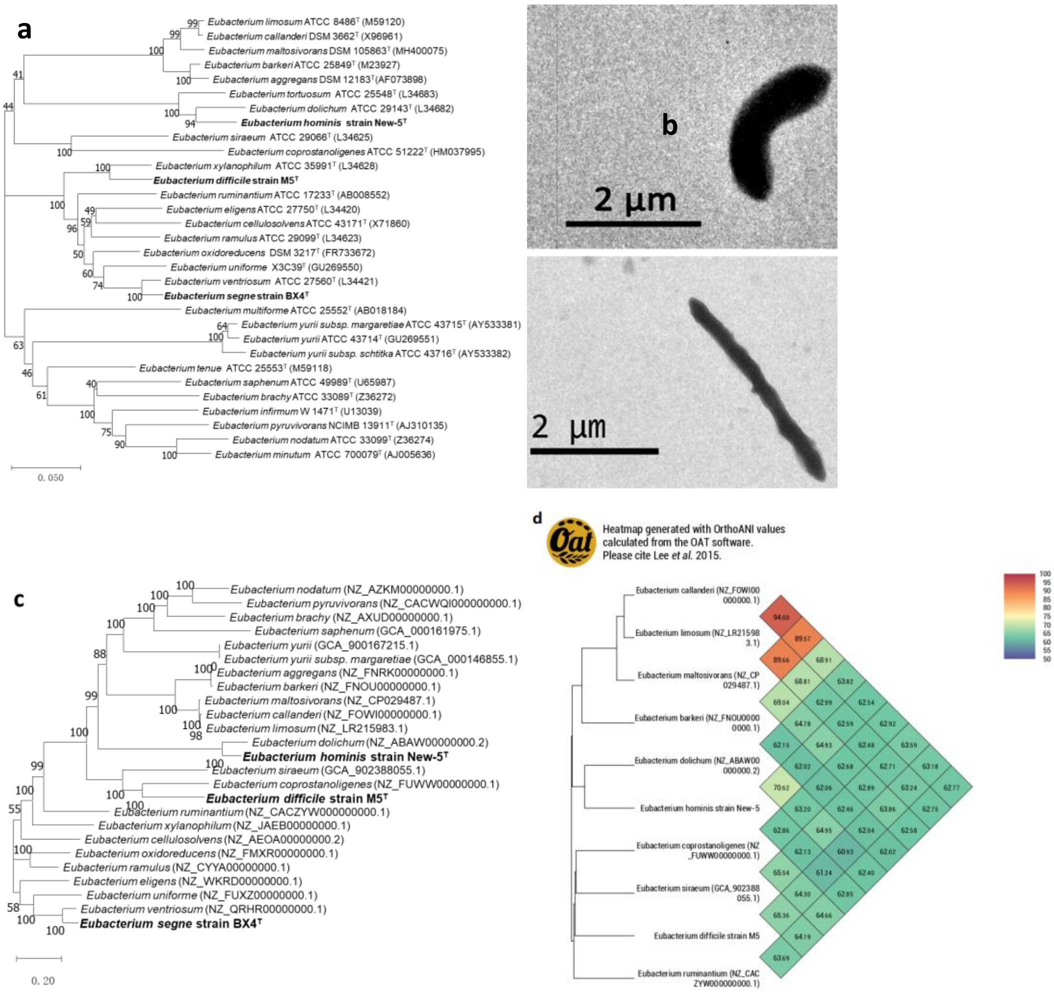

Figure SD-69. The Neighbour-joining phylogenetic tree based on 16S rRNA gene sequences (a) and the cell morphology (b) of strain M5^T. GenBank accession numbers are given in parentheses. Percentages of bootstrap support are shown at branch nodes. Bootstrap value was 1000. Bar: 0.05 substitutions per nucleotide position. The phylogenominc tree based on gemome-sequence-alignment (c) and OrthoANI heatmap (d) of strain M5^T and its neighbor species. Refseq ID of representative genomes are given in parentheses. Percentages of bootstrap support are shown at branch nodes. Bar: 0.2 substitutions per nucleotide position.

Taxon 70: *Hungatella hominis* sp. nov.

Description *of Hungatella hominis* sp. nov. (ho'mi.nis L. gen. masc. n. hominis, of a human being, referring to the human gut habitat)

The taxon 70, respresented by strain NSJ-66^T (16S rRNA sequence accession number in NMDC is NMDCN0000125), is phylogenetically closest to *Hungatella effluvii*, with 16S rRNA gene identity of 98.6 %. Phylogenetic tree shows that strain NSJ-66^T clusters with other members of the genus *Hungatella,* (Figure SD-70a), suggesting strain NSJ-66^T is a member of the genus *Hungatella*. The genome of strain NSJ-66^T was sequenced and the NMDC accession number is NMDC60014067. Genome-based analysis showed that the ANI value of genomes of strain NSJ-66^T and *Hungatella effluvii* UB-B.2^T (NZ QJKD00000000) is 95.21 % and the dDDH estimation is 64.30 %. According to the phylogenominc tree (Figure SD-70c), the ANIs and dDDH values of genomes between strain NSJ-66^T (NMDC accession number is NMDC60014067) and phylogenomically neighbored genomes were calculated, and the highest ANI and dDDH values are 86.88 and 33.6 %, respectively, between the query genome of *Hungatella hathewayi* (NZ_WNME00000000.1); and difference in % G+C between genomes of strain NSJ-66^T and *Hungatella hathewayi* (NZ_WNME00000000.1) is 0.52; OrthoANI heatmap (Figure SD-70d) shows the phylogenomic status of the corresponding neighbor species based on OrthoANI values. Based on these results, we concluded that the strain NSJ-66^T represents of new species of the genus *Hungatella*, and the name *Hungatella hominis* sp. nov. is proposed.

Cells are strictly anaerobic, fusiform rods with flagella (2.1-4.4 µm long by 1.2-1.6 µm wide, Figure SD-70b); cells appear singly or in (dividing) pairs. Circular, cream-colored, smooth, lightly raised, entire colonies appear on modified mGAM agar plate after 2 days of incubation. Growth occurs at 37°C and at pH 7.0-7.5. The organism uses dextrin amygdalin, m-tartaric acid and urocanic acid as the carbon source for growth, and weakly metabolizes acetyl-D glucosamine, arbutin, D-cellobiose, dulcitol, D-fructose, D-galacturonic acid, gentiobiose, m-Inositol, maltose, α-methyl-D-galactoside, glycyl-L-proline, thymidine-5'-monophosphate acetyl-D glucosamine, arbutin, D-cellobiose, dulcitol, D-fructose, D-galacturonic acid, gentiobiose, m-Inositol, maltose, α-methyl-D-galactoside, glycyl-L-proline and thymidine-5'-monophosphate. The DNA G+C content of the type strain NSJ-66^T is 52.65 mol %. The type strain NSJ-66^T (=CGMCC 1.32842^T =KCTC 25127^T) was isolated from the faeces of a healthy adult.

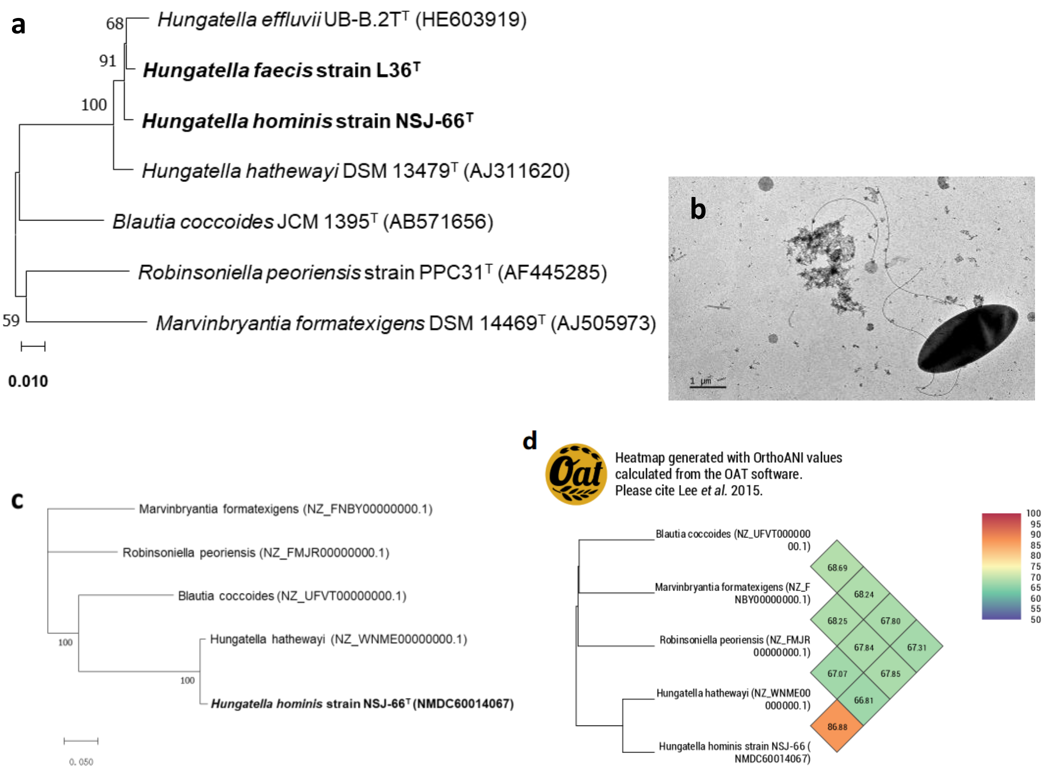

Figure SD-70. The Neighbour-joining phylogenetic tree based on 16S rRNA gene sequences (a) and the cell morphology (b) of strain NSJ-66^T. GenBank accession numbers are given in parentheses. Percentages of bootstrap support are shown at branch nodes. Bootstrap value was 1000. Bar: 0.01 substitutions per nucleotide position. The phylogenominc tree based on gemome-sequence-alignment (c) and OrthoANI heatmap (d) of strain NSJ-66^T and its neighbor species. Refseq ID of representative genomes are given in parentheses. Percentages of bootstrap support are shown at branch nodes. Bar: 0.050 substitutions per nucleotide position.

Taxon 72: *Lachnospira hominis* sp. nov.

Description *of Lachnospira hominis* sp. nov. (ho'mi.nis. L. gen. masc. n. *hominis*, of a human being, referring to the human gut habitat)
The taxon 72, respresented by strain NSJ-43^T (16S rRNA sequence accession number in NMDC is NMDCN0000127), is phylogenetically closest to *Lachnospira pectinoschiza*, with 16S rRNA gene identity of 96.6 %. Phylogenetic tree shows that strain NSJ-43^T clusters with other members of the genus *Lachnospira,* (Figure SD-72a), suggesting strain NSJ-43^T is a member of the genus *Lachnospira*. The genome of strain NSJ-43^T was sequenced and the NMDC accession number is NMDC60014069. Genome-based analysis showed that the ANI value of genomes of strain NSJ-43^T and *Lachnospira pectinoschiza* 150-1^T (NZ FNHZ00000000) is 77.99 % and the dDDH estimation is 25.00 %. According to the phylogenominc tree (Figure SD-72c), the ANIs and dDDH values of genomes between strain NSJ-43^T (NMDC accession number is NMDC60014069) and phylogenomically neighbored genomes were calculated, and the highest ANI and dDDH values are 69.05 and 31 %, respectively, between the query genome of *Coprococcus eutactus* (GCF_000154425.1); and difference in % G+C between genomes of strain NSJ-43^T and *Coprococcus eutactus* (GCF_000154425.1) is 6.5; OrthoANI heatmap (Figure SD-72d) shows the phylogenomic status of the corresponding neighbor species based on OrthoANI values. Based on these results, we concluded that the strain NSJ-43^T represents of new species of the genus *Lachnospira*, and the *Lachnospira hominis* sp. nov. is proposed.
Cells are strictly anaerobic straight rods with peritrichous flagella (3.2-5.0 µm long by 0.5-0.7 µm wide, Figure SD-72b). Tiny, circular, brown, raised, entire colonies appear on modified mGAM agar plate after 3 days of incubation. Growth occurs at 37°C and at pH 7.0-7.5. The organism uses D-fructose, L-fucose, D-galactose, D-galacturonic acid, α-D-glucose, D-mannose, 3-melthyl-D-glucose, palatinose, L-rhamnose and pyruvic acid as carbon source for growth, and weakly metabolizes α-ketobutyric acid. The DNA G+C content of the type strain NSJ-43^T is 52.93 mol %. The type strain NSJ-43^T (=CGMCC 1.32816^T) was isolated from the faeces of a healthy adult.

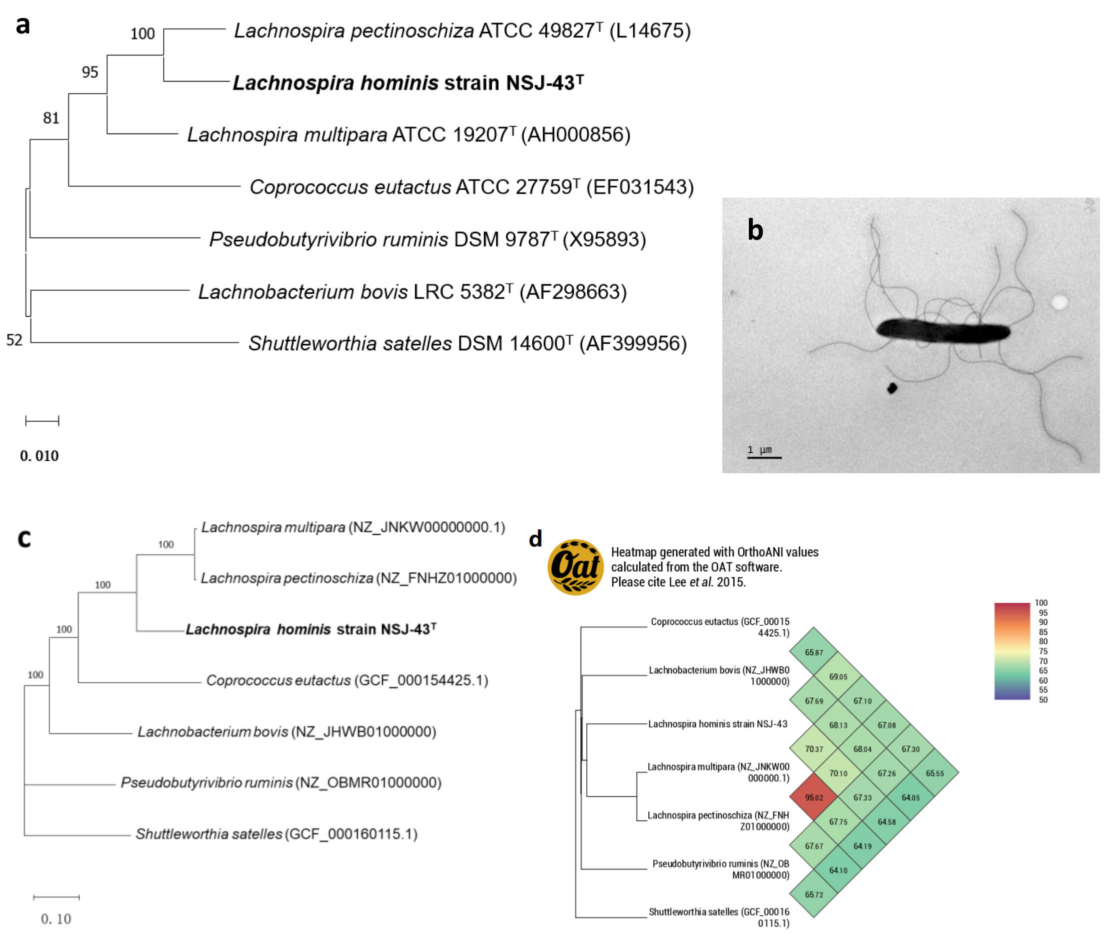

Figure SD-72. The Neighbour-joining phylogenetic tree based on 16S rRNA gene sequences (a) and the cell morphology (b) of strain NSJ-43^T. GenBank accession numbers are given in parentheses. Percentages of bootstrap support are shown at branch nodes. Bootstrap value was 1000. Bar: 0.01 substitutions per nucleotide position. The phylogenominc tree based on gemome-sequence-alignment (c) and OrthoANI heatmap (d) of strain NSJ-43^T and its neighbor species. Refseq ID of representative genomes are given in parentheses. Percentages of bootstrap support are shown at branch nodes. Bar: 0.10 substitutions per nucleotide position.

Taxon 73: *Ruminococcus hominis* sp. nov.

Description of *Ruminococcus hominis* sp. nov. (ho'mi.nis. L. gen. masc. n. *hominis*, of a human being, referring to the human gut habitat)

The taxon 73, represented by strain NSJ-13^T (16S rRNA sequence accession number in NMDC is NMDCN0000128), is closest to *Ruminococcus lactaris*, with 16S rRNA gene identity of 97.2 %. Phylogenetic tree shows that strain NSJ-13^T clusters with other members of the genus *Ruminococcus* (Figure SD-73a), suggesting that strain NSJ-13^T is a member of the genus *Ruminococcus.* The genome of strain NSJ-13^T was sequenced and the NMDC accession number is NMDC60014070. Genome-based analysis on *Ruminococcus lactaris* strain ATCC 29176^T (NZ ABOU00000000) and type strain NSJ-13^T reveals that the ANI value is 74.51 % and the dDDH estimation is 24.50. According to the phylogenominc tree (Figure SD-73c), the ANIs and dDDH values of genomes between strain NSJ-13^T (NMDC accession number is NMDC60014070) and phylogenomically neighbored genomes were calculated, and the highest ANI and dDDH values are 73.75 and 24.5 %, respectively, between the query genome of *Ruminococcus lactaris* (NZ_DS990209); and difference in % G+C between genomes of strain NSJ-13^T and *Ruminococcus lactaris* (NZ_DS990209) is 4.1; OrthoANI heatmap (Figure SD-73d) shows the phylogenomic status of the corresponding neighbor species based on OrthoANI values. Based on these results, we concluded that the strain NSJ-13^T represents a new species of the genus *Ruminococcus*, and the name *Ruminococcus hominis sp. nov.* is proposed.

Cells are strictly anaerobic, spiral shaped and vibrio or rod shaped cells are also observed (2.6-5.2 µm long by 0.6-0.8 µm wide Figure SD-73b); cells appear singly or in dividing pairs, dividing cells are equal in sizes and mostly in oval shaped rather than firmly spherical. Circular to irregular, ivory, flat colonies appear on modified mGAM agar plate after 3 days of incubation. The organism uses L-fucose, palatinose and inosine as the carbon source for growth, and weakly metabolizes dextrin, D-fructose,D-galacturonic acid, 3-melthyl-D-glucose, L-rhamnose, glyoxylic acid and pyruvic acid. The DNA G+C content of the type strain NSJ-13^T is 59.53 mol %. The type strain is NSJ-13^T (=CGMCC 1.5249^T), which was isolated from the faeces of a healthy adult.

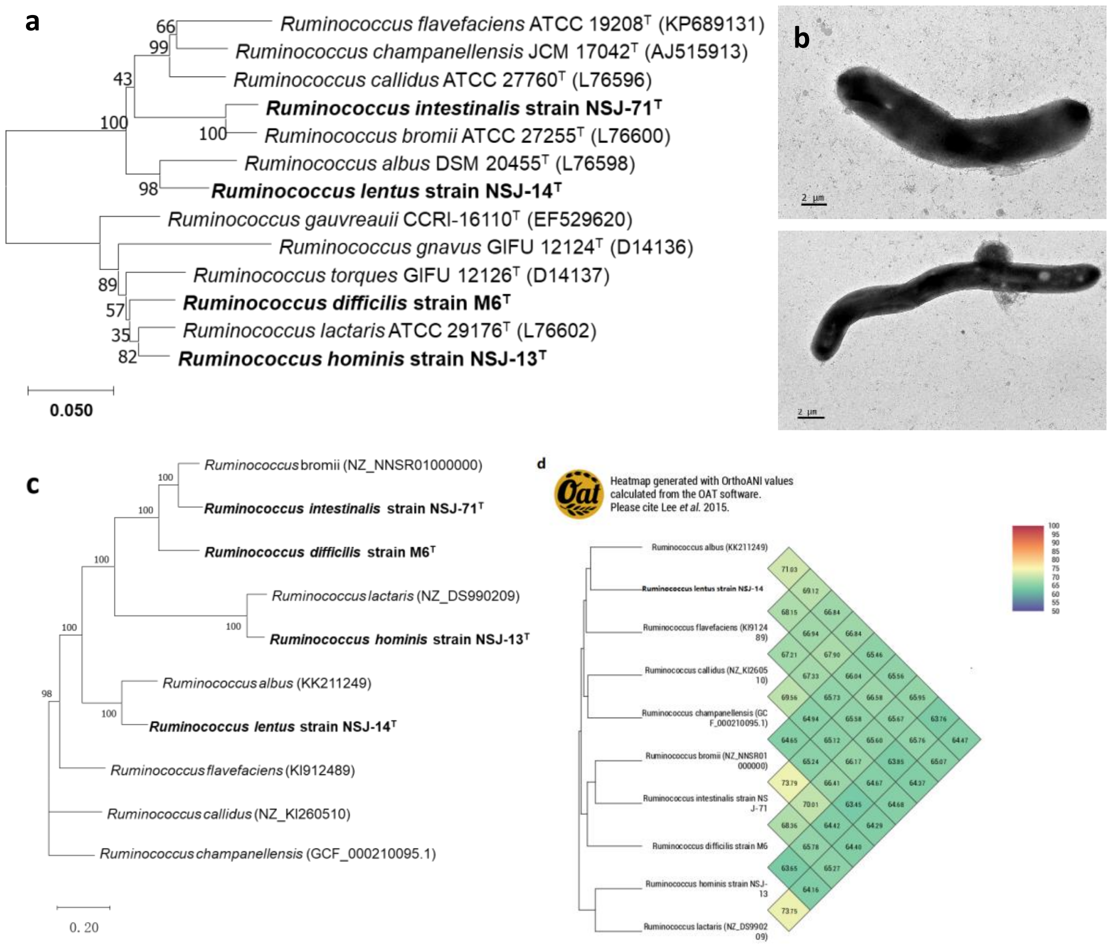

Figure SD-73. The Neighbour-joining phylogenetic tree based on 16S rRNA gene sequences (a) and the cell morphology (b) of strain NSJ-13^T. GenBank accession numbers are given in parentheses. Percentages of bootstrap support are shown at branch nodes. Bootstrap value was 1000. Bar: 0.05 substitutions per nucleotide position. The phylogenominc tree based on gemome-sequence-alignment (c) and OrthoANI heatmap (d) of strain NSJ-13^T and its neighbor species. Refseq ID of representative genomes are given in parentheses. Percentages of bootstrap support are shown at branch nodes. Bar: 0.20 substitutions per nucleotide position.

Taxon 74: *Mediterraneibacter hominis* sp. nov.

Description of *Mediterraneibacter hominis* sp. nov. (ho'mi.nis. L. gen. masc. n. *hominis*, of a human being, referring to the human gut habitat)
The taxon 74, represented by strain NSJ-55^T (16S rRNA sequence accession number in NMDC is NMDCN0000129), is closest to *Mediterraneibacter massiliensis*, with 16S rRNA gene identity of 95.5 %. Phylogenetic tree shows that strain NSJ-55^T clusters with other members of the genus *Mediterraneibacter* (Figure SD-74a), suggesting that strain NSJ-55^T is a member of the genus *Mediterraneibacter.* The genome of strain NSJ-55^T was sequenced and the NMDC accession number is NMDC60014071. Genome-based analysis on *Mediterraneibacter massiliensis* strain AT10^T (NZ FAVJ00000000) and type strain NSJ-55^T reveals that the ANI value is 78.71 % and the dDDH estimation is 22.00 %. According to the phylogenominc tree (Figure SD-74c), the ANIs and dDDH values of genomes between strain NSJ-55^T (NMDC accession number is NMDC60014071) and phylogenomically neighbored genomes were calculated, and the highest ANI and dDDH values are 79.04 and 22 %, respectively, between the query genome of *Mediterraneibacter massiliensis* (GCF_001487105.1); and difference in % G+C between genomes of strain NSJ-55^T and *Mediterraneibacter massiliensis* (GCF_001487105.1) is 0.95; OrthoANI heatmap (Figure SD-74d) shows the phylogenomic status of the corresponding neighbor species based on OrthoANI values. Based on these results, we concluded that the strain NSJ-55^T represents a new species of the genus *Mediterraneibacter*, and the name *Mediterraneibacter hominis* sp. nov. is proposed.
Cells are strictly anaerobic, rod shaped with slightly peaked ends (1.1-1.9 µm long by 0.4-0.8 µm wide, Figure SD-74b). Growth occurs at 37°C and at pH 7.0-7.5. The organism uses N-acetyl-D galactosamine, N-acetyl-D glucosamine, adonitol, D-cellobiose, N-acetyl-β-D mannosamine, dextrin, D-fructose, L-fucose, D-galactose, D-galacturonic acid, gentiobiose, D-gluconic acid, D-glucosaminic acid, α-D-glucose, glucose-6-phosphate, lactulose, maltose, D-mannose, D-melezitose, D-melibiose, 3-melthyl-D-glucose, α-methyl-D-glucoside, D-raffinose, L-rhamnose, salicin, D-sorbitol, palatinose, stachyose, sucrose, turanose, acetic acid, formic acid, glyoxylic acid, α-ketobutyric acid, a-ketovaleric acid, propionic acid, pyruvic acid, pyruvic acid methyl ester, 2'-deoxy adenosine, inosine, thymidine and uridine as the carbon source for growth, and weakly metabolizes D-arabitol, i-erythritol i-erythritol, glycerol, maltotriose, D-trehalose and D-saccharic acid. The DNA G+C content of the type strain is 41.5 mol %. The type strain is NSJ-55^T (=CGMCC 1.32831^T), which was isolated from the faeces of a healthy adult.

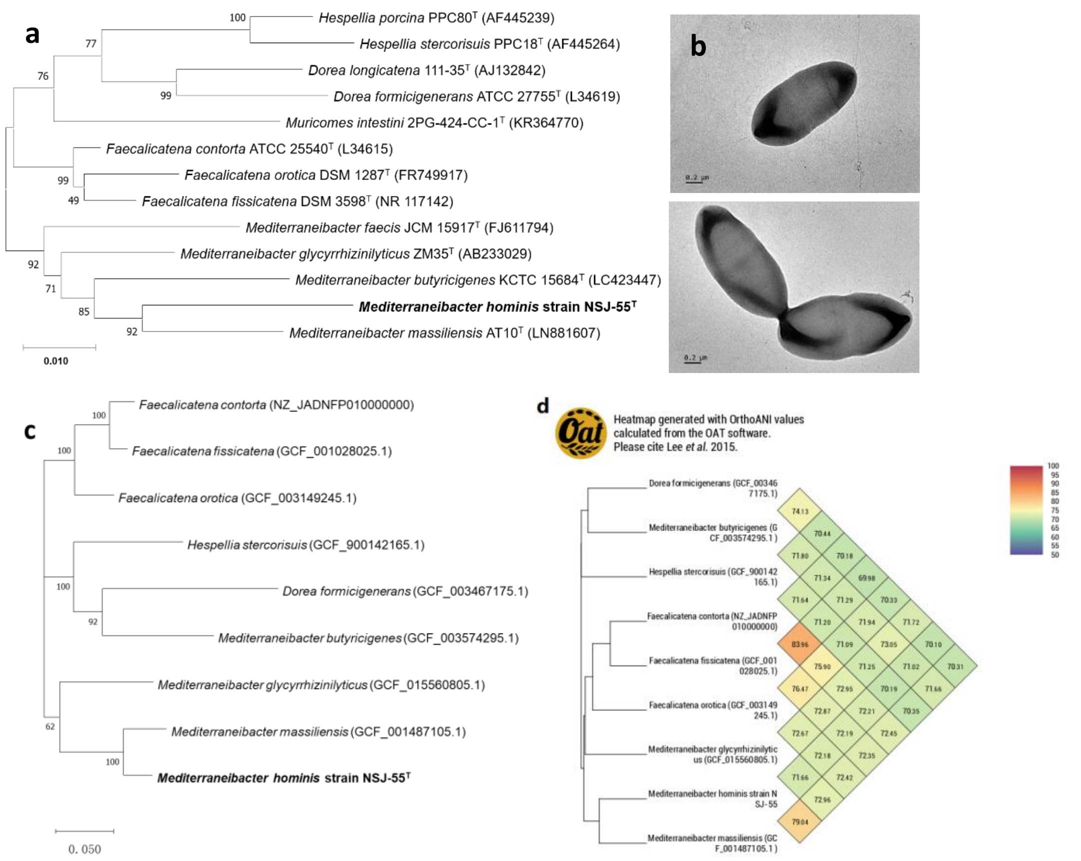

Figure SD-74. The Neighbour-joining phylogenetic tree based on 16S rRNA gene sequences (a) and the cell morphology (b) of strain NSJ-55^T. GenBank accession numbers are given in parentheses. Percentages of bootstrap support are shown at branch nodes. Bootstrap value was 1000. Bar: 0.01 substitutions per nucleotide position. The phylogenominc tree based on gemome-sequence-alignment (c) and OrthoANI heatmap (d) of strain NSJ-55^T and its neighbor species. Refseq ID of representative genomes are given in parentheses. Percentages of bootstrap support are shown at branch nodes. Bar: 0.050 substitutions per nucleotide position.

Taxon 75: *Ruminococcus difficilis* sp. nov.

Description of *Ruminococcus difficilis* sp. nov. (dif.fi'ci.lis. L. masc. adj. *difficilis*, difficult, referring the difficulty of culturing the strain)
The taxon 75, represented by strain M6^T (16S rRNA sequence accession number in NMDC is NMDCN000012A), is closest to *Ruminococcus lactaris*, with 16S rRNA gene identity of 95.2 %. Phylogenetic tree shows that strain M6^T clusters with other members of the genus *Ruminococcus* (Figure SD-75a), suggesting that strain M6^T is a member of the genus *Ruminococcus.* The genome of strain M6^T was sequenced and the NMDC accession number is NMDC60014072. Genome-based analysis on *Ruminococcus lactaris* strain ATCC 29176^T (NZ ABOU00000000) and type strain M6^T reveals that the ANI value is 73.53 % and the dDDH estimation is 39.90 %. According to the phylogenominc tree (Figure SD-75c), the ANIs and dDDH values of genomes between strain M6^T (NMDC accession number is NMDC60014072) and phylogenomically neighbored genomes were calculated, and the highest ANI and dDDH values are 70.01 and 27.5 %, respectively, between the query genome of *Ruminococcus bromii* (NZ_NNSR01000000); and difference in % G+C between genomes of strain M6^T and Ruminococcus bromii (NZ_NNSR01000000) is 11.22; OrthoANI heatmap (Figure SD-75d) shows the phylogenomic status of the corresponding neighbor species based on Ortho-ANI values. Based on these results, we concluded that the strain M6^T represents a new species of the genus *Ruminococcus*, and the name *Ruminococcus difficilis* sp.nov. is proposed.
Cells are strictly anaerobic and straight or slightly curved rods with peritrichous flagella (2.6-5.2 μm long by 0.6-0.8 μm wide, Figure SD-75b). The organism uses D-fructose, L-fucose, D-galactose, D-galacturonic acid, gentiobiose, α-D-glucose, glucose-6-phosphate, D-mannose, D-melibiose, 3-melthyl-D-glucose, palatinose, L-rhamnose, glyoxylic acid, pyruvic acid and pyruvic acidmethyl ester as the carbon source for growth, and weakly metabolizes dextrin and D-gluconic acid. The DNA G+C content of the type strain M6^T is 53.90 mol %. The type strain is M6^T (=CGMCC 1.32867^T), which was isolated from the faeces of a healthy adult.

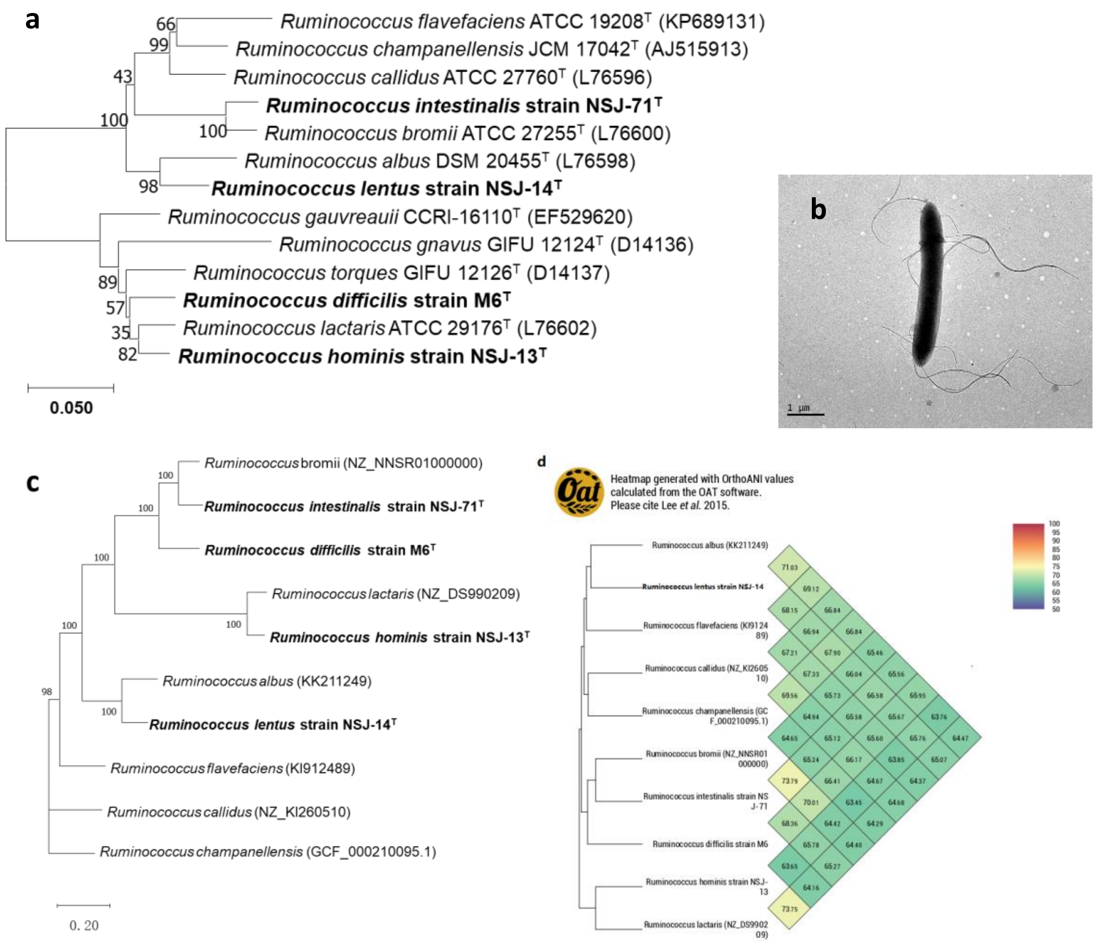

Figure SD-75. The Neighbour-joining phylogenetic tree based on 16S rRNA gene sequences (a) and the cell morphology (b) of strain M6^T. GenBank accession numbers are given in parentheses. Percentages of bootstrap support are shown at branch nodes. Bootstrap value was 1000. Bar: 0.05 substitutions per nucleotide position. The phylogenominc tree based on gemome-sequence-alignment (c) and OrthoANI heatmap (d) of strain M6^T and its neighbor species. Refseq ID of representative genomes are given in parentheses. Percentages of bootstrap support are shown at branch nodes. Bar: 0.20 substitutions per nucleotide position.

Taxon 76: *Roseburia lenta* sp. nov.

Description of *Roseburia lenta* sp. nov. (len'ta. L. fem. adj. *lenta*, slow, referring to the slow growth of the type strain)

The taxon 76, represented by strain NSJ-9^T (16S rRNA sequence accession number in NMDC is NMDCN000012B), is closest to *Roseburia hominis*, with 16S rRNA gene identity of 94.9 %. Phylogenetic tree shows that strain NSJ-9^T clusters with other members of the genus *Roseburia* (Figure SD-76a), suggesting that strain NSJ-9^T is a member of the genus *Roseburia.* The genome of strain NSJ-9^T was sequenced and the NMDC accession number is NMDC60014073. Genome-based analysis on *Roseburia hominis* strain A2-183^T (NC 015977) and type strain NSJ-9^T reveals that the ANI value is 70.18 % and the dDDH estimation is 23.00 %. According to the phylogenominc tree (Figure SD-76c), the ANIs and dDDH values of genomes between strain NSJ-9^T (NMDC accession number is NMDC60014073) and phylogenomically neighbored genomes were calculated, and the highest ANI and dDDH values are 71.01 and 27.4 %, respectively, between the query genome of *Roseburia zhanii* strain BX1005^T (NMDC60014075); and difference in % G+C between genomes of strain NSJ-9^T and *Roseburia zhanii* strain BX1005^T (NMDC60014075) is 4.55; OrthoANI heatmap (Figure SD-76d) shows the phylogenomic status of the corresponding neighbor species based on OrthoANI values. Based on these results, we concluded that the strain NSJ-9^T represents a new species of the genus *Roseburia*, and the name *Roseburia lenta sp.*nov. is proposed.

Cells are strictly anaerobic, straight rods or comma-shaped short rods or long, thin rods with peritrichous flegella (2.1-22.9 μm long by 0.4-0.6 μm wide, Figure SD-76b); cells appear singly, in dividing pairs or in dividing chains. Pinpoint, ivory, dry, slightly raised, entire colonies appear on modified mGAM agar plate after 4 days of incubation. Growth occurs at 37°C and at pH range of 7.0-7.5. The organism uses D-cellobiose, dextrin, D-fructose, L-fucose, D-galactose, D-galacturonic acid, gentiobiose, α-D-glucose, glucose-6-phosphate, lactulose, D-mannose, D-melibiose, 3-melthyl-D-glucose, palatinose, L-rhamnose, salicin, turanose, glyoxylic acid, α-ketobutyric acid, pyruvic acid and pyruvic acidmethyl ester as carbon source for growth, and weakly metabolizes maltotriose, ß-methyl-D-galactoside, D-raffinose, D-sorbitol, stachyose and sucrose. DNA G+C content of the type strain NSJ-9^T is 44.90 mol %. The type strain is NSJ-9^T (=CGMCC 1.32469^T), which was isolated from the faeces of a healthy adult.

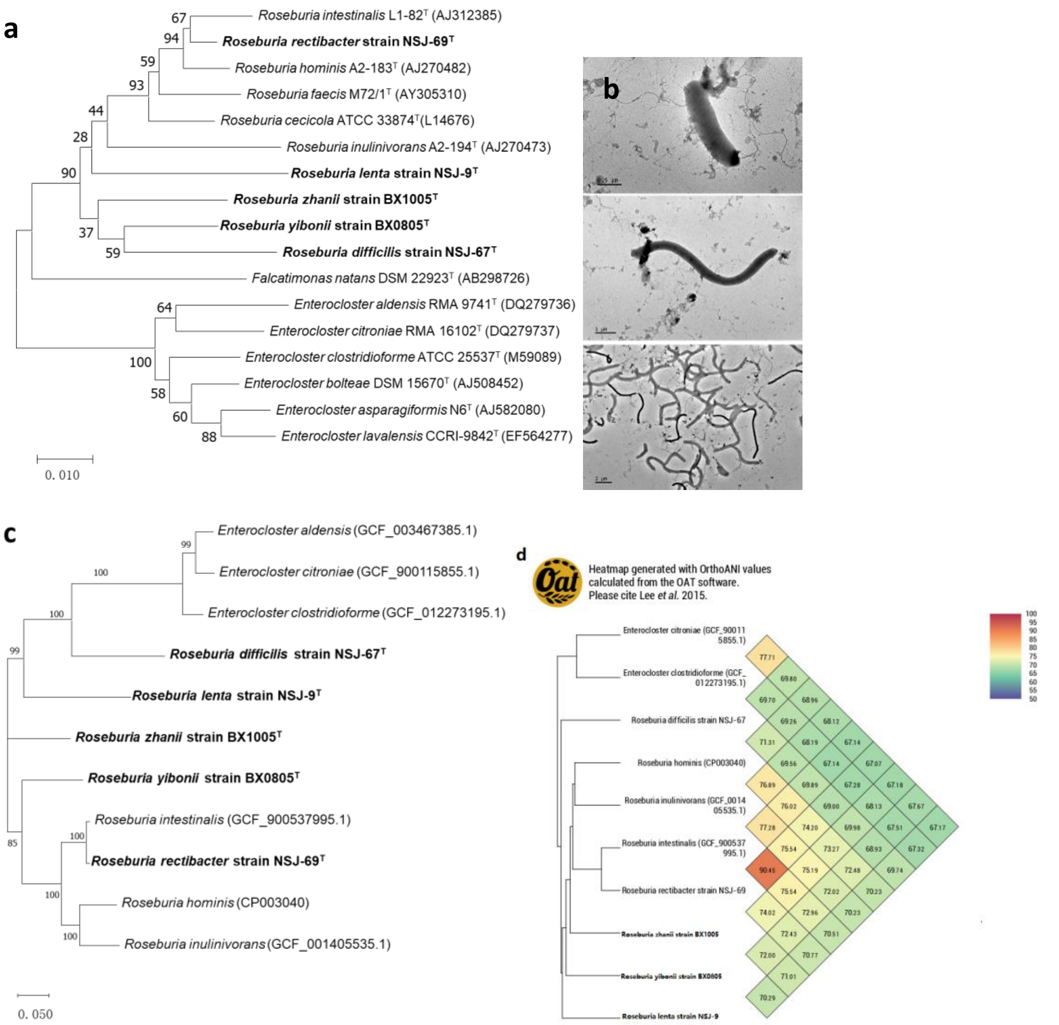

Figure SD-76. The Neighbour-joining phylogenetic tree based on 16S rRNA gene sequences (a) and the cell morphology (b) of strain NSJ-9^T. GenBank accession numbers are given in parentheses. Percentages of bootstrap support are shown at branch nodes. Bootstrap value was 1000. Bar: 0.01 substitutions per nucleotide position. The phylogenominc tree based on gemome-sequence-alignment (c) and OrthoANI heatmap (d) of strain NSJ-9^T and its neighbor species. Refseq ID of representative genomes are given in parentheses. Percentages of bootstrap support are shown at branch nodes. Bar: 0.050 substitutions per nucleotide position.

Taxon 77: *Roseburia yibonii* sp. nov.

Description of *Roseburia yibonii* sp. nov. (yi.bon'i.i N.L. gen. masc. n. *yibonii*, referring to Chinese actor Wang Yibon, whose series inspired the researcher during the bacterial identification)

The taxon 77, represented by strain BX0805^T (16S rRNA sequence accession number in NMDC is NMDCN000012C), is closest to *Roseburia hominis*, with 16S rRNA gene identity of 95.1 %. Phylogenetic tree shows that strain BX0805^T clusters with other members of the genus *Roseburia* (Figure SD-77a), suggesting that strain BX0805^T is a member of the genus *Roseburia.* The genome of strain BX0805^T was sequenced and the NMDC accession number is NMDC60014074. Genome-based analysis on *Roseburia hominis* A2-183^T (NC 015977) and type strain BX0805^T reveals that the ANI value is 67.20 % and the dDDH estimation is 23.30 %. According to the phylogenominc tree (Figure SD-77c), the ANIs and dDDH values of genomes between strain BX0805^T (NMDC accession number is NMDC60014074) and phylogenomically neighbored genomes were calculated, and the highest ANI and dDDH values are 72.96 and 24.5 %, respectively, between the query genome of *Roseburia intestinalis* (GCF_900537995.1); and difference in % G+C between genomes of strain BX0805^T and *Roseburia intestinalis* (GCF_900537995.1) is 3.75; OrthoANI heatmap (Figure SD-77d) shows the phylogenomic status of the corresponding neighbor species based on OrthoANI values. Based on these results, we concluded that the strain BX0805^T represents a new species of the genus *Roseburia*, and the name *Roseburia yibonii* sp.nov. is proposed.

Cells are strictly anaerobic, slightly curved rod-shaped or vibrio-shaped with spiky ends and cells with clavated ends are also observed (2.2-3.1 µm long by 0.5-0.8 µm wide, Figure SD-77b); cells appear singly or in dividing pairs. The organism uses D-fructose, L-fucose, D-galactose, D-galacturonic acid, gentiobiose, α-D-glucose, 3-melthyl-D-glucose, glucose-6-phosphate,lactulose, D-mannose, D-melibiose, palatinose, L-rhamnose, turanose, glyoxylic acid,pyruvic acid and pyruvic acid methyl ester as the carbon source for growth as the carbon source for growth, and weakly metabolizes N-acetyl-β-D mannosamine, arbutin, α-cyclodextrin, dextrin, i-erythritol, D-gluconic acid, glucose-1-phosphate, a-D-lactose, a-methyl-D-galactoside, ß-methyl-D-galactoside, D-trehalose, acetic acid, propionic acid and thymidine. The DNA G+C content of the type strain BX0805^T is 40.30 mol %. The type strain is BX0805^T (=CGMCC 1.32827^T), which was isolated from the faeces of a healthy adult.

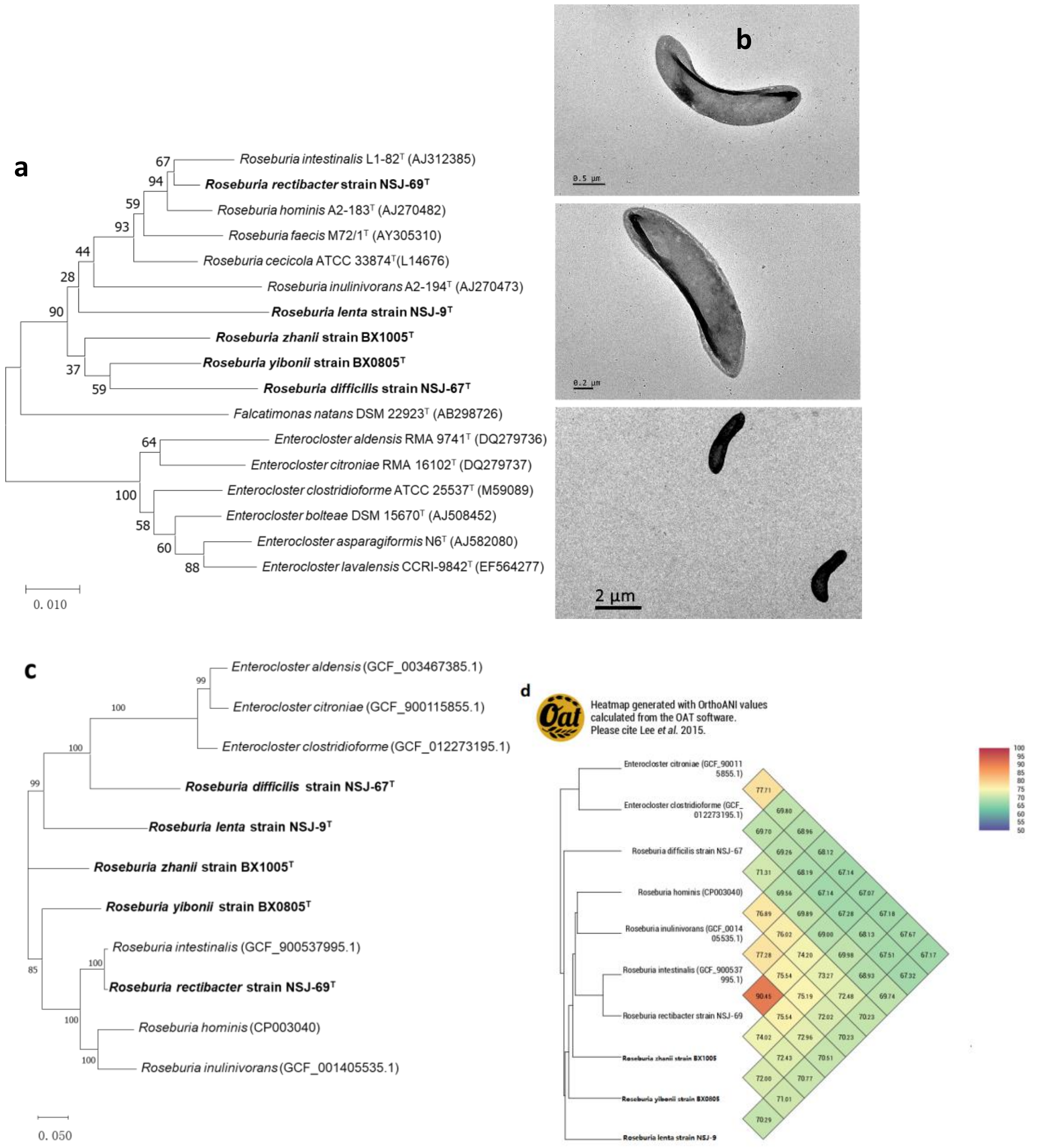

Figure SD-77. The Neighbour-joining phylogenetic tree based on 16S rRNA gene sequences (a) and the cell morphology (b) of strain BX0805^T. GenBank accession numbers are given in parentheses. Percentages of bootstrap support are shown at branch nodes. Bootstrap value was 1000. Bar: 0.01 substitutions per nucleotide position. The phylogenominc tree based on gemome-sequence-alignment (c) and OrthoANI heatmap (d) of strain BX0805^T and its neighbor species. Refseq ID of representative genomes are given in parentheses. Percentages of bootstrap support are shown at branch nodes. Bar: 0.05 substitutions per nucleotide position.

Taxon 78: *Roseburia zhanii* sp. nov.

Description of *Roseburia zhanii* sp. nov. (zha'ni.i N.L. gen. masc. n. *zhanii*, of Zhan, referring to Zhan Xiao, a Chinese actor whose series inspired the researcher during the bacterial identification)

The taxon 78, represented by strain BX1005^T (16S rRNA sequence accession number in NMDC is NMDCN000012D), is closest to *Roseburia cecicola*, with 16S rRNA gene identity of 95.7 %. Phylogenetic tree shows that strain BX1005^T clusters with other members of the genus *Roseburia* (Figure SD-78a), suggesting that strain BX1005^T is a member of the genus *Roseburia.* The genome of strain BX1005^T was sequenced and the NMDC accession number is NMDC60014075. The genomic sequence of *Roseburia cecicola* is not availale, thus, Genome-based analysis on the second closely neighboured strain *Roseburia faecis* strain DSM 16840^T (NZ CYXV00000000) and type strain BX1005^T reveals that the ANI value is 72.35 % and the dDDH estimation is 31.10 %. According to the phylogenominc tree (Figure SD-78c), the ANIs and dDDH values of genomes between strain BX1005^T (NMDC accession number is NMDC60014075) and phylogenomically neighbored genomes were calculated, and the highest ANI and dDDH values are 75.54 and 34.6 %, respectively, between the query genome of *Roseburia intestinalis* (GCF_900537995.1); and difference in % G+C between genomes of strain BX1005^T and *Roseburia intestinalis* (GCF_900537995.1) is 2.3; OrthoANI heatmap (Figure SD-78d) shows the phylogenomic status of the corresponding neighbor species based on OrthoANI values. Based on these results, we concluded that the strain BX1005^T represents a new species of the genus *Roseburia*, and the name *Roseburia zhanii* sp.nov. is proposed.

Cells are strictly anaerobic, straight rod shaped with round ends and peritrichous flagella (2.5-5.9 µm long by 0.9-1.5 µm wide, Figure SD-78b); The organism uses D-fructose, L-fucose, D-galactose, D-galacturonic acid, α-D-glucose, D-mannose, 3-melthyl-D-glucose, palatinose, L-rhamnose and pyruvic acid as the carbon source for growth as the carbon source for growth, and weakly metabolizes gentiobiose, D-glucosaminic acid, D-melibiose and glyoxylic acid. The DNA G+C content of the type strain BX1005^T is 40.30 mol %. The type strain is BX1005^T (=CGMCC 1.32828^T), which was isolated from the faeces of a healthy adult.

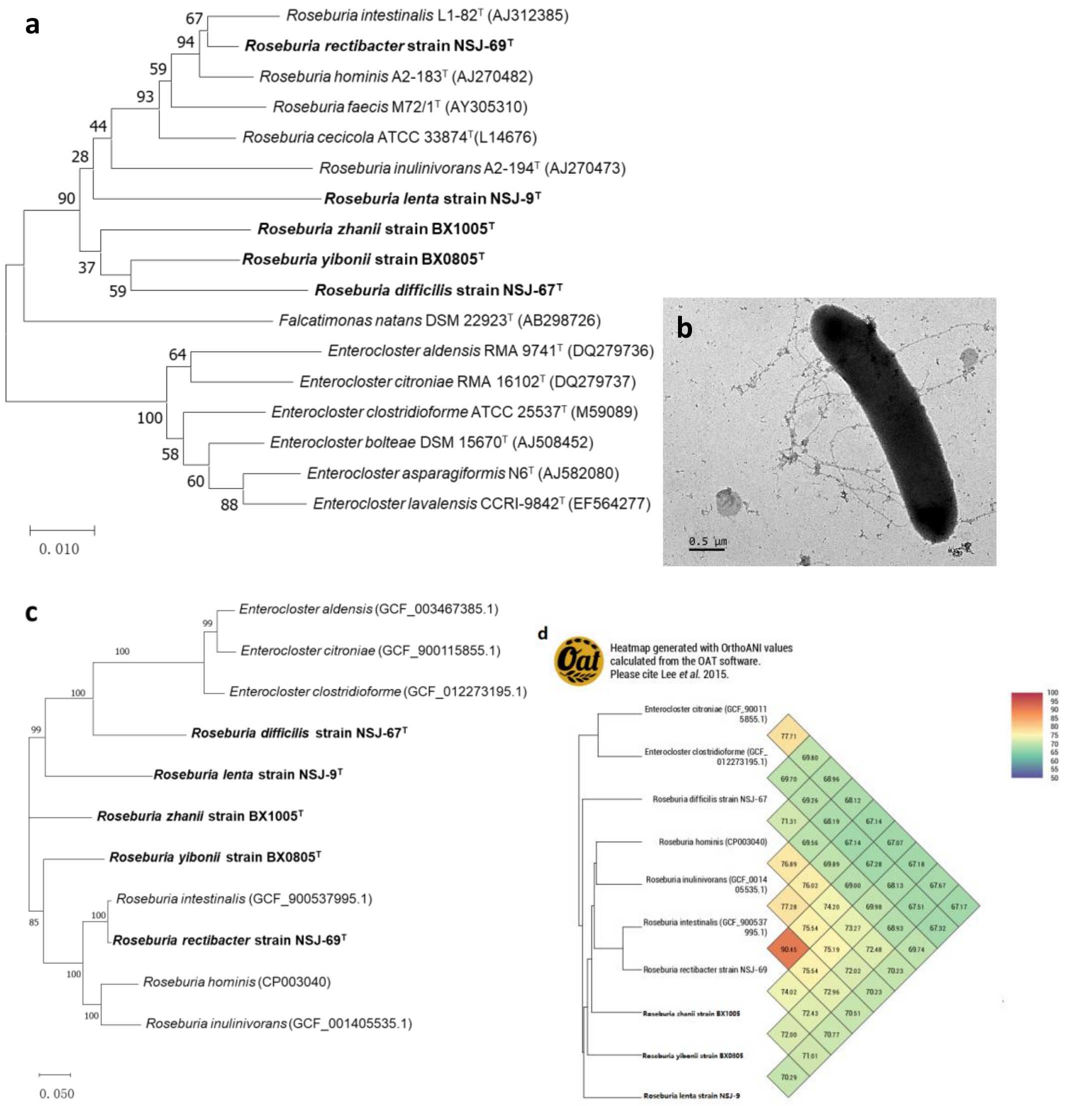

Figure SD-78. The Neighbour-joining phylogenetic tree based on 16S rRNA gene sequences (a) and the cell morphology (b) of strain BX1005^T. GenBank accession numbers are given in parentheses. Percentages of bootstrap support are shown at branch nodes. Bootstrap value was 1000. Bar: 0.01 substitutions per nucleotide position. The phylogenominc tree based on gemome-sequence-alignment (c) and OrthoANI heatmap (d) of strain BX1005^T and its neighbor species. Refseq ID of representative genomes are given in parentheses. Percentages of bootstrap support are shown at branch nodes. Bar: 0.050 substitutions per nucleotide position.

Taxon 79: *Roseburia rectibacter* sp. nov.

Description of *Roseburia rectibacter* sp. nov. (rec.ti.bac'ter. L. masc. adj. rectus, straight; N.L. masc. n. bacter, rod; N.L. masc. n. *rectibacter*, straight rod shaped, referring to the cell shape of the strain)

The taxon 79, represented by strain NSJ-69^T (16S rRNA sequence accession number in NMDC is NMDCN000012E), is closest to *Roseburia hominis*, with 16S rRNA gene identity of 97.9 %. Phylogenetic tree shows that strain NSJ-69^T clusters with other members of the genus *Roseburia* (Figure SD-79a), suggesting that strain NSJ-69^T is a member of the genus *Roseburia.* The genome of strain NSJ-69^T was sequenced and the NMDC accession number is NMDC60014076. Genome-based analysis on *Roseburia hominis* strain A2-183^T (NC 015977) and type strain NSJ-69^T reveals that the ANI value is 73.81 % and the dDDH estimation is 23.00 %. According to the phylogenominc tree (Figure SD-79c), the ANIs and dDDH values of genomes between strain NSJ-69^T (NMDC accession number is NMDC60014076) and phylogenomically neighbored genomes were calculated, and the highest ANI and dDDH values are 90.45 and 41.3 %, respectively, between the query genome of *Roseburia intestinalis* (GCF_900537995.1); and difference in % G+C between genomes of strain NSJ-69^T and *Roseburia intestinalis* (GCF_900537995.1) is 1.68; OrthoANI heatmap (Figure SD-79d) shows the phylogenomic status of the corresponding neighbor species based on OrthoANI values. Based on these results, we concluded that the strain NSJ-69^T represents a new species of the genus *Roseburia*, and the name *Roseburia rectibacter* sp.nov. is proposed.

Cells are strictly anaerobic, straight rod shaped with square ends and peritrichous flagella (2.5-5.9 µm long by 0.9-1.5 µm wide, Figure SD-79b); cells appear singly, in dividing pairs or in dividing chains. The organism uses m-tartaric acid and urocanic acid as the carbon source for growth, and weakly metabolizes N-acetyl-D glucosamine, dextrin, glucose-6-phosphate, dulcitol, m-inositol, glycyl-L-methionine and glycyl-L-proline. The DNA G+C content of the type strain NSJ-69^T is 41.00 mol %. The type strain is NSJ-69^T (=CGMCC 1.32845), which was isolated from the faeces of a healthy adult.

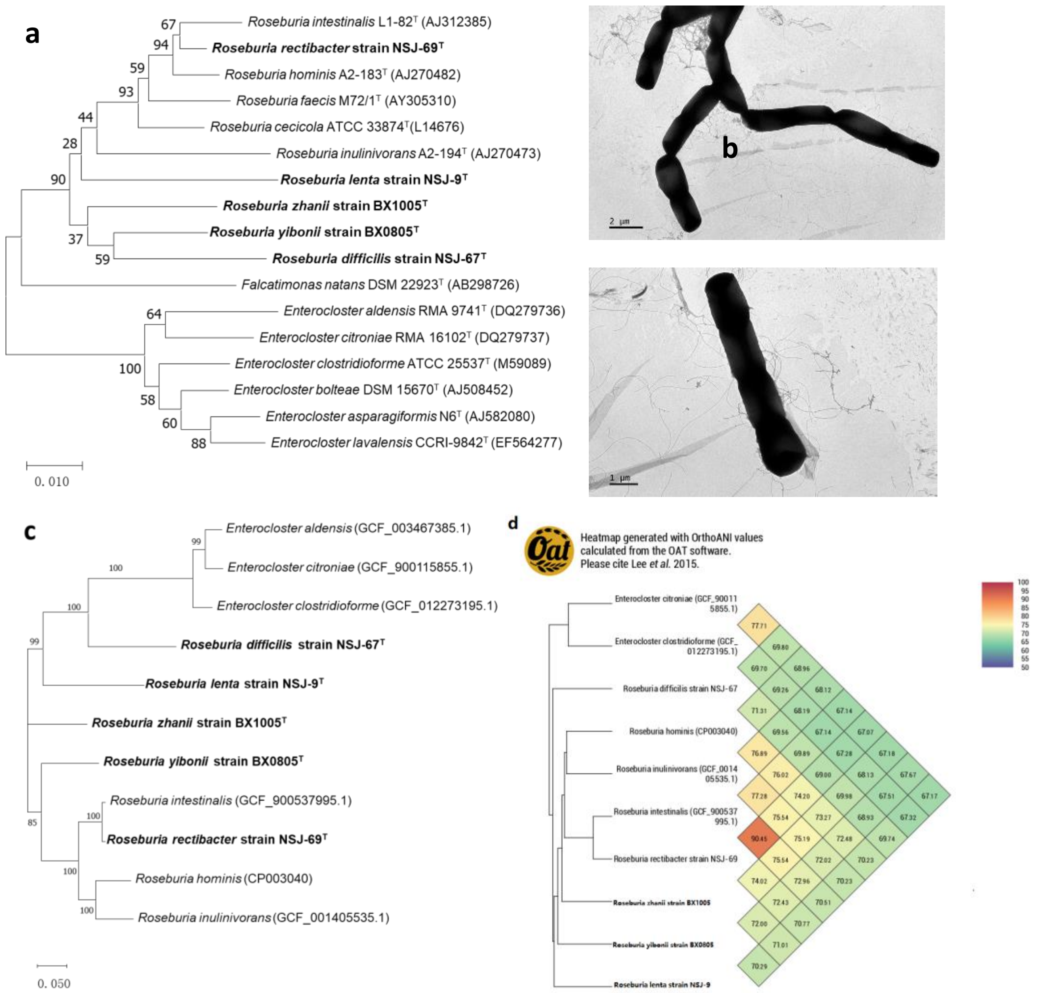

Figure SD-79. The Neighbour-joining phylogenetic tree based on 16S rRNA gene sequences (a) and the cell morphology (b) of strain NSJ-69^T. GenBank accession numbers are given in parentheses. Percentages of bootstrap support are shown at branch nodes. Bootstrap value was 1000. Bar: 0.01 substitutions per nucleotide position. The phylogenominc tree based on gemome-sequence-alignment (c) and OrthoANI heatmap (d) of strain NSJ-69^T and its neighbor species. Refseq ID of representative genomes are given in parentheses. Percentages of bootstrap support are shown at branch nodes. Bar: 0.050 substitutions per nucleotide position.

Taxon 80: *Roseburia difficilis* sp. nov.

Description of *Roseburia difficilis* sp. nov. (dif.fi'ci.lis. L. fem. adj. *difficilis*, difficult, referring the difficulty of culturing the strain)

The taxon 80, represented by strain NSJ-67^T (16S rRNA sequence accession number in NMDC is NMDCN000012F), is closest to *Roseburia faecis*, with 16S rRNA gene identity of 95.3 %. Phylogenetic tree shows that strain NSJ-67^T clusters with other members of the genus *Roseburia* (Figure SD-80a), suggesting that strain NSJ-67^T is a member of the genus *Roseburia.* The genome of strain NSJ-67^T was sequenced and the NMDC accession number is NMDC60014077. Genome-based analysis on *Roseburia faecis* strain M72/1^T (NZ CVRR00000000) and type strain NSJ-67^T reveals that the ANI value is 68.81 % and the dDDH estimation is 31.30. According to the phylogenominc tree (Figure SD-80c), the ANIs and dDDH values of genomes between strain NSJ-67^T (NMDC accession number is NMDC60014077) and phylogenomically neighbored genomes were calculated, and the highest ANI and dDDH values are 69.31 and 19.3 %, respectively, between the query genome of *Enterocloster aldensis* (GCF_003467385.1); and difference in % G+C between genomes of strain NSJ-67^T and *Enterocloster aldensis* (GCF_003467385.1) is 1.26; OrthoANI heatmap (Figure SD-80d) shows the phylogenomic status of the corresponding neighbor species based on OrthoANI values. Based on these results, we concluded that the strain NSJ-67^T represents a new species of the genus *Roseburia*, and the name *Roseburia difficilis* sp.nov. is proposed.

Cells are strictly anaerobic, spherical in shape with peritrichous flagella (diameter 0.8-1.9 μm, Figure SD-80b); Growth occurs at 37°C and at pH 7.0-7.5. The DNA G+C content of the type strain NSJ-67^T is 50.40 mol %. The type strain NSJ-67^T (= CGMCC 1.32843) was isolated from the faeces of a healthy adult.

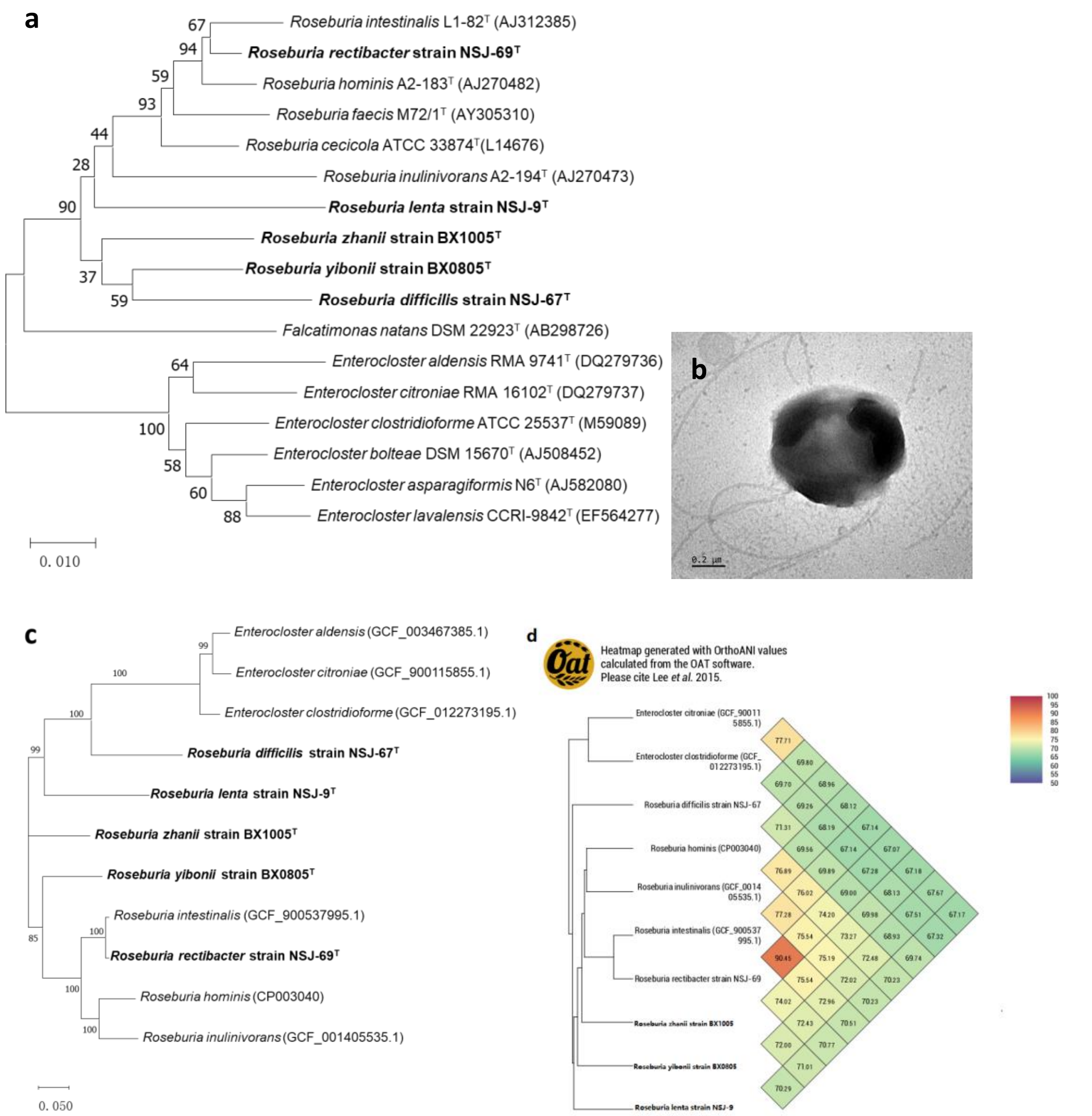

Figure SD-80. The Neighbour-joining phylogenetic tree based on 16S rRNA gene sequences (a) and the cell morphology (b) of strain NSJ-67^T. GenBank accession numbers are given in parentheses. Percentages of bootstrap support are shown at branch nodes. Bootstrap value was 1000. Bar: 0.01 substitutions per nucleotide position. The phylogenominc tree based on gemome-sequence-alignment (c) and OrthoANI heatmap (d) of strain NSJ-67^T and its neighbor species. Refseq ID of representative genomes are given in parentheses. Percentages of bootstrap support are shown at branch nodes. Bar: 0.05 substitutions per nucleotide position.

Taxon 81: *Agathobaculum hominis* sp. nov.

Description *of Agathobaculum hominis* sp. nov. (ho'mi.nis L. gen. masc. n. *hominis*, of a human being, referring to the human gut habitat)

The taxon 81, respresented by strain M2^T (16S rRNA sequence accession number in NMDC is NMDCN000012G), is phylogenetically closest to *Agathobaculum butyriciproducens*, with 16S rRNA gene identity of 98.52 %. Phylogenetic tree shows that strain M2^T clusters with other members of the genus *Agathobaculum,* (Figure SD-81a), suggesting strain M2^T is a member of the genus *Agathobaculum*. The genome of strain M2^T was sequenced and the NMDC accession number is NMDC60014078. Genome-based analysis showed that the ANI value of genomes of strain M2^T and *Agathobaculum butyriciproducens* SR79^T (NZ QEKJ00000000) is 81.59 % and the dDDH estimation is 27.10 %. According to the phylogenominc tree (Figure SD-81c), the ANIs and dDDH values of genomes between strain M2^T (NMDC accession number is NMDC60014078) and phylogenomically neighbored genomes were calculated, and the highest ANI and dDDH values are 81.28 and 27.1 %, respectively, between the query genome of *Agathobaculum butyriciproducens* (NZ_JNJN00000000.1); and difference in % G+C between genomes of strain M2^T and *Agathobaculum butyriciproducens* (NZ_JNJN00000000.1) is 2.67; OrthoANI heatmap (Figure SD-81d) shows the phylogenomic status of the corresponding neighbor species based on OrthoANI values. Based on these results, we concluded that the strain M2^T represents of new species of the genus *Agathobaculum*, and the name *Agathobaculum hominis* sp. nov. is proposed.

Cells are strictly anaerobic, oval to rod shaped with spiky ends in various sizes (1.8-3.5 µm long by 1.3-1.5 µm wide, Figure SD-81b); cells appear singly, in dividing pairs or in chains. Pinpoint, pale yellow, tiny, raised colonies appear on modified mGAM agar plate after 3 days of incubation. Growth occurs at 37°C and at pH 7.0-7.5. The organism uses D-fructose, L-fucose, D-galactose, D-galacturonic acid, m-inositol, D-mannose, 3-melthyl-D-glucose, palatinose, L-rhamnose, pyruvic acid and inosine as the carbon source for growth, and weakly metabolizes gentiobiose, α-D-glucose and gentiobiose. The DNA G+C content of the type strain M2^T (=CGMCC 1.32866^T)is 65.58 mol %. The type strain M2^T was isolated from the faeces of a healthy adult.

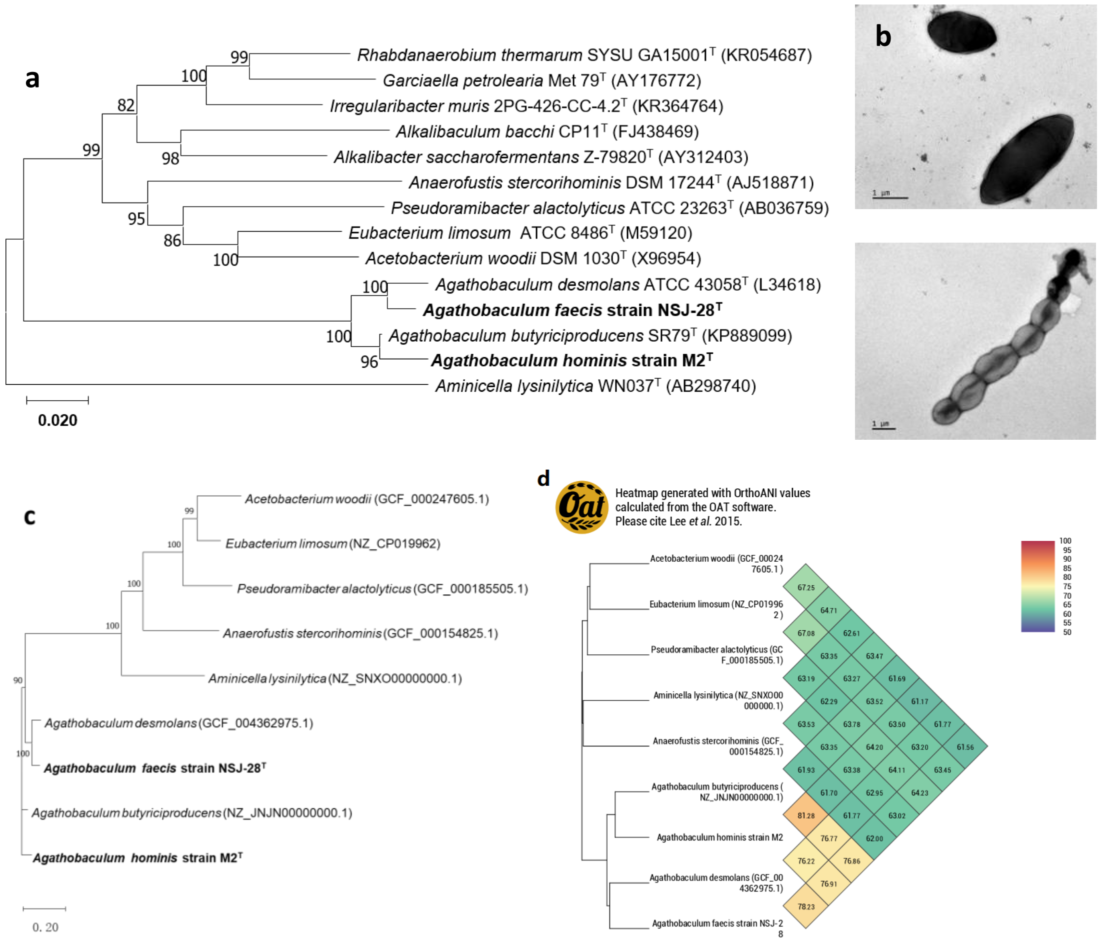

Figure SD-81. The Neighbour-joining phylogenetic tree based on 16S rRNA gene sequences (a) and the cell morphology (b) of strain M2^T. GenBank accession numbers are given in parentheses. Percentages of bootstrap support are shown at branch nodes. Bootstrap value was 1000. Bar: 0.02 substitutions per nucleotide position. The phylogenominc tree based on gemome-sequence-alignment (c) and OrthoANI heatmap (d) of strain M2^T and its neighbor species. Refseq ID of representative genomes are given in parentheses. Percentages of bootstrap support are shown at branch nodes. Bar: 0.20 substitutions per nucleotide position.

Taxon 82: ***Agathobaculum faecis*** **sp. nov.**

Description ***of Agathobaculum faecis*** **sp. nov.** (fae'cis. L. gen. fem. n. *faecis*, of faeces, from which the organism was isolated)

The taxon 82, respresented by strain NSJ-28^T (16S rRNA sequence accession number in NMDC is NMDCN000012H), is phylogenetically closest to *Agathobaculum desmolans*, with 16S rRNA gene identity of 98.1 %. Phylogenetic tree shows that strain NSJ-28^T clusters with other members of the genus *Agathobaculum,* (Figure SD-82a), suggesting strain NSJ-28^T is a member of the genus *Agathobaculum*. The genome of strain NSJ-28^T was sequenced and the NMDC accession number is NMDC60014079. Genome-based analysis showed that the ANI value of genomes of strain NSJ-28^T and *Agathobaculum desmolans* ATCC 43058^T (NZ JNJN00000000) is 78.23 % and the dDDH estimation is 22.30 %. According to the phylogenominc tree (Figure SD-82c), the ANIs and dDDH values of genomes between strain NSJ-28^T (NMDC accession number is NMDC60014079) and phylogenomically neighbored genomes were calculated, and the highest ANI and dDDH values are 76.22 and 22.3 %, respectively, between the query genome of *Agathobaculum desmolans* (GCF_004362975.1); and difference in % G+C between genomes of strain NSJ-28^T and *Agathobaculum desmolans* (GCF_004362975.1) is 2.62; OrthoANI heatmap (Figure SD-82d) shows the phylogenomic status of the corresponding neighbor species based on OrthoANI values. Based on these results, we concluded that the strain NSJ-28^T represents of new species of the genus *Agathobaculum*, and the name *Agathobaculum faecis* sp. nov. is proposed.

Cells are strictly anaerobic, straight rods (1.8-2.3 µm long by 0.7-0.9 µm wide, Figure SD-82b) with peritrichous flagella (Figure SD-82b). Cells appear singly or in dividing pairs. Irregular, pinpoint, cream-colored, slightly raised colonies appear on modified mGAM agar plate after 3 days of incubation. Growth occurs at 37°C and at pH of 7.0-7.5. The organism uses β-cyclodextrin, m-tartaric acid, urocanic acid, glycyl-L-glutamine and thymidine-5'-monophosphate as the carbon source for growth, and weakly metabolizesα-cyclodextrin, α-D-glucose, sucrose and D-trehalose. The DNA G+C content of the type strain NSJ-28^T is 65.58 mol %. The type strain NSJ-28^T (=CGMCC 1.32801^T) was isolated from the faeces of a healthy adult.

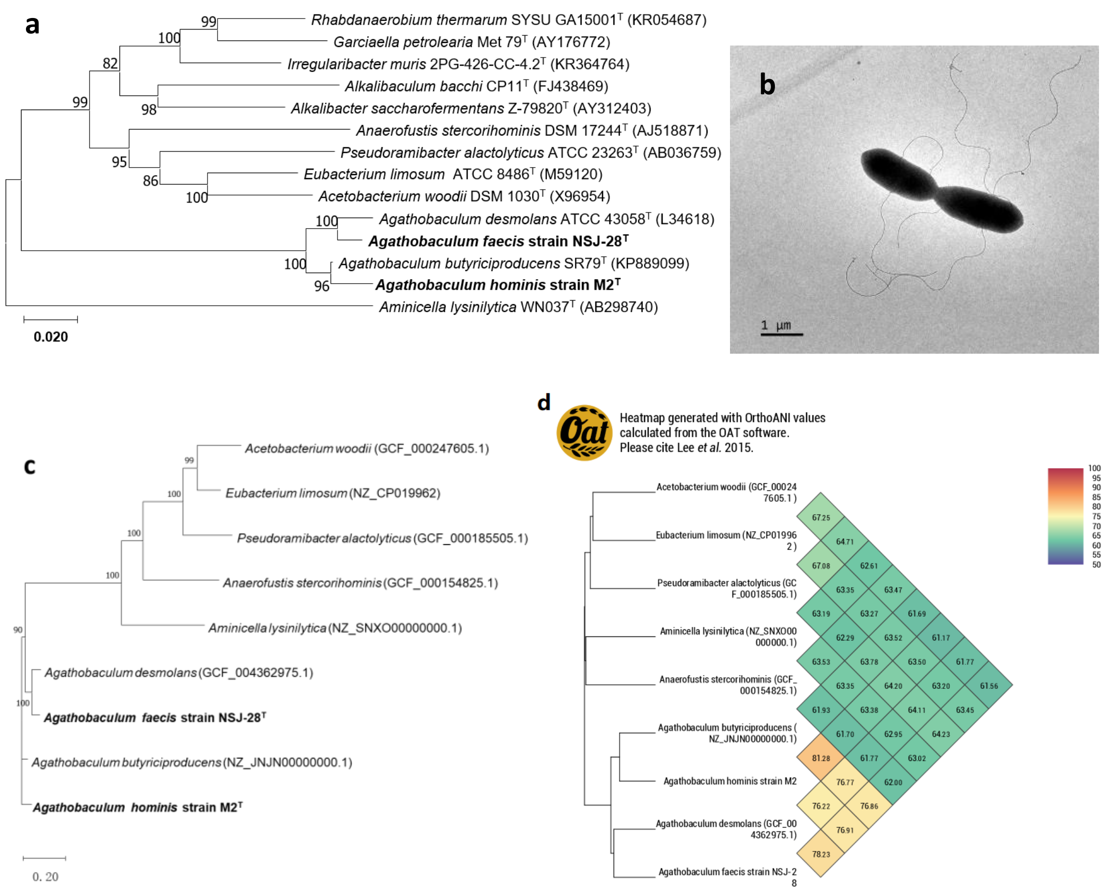

Figure SD-82. The Neighbour-joining phylogenetic tree based on 16S rRNA gene sequences (a) and the cell morphology (b) of strain NSJ-28^T. GenBank accession numbers are given in parentheses. Percentages of bootstrap support are shown at branch nodes. Bootstrap value was 1000. Bar: 0.02 substitutions per nucleotide position. The phylogenominc tree based on gemome-sequence-alignment (c) and OrthoANI heatmap (d) of strain NSJ-28^T and its neighbor species. Refseq ID of representative genomes are given in parentheses. Percentages of bootstrap support are shown at branch nodes. Bar: 0.20 substitutions per nucleotide position.

Taxon 83: *Anaerotruncus massiliensis* sp.

Description *of Anaerotruncus massiliensis* sp. (mas.si.li.en'sis. L. masc./fem. adj. *massiliensis*, pertaining to Marseille, France, where the organism was for the first time isolated)

The taxon 83, respresented by strain 22A2-44^T (16S rRNA sequence accession number in NMDC is NMDCN000012I), is phylogenetically closest to *Anaerotruncus colihominis*, with 16S rRNA gene identity of 96.33 %. Phylogenetic tree shows that strain 22A2-44^T clusters with other members of the genus *Anaerotruncus,* (Figure SD-83a), suggesting strain 22A2-44^T is a member of the genus *Anaerotruncus.* The genome of strain 22A2-44^T was sequenced and the NMDC accession number is NMDC60014080. Genome-based analysis showed that the ANI value of genomes of strain 22A2-44^T and *Anaerotruncus colihominis* strain WAL 14565^T (NZ ABGD00000000) is 73.84 % and the dDDH estimation is 14.10 %. According to the phylogenominc tree (Figure SD-83c), the ANIs and dDDH values of genomes between strain 22A2-44^T (NMDC accession number is NMDC60014080) and phylogenomically neighbored genomes were calculated, and the highest ANI and dDDH values are 73.8422 and 20.9 %, respectively, between the query genome of *Anaerotruncus colihominis* (GCF_015554285.1); and difference in % G+C between genomes of strain 22A2-44^T and *Anaerotruncus colihominis* (GCF_015554285.1) is 9.17; OrthoANI heatmap (Figure SD-83d) shows the phylogenomic status of the corresponding neighbor species based on OrthoANI values. Based on these results, we concluded that the strain 22A2-44^T represents of new species of the genus *Anaerotruncus*, the name *Anaerotruncus massiliensis* sp. nov. is proposed by Togo. Here we present the characteristics of the type strain to validate the species name *Anaerotruncus massiliensis*.

Cells are strictly anaerobic, gram positive, long straight rods (2.0-4.5 μm long by 0.5-0.7 μm wide, Figure SD-83b). Cells appear singly or in dividing pairs. Irregular, semi-transparent, slightly raised colonies appear on YCFA agar plate after 3 days of incubation. Growth occurs at 37°C and at pH of 7.0-7.5. The organism uses N-acetyl-D-glucosamine, N-acetyl-β-D-mannosamine, D-arabitol, D-cellobiose, dextrin, i-erythritol, D-fructose, L-fucose, D-galactose, D-galacturonic acid, gentiobiose, D-gluconic acid, D-glucosaminic acid, α-D-glucose, glucose-1-phosphate, glucose-6-phosphate, glycerol, α-D-lactose, lactulose, maltose, maltotriose, D-mannose, D-melezitose, glycerol1, 3-melthyl-D-glucose, ß-methyl-D-galactoside, α-methyl-D-glucoside, palatinose, L-rhamnose, D-sorbitol, sucrose, D-trehalose, turanose, glyoxylic acid, α-ketobutyric acid, α-ketovaleric acid, D-lactic acidmethyl ester, L-malic acid, pyruvic acidDextrin, pyruvic acidmethyl ester, L-glutamic acid, L-methionine, L-phenylalanine, L-valine plus L-aspartic acid, 2'-deoxyadenosine, inosine, thymidine-5'-monophosphate, uridine-5'-monophosphate, thymidine, uridine as the carbon source for growth, and weakly utilizes N-acetyl-D-galactosamine, amygdalin, ß-methyl-D-glucoside, D-saccharic acid, m-tartaric acid, L-alanine, L-alanyl-L-glutamine and glycyl-L-methionine. The DNA G+C content of the type strain 22A2-44^T is 64.2 mol %. The type strain 22A2-44^T

(=CGMCC 1.5238^T=NBRC 113434^T) was isolated from the faeces of a healthy adult.

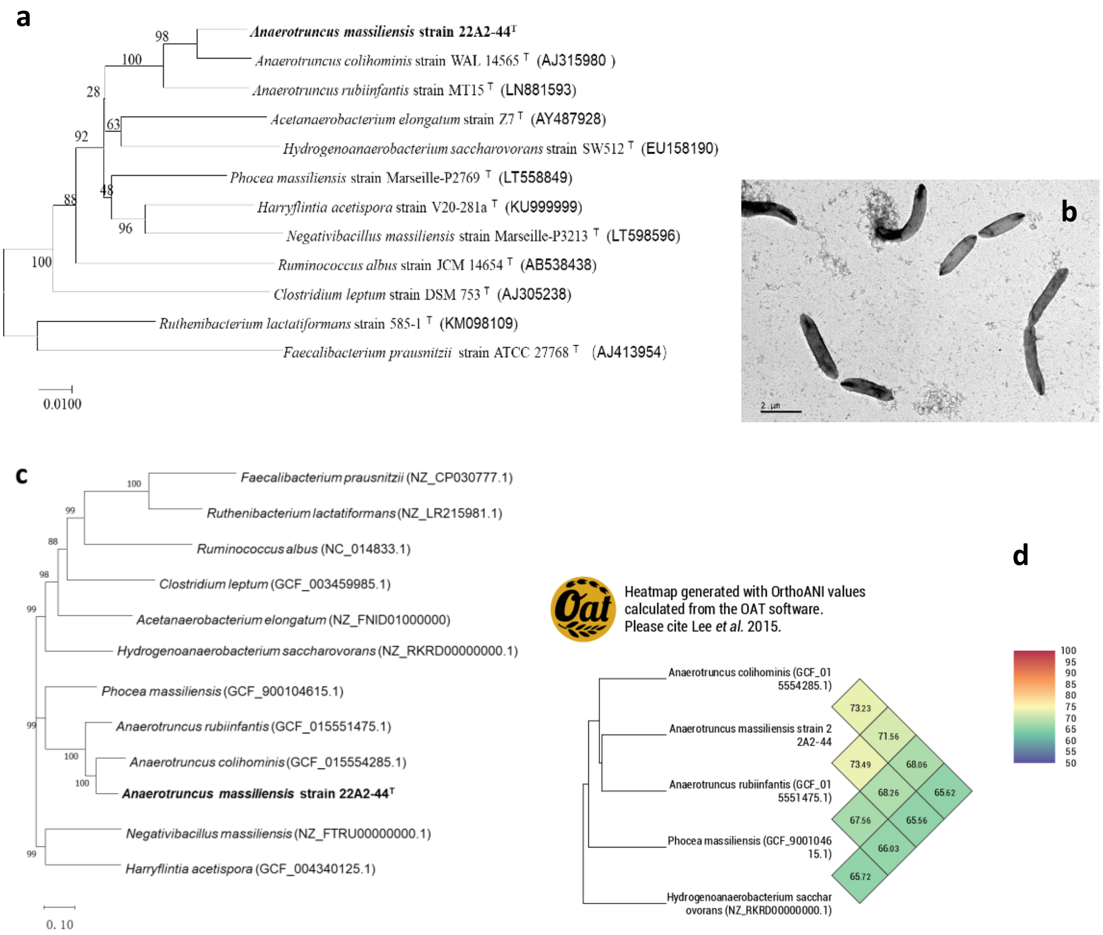

Figure SD-83. The Neighbour-joining phylogenetic tree based on 16S rRNA gene sequences (a) and the cell morphology (b) of strain 22A2-44^T. GenBank accession numbers are given in parentheses. Percentages of bootstrap support are shown at branch nodes. Bootstrap value was 1000. Bar: 0.01 substitutions per nucleotide position. The phylogenominc tree based on gemome-sequence-alignment (c) and OrthoANI heatmap (d) of strain 22A2-44^T and its neighbor species. Refseq ID of representative genomes are given in parentheses. Percentages of bootstrap support are shown at branch nodes. Bar: 0.1 substitutions per nucleotide position.

Taxon 84: *Dysosmobacter segnis* sp. nov.

Description *of Dysosmobacter segnis* sp. nov. (seg'nis. L. masc. adj. *segnis*, slow, inactive, lazy, referring the slow growth of the strain)
The taxon 84, respresented by strain BX15^T (16S rRNA sequence accession number in NMDC is NMDCN000012J), is phylogenetically closest to *Dysosmobacter welbionis*[9], with 16S rRNA gene identity of 95.7 %. Phylogenetic tree shows that strain BX15^T clusters with other members of the genus *Dysosmobacter* (Figure SD-84a), suggesting strain BX15^T is a member of the genus *Dysosmobacter.* The genome of strain BX15^T was sequenced and the NMDC accession number is NMDC60014081. Genome-based analysis showed that the ANI value of genomes of strain BX15^T and *D. welbionis* J115^T (NZ BAGW00000000) is 75.85 % and the dDDH estimation is 23.70 %. According to the phylogenominc tree (Figure SD-84c), the ANIs and dDDH values of genomes between strain BX15^T (NMDC accession number is NMDC60014081) and phylogenomically neighbored genomes were calculated, and the highest ANI and dDDH values are 76.7 and 23.7 %, respectively, between the query genome of *Dysosmobacter welbionis* (GCF_005121165.1); and difference in % G+C between genomes of strain BX15^T and *Dysosmobacter welbionis* (GCF_005121165.1) is 3.4; OrthoANI heatmap (Figure SD-84d) shows the phylogenomic status of the corresponding neighbor species based on OrthoANI values. Based on these results, we concluded that the strain BX15^T represents of new species of the genus *Dysosmobacter*, and the name *Dysosmobacter hominis* sp. nov. is proposed
Cells are strictly anaerobic rods (1.6-4.0 μm long by 0.6-0.8 μm wide, Figure SD-84b); cells appear singly. Growth occurs at 37°C and at pH 7.0-7.5. The DNA G+C content of the type strain BX15^T is 55.5 mol %. The type strain 22A2-44^T (=CGMCC 1.32894^T) was isolated from the faeces of a healthy adult.

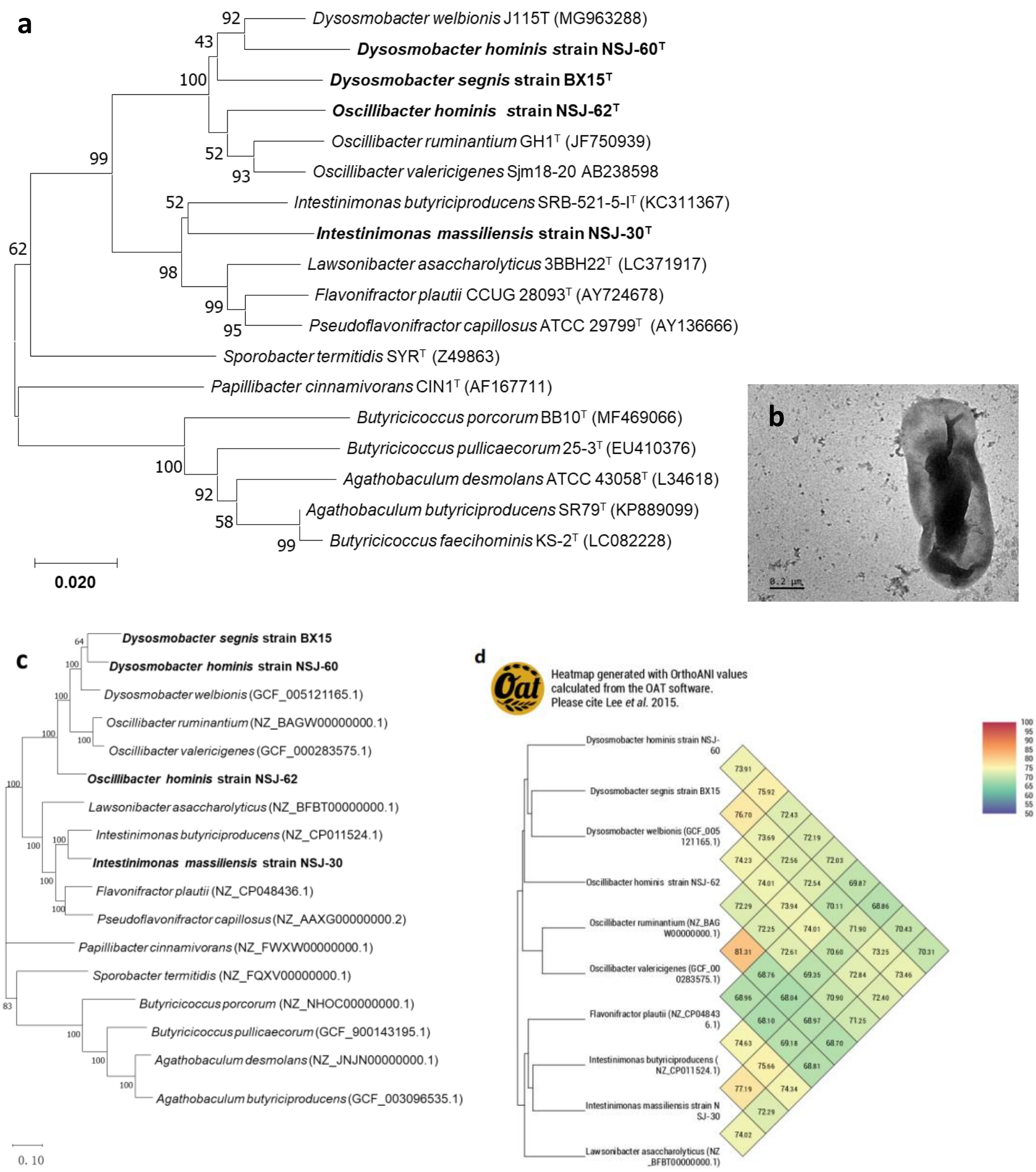

Figure SD-84. The Neighbour-joining phylogenetic tree based on 16S rRNA gene sequences (a) and the cell morphology (b) of strain BX15^{T}. GenBank accession numbers are given in parentheses. Percentages of bootstrap support are shown at branch nodes. Bootstrap value was 1000. Bar: 0.02 substitutions per nucleotide position. The phylogenominc tree based on gemome-sequence-alignment (c) and OrthoANI heatmap (d) of strain BX15^{T} and its neighbor species. Refseq ID of representative genomes are given in parentheses. Percentages of bootstrap support are shown at branch nodes. Bar: 0.10 substitutions per nucleotide position.

Taxon 85: *Dysosmobacter hominis* sp. nov.

Description *of Dysosmobacter hominis* sp. nov. (ho'mi.nis L. gen. masc. n. *hominis*, of a human being, referring to the human gut habitat)

The taxon 85, respresented by strain NSJ-60^T (16S rRNA sequence accession number in NMDC is NMDCN000012K), is phylogenetically closest to *Dysosmobacter welbionis*[9], with 16S rRNA gene identity of 96.3 %. Phylogenetic tree shows that strain NSJ-60^T clusters with other members of the genus *Dysosmobacter* (Figure SD-85a), suggesting strain NSJ-60^T is a member of the genus *Dysosmobacter.* The genome of strain NSJ-60^T was sequenced and the NMDC accession number is NMDC60014082. Genome-based analysis showed that the ANI value of genomes of strain NSJ-60^T and *D. welbionis* J115^T (NZ BAGW00000000) is 76.38 % and the dDDH estimation is 19.7 %. According to the phylogenominc tree (Figure SD-85c), the ANIs and dDDH values of genomes between strain NSJ-60^T (NMDC accession number is NMDC60014082) and phylogenomically neighbored genomes were calculated, and the highest ANI and dDDH values are 75.92 and 22 %, respectively, between the query genome of *Dysosmobacter segnis* strain BX15^T (NMDC60014081); and difference in % G+C between genomes of strain NSJ-60^T and *Dysosmobacter segnis* strain BX15^T (NMDC60014081) is 0.17; OrthoANI heatmap (Figure SD-85d) shows the phylogenomic status of the corresponding neighbor species based on OrthoANI values. Based on these results, we concluded that the strain NSJ-60^T represents of new species of the genus *Dysosmobacter*, and the name *Dysosmobacter hominis* sp. nov. is proposed

Cells are strictly anaerobic, straight rods (1.2-3.8 µm long by 0.6-0.9 µm wide, Figure SD-85b) with flagella monotrichous or amphitrichous on the spiky ends, and cells appear singly or in dividing pairs. Tiny, pinpoint, yellow, thin colonies appear on modified mGAM agar plate after 6 days of incubation. Growth occurs at 37°C and at pH 7.0-7.5. The organism uses dextrin, D-fructose, L-fucose, D-galactose, D-galacturonic acid, α-D-glucose, glucose-6-phosphate, 3-melthyl-D-glucose, gentiobiose, palatinose, L-rhamnose, fumaric acid, L-glutamic acid, D-mannose, glyoxylic acid, L-malic acid, pyruvic acid, D-melibiose, pyruvic acidmethyl ester, L-serine, 2'-deoxyadenosine, inosine and uridine as the carbon source for growth, and weakly metabolizes D-cellobiose, i-erythritol, D-gluconic acid, D-glucosaminic acid, glucose-1-phosphate, lactulose, turanose, α-ketobutyric acid, α-ketovaleric acid and L-alanyl-L-glutamine. The DNA G+C content of the type strain NSJ-60^T is 58.6 mol %. The type strain NSJ-60^T (=CGMCC 1.32836^T) was isolated from the faeces of a healthy adult.

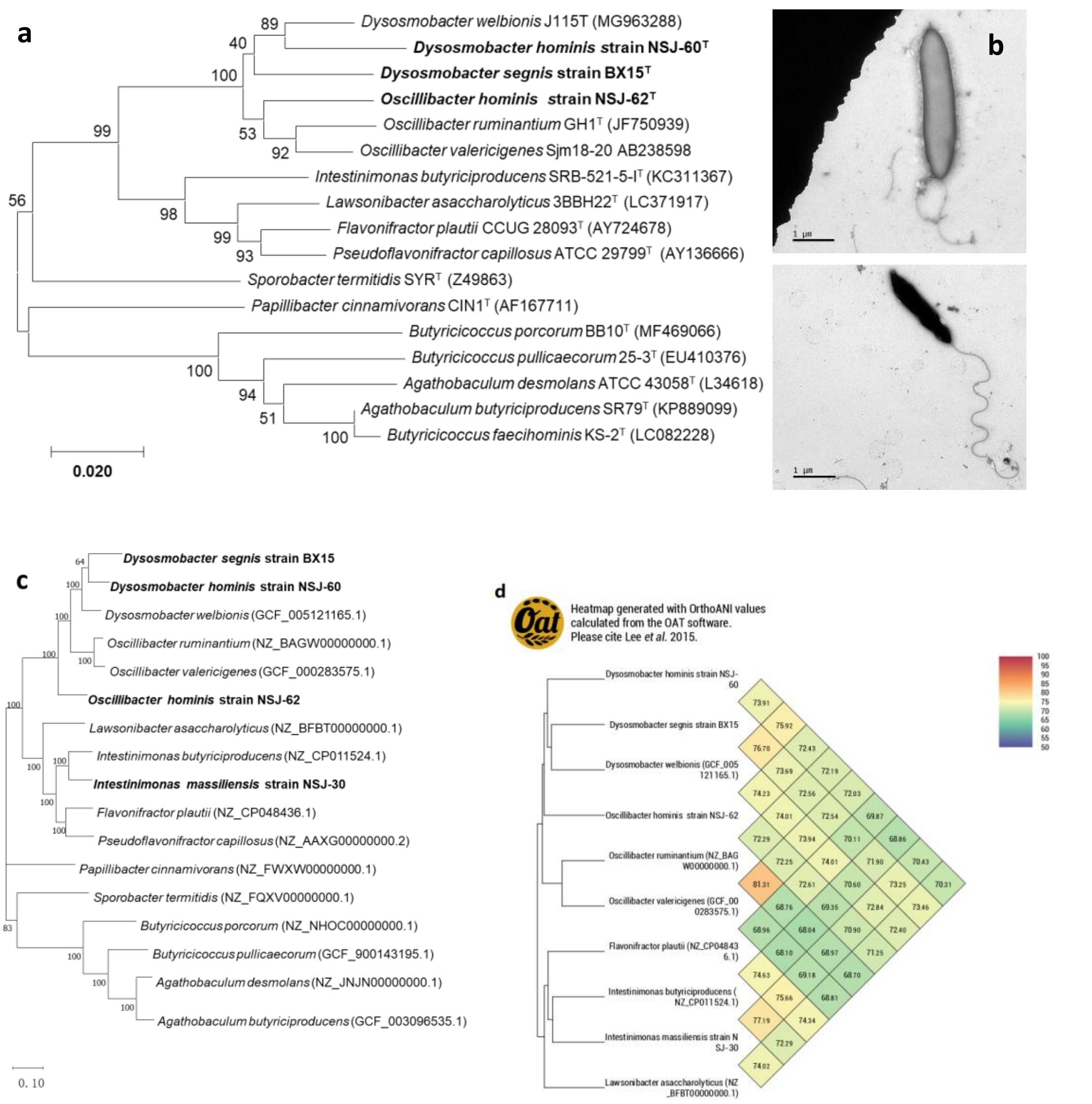

Figure SD-85. The Neighbour-joining phylogenetic tree based on 16S rRNA gene sequences (a) and the cell morphology (b) of strain NSJ-60^T. GenBank accession numbers are given in parentheses. Percentages of bootstrap support are shown at branch nodes. Bootstrap value was 1000. Bar: 0.02 substitutions per nucleotide position. The phylogenominc tree based on gemome-sequence-alignment (c) and OrthoANI heatmap (d) of strain NSJ-60^T and its neighbor species. Refseq ID of representative genomes are given in parentheses. Percentages of bootstrap support are shown at branch nodes. Bar: 0.10 substitutions per nucleotide position.

Taxon 86: *Faecalibacterium hominis* sp. nov.

Description *of Faecalibacterium hominis* sp. nov. (ho'mi.nis L. gen. masc. n. hominis, of a human being, referring to the human gut habitat)

The taxon 86, respresented by strain 4P-15^T (16S rRNA sequence accession number in NMDC is NMDCN000012L), is phylogenetically closest to *Faecalibacterium prausnitzii*, with 16S rRNA gene identity of 98.06 %. Phylogenetic tree shows that strain 4P-15^T clusters with other members of the genus *Faecalibacterium* (Figure SD-86a), suggesting strain 4P-15^T is a member of the genus *Faecalibacterium.* The genome of strain 4P-15^T was sequenced and the NMDC accession number is NMDC60014083. Genome-based analysis showed that the ANI value of genomes of strain 4P-15^T and *Faecalibacterium prausnitzii* ATCC 27768^T (NZ ACOP00000000) is 80.04 % and the dDDH estimation is 29.10 %. According to the phylogenominc tree (Figure SD-86c), the ANIs and dDDH values of genomes between 4P-15^T (NMDC accession number is NMDC60014083) and phylogenomically neighbored genomes were calculated, and the highest ANI and dDDH values are 84.18 and 29.1 %, respectively, between the query genome of *Faecalibacterium prausnitzii* (NZ_PXUP00000000.1); and difference in % G+C between genomes of 4P-15^T and *Faecalibacterium prausnitzii* (NZ_PXUP00000000.1) is 0.39; OrthoANI heatmap (Figure SD-86d) shows the phylogenomic status of the corresponding neighbor species based on OrthoANI values. Based on these results, we concluded that the strain 4P-15^T represents of new species of the genus *Faecalibacterium*, and the name *Faecalibacterium hominis* sp. nov. is proposed.

Cells are strictly anaerobic, rod shaped (1.3-2.9 μm long by 0.6-0.8 μm wide, Figure SD-86b); cells appear singly or in dividing pairs. Growth occurs at 37°C and at pH 7.0-7.5. The strain uses pyruvic acidmethyl ester, m-tartaric acid and urocanic acid as the carbon source for growth, and weakly metabolizes D-malic acid and pyruvic acid.

The DNA G+C content of the type strain is 59.53 mol %. The type strain is 4P-15^T (=CGMCC 1.5250^T =NBRC 113913^T), which was isolated from the faeces of a healthy adult.

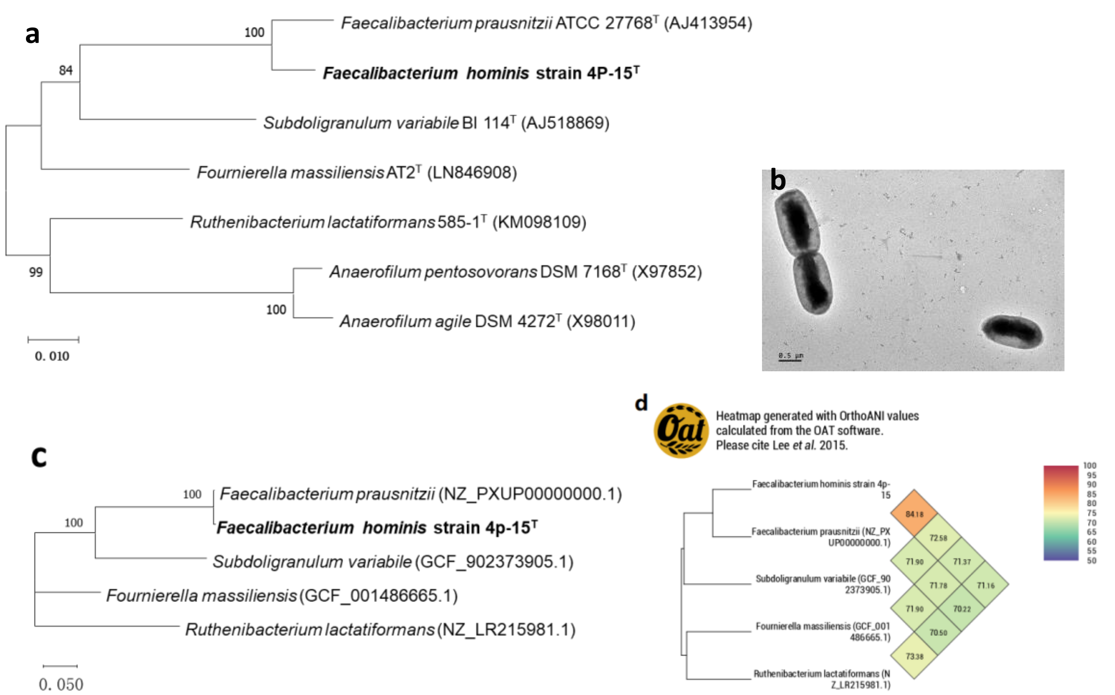

Figure SD-86. The Neighbour-joining phylogenetic tree based on 16S rRNA gene sequences (a) and the cell morphology (b) of strain 4P-15^T. GenBank accession numbers are given in parentheses. Percentages of bootstrap support are shown at branch nodes. Bootstrap value was 1000. Bar: 0.01 substitutions per nucleotide position. The phylogenominc tree based on gemome-sequence-alignment (c) and OrthoANI heatmap (d) of strain 4P-15^T and its neighbor species. Refseq ID of representative genomes are given in parentheses. Percentages of bootstrap support are shown at branch nodes. Bar: 0.05 substitutions per nucleotide position.

Taxon 87: *Flintibacter faecis* sp. nov.

Description *of Flintibacter faecis* sp. nov. (L. gen. fem. n. faecis, of faeces, from which the organism was isolated)

The taxon 87, respresented by strain BX5^T (16S rRNA sequence accession number in NMDC is NMDCN000012M), is phylogenetically closest to *Flintibacter butyricus*, with 16S rRNA gene identity of 96.3 %. Phylogenetic tree shows that strain BX5^T clusters with the member of the genus *Flintibacter* (Figure SD-87a), suggesting strain BX5^T is a member of the genus *Flintibacter.* The genome of strain BX5^T was sequenced and the NMDC accession number is NMDC60014084. Genome-based analysis showed that the ANI value of genomes of strain BX5^T and *F. butyricus* BLS21^T (NZCP044227) is 79.05 % and the dDDH estimation is 23.00 %. According to the phylogenominc tree (Figure SD-87c), the ANIs and dDDH values of genomes between strain BX5^T (NMDC accession number is NMDC60014084) and phylogenomically neighbored genomes were calculated, and the highest ANI and dDDH values are 63.6 and 23.6 %, respectively, between the query genome of *Flintibacter hominis* strain New-19^T (NMDC60014085); and difference in % G+C between genomes of strain BX5^T and *Flintibacter hominis* strain New-19^T (NMDC60014085) is 2.47; OrthoANI heatmap (Figure SD-87d) shows the phylogenomic status of the corresponding neighbor species based on OrthoANI values. Based on these results, we concluded that the strain BX5^T represents of new species of the genus *Flintibacter*, and the name *Flintibacter faecis sp. nov.* is proposed.

Cells are strictly anaerobic rods (1.4-2.6 µm long by 0.6-1.2 µm wide, Figure SD-87b). Growth occurs at 37°C and at pH 7.0-7.5. The DNA G+C content of the type strain BX5^T (CGMCC 1.32861^T) is 58.6 mol %.

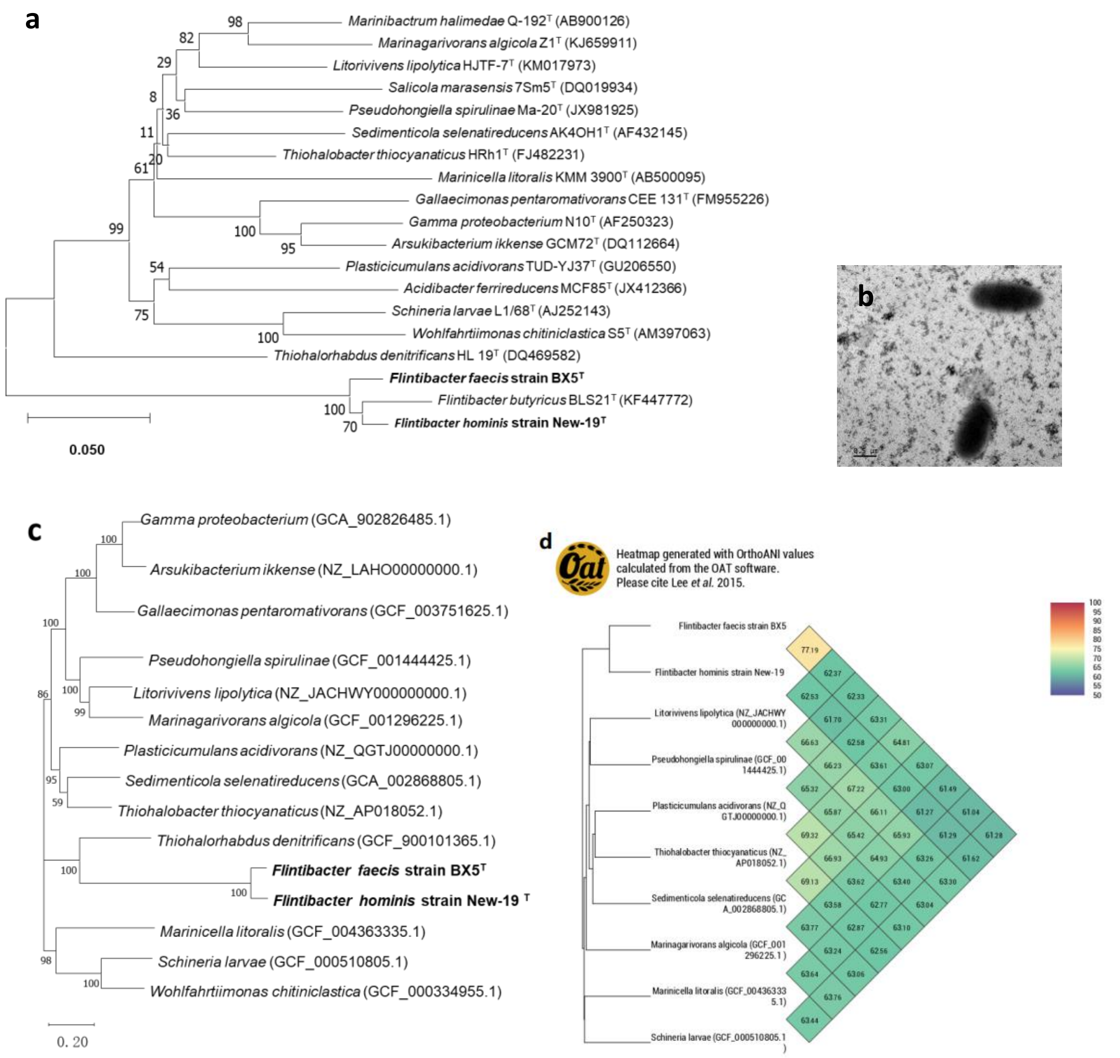

Figure SD-87. The Neighbour-joining phylogenetic tree based on 16S rRNA gene sequences (a) and the cell morphology (b) of strain BX5^{T}. GenBank accession numbers are given in parentheses. Percentages of bootstrap support are shown at branch nodes. Bootstrap value was 1000. Bar: 0.05 substitutions per nucleotide position. The phylogenominc tree based on gemome-sequence-alignment (c) and OrthoANI heatmap (d) of strain BX5^{T} and its neighbor species. Refseq ID of representative genomes are given in parentheses. Percentages of bootstrap support are shown at branch nodes. Bar: 0.20 substitutions per nucleotide position.

Taxon 88: *Flintibacter hominis* sp. nov.

Description *of Flintibacter hominis* sp. nov. (ho'mi.nis. L. gen. masc. n. *hominis*, of a human being, referring to the human gut habitat)

The taxon 88, respresented by strain New-19^T (16S rRNA sequence accession number in NMDC is NMDCN000012N), is phylogenetically closest to *Flintibacter butyricus*[7], with 16S rRNA gene identity of 96.2 %. Phylogenetic tree shows that strain New-19^T clusters with the member of the genus *Flintibacter* (Figure SD-88a), suggesting strain New-19^T is a member of the genus *Flintibacter.* The genome of strain New-19^T was sequenced and the NMDC accession number is NMDC60014085. Genome-based analysis showed that the ANI value of genomes of strain New-19^T and *F. butyricus* BLS21^T (NZCP044227) is 76.95 % and the dDDH estimation is 24.00 %. According to the phylogenominc tree (Figure SD-88c), the ANIs and dDDH values of genomes between strain New-19^T (NMDC accession number is NMDC60014085) and phylogenomically neighbored genomes were calculated, and the highest ANI and dDDH values are 62.308 and 23.6 %, respectively, between the query genome of *Flintibacter faecis* strain BX5^T (NMDC60014084); and difference in % G+C between genomes of strain New-19^T and *Flintibacter faecis* strain BX5^T (NMDC60014084) is 2.47; OrthoANI heatmap (Figure SD-88d) shows the phylogenomic status of the corresponding neighbor species based on OrthoANI values. Based on these results, we concluded that the strain New-19^T represents of new species of the genus *Flintibacter*, and the name *Flintibacter hominis sp. nov.* is proposed.

Cells are strictly anaerobic, straight rods with spiky ends (3.1-4.2 µm long by 0.6-0.8 µm wide, Figure SD-88b). Tiny, cream-colored, smooth, thin, pinpoint colonies appear on modified mGAM agar plate after 3 days of incubation. Growth occurs at 37°C and at pH 7.0-7.5. The organism uses D-fructose, L-fucose, D-galacturonic acid and palatinose as the carbon source for growth, and weakly metabolizes L-rhamnose and pyruvic acid methyl ester. The DNA G+C content of the type strain New-19^T is 60.49 mol %. The type strain New-19^T (=CGMCC 1.31644^T =KCTC 15861^T) was isolated from the faeces of a healthy adult.

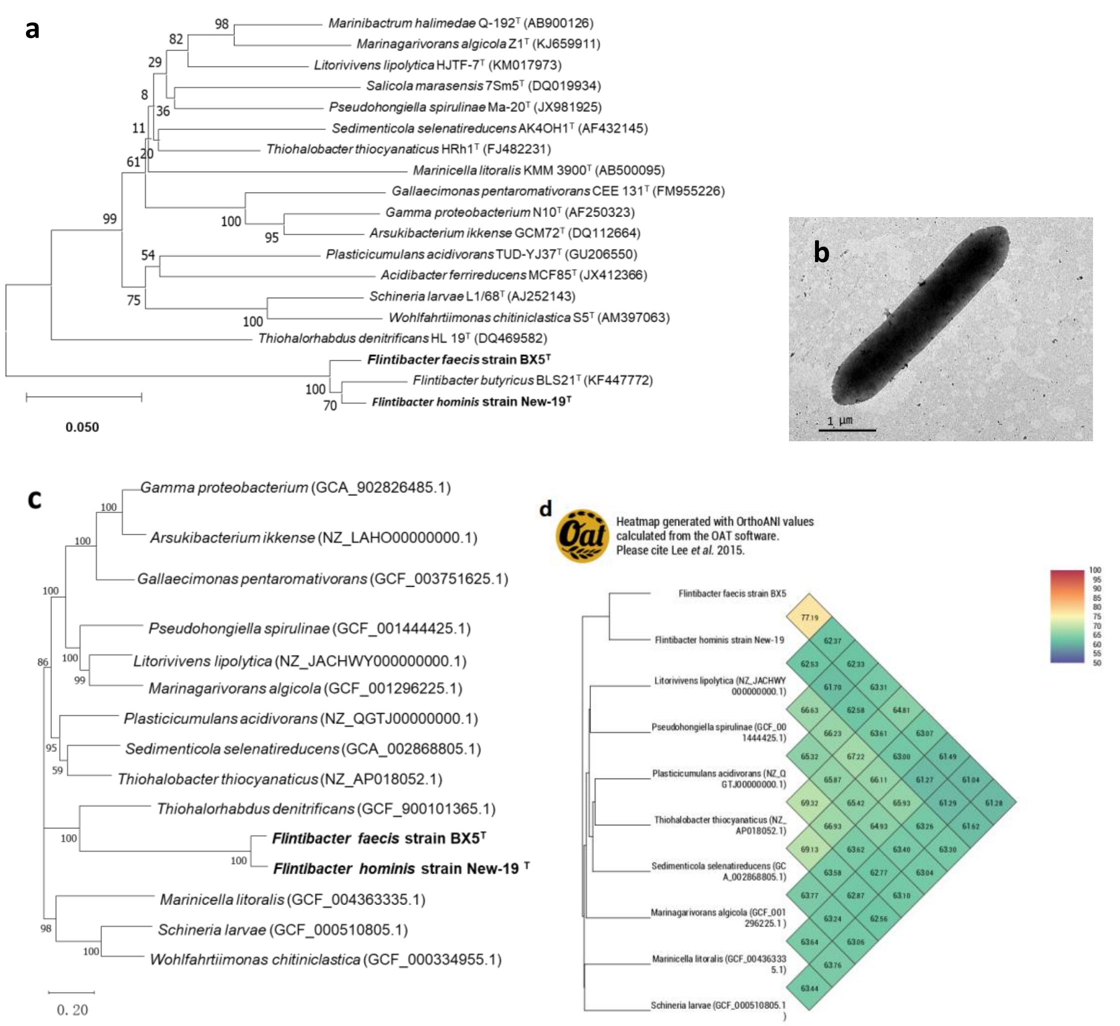

Figure SD-88. The Neighbour-joining phylogenetic tree based on 16S rRNA gene sequences (a) and the cell morphology (b) of strain New-19^T. GenBank accession numbers are given in parentheses. Percentages of bootstrap support are shown at branch nodes. Bootstrap value was 1000. Bar: 0.05 substitutions per nucleotide position. The phylogenominc tree based on gemome-sequence-alignment (c) and OrthoANI heatmap (d) of strain New-19^T and its neighbor species. Refseq ID of representative genomes are given in parentheses. Percentages of bootstrap support are shown at branch nodes. Bar: 0.20 substitutions per nucleotide position.

Taxon 89: *Lawsonibacter hominis* sp. nov.

Description *of Lawsonibacter hominis* sp. nov. (ho'mi.nis. L. gen. masc. n. *hominis*, of a human being, referring to the human gut habitat)
The taxon 89, respresented by strain NSJ-51^T (16S rRNA sequence accession number in NMDC is NMDCN000012O), is phylogenetically closest to *Lawsonibacter asaccharolyticus*, with 16S rRNA gene identity of 95.72 %. Phylogenetic tree shows that strain NSJ-51^T clusters with other members of the genus *Lawsonibacter,* (Figure SD-89a), suggesting strain NSJ-51^T is a member of the genus *Lawsonibacter*. The genome of strain NSJ-51^T was sequenced and the NMDC accession number is NMDC60014086. Genome-based analysis showed that the ANI value of genomes of strain NSJ-51^T and *Lawsonibacter asaccharolyticus* strain 3BBH22^T (NZ BFBT00000000) is 74.03 % and the dDDH estimation is 21.40 %. According to the phylogenominc tree (Figure SD-89c), the ANIs and dDDH values of genomes between strain NSJ-51^T (NMDC accession number is NMDC60014086) and phylogenomically neighbored genomes were calculated, and the highest ANI and dDDH values are 76.41 and 23.6 %, respectively, between the query genome of *Pseudoflavonifractor capillosus* (NZ_AAXG00000000.2); and difference in % G+C between genomes of strain NSJ-51^T and *Pseudoflavonifractor capillosus* (NZ_AAXG00000000.2) is 1.2; OrthoANI heatmap (Figure SD-89d) shows the phylogenomic status of the corresponding neighbor species based on OrthoANI values. Based on these results, we concluded that the strain NSJ-51^T represents of new species of the genus *Lawsonibacter*, and the name *Lawsonibacter hominis* sp. nov. is proposed.
Cells are strictly anaerobic, straight rods (2.0-2.5 µm long by 0.4-0.7 µm wide, Figure SD-89b); cells appear singly or in dividing pairs. Circular, white to translucent, moist, pinpoint colonies appear on modified mGAM agar plate after 1 day of incubation. Growth occurs at 37°C and at pH 7.0-7.5. The organism uses 3-melthyl-D-glucose, D-fructose, L-fucose, D-galactose, D-galacturonic acid, palatinose and L-rhamnose as the carbon source for growth, and weakly metabolizes α-D-glucose, D-mannose and pyruvic acid. The DNA G+C content of the type strain NSJ-51^T is 66.51 mol %. The type strain NSJ-51^T (=CGMCC 1.32825^T=KCTC 25134^T) was isolated from the faeces of a healthy adult.

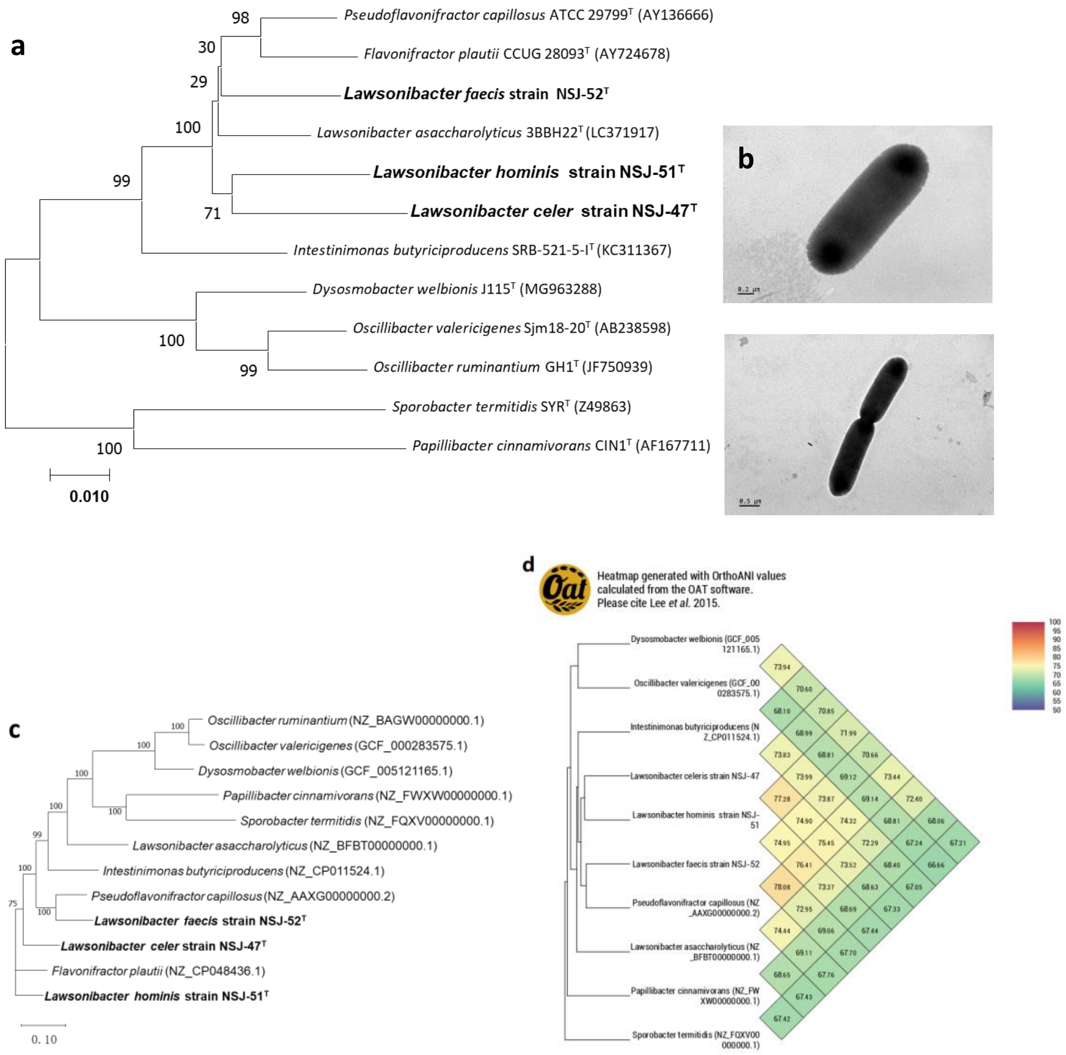

Figure SD-89. The Neighbour-joining phylogenetic tree based on 16S rRNA gene sequences (a) and the cell morphology (b) of strain NSJ-51^T. GenBank accession numbers are given in parentheses. Percentages of bootstrap support are shown at branch nodes. Bootstrap value was 1000. Bar: 0.01 substitutions per nucleotide position. The phylogenominc tree based on gemome-sequence-alignment (c) and OrthoANI heatmap (d) of strain NSJ-51^T and its neighbor species. Refseq ID of representative genomes are given in parentheses. Percentages of bootstrap support are shown at branch nodes. Bar: 0.10 substitutions per nucleotide position.

Taxon 90: *Lawsonibacter faecis* sp. nov.

Description *of Lawsonibacter faecis* sp. nov. (fae'cis. L. gen. fem. n. *faecis*, of faeces, from which the organism was isolated)

The taxon 90, respresented by strain NSJ-52^T (16S rRNA sequence accession number in NMDC is NMDCN000012P), is phylogenetically closest to *Lawsonibacter asaccharolyticus*, with 16S rRNA gene identity of 96.8 %. Phylogenetic tree shows that strain NSJ-52^T clusters with other members of the genus *Lawsonibacter,* (Figure SD-90a), suggesting strain NSJ-52^T is a member of the genus *Lawsonibacter*. The genome of strain NSJ-52^T was sequenced and the NMDC accession number is NMDC60014087. Genome-based analysis showed that the ANI value of genomes of strain NSJ-52^T and *Lawsonibacter asaccharolyticus* strain 3BBH22^T (NZ BFBT00000000) is 81.20 % and the dDDH estimation is 21.40 %. According to the phylogenominc tree (Figure SD-90c), the ANIs and dDDH values of genomes between strain NSJ-52^T (NMDC accession number is NMDC60014087) and phylogenomically neighbored genomes were calculated, and the highest ANI and dDDH values are 78.08 and 22.5 %, respectively, between the query genome of *Pseudoflavonifractor capillosus* (NZ_AAXG00000000.2); and difference in % G+C between genomes of strain NSJ-52^T and *Pseudoflavonifractor capillosus* (NZ_AAXG00000000.2) is 2.87; OrthoANI heatmap (Figure SD-90d) shows the phylogenomic status of the corresponding neighbor species based on OrthoANI values. Based on these results, we concluded that the strain NSJ-52^T represents of new species of the genus *Lawsonibacter*, and the name *Lawsonibacter faecis* sp. nov. is proposed.

Cells are strictly anaerobic, straight or slightly curved club-shaped rods (1.2-5.2 µm long by 0.6-0.9 µm wide, Figure SD-90b). Circular, white to translucent, moist, smooth, pinpoint colonies appear on modified mGAM agar plate after 2 days of incubation. Growth occurs at 37°C and at pH 7.0-7.5. The organism uses L-fucose, gentiobiose, D-gluconic acid, α-methyl-D glucoside, α-hydroxybutyric acid, D-lactic acidmethyl ester,L-malic acid, propionic acid, pyruvic acidmethyl ester, L-methionine, 2’-deoxyadenosine, inosine and uridine-5’-monophosphate as the carbon source for growth, and weakly metabolizes glucose-1-phosphate, glycerol, 3-melthyl-D-glucose, sucrose, formic acid, D-saccharic acid, succinamic acid, succinic acid, L-alanyl-L-threonine, L-serine, L-valine plus L-aspartic acid, thymidine and thymidine-5’-monophosphate. The DNA G+C content of the type strain NSJ-52^T is 67.26 mol %. The type strain NSJ-52^T (=CGMCC 1.32826^T =KCTC 25135^T) was isolated from the faeces of a healthy adult.

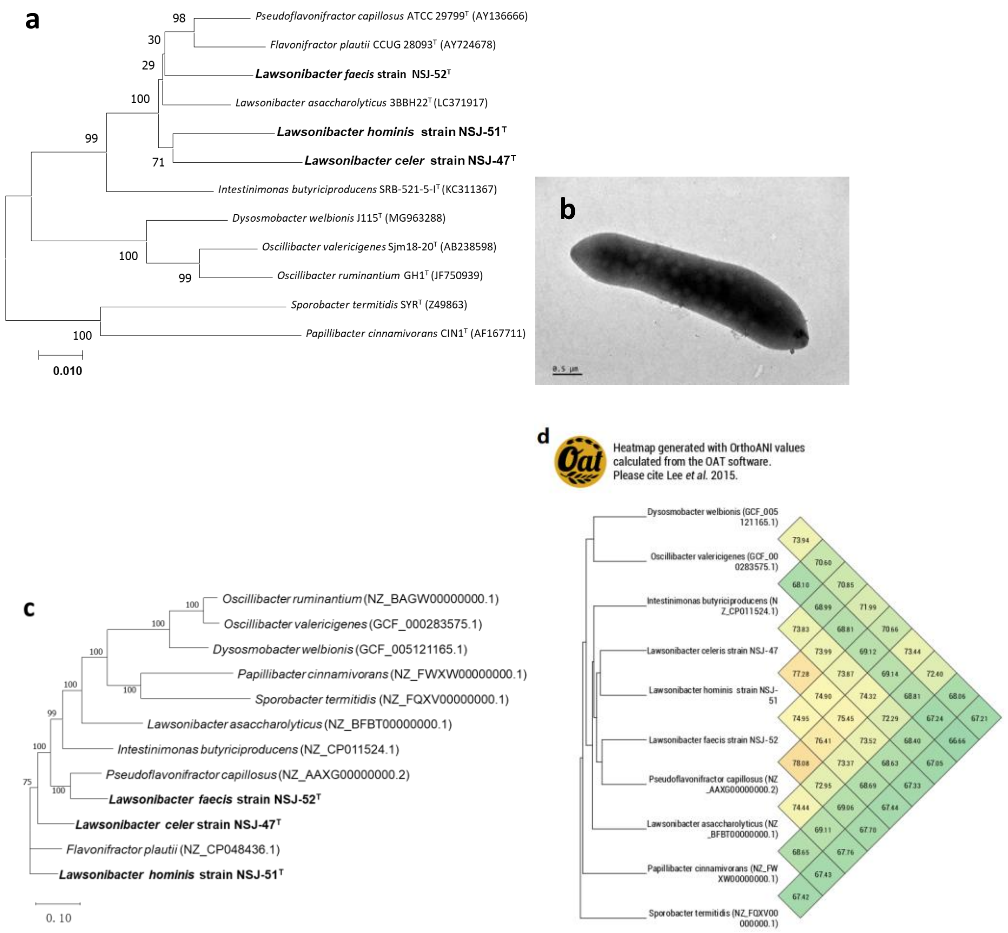

Figure SD-90. The Neighbour-joining phylogenetic tree based on 16S rRNA gene sequences (a) and the cell morphology (b) of strain NSJ-52^T. GenBank accession numbers are given in parentheses. Percentages of bootstrap support are shown at branch nodes. Bootstrap value was 1000. Bar: 0.01 substitutions per nucleotide position. The phylogenominc tree based on gemome-sequence-alignment (c) and OrthoANI heatmap (d) of strain NSJ-52^T and its neighbor species. Refseq ID of representative genomes are given in parentheses. Percentages of bootstrap support are shown at branch nodes. Bar: 0.1 substitutions per nucleotide position.

Taxon 91: *Lawsonibacter celer* sp. nov.

Description *of Lawsonibacter celer* sp. nov. (ce'ler. L. masc. adj. celer, rapid, pertaining to fast growth of the strain)

The taxon 91, respresented by strain NSJ-47^T (16S rRNA sequence accession number in NMDC is NMDCN000012Q), is phylogenetically closest to *Lawsonibacter asaccharolyticus*, with 16S rRNA gene identity of 95.9 %. Phylogenetic tree shows that strain NSJ-47^T clusters with the member of the genus *Lawsonibacter,* (Figure SD-91a), suggesting strain NSJ-47^T is a member of the genus *Lawsonibacter*. The genome of strain NSJ-47^T was sequenced and the NMDC accession number is NMDC60014088. Genome-based analysis showed that the ANI value of genomes of strain NSJ-47^T and *Lawsonibacter asaccharolyticus* 3BBH22^T (NZ BFBT00000000) is 76.76 % and the dDDH estimation is 21.20 %. According to the phylogenominc tree (Figure SD-91c), the ANIs and dDDH values of genomes between strain NSJ-47^T (NMDC accession number is NMDC60014088) and phylogenomically neighbored genomes were calculated, and the highest ANI and dDDH values are 77.28 and 21.5 %, respectively, between the query genome of *Lawsonibacter hominis* strain NSJ-51^T (NMDC60014086); and difference in % G+C between genomes of strain NSJ-47^T and *Lawsonibacter hominis* strain NSJ-51^T (NMDC60014086) is 0.26; OrthoANI heatmap (Figure SD-91d) shows the phylogenomic status of the corresponding neighbor species based on OrthoANI values. Based on these results, we concluded that the strain NSJ-47^T represents of new species of the genus *Lawsonibacter*, and the name *Lawsonibacter celer* sp. nov. is proposed.

Cells are strictly anaerobic straight rods (1.8-2.4 µm long by 0.4-0.8 µm wide,Figure SD-91b); cells appear singly. Growth occurs at 37°C and at pH 7.0-7.5. The organism uses dextrin, D-fructose, L-fucose, D-galactose, D-galacturonic acid, gentiobiose, α-D-glucose, glucose-6-phosphate, D-mannose, D-melibiose, 3-melthyl-D-glucose, palatinose,L-rhamnose, acetic acid, glyoxylic acid, α-hydroxybutyric acid, ß-hydroxybutyric acid, α-ketobutyric acid, D,L-lactic acid, L-lactic acid, D-lactic acid methyl ester,pyruvic acid, pyruvic acid methyl ester, L-serine, 2'-deoxyadenosine and inosine as the carbon source for growth, and weakly metabolizes D-cellobiose, i-erythritol, D-gluconic acid, glycerol, maltotriose, stachyose, sucrose, D-trehalose, turanose, formic acid, D-malic acid, L-malic acid, propionic acid, D-saccharic acid, m-tartaric acid and thymidine-5'-monophosphate. The DNA G+C content of the type strain NSJ-47^T is 64.83 mol %. The type strain NSJ-47^T (=CGMCC 1.32820^T) was isolated from the faeces of a healthy adult.

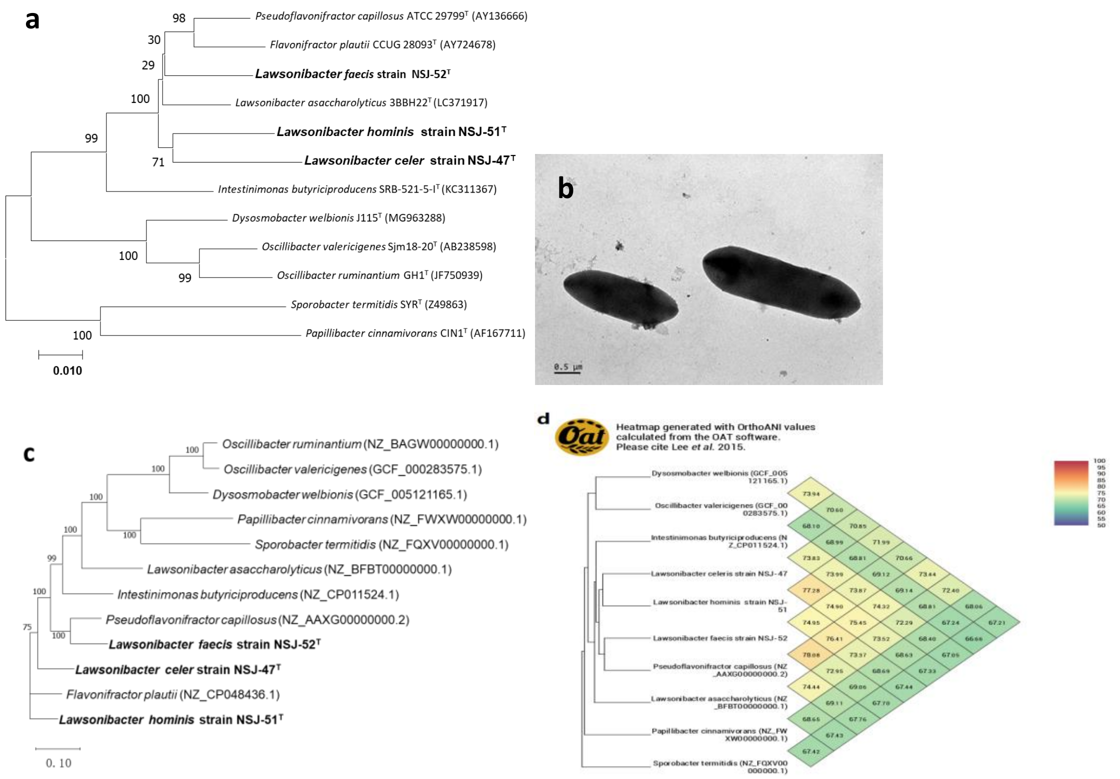

Figure SD-91. The Neighbour-joining phylogenetic tree based on 16S rRNA gene sequences (a) and the cell morphology (b) of strain NSJ-47^{T}. GenBank accession numbers are given in parentheses. Percentages of bootstrap support are shown at branch nodes. Bootstrap value was 1000. Bar: 0.01 substitutions per nucleotide position. The phylogenominc tree based on gemome-sequence-alignment (c) and OrthoANI heatmap (d) of strain NSJ-47^{T} and its neighbor species. Refseq ID of representative genomes are given in parentheses. Percentages of bootstrap support are shown at branch nodes. Bar: 0.10 substitutions per nucleotide position.

Taxon 92: *Neobittarella massiliensis* gen. sp.

Description of *Neobittarella* gen. nov. (Neo.bit.ta.rel'la N.L. fem. n. *Neobittarella*, in honor of microbiologist Fadi Bittar)

The closest phylogenetic neighbor of Taxon 92 as represented by strain NSJ-65^T (16S rRNA sequence accession number in NMDC is NMDCN000012R), is *Ethanoligenens harbinense* (Figure SD-92a). The genome of strain NSJ-65^T was sequenced and the NMDC accession number is NMDC60014089. *E. harbinense* and strain NSJ-65^T has 16S rRNA gene identity of 90.4 % and their DNA G+C content is different by 7.53 %. The genome-based analysis on *E. harbinense* strain and type strain NSJ-65^T reveals that the dDDH and ANI values between *E. harbinense* YUAN-3^T (NC 014828) and strain NSJ-65^T are 19.25 % and 68.30 %, respectively, suggesting *E. harbinense* and strain NSJ-65^T are from different genera of the family *Oscillospiraceae*. According to the phylogenominc tree (Figure SD-92c), the ANIs and dDDH values of genomes between strain NSJ-65^T (NMDC accession number is NMDC60014089) and phylogenomically neighbored genomes were calculated, and the highest ANI and dDDH values are 67.8446 and 22.6 %, respectively, between the query genome of *Ethanoligenens harbinense* (NZ_CP025288.1); and difference in % G+C between genomes of strain NSJ-65^T and *Ethanoligenens harbinense* (NZ_CP025288.1) is 3.67; OrthoANI heatmap (Figure SD-92d) shows the phylogenomic status of the corresponding neighbor species based on OrthoANI values. Cells of the strain NSJ-65^T are rod shaped. The G+C content is around 63.13 mol %. The type species is *Neobittarella massiliensis.*

Description of *Neobittarella massiliensis* sp. nov. (mas.si.li.en'sis L. masc./fem. adj. *massiliensis*, referring to Marseille, where the organism was isolated)

Cells are strictly anaerobic rods (1.5-2.8 μm long by 0.9-1.1 μm wide, Figure SD-92b); cells appear singly or in dividing pairs. Tiny, circular, yellow, entire, convex colonies appear on modified mGAM agar plate after 3 days of incubation. Growth occurs at 37°C and at pH 7.0-7.5. The organism uses dextrin, D-fructose, L-fucose, D-galactose, D-galacturonic acid, α-D-glucose, glucose-6-phosphate, maltose, D-mannose, D-melibiose, 3-melthyl-D-glucose, palatinose,glyoxylic acid, α-ketobutyric acid, α-ketovaleric acid, L-malic acid, pyruvic acid, L-alaninamide, L-alanine, glycyl-L-methionine, L-methionine, L-phenylalanine, L-serine, L-valine, L-valine plus L-aspartic acid, 2’-deoxyadenosine, inosine, thymidine, thymidine-5’-monophosphate, thymidine-5’-monophosphate, uridine-5’-monophosphate as the carbon source for growth, and weakly metabolizes D-cellobiose, glucose-1-phosphate, D-melezitose, turanose, L-alanyl-L-glutamine, L-alanyl-L-threonine and L-asparagine. The G+C content of the type strain NSJ-65^T is around 63.13 mol %. The type strain NSJ-65^T (=CGMCC 1.32841^T = KCTC 25131^T) was isolated from the faeces of a healthy adult.

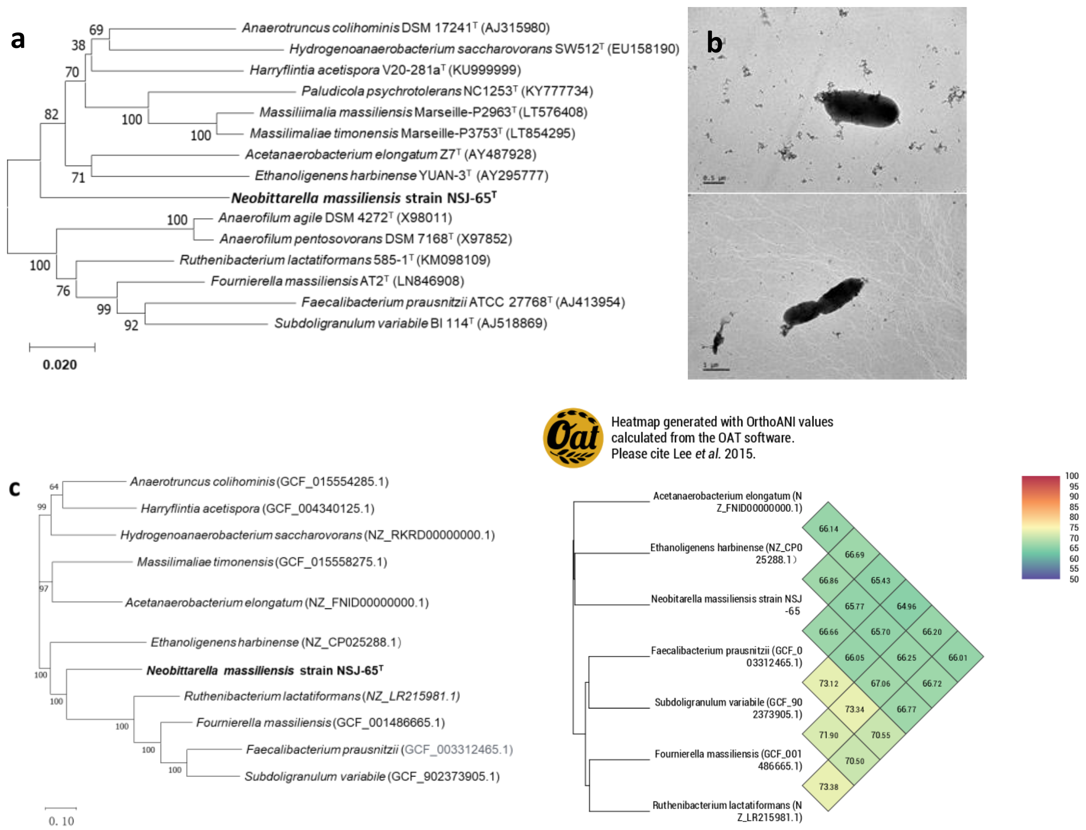

Figure SD-92. The Neighbour-joining phylogenetic tree based on 16S rRNA gene sequences (a) and the cell morphology (b) of strain NSJ-65^{T}. GenBank accession numbers are given in parentheses. Percentages of bootstrap support are shown at branch nodes. Bootstrap value was 1000. Bar: 0.02 substitutions per nucleotide position. The phylogenominc tree based on gemome-sequence-alignment (c) and OrthoANI heatmap (d) of strain NSJ-65^{T} and its neighbor species. Refseq ID of representative genomes are given in parentheses. Percentages of bootstrap support are shown at branch nodes. Bar: 0.1 substitutions per nucleotide position.

Taxon 93: *Oscillibacter hominis* sp. nov.

Description *of Oscillibacter hominis* sp. nov. (ho'mi.nis. L. gen. masc. n. *hominis*, of a human being, referring to the human gut habitat)

The taxon 93, respresented by strain NSJ-62^T (16S rRNA sequence accession number in NMDC is NMDCN000012S), is phylogenetically closest to *Oscillibacter valericigenes*, with 16S rRNA gene identity of 95.9 %. Phylogenetic tree shows that strain NSJ-62^T clusters with other members of the genus *Oscillibacter* (Figure SD-93a), suggesting strain NSJ-62^T is a member of the genus *Oscillibacter.* The genome of strain NSJ-62^T was sequenced and the NMDC accession number is NMDC60014090. Genome-based analysis showed that the ANI value of genomes of strain NSJ-62^T and *O. valericigenes* NBRC 101213^T (NC 016048) is 76.23 % and the dDDH estimation is 20.60 %. According to the phylogenominc tree (Figure SD-93c), the ANIs and dDDH values of genomes between strain NSJ-62^T (NMDC accession number is NMDC60014090) and phylogenomically neighbored genomes were calculated, and the highest ANI and dDDH values are 74.23 and 20.4 %, respectively, between the query genome of *Dysosmobacter welbionis* (GCF_005121165.1); and difference in % G+C between genomes of strain NSJ-62^T and *Dysosmobacter welbionis* (GCF_005121165.1) is 0.32; OrthoANI heatmap (Figure SD-93d) shows the phylogenomic status of the corresponding neighbor species based on OrthoANI values. Based on these results, we concluded that the strain NSJ-62^T represents of new species of the genus *Oscillibacter*, and the name *Oscillibacter hominis* sp. nov. is proposed.

Cells are strictly anaerobic, straight rods with straight spiky ends (1.4-2.1 µm long by 0.6-0.8 µm wide, Figure SD-93b); cells appear singly. Circular, pinpoint cream-colored, smooth, raised colonies appear on modified mGAM agar plate after 4 days of incubation at 37°C. Growth occurs at 37°C and at pH 7.0-7.5. The organism uses D-fructose, L-fucose, D-galacturonic acid and palatinose as the carbon source for growth, and weakly metabolizes D-galactose, α-D-glucose and palatinose. The DNA G+C content of the type strain NSJ-62^T is 53.16 mol %. The type strain NSJ-62^T (=CGMCC 1.32838^T=KCTC 25149^T) was isolated from the faeces of a healthy adult.

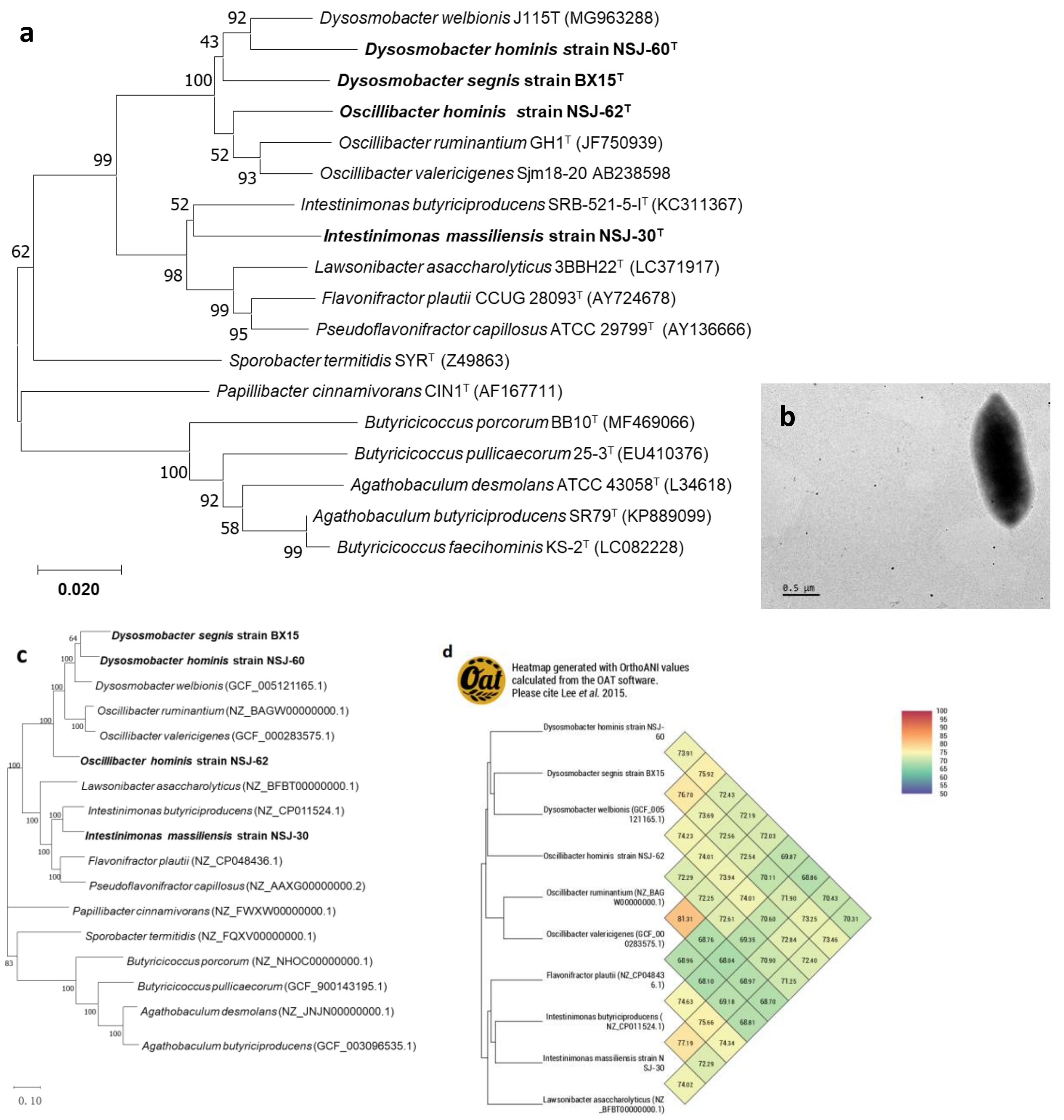

Figure SD-93. The Neighbour-joining phylogenetic tree based on 16S rRNA gene sequences (a) and the cell morphology (b) of strain NSJ-62^{T}. GenBank accession numbers are given in parentheses. Percentages of bootstrap support are shown at branch nodes. Bootstrap value was 1000. Bar: 0.02 substitutions per nucleotide position. The phylogenominc tree based on gemome-sequence-alignment (c) and OrthoANI heatmap (d) of strain NSJ-62^{T} and its neighbor species. Refseq ID of representative genomes are given in parentheses. Percentages of bootstrap support are shown at branch nodes. Bar: 0.1 substitutions per nucleotide position.

Taxon 94: *Pseudoflavonifractor hominis* sp. nov.

Description *of Pseudoflavonifractor hominis* sp. nov. (ho'mi.nis L. gen. masc. n. hominis, of a human being, referring to the human gut habitat)

The taxon 94, respresented by strain New-38^T (16S rRNA sequence accession number in NMDC is NMDCN000012T), is phylogenetically closest to *Pseudoflavonifractor capillosus*, with 16S rRNA gene identity of 98.1 %. Phylogenetic tree shows that strain New-38^T clusters with other members of the genus *Pseudoflavonifractor,* (Figure SD-94a), suggesting strain New-38^T is a member of genus *Pseudoflavonifractor*. The genome of strain New-38^T was sequenced and the NMDC accession number is NMDC60014091. Genome-based analysis showed that the ANI value of genomes of strain New-38^T and *P. capillosus* ATCC 29799^T (NZ AAXG00000000) is 75.37 % and the dDDH estimation is 22.30 %. According to the phylogenominc tree (Figure SD-94c), the ANIs and dDDH values of genomes between strain New-38^T (NMDC accession number is NMDC60014091) and phylogenomically neighbored genomes were calculated, and the highest ANI and dDDH values are 77.11 and 23.8 %, respectively, between the query genome of *Flavonifractor plautii* (NZ_SPHS00000000.1); and difference in % G+C between genomes of strain New-38^T and *Flavonifractor plautii* (NZ_SPHS00000000.1) is 0.59; OrthoANI heatmap (Figure SD-94d) shows the phylogenomic status of the corresponding neighbor species based on OrthoANI values. Based on these results, we concluded that the strain New-38^T represents of new species of the genus *Pseudoflavonifractor*, and the name *Pseudoflavonifractor hominis* sp. nov. is proposed.

Cells are strictly anaerobic, oval-shaped or rod-shaped in various sizes (1.1-4.0 µm long by 0.8-1.1 µm wide,Figure SD-94b); cells appear singly, in dividing pairs or in chains. Round to circular, cream-colored, semi-translucent, raised colonies appear on modified mGAM agar plate after 6 days of incubation. Growth occurs at 37°C and at pH 7.0-7.5. The organism uses D-fructose, L-fucose,D-galactose, D-galacturonic acid, palatinose and L-rhamnose as the carbon source for growth, and weakly metabolizes α-D-glucose, D-mannose, α-ketobutyric acid and pyruvic acid. The DNA G+C content of the type strain New-38^T is 63.72 mol %. The type strain New-38^T (=CGMCC 1.31611^T =KCTC 15862^T) was isolated from the faeces of a healthy adult.

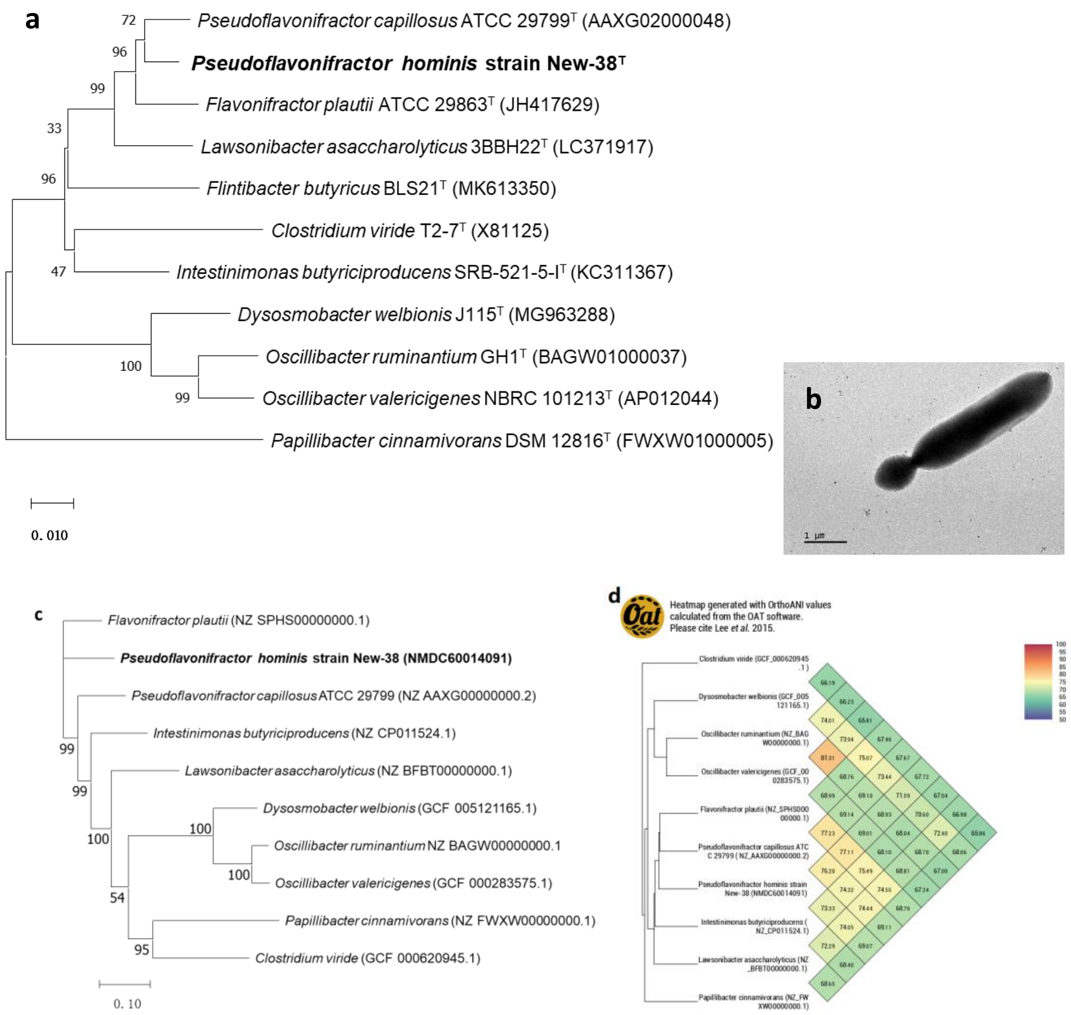

Figure SD-94. The Neighbour-joining phylogenetic tree based on 16S rRNA gene sequences (a) and the cell morphology (b) of strain New-38^T. GenBank accession numbers are given in parentheses. Percentages of bootstrap support are shown at branch nodes. Bootstrap value was 1000. Bar: 0.01 substitutions per nucleotide position. The phylogenominc tree based on gemome-sequence-alignment (c) and OrthoANI heatmap (d) of strain New-38^T and its neighbor species. Refseq ID of representative genomes are given in parentheses. Percentages of bootstrap support are shown at branch nodes. Bar: 0.10 substitutions per nucleotide position.

Taxon 95: *Ruminococcus lentus* sp. nov.

Description of *Ruminococcus lentus* sp. nov. (len'tus. L. masc. adj. *lentus*, slow, referring to the slow growth of the type strain; Nevertheless, *Ruminococcus bicirculans* strain 80/3, which shares 16S rRNA identity of 99.77% with the strain NSJ-14, was effectively but not validly published, and the nomenclature was incorrect, thus we propose *Ruminococcus lentus* as the valid species name).

The taxon 95, represented by strain NSJ-14^T (16S rRNA sequence accession number in NMDC is NMDCN000012U), is closest to *Ruminococcus albus*, with 16S rRNA gene identity of 94.28 %. Phylogenetic tree shows that strain NSJ-14^T clusters with other members of the genus *Ruminococcus* (Figure SD-95a), suggesting that strain NSJ-14^T is a member of the genus *Ruminococcus.* The genome of strain NSJ-14^T was sequenced and the NMDC accession number is NMDC60014092. Genome-based analysis on *Ruminococcus albus* strain 7^T (NC 014833) and type strain NSJ-14^T reveals that the ANI value is 71.73 % and the dDDH estimation is 22.60 %. According to the phylogenominc tree (Figure SD-95c), the ANIs and dDDH values of genomes between strain NSJ-14^T (NMDC accession number is NMDC60014092) and phylogenomically neighbored genomes were calculated, and the highest ANI and dDDH values are 71.03 and 22.6 %, respectively, between the query genome of Ruminococcus albus (KK211249); and difference in % G+C between genomes of strain NSJ-14^T and *Ruminococcus albus* (KK211249) is 0.72; OrthoANI heatmap (Figure SD-95d) shows the phylogenomic status of the corresponding neighbor species based on OrthoANI values. Based on these results, we concluded that the strain NSJ-14^T represents a new species of the genus *Ruminococcus*, and the name *Ruminococcus lentus* sp.nov. is proposed for validation.

Cells are strictly anaerobic, spherical in shape, cells appear singly or in dividing pairs, but no chains are observed, and dividing pairs are in shape of diplococci, but some are unequal in sizes (diameter 1.1-1.4 µm, Figure SD-95b). The organism uses D-fructose, L-fucose, D-galacturonic acid and palatinose as the carbon sources for growth, and weakly metabolizes dextrin, D-galactose, 3-melthyl-D-glucose, L-rhamnose, glyoxylic acid and pyruvic acid. The DNA G+C content of the type strain NSJ-14^T is 50.61 mol %. The type strain is NSJ-14^T (=CGMCC 1.5264^T =KCTC 15952^T), which was isolated from the faeces of a healthy adult.

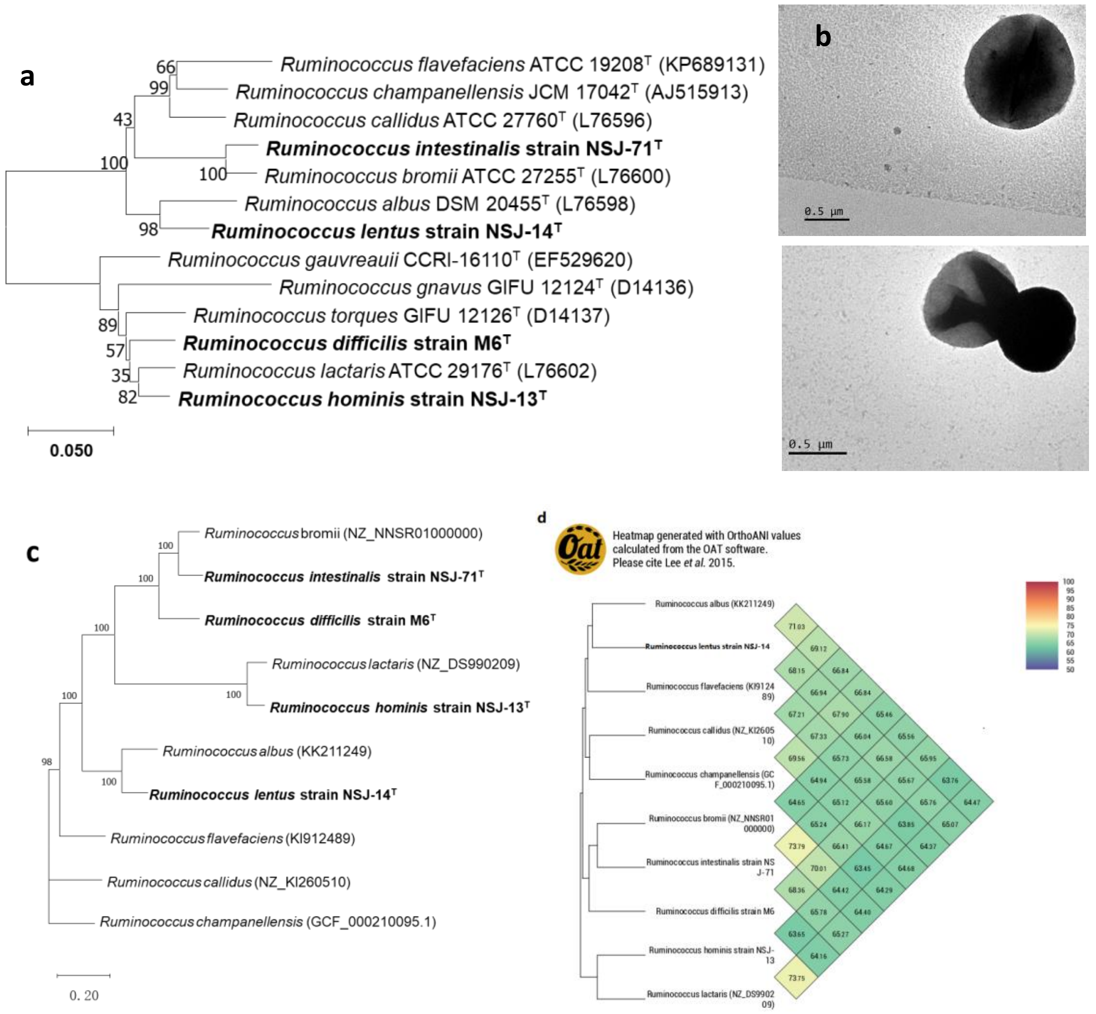

Figure SD-95. The Neighbour-joining phylogenetic tree based on 16S rRNA gene sequences (a) and the cell morphology (b) of strain NSJ-14^T. GenBank accession numbers are given in parentheses. Percentages of bootstrap support are shown at branch nodes. Bootstrap value was 1000. Bar: 0.05 substitutions per nucleotide position. The phylogenomic tree based on gemome-sequence-alignment (c) and OrthoANI heatmap (d) of strain NSJ-14^T and its neighbor species. Refseq ID of representative genomes are given in parentheses. Percentages of bootstrap support are shown at branch nodes. Bar: 0.20 substitutions per nucleotide position.

Taxon 96: *Ruminococcus intestinalis* sp. nov.

Description of *Ruminococcus intestinalis* sp. nov. (in.tes.ti.nal'is L. gen. n. intestinalis, of the intestine, the presumed habitat of the strain NSJ-71^T)

The taxon 96, represented by strain NSJ-71^T (16S rRNA sequence accession number in NMDC is NMDCN000012V), is closest to *Ruminococcus bromii*, with 16S rRNA gene identity of 96.61 %. Phylogenetic tree shows that strain NSJ-71^T clusters with other members of the genus *Ruminococcus* (Figure SD-96a), suggesting that strain NSJ-71^T is a member of the genus *Ruminococcus.* The genome of strain NSJ-71^T was sequenced and the NMDC accession number is NMDC60014093. Genome-based analysis on *Ruminococcus bromii* strain ATCC 27255^T (NZ FMUV00000000) and type strain NSJ-71^T reveals that the ANI value is 73.21 % and the dDDH estimation is 14.70 %. According to the phylogenominc tree (Figure SD-96c), the ANIs and dDDH values of genomes between strain NSJ-71^T (NMDC accession number is NMDC60014093) and phylogenomically neighbored genomes were calculated, and the highest ANI and dDDH values are 73.79 and 22.3 %, respectively, between the query genome of *Ruminococcus bromii* (NZ_NNSR01000000); and difference in % G+C between genomes of strain NSJ-71^T and *Ruminococcus bromii* (NZ_NNSR01000000) is 0.06; OrthoANI heatmap (Figure SD-96d) shows the phylogenomic status of the corresponding neighbor species based on OrthoANI values. Based on these results, we concluded that the strain NSJ-71^T represents a new species of the genus *Ruminococcus*, and the name *Ruminococcus intestinalis sp. nov.* is proposed.

Cells are strictly anaerobic, spherical or oval in shape (diameter 0.9-1.3 µm, Figure SD-96b); cells appear singly or in dividing pairs, dividing cells are equal in sizes and mostly in oval shaped rather than firmly spherical. Circular to irregular, ivory, flat colonies appear on modified mGAM agar plate after 3 days of incubation. Growth occurs at 37°C and at pH 7.0-7.5. The organism uses D-gluconic acid, glucose-6-phosphate, acetic acid, formic acid, glyoxylic acid, propionic acid and L-serine as the carbon source for growth, and weakly metabolizes D-lactic acid methyl ester, glucose-1-phosphate and 3-melthyl-D-glucose. The DNA G+C content of the type strain NSJ-71^T is 39.10 mol %. The type strain is NSJ-71^T (=CGMCC 1.32847^T), which was isolated from the faeces of a healthy adult.

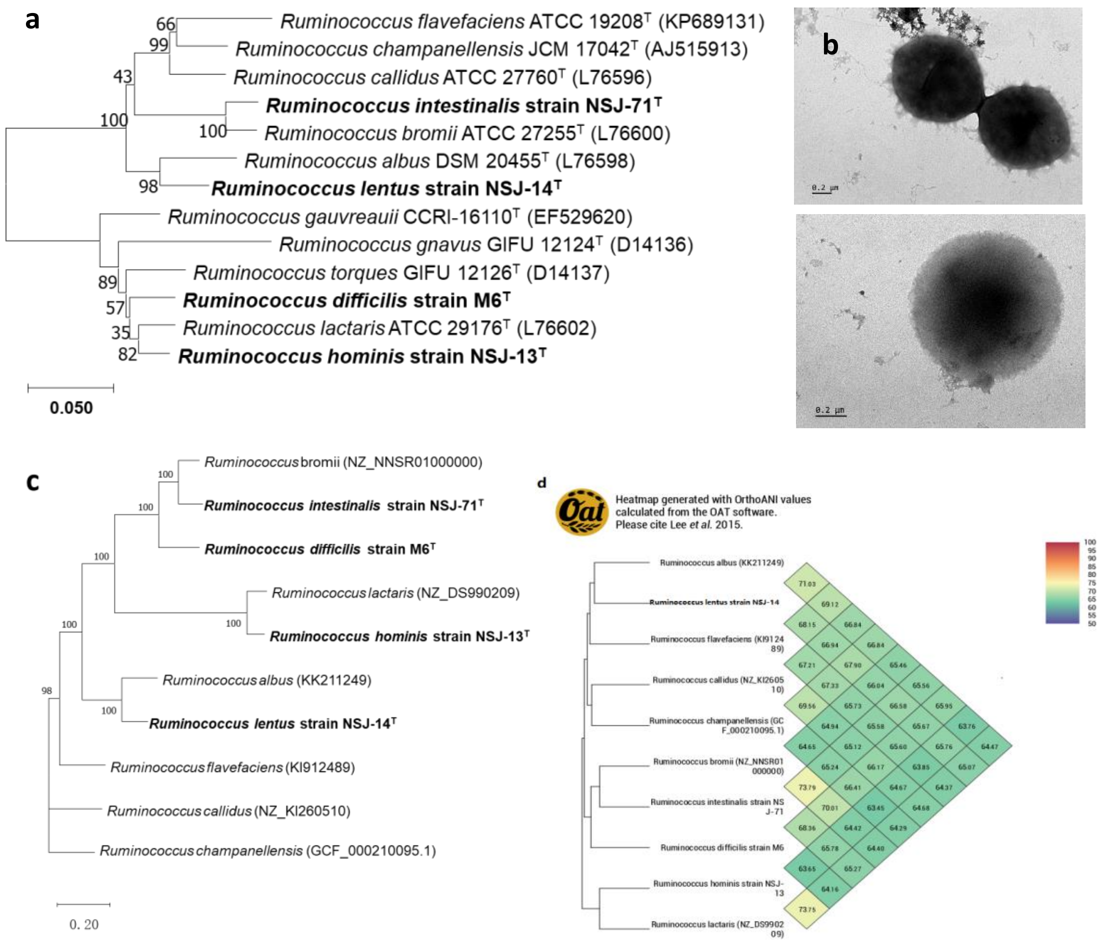

Figure SD-96. The Neighbour-joining phylogenetic tree based on 16S rRNA gene sequences (a) and the cell morphology (b) of strain NSJ-71^{T}. GenBank accession numbers are given in parentheses. Percentages of bootstrap support are shown at branch nodes. Bootstrap value was 1000. Bar: 0.05 substitutions per nucleotide position. The phylogenominc tree based on gemome-sequence-alignment (c) and OrthoANI heatmap (d) of strain NSJ-71^{T} and its neighbor species. Refseq ID of representative genomes are given in parentheses. Percentages of bootstrap support are shown at branch nodes. Bar: 0.20 substitutions per nucleotide position.

Taxon 97: *Paeniclostridium hominis* sp. nov.

Description *of Paeniclostridium hominis* sp. nov. (ho'mi.nis L. gen. masc. n. *hominis*, of a human being, referring to the human gut habitat)

The taxon 97, respresented by strain NSJ-45^T (16S rRNA sequence accession number in NMDC is NMDCN0000130), is phylogenetically closest to *Paeniclostridium ghonii*, with 16S rRNA gene identity of 98.8 %. Phylogenetic tree shows that strain NSJ-45^T clusters with other members of the genus *Paeniclostridium,* (Figure SD-97a), suggesting strain NSJ-45 ^{T} is a member of the genus *Paeniclostridium*. The genome of strain NSJ-45 ^{T} was sequenced and the NMDC accession number is NMDC60014094. Genome-based analysis showed that the ANI value of genomes of strain NSJ-45^T and *Paeniclostridium ghonii* DSM 20695^T (NC 014376) is 84.07 % and the dDDH estimation is 28.10 %. According to the phylogenominc tree (Figure SD-97c), the ANIs and dDDH values of genomes between strain NSJ-45 ^{T} (NMDC accession number is NMDC60014094) and phylogenomically neighbored genomes were calculated, and the highest ANI and dDDH values are 74.8543 and 20.3 %, respectively, between the query genome of *Asaccharospora irregularis* (NZ_FQWX00000000.1); and difference in % G+C between genomes of strain NSJ-45^T and *Asaccharospora irregularis* (NZ_FQWX00000000.1) is 3.24; OrthoANI heatmap (Figure SD-97d) shows the phylogenomic status of the corresponding neighbor species based on OrthoANI values. Based on these results, we concluded that the strain NSJ-45^T represents of new species of the genus *Paeniclostridium*, and the *Paeniclostridium hominis* sp. nov. is proposed.

Cells are strictly anaerobic and straight rods in various sizes (4.0-7.8 μm long by 1.2-1.8 μm wide, Figure SD-97b); cells appear singly or in dividing pairs. Growth occurs at 37°C and at pH 7.0-7.5. The organism uses dextrin, D-fructose, L-fucose, D-galactose, D-galacturonic acid, glucose-6-phosphate, gentiobiose, α-D-glucose, lactulose, maltotriose, D-melibiose, 3-melthyl-D-glucose, palatinose, L-rhamnose, glyoxylic acid, pyruvic acid and pyruvic acidmethyl ester as the carbon source for growth, and weakly metabolizes D-cellobiose, D-gluconic acid, D-glucosaminic acid, α-D-lactose, turanose, acetic acid, formic acid, α-ketobutyric acid and α-ketovaleric acid. The DNA G+C content of the type strain NSJ-45^T is 55.45 mol %. The type strain NSJ-45^T (=CGMCC 1.32818^T) was isolated from the faeces of a healthy adult.

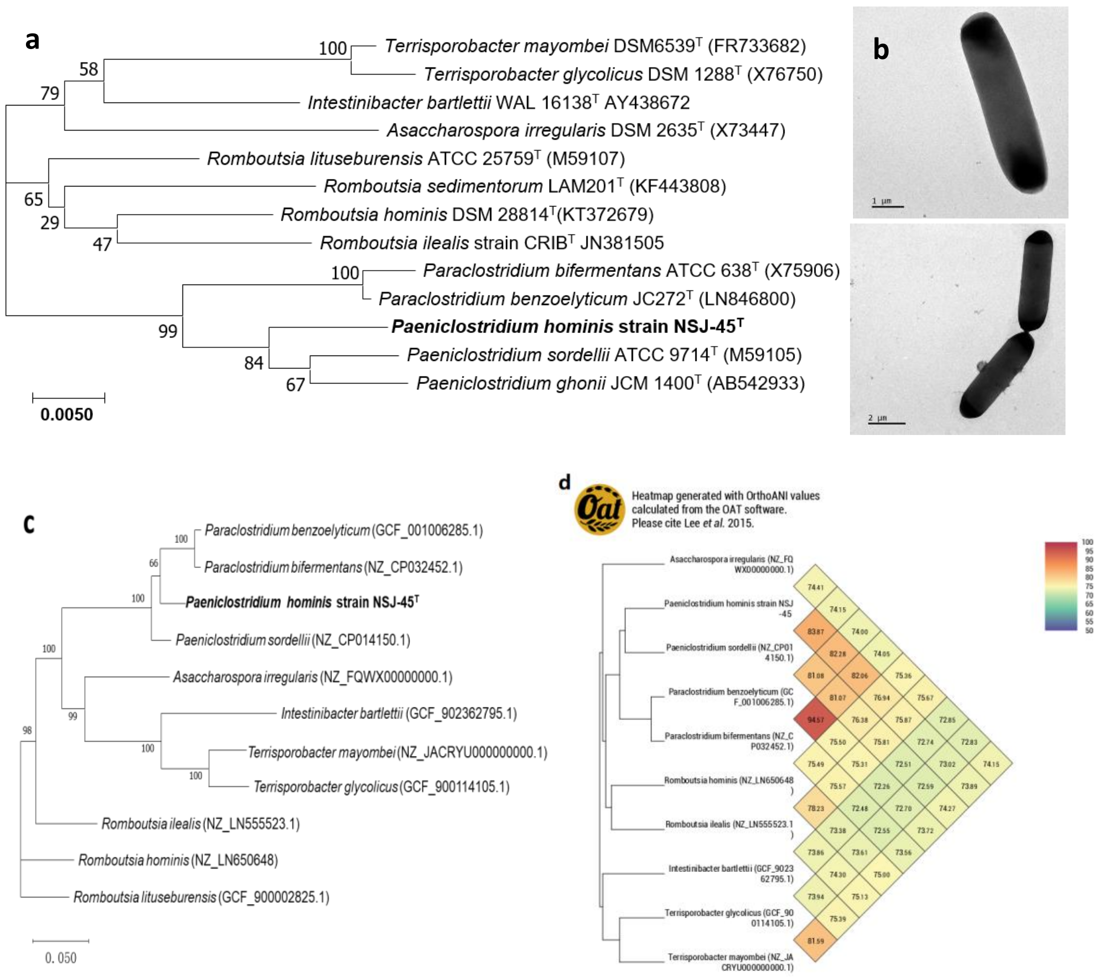

Figure SD-97. The Neighbour-joining phylogenetic tree based on 16S rRNA gene sequences (a) and the cell morphology (b) of strain NSJ-45^T. GenBank accession numbers are given in parentheses. Percentages of bootstrap support are shown at branch nodes. Bootstrap value was 1000. Bar: 0.005 substitutions per nucleotide position. The phylogenominc tree based on gemome-sequence-alignment (c) and OrthoANI heatmap (d) of strain NSJ-45^T and its neighbor species. Refseq ID of representative genomes are given in parentheses. Percentages of bootstrap support are shown at branch nodes. Bar: 0.05 substitutions per nucleotide position.

Taxon 98: *Romboutsia faecis* sp. nov.

Description *of Romboutsia faecis* sp. nov. (fae'cis L. gen. fem. n. *faecis*, referring to faecal origin)

The taxon 98, respresented by strain NSJ-18^T (16S rRNA sequence accession number in NMDC is NMDCN0000131), is phylogenetically closest to *Romboutsia ilealis*, with 16S rRNA gene identity of 97.5 %. Phylogenetic tree shows that strain NSJ-18^T clusters with other members of the genus *Romboutsia,* (Figure SD-98a), suggesting strain NSJ-18^T is a member of the genus *Romboutsia*. The genome of strain NSJ-18^T was sequenced and the NMDC accession number is NMDC60014107. Genome-based analysis showed that the ANI value of genomes of strain NSJ-18^T and *Romboutsia ilealis* CRIB^T (NZ RDBU00000000) is 77.70 % and the dDDH estimation is 22.80 %. According to the phylogenominc tree (Figure SD-98c), the ANIs and dDDH values of genomes between strain NSJ-18^T (NMDC accession number is NMDC60014107) and phylogenomically neighbored genomes were calculated, and the highest ANI and dDDH values are 74.2639 and 20.9 %, respectively, between the query genome of *Intestinibacter bartlettii* (GCF_902362795.1); and difference in % G+C between genomes of strain NSJ-18^T and *Intestinibacter bartlettii* (GCF_902362795.1) is 0.78; OrthoANI heatmap (Figure SD-98d) shows the phylogenomic status of the corresponding neighbor species based on OrthoANI values. Based on these results, we concluded that the strain NSJ-18^T represents of new species of the genus *Romboutsia*, and the name *Romboutsia faecis* sp. nov. is proposed.

Cells are strictly anaerobic, slightly curved or straight rods in various sizes (2.2–5.0 µm long by 1.1–1.4 µm wide, Figure SD-98b); cells appear singly or in (dividing) pairs. Irregular to round, yellow, thin, flat colonies (diameter 1.2 mm) appear on modified mGAM agar plate after 3 days of incubation. Growth occurs at 37°C and at pH 7.0-7.5. The organism uses glucose-6-phosphate, α-ketovaleric acid, pyruvic acid methyl ester, L-methionine, L-valine, 2'-deoxy adenosine and thymidine as the carbon source for growth. The DNA G+C content of the type strain NSJ-18^T is 51.91 mol %. The type strain NSJ-18^T (=CGMCC 1.31399^T) was isolated from the faeces of a healthy adult.

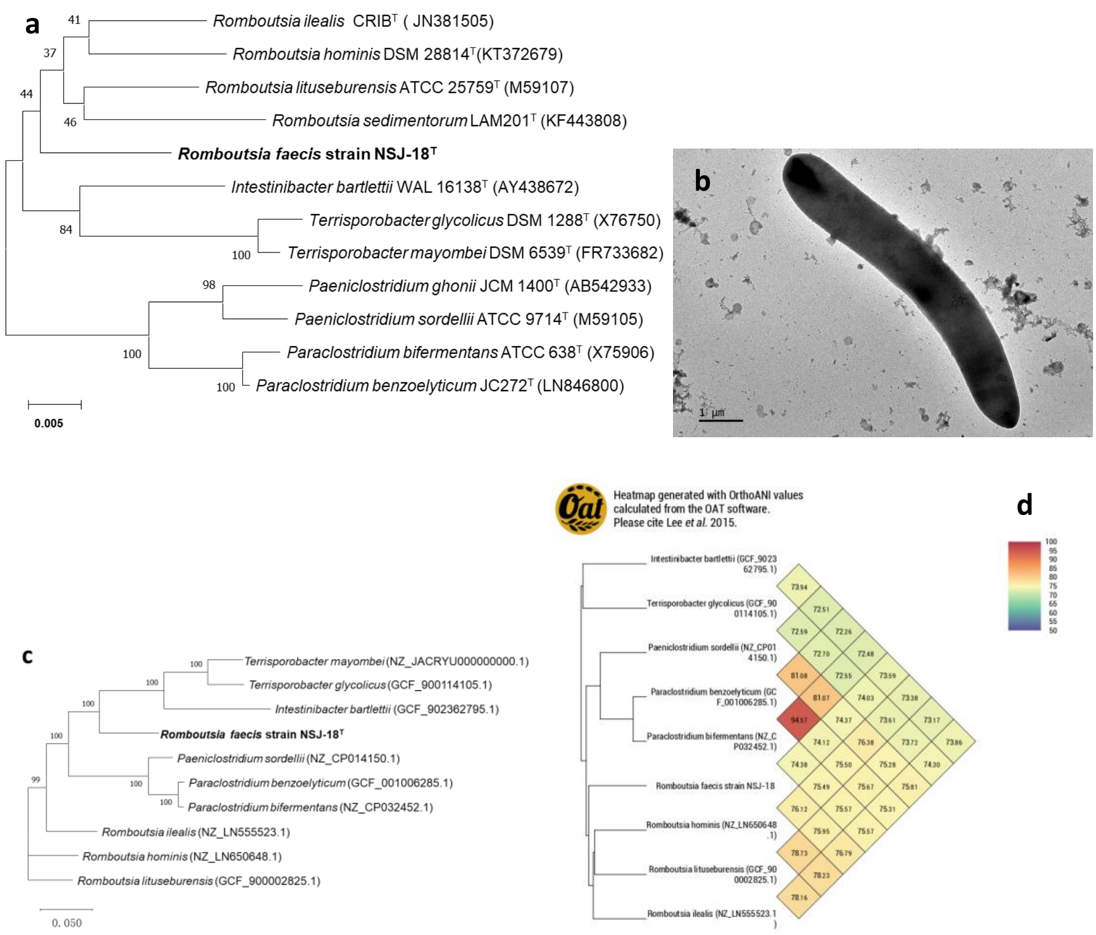

Figure SD-98. The Neighbour-joining phylogenetic tree based on 16S rRNA gene sequences (a) and the cell morphology (b) of strain NSJ-18^T. GenBank accession numbers are given in parentheses. Percentages of bootstrap support are shown at branch nodes. Bootstrap value was 1000. Bar: 0.005 substitutions per nucleotide position. The phylogenominc tree based on gemome-sequence-alignment (c) and OrthoANI heatmap (d) of strain NSJ-18^T and its neighbor species. Refseq ID of representative genomes are given in parentheses. Percentages of bootstrap support are shown at branch nodes. Bar: 0.05 substitutions per nucleotide position.

Taxon 99: *Intestinimonas massiliensis* sp. nov.

Description *of Intestinimonas massiliensis* sp. nov. (ma.si.li.e'n.sis. L. adj. masc. massiliensis, of Massilia, the Latin name of Marseill, where the bacteria was for the first time isolated, species name was given by Durand, But not validly published, here we present the characteristics of the type strain to validate)

The taxon 99, respresented by strain NSJ-30^T (16S rRNA sequence accession number in NMDC is NMDCN0000132), is phylogenetically closest to *Intestinimonas butyriciproducens*, with 16S rRNA gene identity of 95.0 %. Phylogenetic tree shows that strain NSJ-30^T clusters with the member of the genus *Intestinimonas* (Figure SD-99a), suggesting strain NSJ-30^T is a member of the genus *Intestinimonas.* The genome of strain NSJ-30^T was sequenced and the NMDC accession number is NMDC60014095. Genome-based analysis showed that the ANI value of genomes of strain NSJ-30^T and *Intestinimonas butyriciproducens* SRB-521-5-I^T (NZ CP011524) is 77.79 % and the dDDH estimation is 22.10 %. According to the phylogenominc tree (Figure SD-99c), the ANIs and dDDH values of genomes between strain NSJ-30^T (NMDC accession number is NMDC60014095) and phylogenomically neighbored genomes were calculated, and the highest ANI and dDDH values are 77.39 and 21.9 %, respectively, between the query genome of *Intestinimonas butyriciproducens* (NZ_CP011524.1); and difference in % G+C between genomes of strain NSJ-30^T and *Intestinimonas butyriciproducens* (NZ_CP011524.1) is 1.52; OrthoANI heatmap (Figure SD-99d) shows the phylogenomic status of the corresponding neighbor species based on OrthoANI values. Based on these results, we concluded that the strain NSJ-30^T represents of new species of the genus *Intestinimonas*, the name *Intestinimonas massiliensis* sp. is proposed species name was given by Durand, but has not been validated. Thus, we present the characteristics of the type strain to validate the species name as *Intestinimonas massiliensis*.

Cells are strictly anaerobic, straight or slightly curved rods (1.2-3.6 µm long by 0.5-0.8 µm wide, Figure SD-99b), occasionally with tapered ends. Circular, ivory, moist, convex colonies appear on modified mGAM agar plate after 3 days of incubation. Growth occurs at 37°C and at pH of 7.0-7.5. The organism uses L-fucose, 3-melthyl-D-glucose, palatinose, L-rhamnose, α-hydroxybutyric acid, ß-hydroxybutyric acid, α-ketobutyric acid, α-ketovaleric acid, D, L-lactic acid, L-lactic acid, D-lactic acid methyl ester, L-serine, 2'-deoxy adenosine, thymidine, uridine and thymidine-5'-monophosphate as the carbon source for growth, and weakly metabolizes D-arabitol, dextrin, D-fructose, L-alanyl-L-glutamine, L-alanyl-L-histidine, L-alanyl-L-threonine, L-methionine, L-threonine, uridine-5'-monophophate and inosine. The DNA G+C content of the type strain NSJ-30^T is 60.58 mol %. The type strain NSJ-30^T (=CGMCC 1.32803^T =KCTC 25082^T) was isolated from the faeces of a healthy adult.

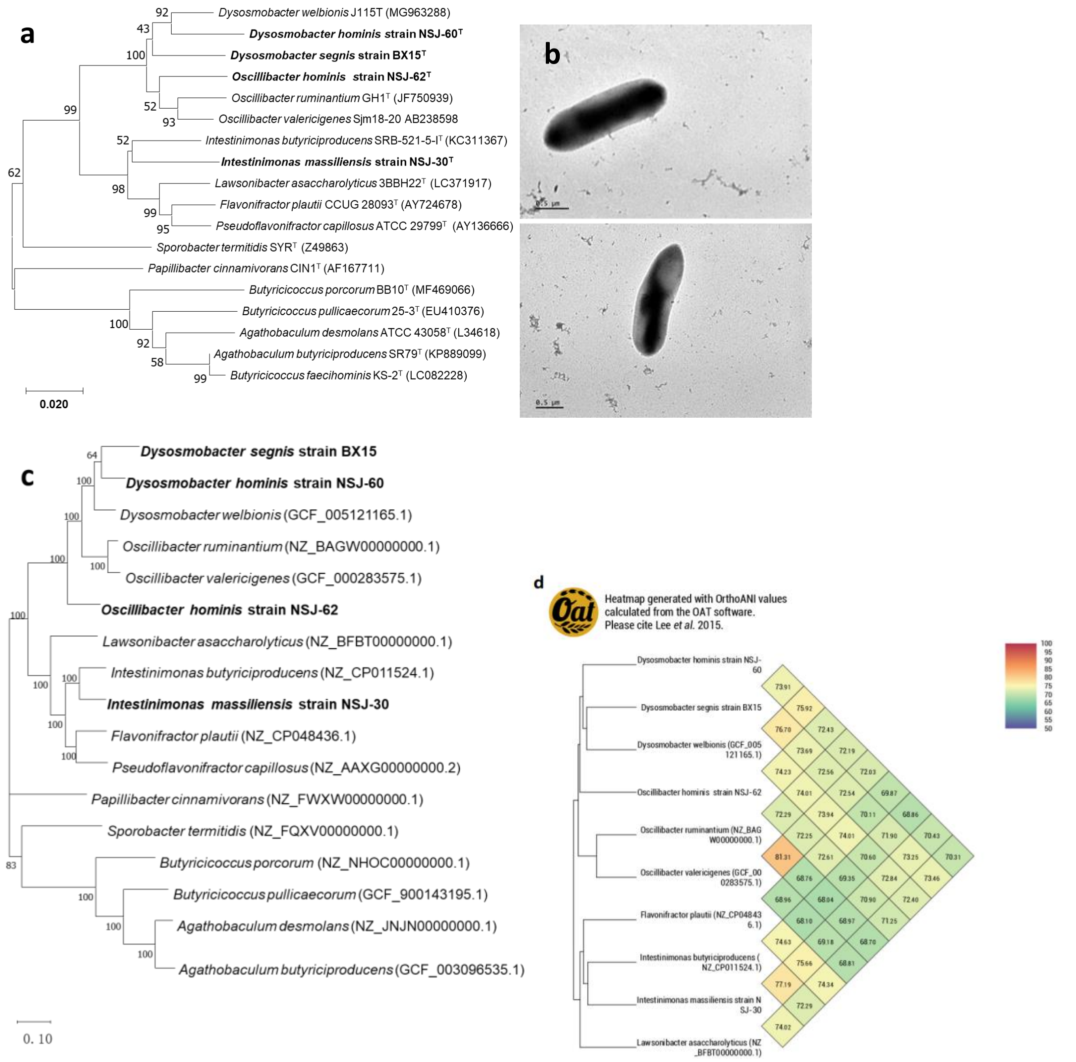

Figure SD-99. The Neighbour-joining phylogenetic tree based on 16S rRNA gene sequences (a) and the cell morphology (b) of strain NSJ-30^{T}. GenBank accession numbers are given in parentheses. Percentages of bootstrap support are shown at branch nodes. Bootstrap value was 1000. Bar: 0.02 substitutions per nucleotide position. The phylogenominc tree based on gemome-sequence-alignment (c) and OrthoANI heatmap (d) of strain NSJ-30^{T} and its neighbor species. Refseq ID of representative genomes are given in parentheses. Percentages of bootstrap support are shown at branch nodes. Bar: 0.1 substitutions per nucleotide position.

Taxon 100: *Hydrogeniiclostridium hominis* sp. nov.

Description *of Hydrogeniiclostridium hominis* sp. nov. (ho'mi.nis L. gen. masc. n. *hominis*, of a human being, referring to the human gut habitat)

The taxon 100, respresented by strain NSJ-41^T (16S rRNA sequence accession number in NMDC is NMDCN0000133), is phylogenetically closest to *Hydrogeniiclostridium mannosilyticum*, with 16S rRNA gene identity of 95.01 %. Phylogenetic tree shows that strain NSJ-41^T clusters with other members of the genus *Hydrogeniiclostridium,* (Figure SD-100a), suggesting strain NSJ-41^T is a member of the genus *Hydrogeniiclostridium*. The genome of strain NSJ-41^T was sequenced and the NMDC accession number is NMDC60014096. Genome-based analysis showed that the ANI value of genomes of strain NSJ-41^T and *Hydrogeniiclostridium mannosilyticum* ASß-methyl-D-galactoside818^T (NZ CBYL000000000) is 75.88 % and the dDDH estimation is 25.40 %. According to the phylogenominc tree (Figure SD-100c), the ANIs and dDDH values of genomes between strain NSJ-41^T (NMDC accession number is NMDC60014096) and phylogenomically neighbored genomes were calculated, and the highest ANI and dDDH values are 65.36 and 24.6 %, respectively, between the query genome of *Acetanaerobacterium elongatum* (NZ_FNID00000000.1); and difference in % G+C between genomes of strain NSJ-41^T and *Acetanaerobacterium elongatum* (NZ_FNID00000000.1) is 18.73; OrthoANI heatmap (Figure SD-100d) shows the phylogenomic status of the corresponding neighbor species based on OrthoANI values. Based on these results, we concluded that the strain NSJ-41^T represents of new species of the genus *Hydrogeniiclostridium*, and the name *Hydrogeniiclostridium hominis* sp. nov. is proposed.

Cells are strictly anaerobic, curved or straight rods in various sizes (1.5-8.0 µm long by 0.5-0.8 µm wide, Figure SD-100b); cells appear singly. Pinpoint, whitish, slightly raised colonies appear on modified mGAM agar plate after 3 days of incubation. Growth occurs at 37°C and at pH 7.0-7.5. The organism uses D-fructose, L-fucose, D-galacturonic acid, glucose-1-phosphate, glucose-6-phosphate and L-rhamnose as the carbon source for growth, and weakly metabolizes 3-melthyl-D-glucose. The DNA G+C content of the type strain NSJ-41^T is 53.47 mol %. The type strain NSJ-41^T (=CGMCC 1.32814^T =KCTC 25093^T) was isolated from the faeces of a healthy adult.

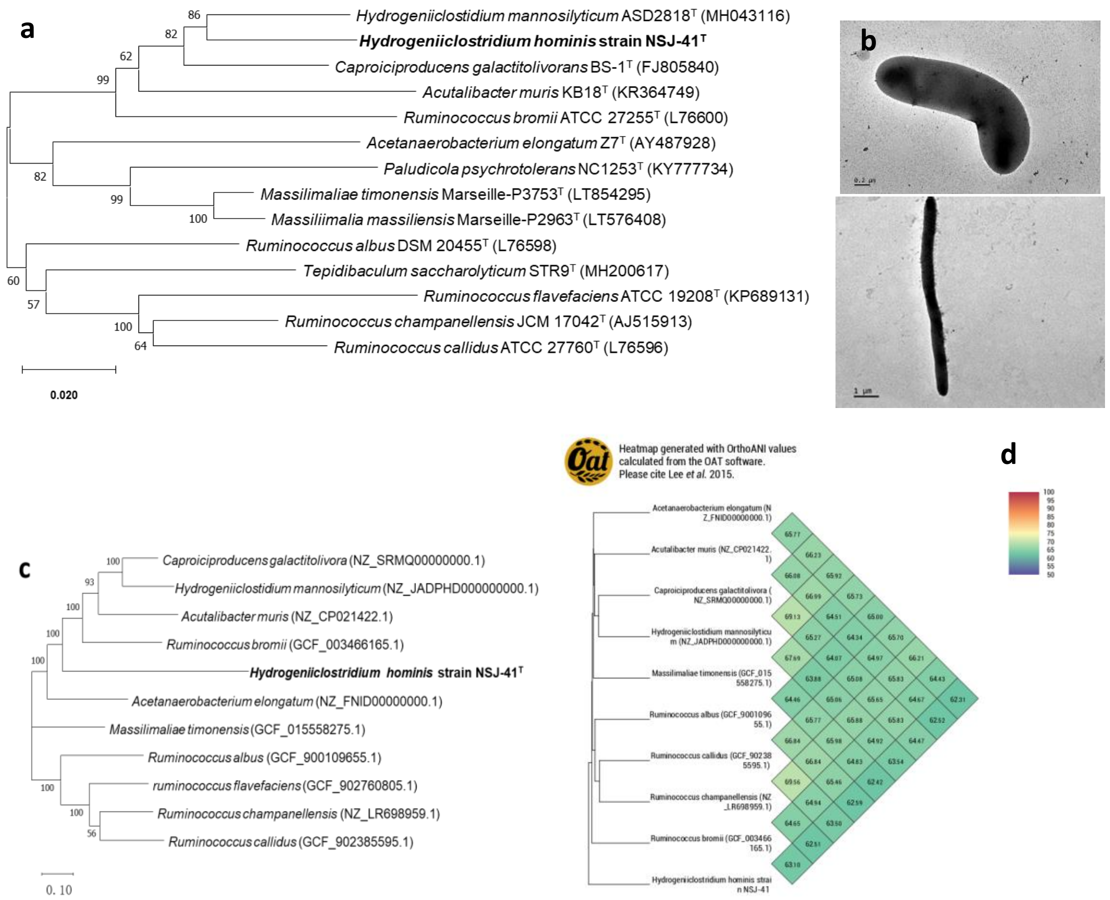

Figure SD-100. The Neighbour-joining phylogenetic tree based on 16S rRNA gene sequences (a) and the cell morphology (b) of strain NSJ-41^{T}. GenBank accession numbers are given in parentheses. Percentages of bootstrap support are shown at branch nodes. Bootstrap value was 1000. Bar: 0.02 substitutions per nucleotide position. The phylogenominc tree based on gemome-sequence-alignment (c) and OrthoANI heatmap (d) of strain NSJ-41^{T} and its neighbor species. Refseq ID of representative genomes are given in parentheses. Percentages of bootstrap support are shown at branch nodes. Bar: 0.1 substitutions per nucleotide position.

Taxon 101: *Catenibacterium faecis* sp. nov.

Description *of Catenibacterium faecis* sp. nov. (fae'cis. L. gen. fem. n. *faecis*, of faeces, from which the organism was isolated)

The taxon 101, respresented by strain NSJ-22^T (16S rRNA sequence accession number in NMDC is NMDCN0000134), is phylogenetically closest to *Catenibacterium mitsuoka*, with 16S rRNA gene identity of 95.79 %. Phylogenetic tree shows that strain NSJ-22^T clusters with other members of the genus *Catenibacterium* (Figure SD-101a), suggesting strain NSJ-22^T is a member of the genus *Catenibacterium.* The genome of strain NSJ-22^T was sequenced and the NMDC accession number is NMDC60014097. Genome-based analysis showed that the ANI value of genomes of strain NSJ-22^T and *C. mitsuokai* JCM 10609^T (NZ ACCK00000000) is 93.79 % and the dDDH estimation is 53.4 %, the GC % difference is 19.79 %. According to the phylogenominc tree (Figure SD-101c), the ANIs and dDDH values of genomes between strain NSJ-22^T (NMDC accession number is NMDC60014097) and phylogenomically neighbored genomes were calculated, and the highest ANI and dDDH values are 93.53 and 53.4 %, respectively, between the query genome of *Catenibacterium mitsuokai* (NZ_CABIWU000000000.1); and difference in % G+C between genomes of strain NSJ-22^T and *Catenibacterium mitsuokai* (NZ_CABIWU000000000.1) is 2.93; OrthoANI heatmap (Figure SD-101d) shows the phylogenomic status of the corresponding neighbor species based on OrthoANI values. Based on these results, we concluded that the strain NSJ-22^T represents of new species of the genus *Catenibacterium*, and the name *Catenibacterium faecis* sp. nov. is proposed.

Cells are strictly anaerobic rods (1.7–4.3 µm long by 0.4–0.7 µm wide, Figure SD-101b); cells appear singly, in dividing pairs or in chains. Tiny, circular, cream-colored, raised to convex, entire colonies appear on modified mGAM agar plate after 6 days of incubation. Growth occurs at 37°C and at pH 7.0-7.5. The organism uses acetyl-D glucosamine, amygdalin, arbutin, D-cellobiose, N-acetyl-β-D mannosamine, D-fructose, D-galactose, D-galacturonic acid, dextrin, gentiobiose, α-D-glucose, glucose-6-phosphate, α-D-lactose, maltose, maltotriose, D-mannose, D-melibiose, ß-methyl-D-glucoside, palatinose, D-raffinose, L-rhamnose, salicin, sucrose, turanose, α-ketobutyric acid, pyruvic acid, pyruvic acid methyl ester, L-methionine, inosine and thymidine as the carbon source for growth, and weakly metabolizes lactulose, 2'-deoxy adenosine. The DNA G+C content of the type strain NSJ-22^T is 55.29 mol %. The type strain NSJ-22^T (=CGMCC 1.31663^T) was isolated from the faeces of a healthy adult.

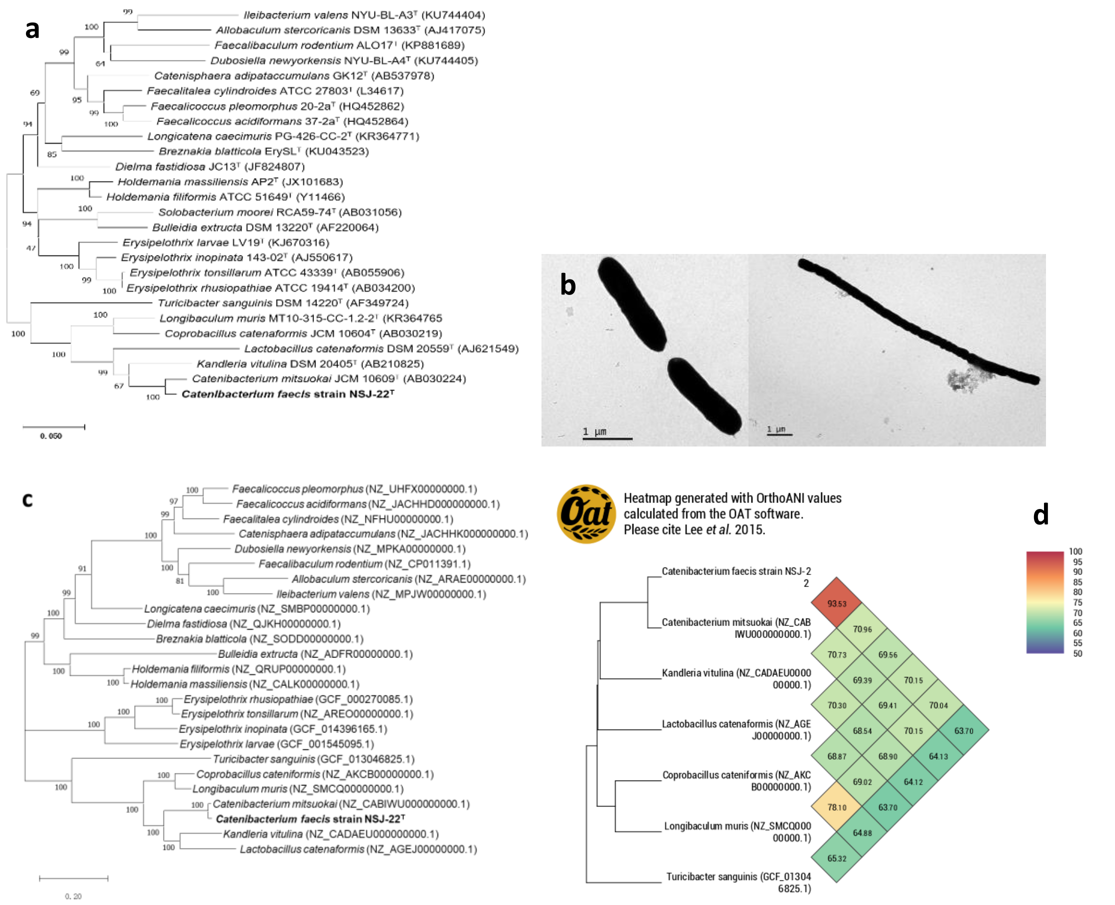

Figure SD-101. The Neighbour-joining phylogenetic tree based on 16S rRNA gene sequences (a) and the cell morphology (b) of strain NSJ-22^{T}. GenBank accession numbers are given in parentheses. Percentages of bootstrap support are shown at branch nodes. Bootstrap value was 1000. Bar: 0.05 substitutions per nucleotide position. The phylogenominc tree based on gemome-sequence-alignment (c) and OrthoANI heatmap (d) of strain NSJ-22^{T} and its neighbor species. Refseq ID of representative genomes are given in parentheses. Percentages of bootstrap support are shown at branch nodes. Bar: 0.2 substitutions per nucleotide position.

Taxon 102: *Eubacterium hominis* sp. nov.

Description of *Eubacterium hominis* sp. nov. (ho'mi.nis. L. gen. masc. n. *hominis*, of a human being, referring to the human gut habitat)

The taxon 1, represented by strain New-5^T (16S rRNA sequence accession number in NMDC is NMDCN0000135), is closest to *Eubacterium dolichum*, with 16S rRNA gene identity of 94.7 %. Phylogenetic tree shows that strain New-5^T clusters with other members of the genus *Eubacterium* (Figure SD-102a), suggesting that strain New-5^T is a member of the genus *Eubacterium.* The genome of strain New-5^T was sequenced and the NMDC accession number is NMDC60014098. Genome-based analysis on *E. dolichum* strain DSM 3991^T (NZ ABAW00000000) and type strain New-5^T reveals that the ANI value is 77.6 % and the dDDH estimation is 23.3 %. According to the phylogenominc tree (Figure SD-102c), the ANIs and dDDH values of genomes between strain New-5^T (NMDC accession number is NMDC60014098) and phylogenomically neighbored genomes were calculated, and the highest ANI and dDDH values are 70.52 and 23.3 %, respectively, between the query genome of *Eubacterium dolichum* (NZ_ABAW00000000.2); and difference in % G+C between genomes of strain New-5^T and *Eubacterium dolichum* (NZ_ABAW00000000.2) is 2.9; OrthoANI heatmap (Figure SD-102d) shows the phylogenomic status of the corresponding neighbor species based on OrthoANI values. Based on these results, we concluded that the strain New-5^T represents a new species of the genus *Eubacterium*, and the name *Eubacterium hominis* sp.nov. is proposed.

Cells are strictly anaerobic, straight rods with rounded ends (1.3-2.2 μm long by 0.6-0.8 μm wide, Figure SD-102b); cells appear singly or in (dividing) pairs. Round to irregular or occasionally spreading, white, smooth, and raised colonies (diameter 1 mm) appear on modified mGAM agar plate after 3 days of incubation at 37°C. Growth occurs at 37°C and at pH of 7.0-7.5. The organism uses N-acetyl-D galactosamine, acetyl-D glucosamine, N-acetyl-β-D mannosamine, amygdalin, dextrin, D-fructose, gentiobiose, α-D-glucose, glucose-1-phosphate, maltose, D-melezitose, 3-melthyl-D-glucose, palatinose, D-raffinose, D-sorbitol, D-trehalose, glyoxylic acid and α-ketobutyric acid as the carbon source for growth, and weakly metabolizes D-cellobiose, L-fucose, D-galactose, D-galacturonic acid, D-gluconic acid, glucose-6-phosphate, α-D-lactose, lactulose, maltotriose, D-mannitol, D-mannose, D-melibiose, sucrose, turanose. The DNA G+C content of the type strain New-5^T is 35.21 mol %. The type strain is New-5^T (=CGMCC 1.32837^T =KCTC 15860^T), which was isolated from the faeces of a healthy adult.

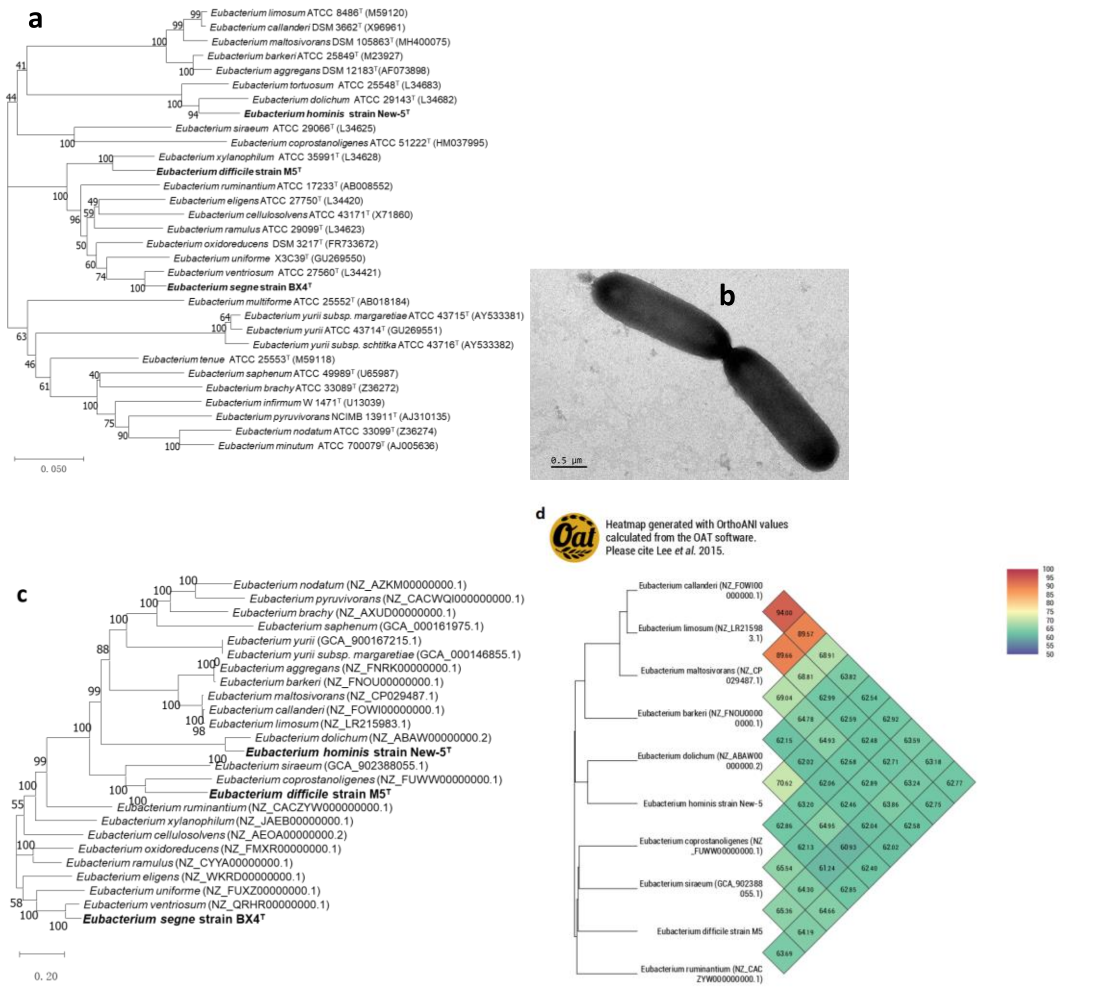

Figure SD-102. The Neighbour-joining phylogenetic tree based on 16S rRNA gene sequences (a) and the cell morphology (b) of strain New-5^T. GenBank accession numbers are given in parentheses. Percentages of bootstrap support are shown at branch nodes. Bootstrap value was 1000. Bar: 0.05 substitutions per nucleotide position. The phylogenominc tree based on gemome-sequence-alignment (c) and OrthoANI heatmap (d) of strain New-5^T and its neighbor species. Refseq ID of representative genomes are given in parentheses. Percentages of bootstrap support are shown at branch nodes. Bar: 0.2 substitutions per nucleotide position.

Taxon 103: *Holdemanella hominis* sp. nov.

Description *of Holdemanella hominis* sp. nov. (ho'mi.nis. L. gen. masc. n. *hominis*, of a human being, referring to the human gut habitat)

The taxon 103, respresented by strain L34^T (16S rRNA sequence accession number in NMDC is NMDCN0000136), is phylogenetically closest to *Holdemanella biformis*, with 16S rRNA gene identity of 99.21 %. Phylogenetic tree shows that strain L34^T clusters with the member of the genus *Holdemanella* (Figure SD-103a), suggesting strain L34^T is a member of the genus *Holdemanella.* The genome of strain L34^T was sequenced and the NMDC accession number is NMDC60014099. Genome-based analysis showed that the ANI value of genomes of strain L34^T and *Holdemanella biformis* DSM 3989^T (ABYT01) is 90.23 % and the dDDH estimation is 40.70 %. According to the phylogenominc tree (Figure SD-103c), the ANIs and dDDH values of genomes between strain L34^T (NMDC accession number is NMDC60014099) and phylogenomically neighbored genomes were calculated, and the highest ANI and dDDH values are 90 and 40.7 %, respectively, between the query genome of *Holdemanella biformis* (GCF_003464225.1); and difference in % G+C between genomes of strain L34^T and *Holdemanella biformis* (GCF_003464225.1) is 0.01; OrthoANI heatmap (Figure SD-103d) shows the phylogenomic status of the corresponding neighbor species based on OrthoANI values. Based on these results, we concluded that the strain L34^T represents of new species of the genus *Holdemanella*, and the name *Holdemanella hominis* sp. nov. is proposed.

Cells are strictly anaerobic, oval or rod-shaped with spiky ends (1.1-2.1 µm long by 0.5-0.9 µm wide, Figure SD-103b); cells appear mostly in dividing pairs or in chains, and rarely singly. Growth occurs at 37°C and at pH 7.0-7.5. The organism uses D-fructose, L-fucose, D-galactose, D-galacturonic acid, gentiobiose, α-D-glucose, glucose-6-phosphate, D-mannose, D-melibiose, 3-melthyl-D-glucose, palatinose, L-rhamnose, glyoxylic acid, α-ketobutyric acid, pyruvic acid and pyruvic acidmethyl ester as the carbon source for growth, and weakly metabolizes D-cellobiose and dextrin. The DNA G+C content of the type strain is 59.53 mol %. The type strain is L34^T (=CGMCC 1.32895^T), which was isolated from the faeces of a healthy adult.

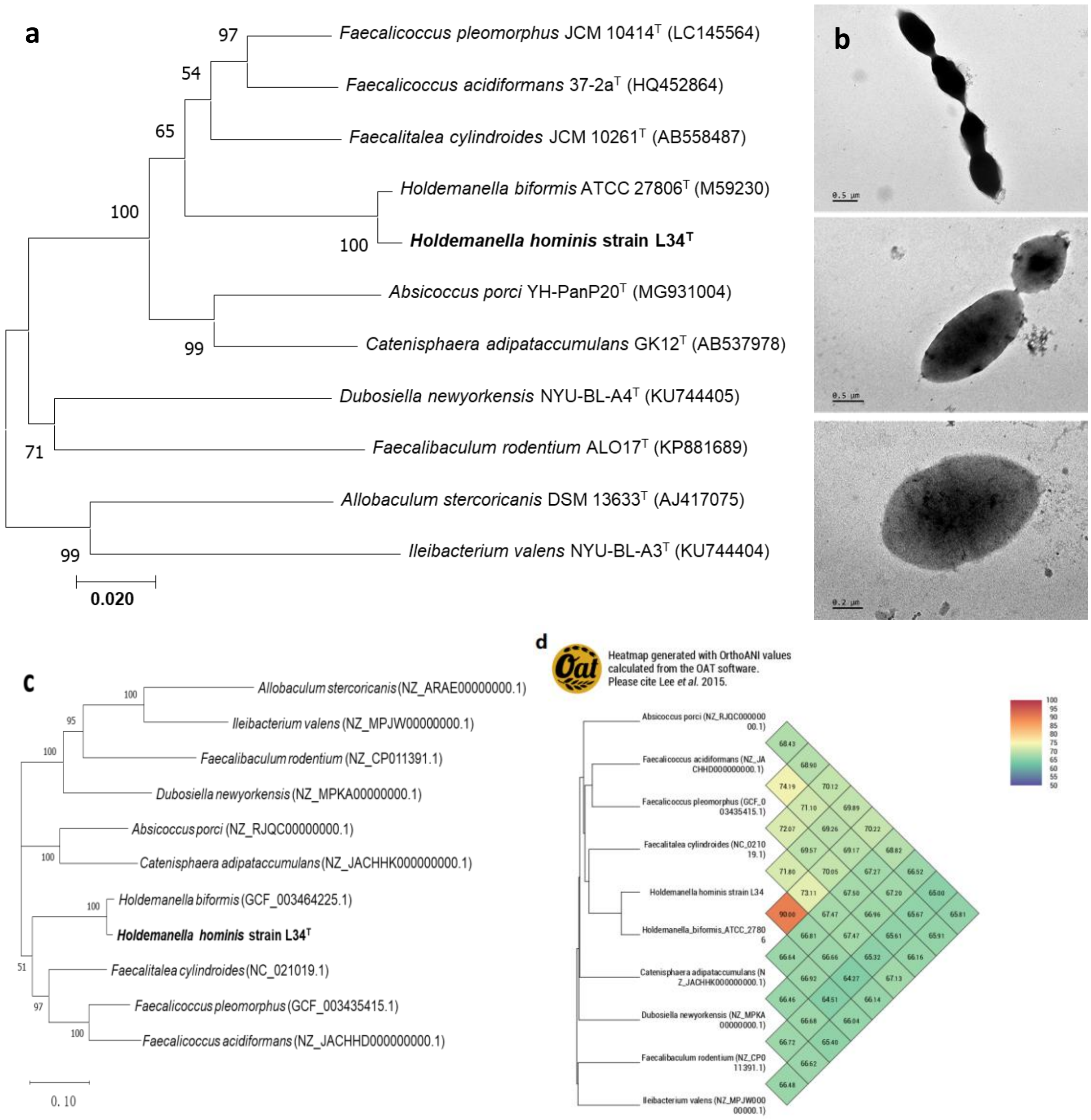

Figure SD-103. The Neighbour-joining phylogenetic tree based on 16S rRNA gene sequences (a) and the cell morphology (b) of strain L34^T. GenBank accession numbers are given in parentheses. Percentages of bootstrap support are shown at branch nodes. Bootstrap value was 1000. Bar: 0.02 substitutions per nucleotide position. The phylogenominc tree based on gemome-sequence-alignment (c) and OrthoANI heatmap (d) of strain L34^T and its neighbor species. Refseq ID of representative genomes are given in parentheses. Percentages of bootstrap support are shown at branch nodes. Bar: 0.1 substitutions per nucleotide position.

Taxon 104: ***Megasphaera hominis*** **sp. nov.**

Description ***of Megasphaera hominis*** **sp. nov.** (ho'mi.nis L. gen. masc. n. *hominis*, of a human being, referring to the human gut habitat)

The taxon 104, respresented by strain NSJ-59^T (16S rRNA sequence accession number in NMDC is NMDCN0000137), is phylogenetically closest to *Megasphaera hexanoica*[8], with 16S rRNA gene identity of 96.6 %. Phylogenetic tree shows that strain NSJ-59^T clusters with other members of the genus *Megasphaera* (Figure SD-104a), suggesting strain NSJ-59^T is a member of the genus *Megasphaera.* The genome of strain NSJ-59^T was sequenced and the NMDC accession number is NMDC60014100. Genome-based analysis showed that the ANI value of genomes of strain NSJ-59^T and *M. hexanoica* MH^T (NZ CP011940) is 75.37 % and the dDDH estimation is 20.4 %. According to the phylogenominc tree (Figure SD-104c), the ANIs and dDDH values of genomes between strain NSJ-59^T (NMDC accession number is NMDC60014100) and phylogenomically neighbored genomes were calculated, and the highest ANI and dDDH values are 75.41 and 20.1 %, respectively, between the query genome of *Megasphaera hexanoica* (CP011940.1); and difference in % G+C between genomes of strain NSJ-59^T and *Megasphaera hexanoica* (CP011940.1) is 1.37; OrthoANI heatmap (Figure SD-104d) shows the phylogenomic status of the corresponding neighbor species based on OrthoANI values. Based on these results, we concluded that the strain NSJ-59^T represents of new species of the genus *Megasphaera*, and the name *Megasphaera hominis* sp. nov. is proposed

Cells are strictly anaerobic, oval shaped with slightly spiky ends (1.1-1.4 µm long by 0.9-1.0 µm wide, Figure SD-104b); cells appear singly or in dividing pairs. Circular or irregular, translucent, smooth, raised, entire colonies appear on modified mGAM agar plate after 3 days of incubation. Growth occurs at 37°C and at pH of 7.0-7.5. The organism uses D-cellobiose, dextrin, D-gluconic acid, α-D-glucose, D-melezitose, palatinose, turanose, D-lactic acid methyl ester and urocanic acid as the carbon source for growth, and weakly metabolizes maltose, amygdalin, β-cyclodextrin, D-galacturonic acid, gentiobiose, glucose-1-phosphate, glucose-6-phosphate, maltotriose, D-mannose, D-melibiose, 3-melthyl-D-glucose, sucrose, glyoxylic acid, α-hydroxybutyric acid, α-ketobutyric acid, α-ketovaleric acid, D, L-lactic acid, L-lactic acid, pyruvic acid and pyruvic acid methyl ester. The DNA G+C content of the type strain NSJ-59^T is 56.12 mol %. The type strain NSJ-59^T (=CGMCC 1.32835^T =KCTC 25147^T) was isolated from the faeces of a healthy adult.

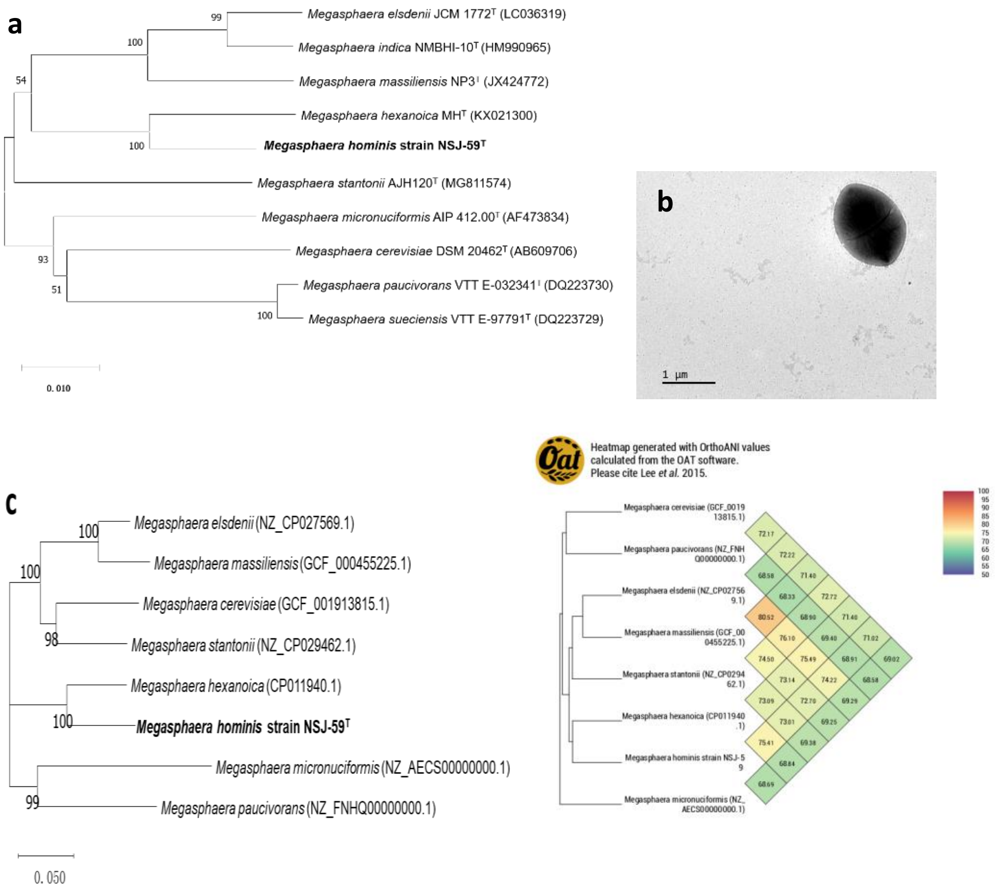

Figure SD-104. The Neighbour-joining phylogenetic tree based on 16S rRNA gene sequences (a) and the cell morphology (b) of strain NSJ-59^{T}. GenBank accession numbers are given in parentheses. Percentages of bootstrap support are shown at branch nodes. Bootstrap value was 1000. Bar: 0.01 substitutions per nucleotide position. The phylogenominc tree based on gemome-sequence-alignment (c) and OrthoANI heatmap (d) of strain NSJ-59^{T} and its neighbor species. Refseq ID of representative genomes are given in parentheses. Percentages of bootstrap support are shown at branch nodes. Bar: 0.05 substitutions per nucleotide position.

Taxon 105: *Veillonella hominis* sp. nov.

Description of *Veillonella hominis* sp. nov. (ho'mi.nis L. gen. masc. n. *hominis*, of a human being, referring to the human gut habitat)

The taxon 105, represented by strain NSJ-78^T (16S rRNA sequence accession number in NMDC is NMDCN0000138), is closest to *Veillonella parvula*, with 16S rRNA gene identity of 98.34 %. Phylogenetic tree shows that strain NSJ-78^T clusters with other members of the genus *Veillonella* (Figure SD-105a), suggesting that strain NSJ-78^T is a member of the genus *Veillonella.* The genome of strain NSJ-78^T was sequenced and the NMDC accession number is NMDC60014101. Genome-based analysis on *Veillonella parvula* strain DSM 2008^T (NC 013520) and type strain NSJ-78^T reveals that the ANI value is 95.24 % and the dDDH estimation is 61 %. According to the phylogenominc tree (Figure SD-105c), the ANIs and dDDH values of genomes between strain NSJ-78^T (NMDC accession number is NMDC60014101) and phylogenomically neighbored genomes were calculated, and the highest ANI and dDDH values are 95.25 and 61 %, respectively, between the query genome of *Veillonella parvula* (NC_013520.1); and difference in % G+C between genomes of strain NSJ-78^T and *Veillonella parvula* (NC_013520.1) is 0.01; OrthoANI heatmap (Figure SD-105d) shows the phylogenomic status of the corresponding neighbor species based on OrthoANI values. Based on these results, we concluded that the strain NSJ-78^T represents a new species of the genus *Veillonella*, and the name *Veillonella hominis sp. nov.* is proposed.

Cells are strictly anaerobic, spherical in shape, cells appear singly or in dividing pairs, no chains are observed, and dividing pairs are in shape of tetracocci (diameter 1.3-1.6 µm, Figure SD-105b). The organism uses D-fructose, L-fucose, D-galactose, D-galacturonic acid, gentiobiose, D-mannose, 3-melthyl-D-glucose, palatinose, L-rhamnose, glyoxylic acid, α-hydroxybutyric acid, α-ketobutyric acid, D,L-lactic acid, L-lactic acid and pyruvic acid as carbon source for growth, and weakly metabolizes α-D-glucose, D-melibiose, D-lactic acid methyl ester, pyruvic acid methyl ester, succinic acid and m-tartaric acid. The DNA G+C content of the type strain NSJ-78^T is 53.79 mol %. The type strain is NSJ-78^T (=CGMCC 1.32854^T= KCTC 25159^T), which was isolated from the faeces of a healthy adult.

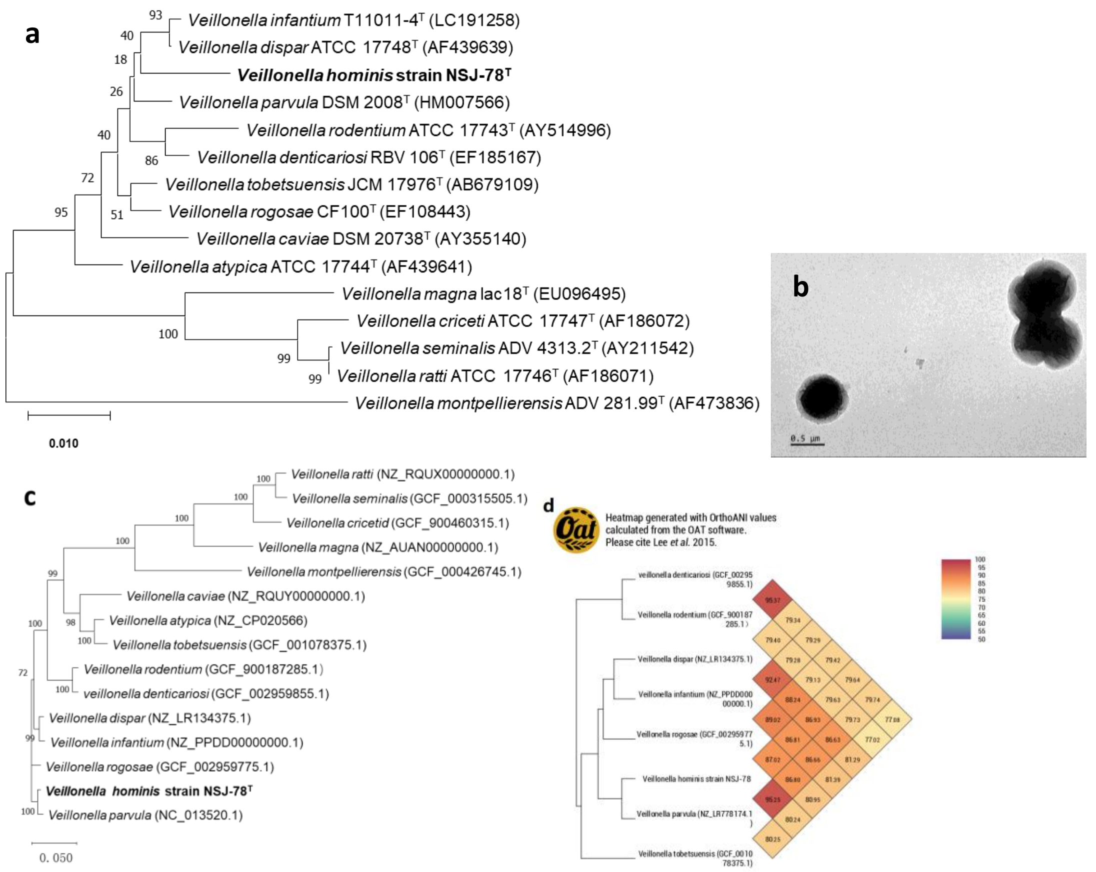

Figure SD-105. The Neighbour-joining phylogenetic tree based on 16S rRNA gene sequences (a) and the cell morphology (b) of strain NSJ-78^T. GenBank accession numbers are given in parentheses. Percentages of bootstrap support are shown at branch nodes. Bootstrap value was 1000. Bar: 0.01 substitutions per nucleotide position. The phylogenominc tree based on gemome-sequence-alignment (c) and OrthoANI heatmap (d) of strain NSJ-78^T and its neighbor species. Refseq ID of representative genomes are given in parentheses. Percentages of bootstrap support are shown at branch nodes. Bar: 0.05 substitutions per nucleotide position.

Taxon 106: *Tissierella hominis* sp. nov.

Description *of Tissierella hominis* sp. nov. (ho'mi.nis L. gen. masc. n. *hominis*, of a human being, referring to the human gut habitat)

The taxon 106, respresented by strain NSJ-26^T (16S rRNA sequence accession number in NMDC is NMDCN0000139), is phylogenetically closest to *Tissierella creatinophila*, with 16S rRNA gene identity of 95.26 %. Phylogenetic tree shows that strain NSJ-26^T clusters with other members of the genus *Tissierella,* (Figure SD-106a), suggesting strain NSJ-26^T is a member of the genus *Tissierella*. The genome of strain NSJ-26^T was sequenced and the NMDC accession number is NMDC60014102. Genome-based analysis showed that the ANI value of genomes of strain NSJ-26^T and *Tissierella creatinophila* KRfumaric acid^T (NZ LTDM00000000) is 75.30 % and the dDDH estimation is 20.80 %. According to the phylogenominc tree (Figure SD-106c), the ANIs and dDDH values of genomes between strain NSJ-26^T (NMDC accession number is NMDC60014102) and phylogenomically neighbored genomes were calculated, and the highest ANI and dDDH values are 72.77 and 20.80 %, respectively, between the query genome of *Tissierella creatinophila* (NZ_LTDM00000000.1); and difference in % G+C between genomes of strain NSJ-26^T and *Tissierella creatinophila* (NZ_LTDM00000000.1) is 0.18; OrthoANI heatmap (Figure SD-106d) shows the phylogenomic status of the corresponding neighbor species based on OrthoANI values. Based on these results, we concluded that the strain NSJ-26^T represents of new species of the genus *Tissierella*, and the name *Tissierella hominis* sp. nov. is proposed.

Cells are strictly anaerobic, short rod shaped (1.9-2.8 μm long by 0.8-1.2 μm wide) with amphitrichous flagella (Figure SD-106b); cells appear singly or in (dividing) pairs. Semi-translucent, moist, raised, pinpoint colonies appear on modified mGAM agar plate after 3 days of incubation. Growth occurs at 37°C and at pH of 7.0-7.5. The organism uses D-fructose, L-fucose, D-galactose, D-galacturonic acid, gentiobiose, α-D-glucose, glucose-6-phosphate, D-mannose, D-melezitose, 3-melthyl-D-glucose, palatinose, L-rhamnose, turanose, fumaric acid, α-ketobutyric acid, α-ketovaleric acid, pyruvic acid, L-alanyl-L-histidine, L-alanyl-L-threonine, L-methionine, L-serine, L-threonine, L-valine, thymidine and uridine as the carbon source for growth, and weakly metabolizes acetyl-D glucosamine, N-acetyl-β-D mannosamine, L-α-glycerol phosphate, α-methyl-D-galactoside, L-malic acid, urocanic acid, L-alaninamide, L-alanine, L-alanyl-L-glutamine, glycyl-L-aspartic acid, glycyl-L-methionine, L-valine plus L-asparagine,L-aspartic acid, D-sorbitol, adonitol, dextrin, glycerol, 2'-deoxy adenosine and inosine. The DNA G+C content of the type strain NSJ-26^T is 51.85 mol %. The type strain NSJ-26^T (=CGMCC 1.31394^T = KCTC 25080^T) was isolated from the faeces of a healthy adult.

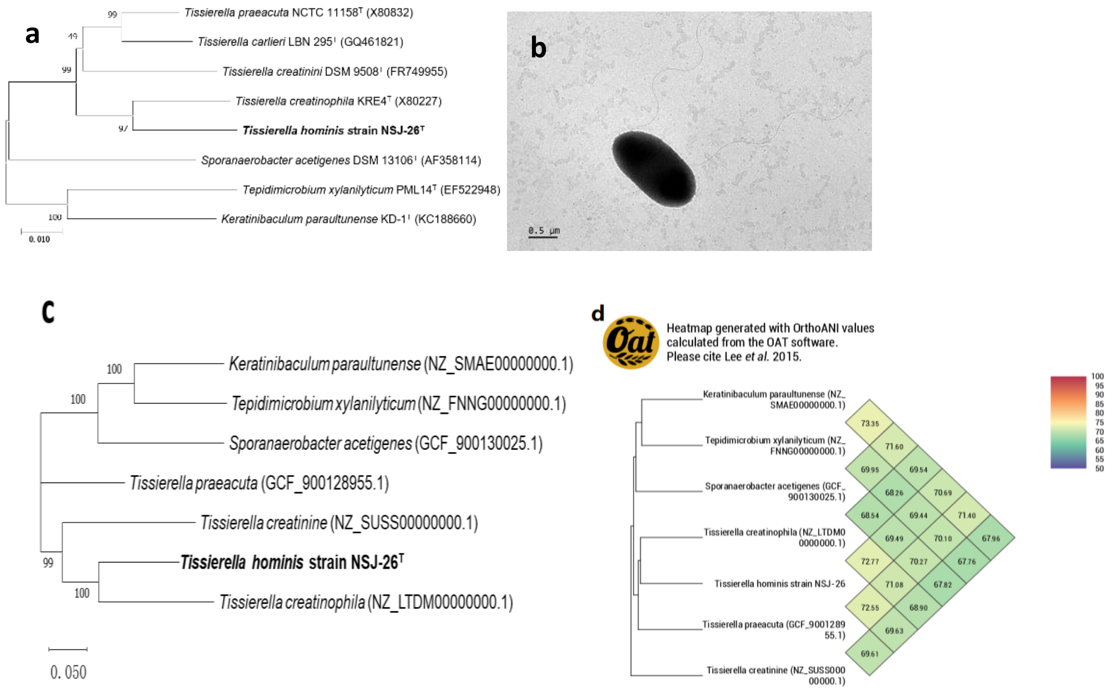

Figure SD-106. The Neighbour-joining phylogenetic tree based on 16S rRNA gene sequences (a) and the cell morphology (b) of strain NSJ-26^T. GenBank accession numbers are given in parentheses. Percentages of bootstrap support are shown at branch nodes. Bootstrap value was 1000. Bar: 0.01 substitutions per nucleotide position. The phylogenominc tree based on gemome-sequence-alignment (c) and OrthoANI heatmap (d) of strain NSJ-26^T and its neighbor species. Refseq ID of representative genomes are given in parentheses. Percentages of bootstrap support are shown at branch nodes. Bar: 0.05 substitutions per nucleotide position.

Taxon 107: ***Fusobacterium hominis*** **sp. nov.**

Description *of Fusobacterium hominis* **sp. nov.** (ho'mi.nis L. gen. masc. n. *hominis*, of a human being, referring to the human gut habitat)

The taxon 107, respresented by strain NSJ-57^T (16S rRNA sequence accession number in NMDC is NMDCN000013A), is phylogenetically closest to *Fusobacterium mortiferum*, with 16S rRNA gene identity of 97.44 %. Phylogenetic tree shows that strain NSJ-57^T clusters with other members of the genus *Fusobacterium,* (Figure SD-107a), suggesting strain NSJ-57^T is a member of the genus *Fusobacterium*. The genome of strain NSJ-57^T was sequenced and the NMDC accession number is NMDC60014103. Genome-based analysis showed that the ANI value of genomes of strain NSJ-57^T and *Fusobacterium mortiferum* DSM 19809^T (NZ CP028102) is 76.88 % and the dDDH estimation is 21.80 %. According to the phylogenominc tree (Figure SD-107c), the ANIs and dDDH values of genomes between strain NSJ-57^T (NMDC accession number is NMDC60014103) and phylogenomically neighbored genomes were calculated, and the highest ANI and dDDH values are 76.97 and 21.8 %, respectively, between the query genome of *Fusobacterium mortiferum* (NZ_CP028102.1);and difference in % G+C between genomes of strain NSJ-57^T and *Fusobacterium mortiferum* (NZ_CP028102.1) is 0.02; OrthoANI heatmap (Figure SD-107d) shows the phylogenomic status of the corresponding neighbor species based on OrthoANI values.Based on these results, we concluded that the strain NSJ-57^T represents of new species of the genus *Fusobacterium*, and the name *Fusobacterium hominis* sp. nov. is proposed.

Cells are strictly anaerobic and spherical in shape (diameter 0.9-1.2 µm, Figure SD-107b); cells appear singly or in (dividing) pairs. Cream-colored, slightly raised, tiny, pinpoint colonies appear on modified mGAM agar plate after 3 days of incubation. Growth occurs at 37°C and at pH 7.0-7.5. The organism uses D-fructose, L-fucose, D-galactose, D-galacturonic acid, gentiobiose, α-D-glucose, glucose-6-phosphate, D,L-α-glycerol phosphate, D-mannose, D-melibiose, 3-melthyl-D-glucose, palatinose, L-rhamnose, glyoxylic acid, α-hydroxybutyric acid, D,L-lactic acid, L-lactic acid, D-lactic acidmethyl ester, pyruvic acid, pyruvic acidmethyl ester, glycyl-L-aspartic acid and inosine as the carbon source for growth, and weakly metabolizes dextrin, D-gluconic acid, lactulose, turanose, acetic acid, ß-hydroxybutyric acid, α-ketobutyric acid, D-saccharic acid and m-tartaric acid. The DNA G+C content of the type strain NSJ-57^T is 29.05 mol %. The type strain NSJ-57^T (=CGMCC 1.32833^T) was isolated from the faeces of a healthy adult.

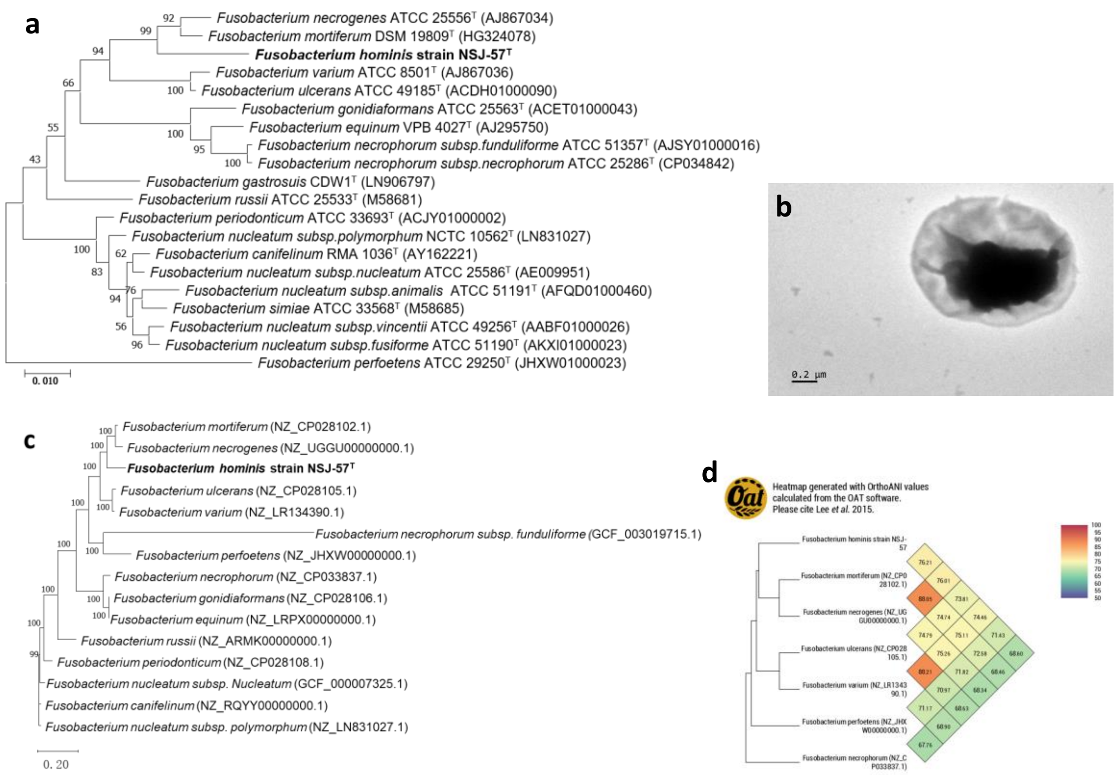

Figure SD-107. The Neighbour-joining phylogenetic tree based on 16S rRNA gene sequences (a) and the cell morphology (b) of strain NSJ-57^{T}. GenBank accession numbers are given in parentheses. Percentages of bootstrap support are shown at branch nodes. Bootstrap value was 1000. Bar: 0.01 substitutions per nucleotide position. The phylogenominc tree based on gemome-sequence-alignment (c) and OrthoANI heatmap (d) of Fusobacterium hominis strain NSJ-57and its neighbor species. Refseq ID of representative genomes are given in parentheses. Percentages of bootstrap support are shown at branch nodes. Bar:0.20 substitutions per nucleotide position.

Taxon 108: *Escherichia hominis* sp. nov.

Description *of Escherichia hominis* sp. nov. (ho'mi.nis L. gen. masc. n. *hominis*, of a human being, referring to the human gut habitat)
The taxon 18, respresented by strain NSJ-73^T (16S rRNA sequence accession number in NMDC is NMDCN000013B), is phylogenetically closest to *Escherichia marmotae*, with 16S rRNA gene identity of 98.88 %. Phylogenetic tree shows that strain NSJ-73^T clusters with other members of the genus *Escherichia,* (Figure SD-108a), suggesting strain NSJ-73^T is a member of the genus *Escherichia*. The genome of strain NSJ-73^T was sequenced and the NMDC accession number is NMDC60014104. Genome-based analysis showed that the ANI value of genomes of strain NSJ-73^T and *Escherichia marmotae* HT073016^T (NZ CP025979) is 91.17 % and the dDDH estimation is 43.60 %. According to the phylogenominc tree (Figure SD-108c), the ANIs and dDDH values of genomes between strain NSJ-73^T (NMDC accession number is NMDC60014104) and phylogenomically neighbored genomes were calculated, and the highest ANI and dDDH values are 89.53 and 40.1 %, respectively, between the query genome of *Escherichia albertii* (NZ_AP014857.1); and difference in % G+C between genomes of strain NSJ-73^T and *Escherichia albertii* (NZ_AP014857.1) is 0.89; OrthoANI heatmap (Figure SD-108d) shows the phylogenomic status of the corresponding neighbor species based on OrthoANI values.Based on these results, we concluded that the strain NSJ-73^T represents of new species of the genus *Escherichia*, and the name *Escherichia hominis* sp. nov. is proposed.
Cells are strictly anaerobic, straight rods (1.2-2.4 µm long by 0.6-0.8 µm wide, Figure SD-108b); cells appear singly. Tiny, semi-translucent, pinpoint colonies appear on modified mGAM agar plate after 4 days of incubation. Growth occurs at 37°C and at pH 7.0-7.5. The organism uses N-acetyl-D galactosamine, acetyl-D glucosamine, N-acetyl-β-D mannosamine, adonitol, amygdalin, D-arabitol, D-cellobiose, α-cyclodextrin, i-erythritol, D-fructose, D-galactose, glycerol, α-D-lactose, D-mannose, α-methyl-D-galactoside, L-lactic acid, m-tartaric acid and urocanic acid as the carbon source for growth, and weakly metabolizes arbutin, β-cyclodextrin, dextrin, dulcitol, D-galacturonic acid, D-gluconic acid, D, L-α-glycerol phosphate, m-Inositol, stachyose, D-melezitose, ß-methyl-D-galactoside, L-rhamnose, D-melibiose, D-sorbitol, sucrose, α-ketovaleric acid, pyruvic acid, D-glucosaminic acid, α-D-glucose,glycyl-L-proline, uridine and thymidine-5'-monophosphate. The DNA G+C content of the type strain NSJ-73^T is 62.38 mol %. The type strain NSJ-73^T (=CGMCC 1.32849^T) was isolated from the faeces of a healthy adult.

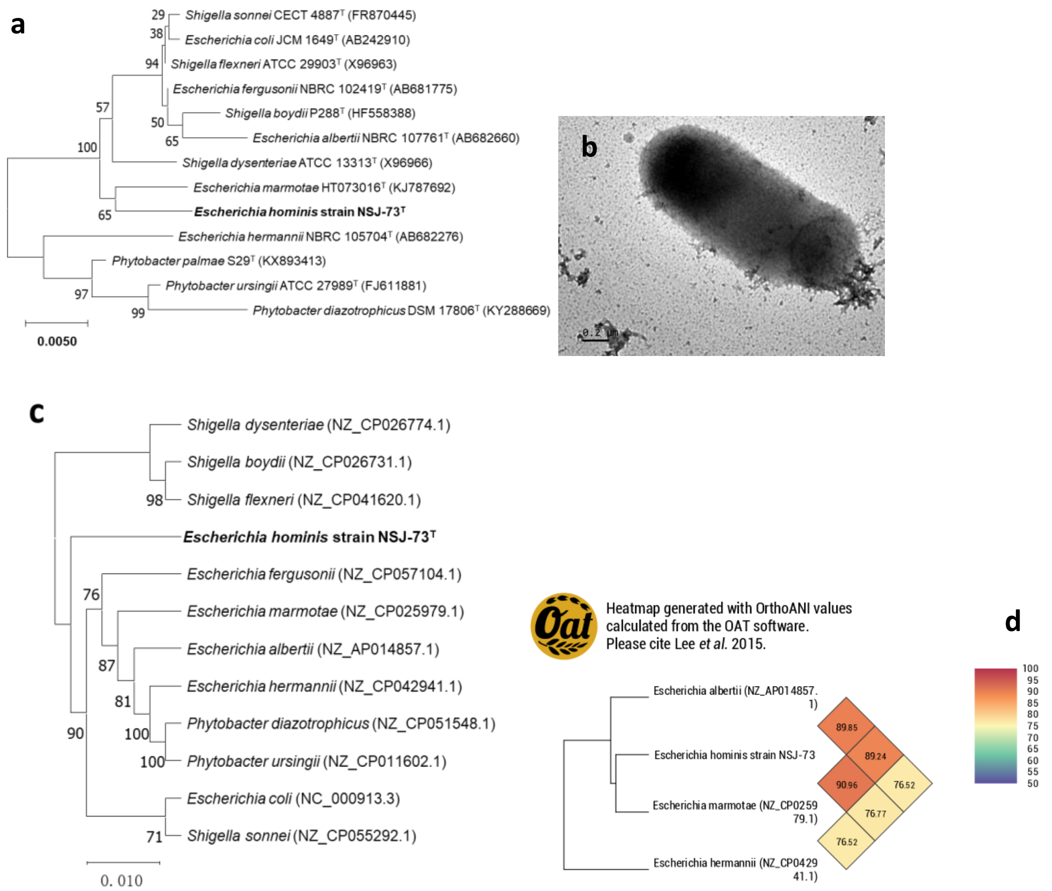

Figure SD-108. The Neighbour-joining phylogenetic tree based on 16S rRNA gene sequences (a) and the cell morphology (b) of strain NSJ-73^T. GenBank accession numbers are given in parentheses. Percentages of bootstrap support are shown at branch nodes. Bootstrap value was 1000. Bar: 0.005 substitutions per nucleotide position. The phylogenominc tree based on gemome-sequence-alignment (c) and OrthoANI heatmap (d) of Escherichia hominis strain NSJ-73^T and its neighbor species. Refseq ID of representative genomes are given in parentheses. Percentages of bootstrap support are shown at branch nodes. Bar:0.010 substitutions per nucleotide position.
